# Methylation Profiles Reveal Distinct Subgroup of Hepatocellular Carcinoma Patients with Poor Prognosis

**DOI:** 10.1371/journal.pone.0104158

**Published:** 2014-08-05

**Authors:** Way-Champ Mah, Thomas Thurnherr, Pierce K. H. Chow, Alexander Y. F. Chung, London L. P. J. Ooi, Han Chong Toh, Bin Tean Teh, Yogen Saunthararajah, Caroline G. L. Lee

**Affiliations:** 1 Department of Biochemistry, Yong Loo Lin School of Medicine, National University of Singapore, Singapore, Singapore; 2 Division of Medical Sciences, Humphrey Oei Institute of Cancer Research, National Cancer Centre Singapore, Singapore, Singapore; 3 NUS Graduate School for Integrative Sciences and Engineering, National University of Singapore, Singapore, Singapore; 4 Duke-NUS Graduate Medical School, Singapore, Singapore; 5 Department of Surgery, Singapore General Hospital, Singapore, Singapore; 6 Department of Surgical Oncology, National Cancer Centre Singapore, Singapore, Singapore; 7 Department of Medical Oncology, National Cancer Centre Singapore, Singapore, Singapore; 8 Cancer Science Institute of Singapore, National University of Singapore, Singapore, Singapore; 9 Department of Hematologic Oncology and Blood Disorders, Taussig Cancer Institute, Cleveland Clinic, Cleveland, Ohio, United States of America; The University of Hong Kong, China

## Abstract

Hepatocellular Carcinoma (HCC) is one of the leading causes of cancer-associated mortality worldwide. However, the role of epigenetic changes such as aberrant DNA methylation in hepatocarcinogenesis remains largely unclear. In this study, we examined the methylation profiles of 59 HCC patients. Using consensus hierarchical clustering with feature selection, we identified three tumor subgroups based on their methylation profiles and correlated these subgroups with clinicopathological parameters. Interestingly, one tumor subgroup is different from the other 2 subgroups and the methylation profile of this subgroup is the most distinctly different from the non-tumorous liver tissues. Significantly, this subgroup of patients was found to be associated with poor overall as well as disease-free survival. To further understand the pathways modulated by the deregulation of methylation in HCC patients, we integrated data from both the methylation as well as the gene expression profiles of these 59 HCC patients. In these patients, while 4416 CpG sites were differentially methylated between the tumors compared to the adjacent non-tumorous tissues, only 536 of these CpG sites were associated with differences in the expression of their associated genes. Pathway analysis revealed that forty-four percent of the most significant upstream regulators of these 536 genes were involved in inflammation-related NFκB pathway. These data suggest that inflammation via the NFκB pathway play an important role in modulating gene expression of HCC patients through methylation. Overall, our analysis provides an understanding on aberrant methylation profile in HCC patients.

## Introduction

Hepatocellular Carcinoma (HCC) is ranked the fifth most commonly diagnosed cancer in men and seventh in women [1]. It is particularly prevalent in Asia, with a majority of the cases diagnosed in China [2]. Presently, the molecular pathogenesis of hepatocellular carcinoma remains elusive. Even though studies have identified TP53 [3], [4], CTNNB1 [5], [6], and AXIN1 [7], [8] to be mutated in HCC, these events remain rare [9], [10]. In addition to genetic abnormalities, studies have begun to focus on epigenetic changes in HCC patients as alternative mechanisms playing roles in hepatocarcinogenesis. Aberrant DNA methylation is one such example. In fact, in early single-gene analysis, tumor suppressor genes including P16 [11], [12], CDH1 and GSTP1 [13], [14] were found to be hypermethylated in HCC. With the advent of high throughput technology, a few genome-wide methylation profiling studies reported CpG dinucleotides to be differentially methylated between tumors and adjacent non-tumorous liver tissues [15], [16], [17], [18], [19], [20], [21], as well as between cirrhotic liver and HCC [22], [23]. Nonetheless, much remains to be studied with regards to the role of aberrant methylation in hepatocarcinogenesis as we have only just begun to unveil the methylome of HCC. The heterogeneity of the methylome amongst different tumor samples is particularly important and is yet to be investigated.

In this study, we investigated the methylome of Asian HCC patients by comprehensively profiling the methylation levels of 59 HCC samples from Singapore. We explored the feasibility of subgrouping the tumors molecularly based on their methylation profiles and determine if any of these subgroups can predict the clinical prognosis of the patients. We also integrated data from both the methylation as well as the gene expression profiles to give us a glimpse of the pathways affected by the deregulation of methylation in HCC patients.

## Materials and Methods

### HCC samples

Fifty nine tumorous and adjacent non-tumorous liver tissues of Hepatocellular Carcinoma patients were obtained from the National Cancer Centre of Singapore (NCCS)/SingHealth Tissue Repository with patients’ written informed consent. Tissue samples were surgically resected, flash-frozen in liquid nitrogen and stored at −80°C until use. All research protocols were approved by the SingHealth Centralized Institutional Review Board (CIRB; approval 2008/440/B).

### DNA extraction and bisulfite treatment

Genomic DNA was extracted from patients’ tissue using QIAamp DNA mini kit (Qiagen, Germany) according to manufacturer’s protocol. Eight hundred nanogram of genomic DNA was bisulfite converted using EZ-96 DNA methylation kit (Zymo Research, USA) for Infinium array and Qiagen Epitect kit (Qiagen, Germany) for pyrosequencing, according to respective manufacturer’s protocol. Purified bisulfite-treated DNA was stored at −20°C until use.

### Methylation profiling

Methylation level of patient’s DNA was quantified using the Infinium HumanMethylation27 BeadChip (Illumina, USA) according to manufacturer’s manual. Replicate samples of one subject were included as quality control for reproducibility of the assay. The Infinium BeadChip contains 27,578 CpG sites, encompassing 14,495 genes for interrogation. CpG probes with detection p-value greater than 0.05 were removed from subsequent analysis as they were not significantly different from the negative control probes and background noise. This array interrogates the methylation status at ∼97% of promoter regions defined as 2 kb around the transcription start site. Methylation values of CpG sites and their associated genomic characteristics were obtained from the Illumina® Genome Studio software (Illumina, USA). Methylation levels were reported as β-values, with a range from 0 to 1. A β-value of zero indicates a low level of methylation, while a β-value close to 1 indicates a high level of methylation. All BeadChip assays were processed at the Duke-NUS Genome Biology Facility, Singapore. Data have been deposited into the Gene Expression Omnibus (GEO) database, under the accession number GSE57956.

### Pyrosequencing

Pyrosequencing was used to validate the methylation profile observations and was carried out according to manufacturer’s protocol using the Pyromark Q24 machine (Qiagen, Germany). 20 out of the 59 patient samples were randomly chosen for validation. Genomic DNA was first bisulfite modified according to Epitect kit protocol (Qiagen, Germany) and its purified product was subsequently amplified using Pyromark PCR kit (Qiagen, Germany). The sequencing primers, biotinylated PCR primers, paired primers and annealing temperature used in the PCR step are listed in Table S1. The sequencing results were analysed and exported by Pyromark CpG software (Qiagen, Germany).

### Gene expression profiling

Gene expression profiling was carried out using RNA extracted by Qiagen RNeasy mini kit (Qiagen, Germany) from 59 patients’ tissue samples using two different microarray platforms. The first batch of twenty samples was profiled as described previously [24]. Briefly, five hundred nanograms of total RNA from each sample were processed and hybridized to Agilent Whole Human Genome Oligo Microarray according to manufacturer’s protocol (Feature number: G4112A, Agilent Technologies, USA). Microarray images were read out using Agilent Feature Extraction Software (Agilent Technologies, USA). The remaining thirty nine samples were assessed using Illumina Human WG-6 expression BeadChip (Illumina, USA). Briefly, seven hundred and fifty nanogram of total RNA from each sample was processed and hybridized to the BeadChip according to the manufacturer’s manual. All BeadChip assays were processed at the Duke-NUS Genome Biology Facility, Singapore. Data have been deposited into the GEO database, under the accession number GSE57957.

### Real-time quantitative PCR

Observations obtained from expression profiling were validated using Bio-Rad CFX96™ real-time PCR detection system (Bio-rad, USA) according to manufacturer’s protocol. Primers and annealing temperatures used in these validation assays are listed in Table S2. Each assay consists of 5 µl of 2X Maxima™ SYBR Green qPCR master mix (Fermentas, USA), 0.2 µM of forward and reverse primers, and 1 µl of 5-times diluted cDNA as template. Real-time PCR reactions were done in triplicates, and threshold cycle numbers (Ct) were determined at the level that showed the best kinetic PCR parameters. No-template control was used as negative control, and melting curves were obtained to confirm specificity of the PCR product. The 2^−ΔΔCt^ method was used to measure the relative quantification of a target gene [25].

### Analysis of methylation data

β-values were first imported into the Partek Genomics Suite (Partek Inc, USA) and then quantile normalized. CpG probes located in sex chromosomes were excluded from the analysis to avoid gender bias. Differentially methylated CpG loci between tumors and adjacent non-tumorous tissues were identified using analysis of variance (ANOVA) method, where p-values generated were subjected to multiple test correction using Benjamini and Hochberg (B-H) method. A CpG site is considered differentially methylated when its false discovery rate (FDR) adjusted p-value is less than 0.05 and change of β-value is greater than 0.1. Hierarchical clustering of the 4416 differentially methylated probes was performed using the Pearson correlation coefficient as a distance metric and the average linkage agglomerative method.

### Analysis and integration of gene expression data

Raw data from Agilent and Illumina microarrays were log2-transformed and subsequently loaded into R session. Annotation packages, namely “lumiHumanAll.db” [26], “lumiHumanIDMapping” [27] and “hgug4112a.db” [28] were used to map array probes to accession numbers. Accession numbers between the two arrays were matched and merged into a single table. To ensure that the datasets generated from the two types of arrays are comparable and can be analysed together meaningfully, batch correction method [29] was performed to remove platform specific effects (Figure S1A). We profiled two patient samples on both arrays and used them for quality assessment of the dataset. As evident in Figure S1B, mapped probes intensities from the two microarray platforms are highly correlated (R^2^≥0.93). Differentially expressed genes between samples were identified based on previously published method [30]. FDR adjusted p-value of less than 0.05 and an absolute fold change of greater than 1.2 were used as cut-offs for analysis. For genes with multiple probes, the probe with the most significant difference was selected. DNA methylation and gene expression data sets were merged using Partek Genomics Suite (Partek Inc, USA) and their correlations were measured using the Pearson correlation coefficient.

### Consensus hierarchical clustering with feature selection

Consensus clustering, a resampling-based class discovery method, is commonly used for clustering of gene expression data [31]. In this study, a modified method called consensus hierarchical clustering with feature selection (CHC-FS) [32] was performed to identify HCC subgroups based on methylation profiles of tumors. The R package ConsensusClusterPlus was used to carry out CHC [33]. CHC applies hierarchical clustering on 80% of all the samples, which was repeated one thousand times, using Pearson correlation as a distance metric between samples. The frequency by which two tumors clustered together in one thousand repeats was recorded as consensus index. Consensus indices of each pair of samples were then visualized as consensus matrix. A value close to zero or one will signify that a pair of tumors almost never or always clustered together in 1000 iterations of clustering. To retain the probes that are more informative towards the tumor subgroups, CpG probes (features) with the most significant difference between subgroups were selected by using a modified limma method [30] with FDR as multiple test correction. Such feature selection improved clustering stability. We repeated the analysis with the consensus k-means clustering method with feature selection to validate the subgroups found by CHC-FS.

### Pathway and gene ontology analysis

Pathway and gene ontology analysis using Ingenuity Pathway Analysis (IPA) software (Ingenuity® Systems, www.ingenuity.com) were performed on shortlisted genes. Biological functions and pathways were deemed statistically enriched when the FDR adjusted p-values were less than 0.05 in Fisher’s exact test. Z-scores and p-values were used to predict potential upstream regulators. Briefly, Z-score is calculated based on the vector of gene expression in an input gene list. A positive Z-score indicates that the upstream regulator is predicted to be “activated” as a result of activation of downstream genes. A negative Z-score on the other hand indicates that it is predicted to be “inhibited” as a result of repression of downstream genes. P-value tests the probability of the genes in the gene list being regulated by an upstream regulator by chance.

### Statistical analysis

Statistical tests and data visualization were performed using either Partek Genomics Suite (Partek Inc, USA) or statistical package in R (www.r-project.org). P-values less than 0.05 were considered significant unless otherwise stated. Fisher’s exact test was used to study the association between clinical variables and tumor subgroups. Survival analysis was done using Kaplan-Meier method with generalised Wilcoxon test. Prognostic factors were evaluated based on Cox proportional hazards model.

## Results

### Characteristics of HCC samples

Fifty-nine HCC patients were recruited into the study and their clinicopathological data is summarized in Table 1. Patients were all Asian, with median age of 65. Among the tumor samples, 90% of them were male. This is consistent with the report given by the Singapore Cancer Registry [34], where HCC was more prevalent in men. About 60% of the patients had been infected with Hepatitis B Virus (HBV) confirming the observation from epidemiological studies which reported HBV infection as a major risk factor for HCC, especially in Asia [35]. Coefficients of determination (R^2^) of methylation values within triplicates were consistently 0.96 (Figure S2). Such high concordance was similarly observed in other studies [17], [22], thus confirming the reproducibility of the array.

**Table 1:** Clinicopathological data for 59 HCC patients.

Parameters	Available Data	Variables	n	%
Age at diagnosis (Median = 65, range 35–85)	59	≥65 years old	31	53
		<65 years old	28	47
Gender	59	Male	53	90
		Female	6	10
HBV status	59	Postive	36	61
		Negative	23	39
Tumor size	59	≥5 cm	33	56
		<5 cm	26	44
Differentiation (Edmonson)	59	I	5	8.5
		II	23	39
		III	26	44
		IV	5	8.5
TNM staging	58	1	32	55
		2	16	28
		3	10	17
Cirrhosis	58	Absent	37	64
		Present	21	36
Tumor multifocality	55	Absent	45	82
		Present	10	18
Tumor encapsulation	53	Absent	35	66
		Present	18	34
AFP level	51	≥100 ng/ml	36	71
		<100 ng/ml	15	29

### Consensus hierarchical clustering reveals distinct tumor subgroups in HCC

**Figure 1:**
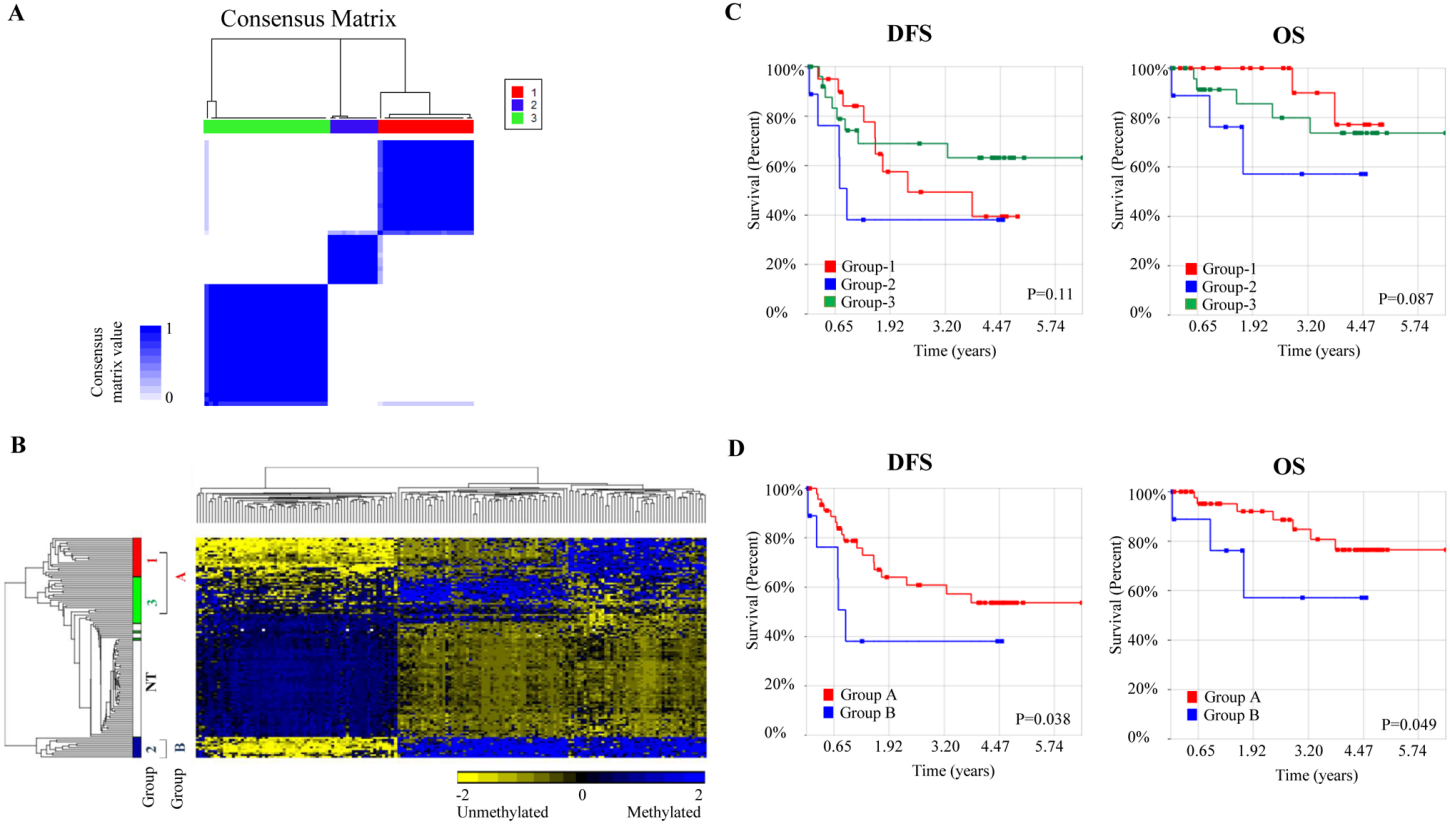
Clustering analysis of 59 HCC tumors reveals 3 subgroups. (A) Consensus matrix (B) 2D hierarchical clustering of 170 probes that overlapped between 4416 differentially methylated CpG sites (between tumors and adjacent non-tumorous tissues) and 199 CpG loci that divided tumors into three subgroups. Subgroups were labeled as Group-1 (red), Group-2 (blue), Group-3 (green) and adjacent non-tumorous tissues, NT (white). Group A represents both Group-1 and Group-3, while Group B is Group-2. (C) Survival curves for the original 3 subgroups identified by CHC-FS. (D) Survival curve for Group B versus Group A. P-value was calculated by generalised Wilcoxon method. OS, overall survival; DFS, disease free survival.

As there is more variability in the methylation profile amongst the tumors of HCC patients compared to the adjacent non-tumorous tissues, we explored if there are subgroups of patients based on their methylation profile that can be correlated with clinicopathological features. Consensus hierarchical clustering with feature selection (CHC-FS) was thus performed on the top 5% most variable CpG probes within the 59 tumors. Three tumor subgroups were identified as evident from the consensus matrix in Figure 1A. K-means consensus clustering [36] confirmed the result as it yielded similar subgroups (Figure S3). 199 probes (features) were found to be sufficient in defining the subgroups (Figure S4). Among 199 probes, 85% (170 CpG probes) were found differentially methylated between tumors and adjacent non-tumorous tissues. The genes associated with 20 of these 170 CpG probes exhibited differential gene expression between the tumors and adjacent non-tumorous tissues (Table S3).

As evident from the heat-map shown in Figure 1B, based on hierarchical clustering, these 170 CpG probes alone can distinguish the 3 subgroups of tumors. The methylation profile of one tumor subgroup, Group-2, was found to be the most distinctly different from the non-tumorous liver tissues out of the 3 subgroups (Figure 1B). Although methylation should conventionally be inversely correlated with gene expression, however, gene expression profiles were not able to distinguish these subgroups.

### Tumor subgroup associated with poorer overall and disease-free survival

As three subgroups of patients were identified based on their methylation profile, we examined whether these subgroups are correlated with clinicopathological characteristics or survival potential. These tumor subgroups were found not to be statistically associated with any of the clinicopathological parameters examined (p-value>0.05, Table S4). Survival analyses, however, revealed that Group-2 patients consistently had worse overall (p-value = 0.087) and disease-free survival (p-value = 0.11) compared to the other subgroups, although the difference was not statistically significant (Figure 1C). To evaluate if Group-2 patients have poorer survival potential compared to the other groups, we reanalysed the data, combining Group-1 and Group-3 patients together to form Group A while Group-2 patients were renamed to Group B. As evident in Figure 1D, Group B patients have significantly poorer overall (p-value = 0.049) and disease-free survival (p-value = 0.038) compared to all the other patients (Group A). Univariate analysis further confirmed this observation. Group B patients were found to have ∼4 times increased risk of death/morbidity compared to Group A (Overall survival: p-value = 0.017; Hazard ratio = 4.73; 95% Confidence interval = 1.32–16.9 and Disease-free survival: p-value = 0.003; Hazard ratio = 3.99, 95% Confidence Interval = 1.61–9.86) (Table 2).

**Table 2:** Univariate analysis of clinicopathological variables for overall survival (OS) and disease-free survival (DFS) in 58 patients (one patient did not have survival information).

Parameters	Variables	OS		DFS	
		HR (95% CI)	p-value*	HR (95% CI)	p-value*
Age at diagnosis	<65 years old	1	0.23	1	0.68
	≥65 years old	2.6 (0.55–12.25)		0.84 (0.36–1.96)	
HBV status	Postive	1	0.95	1	0.97
	Negative	0.96 (0.27–3.42)		0.99 (0.42–2.31)	
Tumor size	Lower quartile	1	**0.006**	1	**0.021**
	Upper quartile	3.74 (1.45–9.65)		2.17 (1.13–4.20)	
Differentiation (Edmonson)	I, II	1	0.38	1	0.41
	III, IV	0.57 (0.16–2.01)		0.70 (0.30–1.63)	
TNM staging	1	1	**0.034**	1	**0.025**
	2, 3	4.37 (1.12–17.05)		2.66 (1.13–6.27)	
Cirrhosis	Absent	1	0.067	1	0.062
	Present	3.31 (0.92–11.89)		2.23 (0.96–5.17)	
AFP level	<100 ng/ml	1	0.102	1	**0.045**
	≥100 ng/ml	3.02 (0.80–11.43)		2.67 (1.02–6.98)	
Tumor encapsulation	Absent	1	0.936	1	0.973
	Present	0.95 (0.24–3.78)		1.02 (0.4–2.55)	
Tumor multifocality	Absent	1	0.887	1	0.754
	Present	1.16 (0.14–9.36)		1.22 (0.36–4.18)	
Group	A	1	**0.017**	1	**0.003**
	B	4.73 (1.32–16.91)		3.99 (1.61–9.86)	

*p-values less than 0.05 were in bold.

### Differentially methylated CpG loci between tumors and adjacent non-tumorous tissues

**Figure 2:**
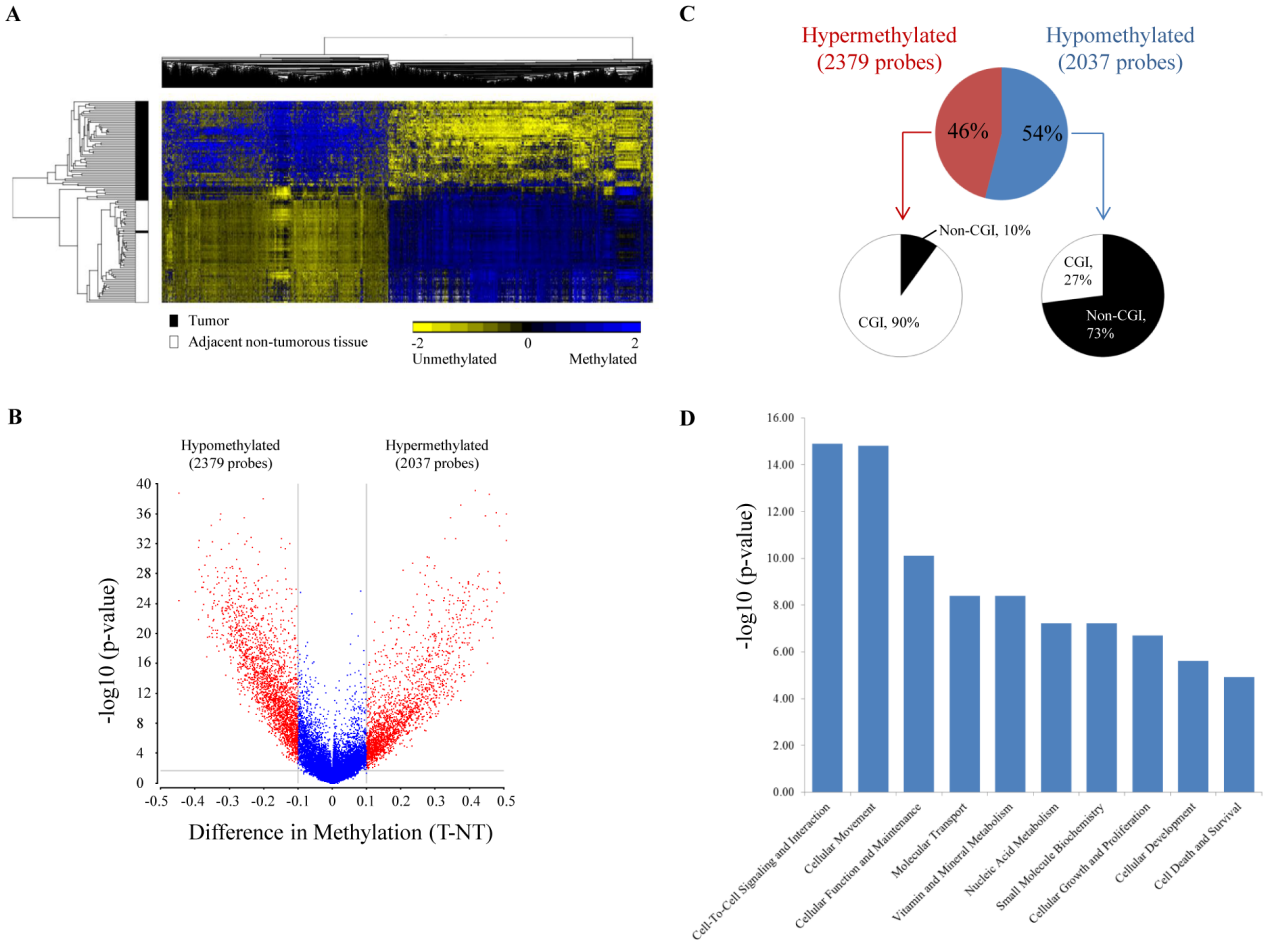
Methylation profiling of 59 HCC patients. (A) Hierarchical clustering of 4416 most significantly differentially methylated CpG probes between tumor and adjacent non-tumorous tissues. (B) Volcano plot for DNA methylation profiles of 59 patients. Y-axis indicates the minus log10 of p-value for each probe, and X-axis shows the mean methylation difference between tumor and adjacent non-tumor. CpG sites that were statistically significant (FDR adjusted p-value<0.05) and had |Δβ|>0.1, were labeled in red. (C) Characteristics of differentially methylated CpG sites. Percentage of significantly hyper- or hypo-methylated CpG sites located in CGI were compared against overall CpG sites in Infinium HumanMethylation27 BeadChip. (D) Top 10 biological functions that are most significantly enriched in our dataset using IPA. X-axis shows the minus log10 of FDR adjusted p-value for Fisher’s exact test.

Differentially methylated CpG sites were identified between the tumor and the adjacent non-tumorous tissues. Analysis of variance (ANOVA), with FDR adjusted p-value of less than 0.05 was employed to analyse the significance of differences between the methylation profiles of tumors versus non-tumorous tissues. With a minimum mean β-value difference of 0.1, 4416 CpG sites were identified to be differentially methylated. Using hierarchical clustering, these 4416 probes were able to clearly distinguish tumors from non-tumorous tissues, except for one tumor tissue which clustered together with non-tumorous tissues (Figure 2A). 54% of the probes (2379 probes) were hypomethylated, while 46% (2037 probes) were hypermethylated in tumors (Figure 2B, C). Frequently methylated genes in HCC such as P16/CDKN2A [11], [12], CDH1 and GSTP1 [13], [14] were also found to be similarly hypermethylated in our tumor samples (Table S5). Further characterization of these sites revealed that ∼90% (p-value = 0.004) of the hypermethylated probes were localized at CpG islands (CGI) while only 27% (p-value<0.0001) of the hypomethylated probes were found to reside within CGI (Figure 2C) suggesting that hypermethylation tends to occur primarily in CGI regions while hypomethylation tends to occur outside CGI. Such distribution has been consistently observed in other studies as well [17], [22], [37].

Ingenuity Pathway Analysis (IPA) was employed to further elucidate the possible biological functions of the genes associated with aberrant methylation. As shown in Figure 2D, genes associated with aberrant methylation are primarily genes involved in cell-to-cell signaling (FDR adjusted p-value = 1.28×10^−15^), cellular movement (FDR adjusted p-value = 1.52×10^−15^), cellular function and maintenance (FDR adjusted p-value = 7.88×10^−11^). Detailed gene sets that are aberrantly methylated are listed in Table S6.

### Pyrosequencing confirms aberrant methylation

**Figure 3:**
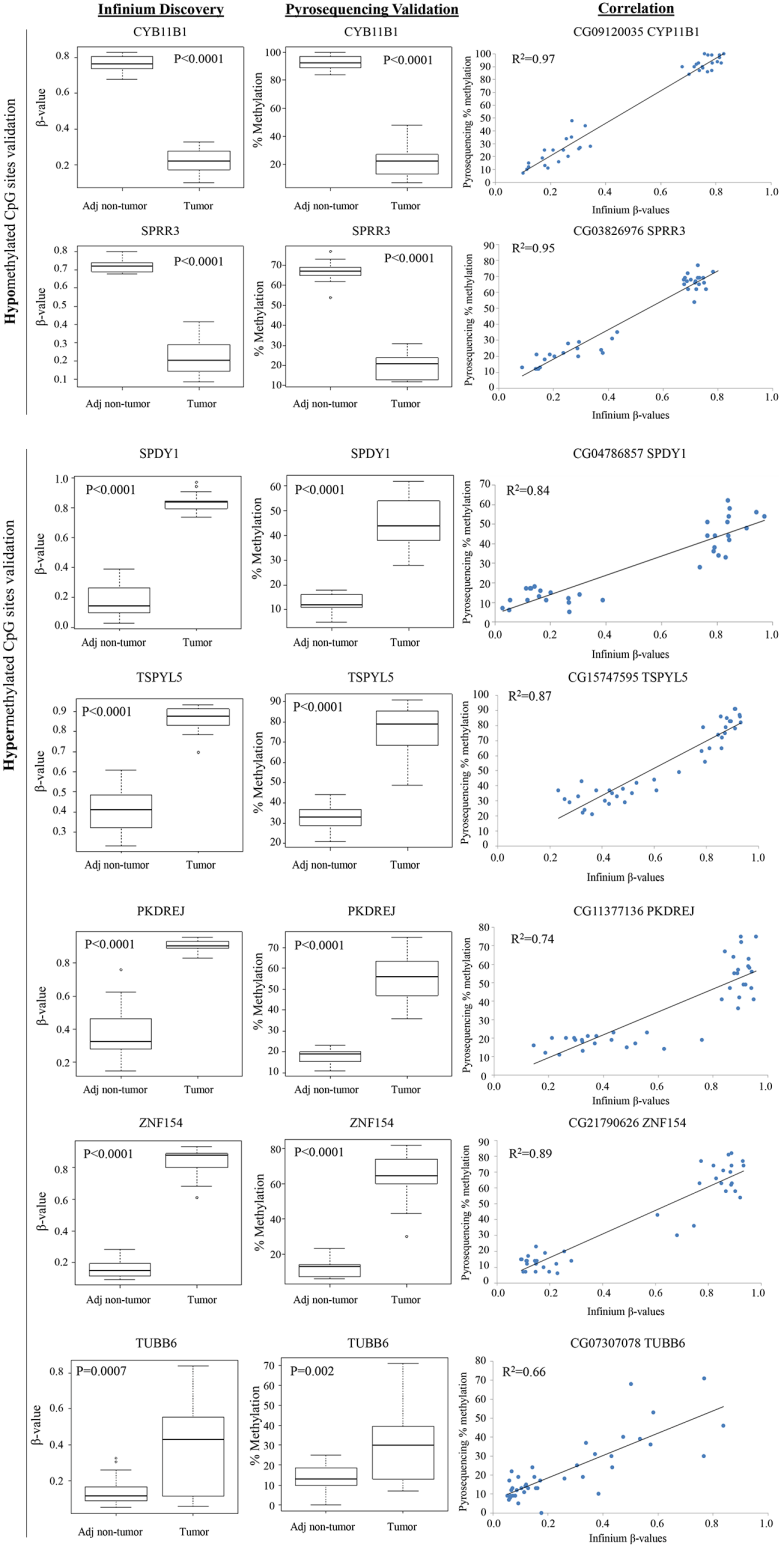
Validation of aberrantly methylated genes in tumors compared to adjacent non-tumorous tissues. Validation of methylation data was done using pyrosequencing. T-test was used to test the difference between tumors and adjacent non-tumorous tissues. Correlation between Infinium’s β-values and pyrosequencing’s percentage of methylation was measured using Pearson’s method. R^2^ is the squared value of the Pearson correlation coefficient.

To validate results obtained from Infinium HumanMethylation27 BeadChip, an independent method, pyrosequencing, was employed to determine the methylation status of two hypomethylated (CYB11B1 and SPRR3) and seven hypermethylated (SPDY1, TSPYL5, PKDREJ, ZNF154, TUBB6, CYB5R2 and SH3YL1) genes. These genes were selected primarily based on their statistical significance and availability of optimised primers for pyrosequencing. As shown in Figure 3, pyrosequencing successfully confirmed the aberrant methylation of these genes in the direction observed on the Infinium BeadChip. To evaluate the robustness of the analysis, TUBB6, which is ranked very low based on significance (774^th^, FDR adjusted p-value<0.001), was included in the validation. As evident from Figure 3, aberrant methylation was clearly observed for TUBB6 by pyrosequencing with a modest p-value of 0.002. Overall, β-values determined by the Infinium BeadChip correlated well with data from pyrosequencing (R^2^ values range from 0.66 to 0.97, Figure 3).

### Integrative analysis reveals that the NFκB pathway plays a central role in modulating gene expression of HCC patients through methylation

**Figure 4:**
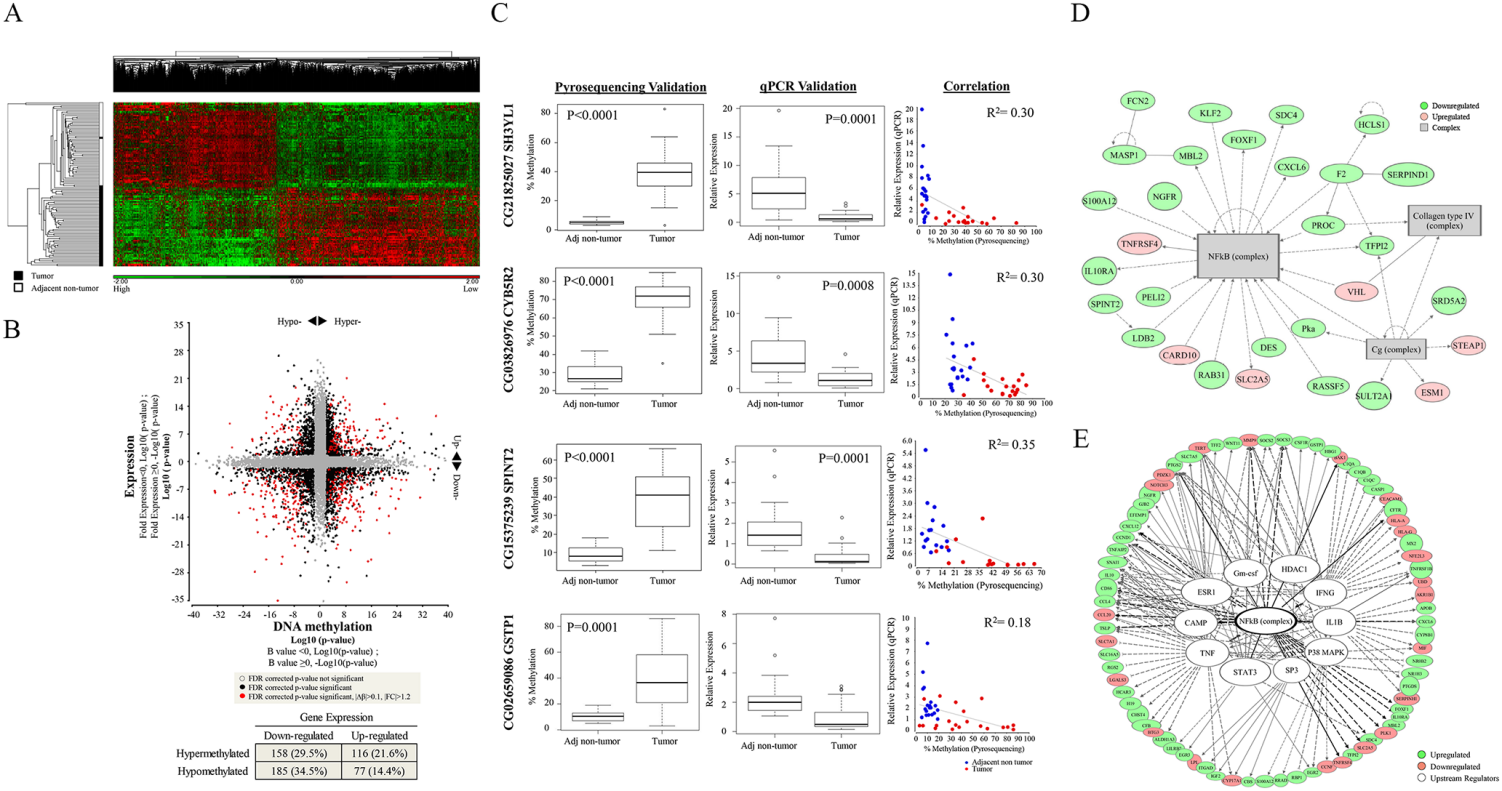
Integrated analysis of methylation and gene expression in 59 HCC patients. (A) Hierarchical clustering of the 3185 most significantly differentially expressed genes between tumors and adjacent non-tumorous tissues. (B) Starburst plot was constructed by plotting transformed log10 p-value of differentially expressed genes (Y-axis) versus transformed log10 p-value of methylation difference (X-axis) between tumor and adjacent non-tumorous tissues. Genes with FDR adjusted p-value<0.05 were labeled in black. Genes with absolute fold change >1.2, difference in β-value greater than 0.1 and which met the statistical cut-off (FDR adjusted p-value<0.05) were labeled in red. Directional change of expression and methylation are indicated by the black arrow head. Table at lower panel shows the percentage of genes with significant positive and negative correlations between gene expression and methylation data. (C) Validation results for SH3YL1, CYB5R2, SPINT2 and GSTP1. Methylation and gene expression data were validated by pyrosequencing and quantitative PCR respectively. T-test was used to compare the difference in methylation or gene expression between two groups; Pearson correlation was used to measure association between pyrosequencing and Infinium data, and between gene expression and methylation data. (D) Top network derived from the 536 aberrantly methylated and deregulated genes where NFκB complex served as primary node. (E) Predicted upstream regulators from IPA. Eleven upstream regulators (including NFκB complex) were associated with the NFκB pathway. Red spheres indicate genes that were upregulated in tumor; green spheres indicate downregulated genes in tumor compared to adjacent non-tumorous tissues; grey boxes represent complexes and white spheres represent upstream regulators.

To elucidate the genes and pathways deregulated through aberrant methylation, we integrated data from the methylation profiles with those from the gene expression profiles. Using FDR adjusted p-value of less than 0.05 and fold change of 1.2 as threshold criteria, 3185 genes were identified to be differentially expressed between the tumors and adjacent non-tumorous tissues (Figure 4A). About 17% of these genes (536/3185 genes, Table S7) were found to be associated with aberrant methylation. Among the 536 genes, almost half of them showed inverse correlation with methylation (44%, Figure 4B), consistent with the conventional association between gene expression and methylation. The other 56% of the genes were positively correlated, suggesting that the regulation of gene expression may be more complex and may involve other epigenetic and non-epigenetic mechanisms [38], [39].

Four potential tumor suppressor genes which were amongst the top 15 significantly hypermethylated genes (based on FDR-adjusted p-value) with corresponding down-regulated gene expression were selected for further validation. Two of these, GSTP1 [13], [40] and SPINT2 [41], [42] were previously reported tumor suppressor genes which were hypermethylated in tumors with corresponding down-regulated gene expression, while the other 2 (CYB5R2 and SH3YL1) represent potential novel tumor suppressor genes. Quantitative real-time PCR and pyrosequencing successfully validated all four genes (Figure 4C). Liver cancer cell lines expressing CYB5R2 were found to grow slower compared to control cells (Figure S5).

The top biological functions associated with these aberrantly methylated and deregulated genes were similar to those identified for aberrantly methylated genes only as described earlier. Cellular movement (FDR adjusted p-value = 5.17×10^−12^), cell-to-cell signaling and interaction (FDR adjusted p-value = 7.80×10^−7^), cellular function and maintenance (FDR adjusted p-value = 2.48×10^−5^) were similarly enriched in this dataset (Table S8). Interestingly, the top network associated with these 536 aberrantly methylated and deregulated genes was found to have NFκB as its primary node (Figure 4D). Notably, when we investigated the potential regulators of these 536 deregulated genes, the NFκB complex was again found to be the most significantly enriched upstream regulator (44% or 11/25, p-value<0.05) (Figure 4E, Table S9).

## Discussion

Genome-wide DNA methylation profiles (GWMP) may serve as a promising useful tool to subtype tumors for correlation with clinical characteristics and/or outcomes. Thus far, GWMP has been successfully employed to identify subgroups of breast cancer [36] and glioma [43] patients with different survival outcomes. However, GWMP has yet to be employed to identify subgroups of HCC patients.

In this study, GWMP was employed to identify a subgroup of HCC patients (Group B) who have worse disease-free as well as overall survival compared to the other patients. Notably, several genes such as ALX4 [44], CHD5 [45], MYOD1 [46], NEUROG1 [47], and RASSF5 [48], whose methylation profile distinguishes this group of HCC patients from the other HCC patients, were previously reported to be similarly hypermethylated and associated with poor prognosis in colorectal cancer (Table S3).

Our data is consistent with the multistep process of HCC where methylation events accumulate as the disease progresses [49], [50]. All 170 methylation probes of Group B patients with poorer disease-free and overall survival were found to be distinctly different from the methylation profiles of non-tumorous liver tissues. On the other hand, patients in Group A have subsets of 170 methylation probes with similar methylation profile as the non-tumorous tissues, thus leading to better disease-free and overall survival profile. Hence, GWMP may supplement existing strategies in the molecular characterization of HCC to classify groups of patients with different prognostic outcomes.

There are currently numerous studies examining CpG sites which were differentially methylated between the tumor and adjacent non-tumorous tissues in HCC patients (please see review [51]). However, these studies do not integrate GWMP with gene expression profiles to identify genes and pathways that are deregulated through aberrant methylation. Integrating methylation with gene expression, we found that cellular movement and cell-to-cell signaling and interaction were highly enriched among genes that were deregulated by aberrant methylation.

Notably, the top network associated with aberrantly methylated and deregulated genes, as well as potential regulators of these aberrantly methylated and deregulated genes were centered on the NFκB complex. As the NFκB pathway was found to be a pivotal link between inflammation and cancer [52] and HCC is a typical inflammation-associated cancer [53], our data suggest that the inflammatory process during hepatocarcinogenesis may deregulate genes associated with the NFκB pathway through aberrant methylation. This is consistent with the observation that aberrant CpG methylation is often seen during chronic inflammation and in precancerous lesions, suggesting that aberrant methylation may be an early event in tumorigenesis which could serve as a useful tumor biomarker [54]. A recent study reported that hepatitis virus infection could activate innate immune response and lead to alterations in DNA methylation in chimeric mice with humanized livers [55]. NFκB was also found to be induced in these hepatitis-infected samples [55]. It may thus be worthwhile to further explore the link between inflammation, aberrant methylation and the NFκB pathway to facilitate the rational design of therapeutic strategies targeting methylation and the NFκB pathway for HCC treatment.

### Conclusions

In conclusion, genome-wide methylation profiling facilitated the identification of a subgroup of HCC patients with poorer prognosis which can potentially serve as a prognostic biomarker. Integration of genome-wide methylation and gene expression profiles highlighted the NFκB pathway as the central pathway associated with aberrant methylation paving the way for further elucidation of the link between inflammation, methylation and the NFκB pathway to facilitate the development of novel therapeutic strategies for HCC.

## Supporting Information

Figure S1 **Quality assessment of batch correction between two microarrays.** (A) PCA plots for tumor (T) and adjacent non-tumorous tissues (NT) before (top) and after (bottom) batch correction. The variance caused by difference in profiling microarrays was removed through batch correction. (B) Correlations of batch corrected and quantile normalized log2 intensities of the same patient sample profiled with different microarrays. R^2^ values range from 0.93 to 0.96. This indicates that although different microarrays were used, the biological variance within the same sample was still preserved after batch correction.
(TIF)

Figure S2 **Quality assessment of reproducibility of Illumina HumanMethylation27 BeadChips.** Sample 43T was repeated 3 times and correlations between replicates were measured. R^2^ is the squared value of the Pearson correlation coefficient.
(TIF)

Figure S3 **Heatmap of K-means consensus clustering matrices after feature selection.** Three subgroups were observed.
(TIF)

Figure S4 **Hierarchical clustering of tumors using the probes identified in CHC-FS.** 3 subgroups were identified and labeled as Group-1 (red), Group-2 (blue) and Group-3 (green). Group A represents both Group-1 and Group-3, while Group B is Group-2.
(TIF)

Figure S5 **Characterization of CYB5R2 in liver cell lines.** Experimental validation of (A) methylation levels, (B) transcript levels, (C) protein levels of CYB5R2 in respective liver cell lines. (D) Cells infected with adenoviral vector carrying control and CYB5R2 gene were monitored under microscope and images were captured every 2 hours to track their proliferation rate based on the surface area of zsGreen fluorescence. Y-axis is the difference in zsGreen area between time zero and the time when the next image was taken; X-axis is the number of hours after 24 hours post infection. *t-test, p-value<0.05. (E) Representative cell images at 24 and 48 hours post infection.
(TIF)

Table S1 **Primers used in pyrosequencing.**
(PDF)

Table S2 **Primers used for quantitative real-time PCR.**
(PDF)

Table S3 **170 differentially methylated CpG loci that were selected in Consensus Hierarchical Clustering with feature selection.** 20 out of 170 genes have differential expression between tumor and adjacent non-tumorous tissues.
(PDF)

Table S4 **Correlation between tumor subgroups and clinicopathological parameters in HCC samples.** Fisher’s exact test was used to test the correlation between tumor subgroups and clinicopathological parameters.
(PDF)

Table S5 **4416 differentially methylated CpG loci between tumors and adjacent non-tumorous tissues.**
(PDF)

Table S6 **IPA results for top biological functions enriched in differentially methylated dataset.**
(PDF)

Table S7 **536 genes with aberrant methylation and associated change of expression.**
(PDF)

Table S8 **IPA results for top biological functions enriched in 536 genes with differential methylation and associated expression change.**
(PDF)

Table S9 **Potential upstream regulators predicted by Ingenuity® knowledge base.** Z-score was computed based on the direction change of gene expression in input dataset. Overlap p-value tests the probability of having the targets of upstream regulator in our input dataset by chance.
(PDF)
