# Correction: Methylation Profiles Reveal Distinct Subgroup of Hepatocellular Carcinoma Patients with Poor Prognosis

**DOI:** 10.1371/journal.pone.0146690

**Published:** 2016-01-11

**Authors:** Way-Champ Mah, Thomas Thurnherr, Pierce K. H. Chow, Alexander Y. F. Chung, London L. P. J. Ooi, Han Chong Toh, Bin Tean Teh, Yogen Saunthararajah, Caroline G. L. Lee

In Table 1, there are errors in the AFP row. The number of patients (n) with AFP levels <100ng/ml should be 36, and the n with AFP >100ng/ml should be 15. Please view the corrected Table 1 here.

**Table 1:** Clinicopathological data for 59 HCC patients.

Parameters	Available Data	Variables	n	%
Age at diagnosis (Median = 65, range 35–85)	59	≥65 years old	31	53
		<65 years old	28	47
Gender	59	Male	53	90
		Female	6	10
HBV status	59	Postive	36	61
		Negative	23	39
Tumor size	59	≥5 cm	33	56
		<5 cm	26	44
Differentiation (Edmonson)	59	I	5	8.5
		II	23	39
		III	26	44
		IV	5	8.5
TNM staging	58	1	32	55
		2	16	28
		3	10	17
Cirrhosis	58	Absent	37	64
		Present	21	36
Tumor multifocality	55	Absent	45	82
		Present	10	18
Tumor encapsulation	53	Absent	35	66
		Present	18	34
AFP level	51	>100ng/ml	15	71
		<100ng/ml	36	29
