# Supplementary figures and images for: Methylation Profiles Reveal Distinct Subgroup of Hepatocellular Carcinoma Patients with Poor Prognosis
Source: PLoS One. 2014 Aug 5;9(8):e104158. doi: 10.1371/journal.pone.0104158 (PMC4122406; doi:10.1371/journal.pone.0104158)

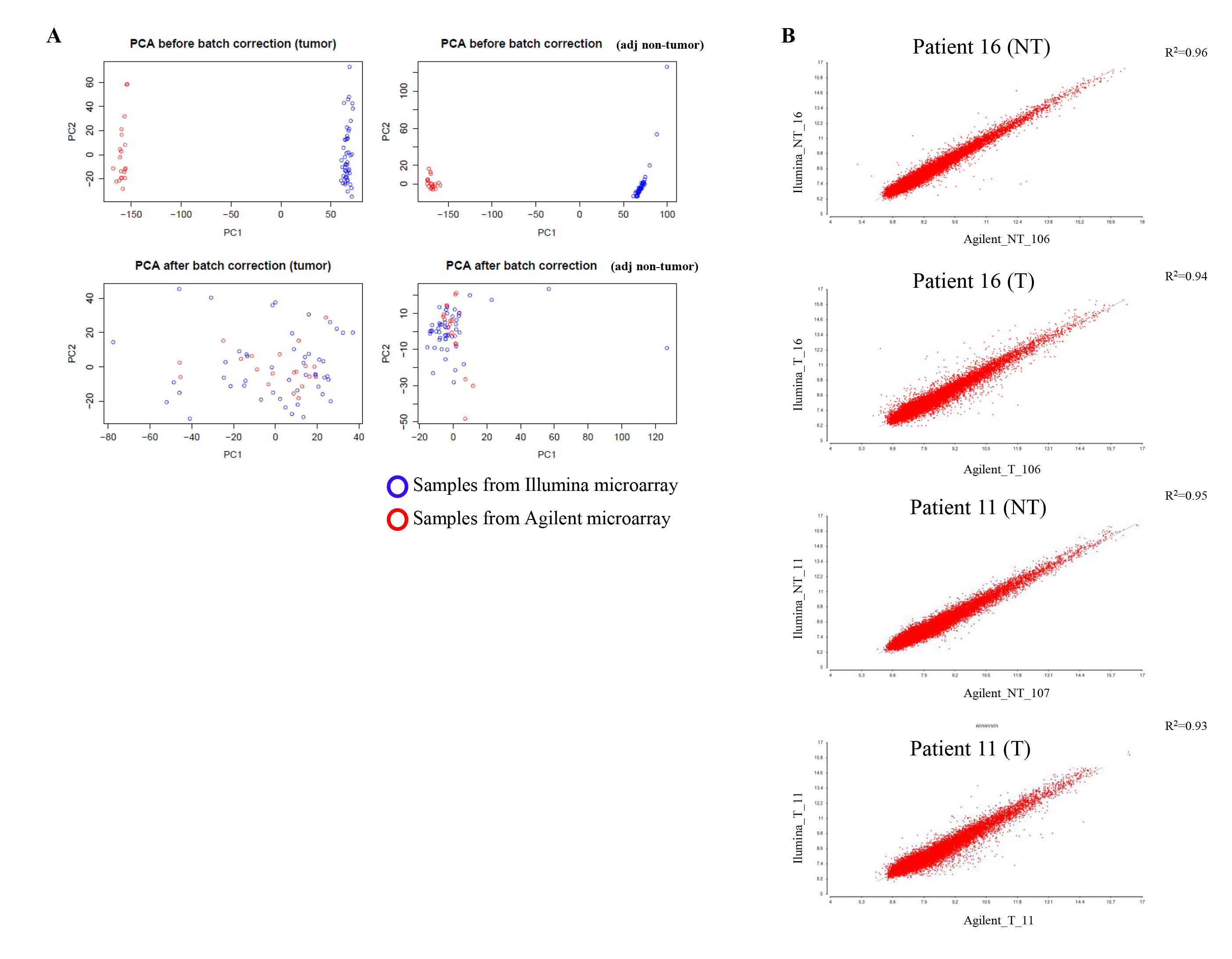

Supplement: Figure S1 — Quality assessment of batch correction between two microarrays. (A) PCA plots for tumor (T) and adjacent non-tumorous tissues (NT) before (top) and after (bottom) batch correction. The variance caused by difference in profiling microarrays was removed through batch correction. (B) Correlations of batch corrected and quantile normalized log2 intensities of the same patient sample profiled with different microarrays. R2 values range from 0.93 to 0.96. This indicates that although different microarrays were used, the biological variance within the same sample was still preserved after batch correction. (TIF) [file pone.0104158.s001.tif]

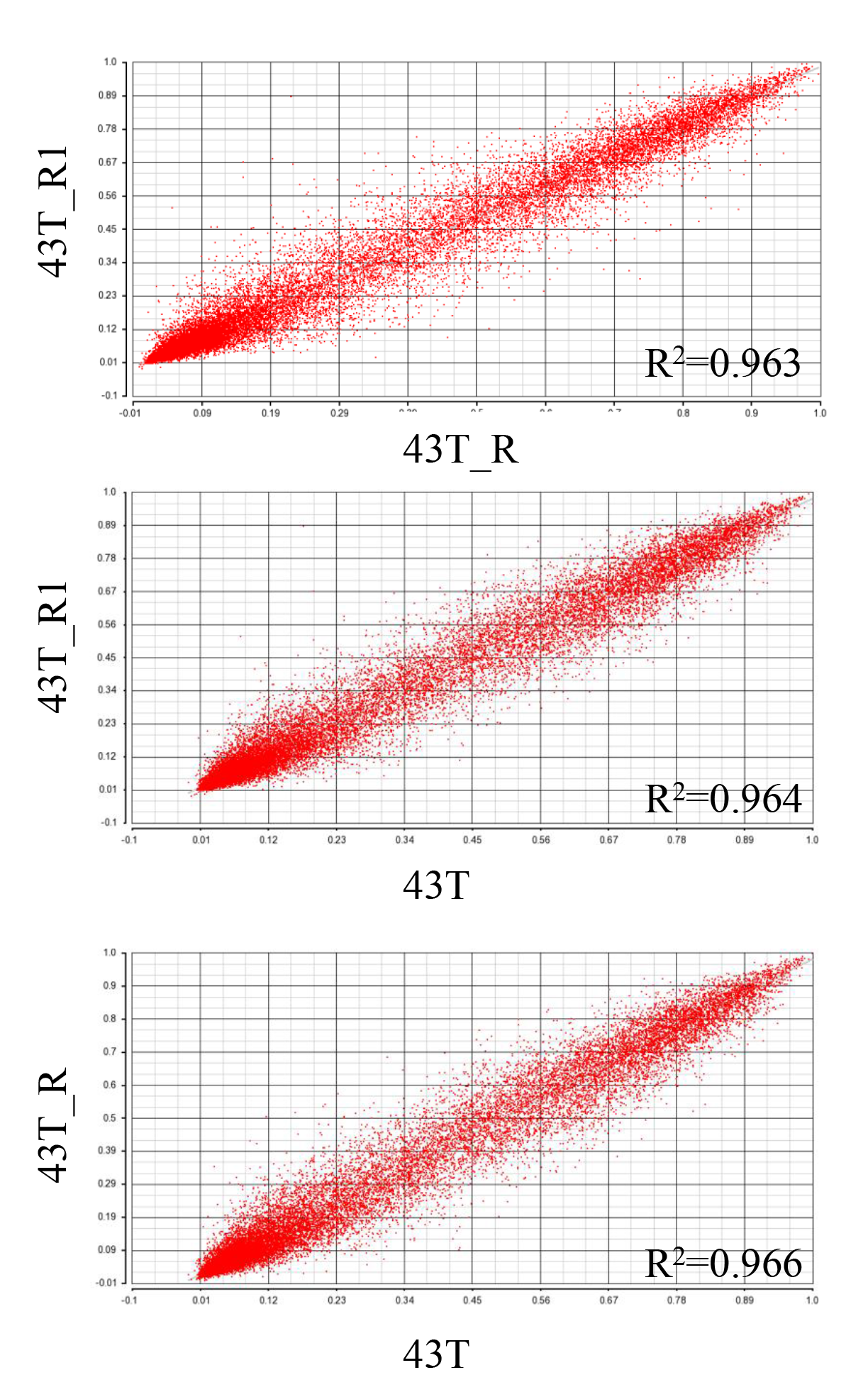

Supplement: Figure S2 — Quality assessment of reproducibility of Illumina HumanMethylation27 BeadChips. Sample 43T was repeated 3 times and correlations between replicates were measured. R2 is the squared value of the Pearson correlation coefficient. (TIF) [file pone.0104158.s002.tif]

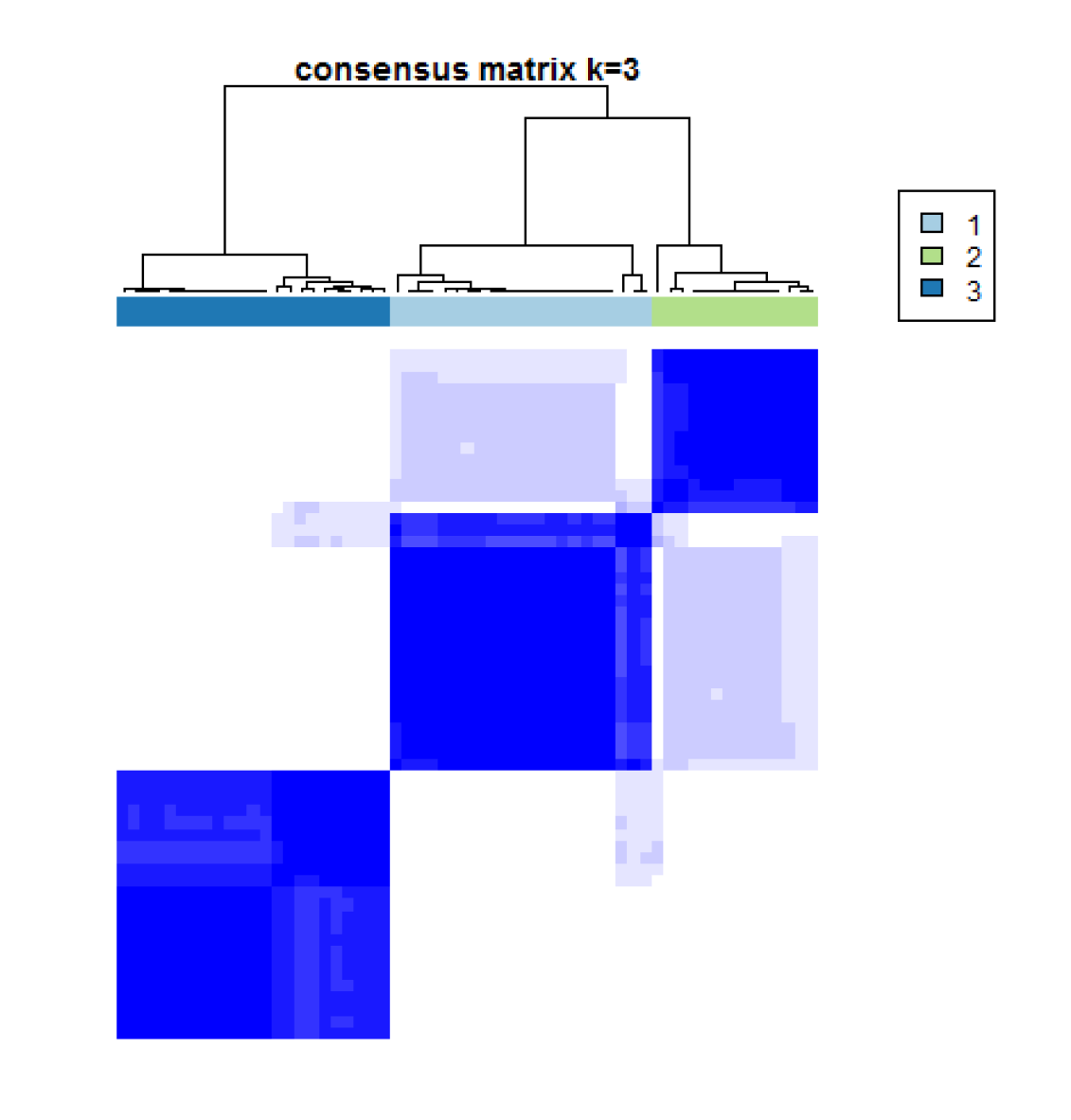

Supplement: Figure S3 — Heatmap of K-means consensus clustering matrices after feature selection. Three subgroups were observed. (TIF) [file pone.0104158.s003.tif]

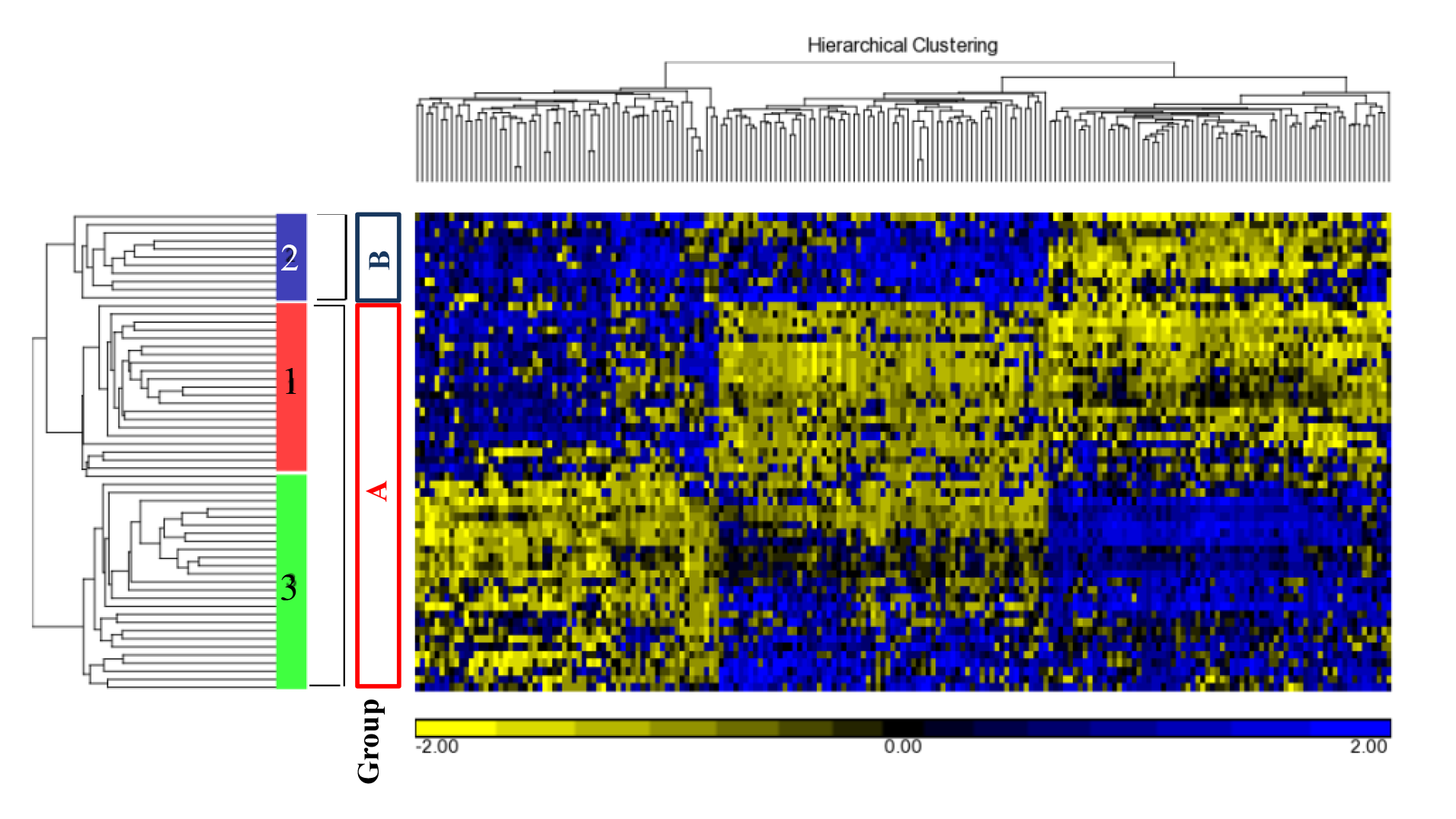

Supplement: Figure S4 — Hierarchical clustering of tumors using the probes identified in CHC-FS. 3 subgroups were identified and labeled as Group-1 (red), Group-2 (blue) and Group-3 (green). Group A represents both Group-1 and Group-3, while Group B is Group-2. (TIF) [file pone.0104158.s004.tif]

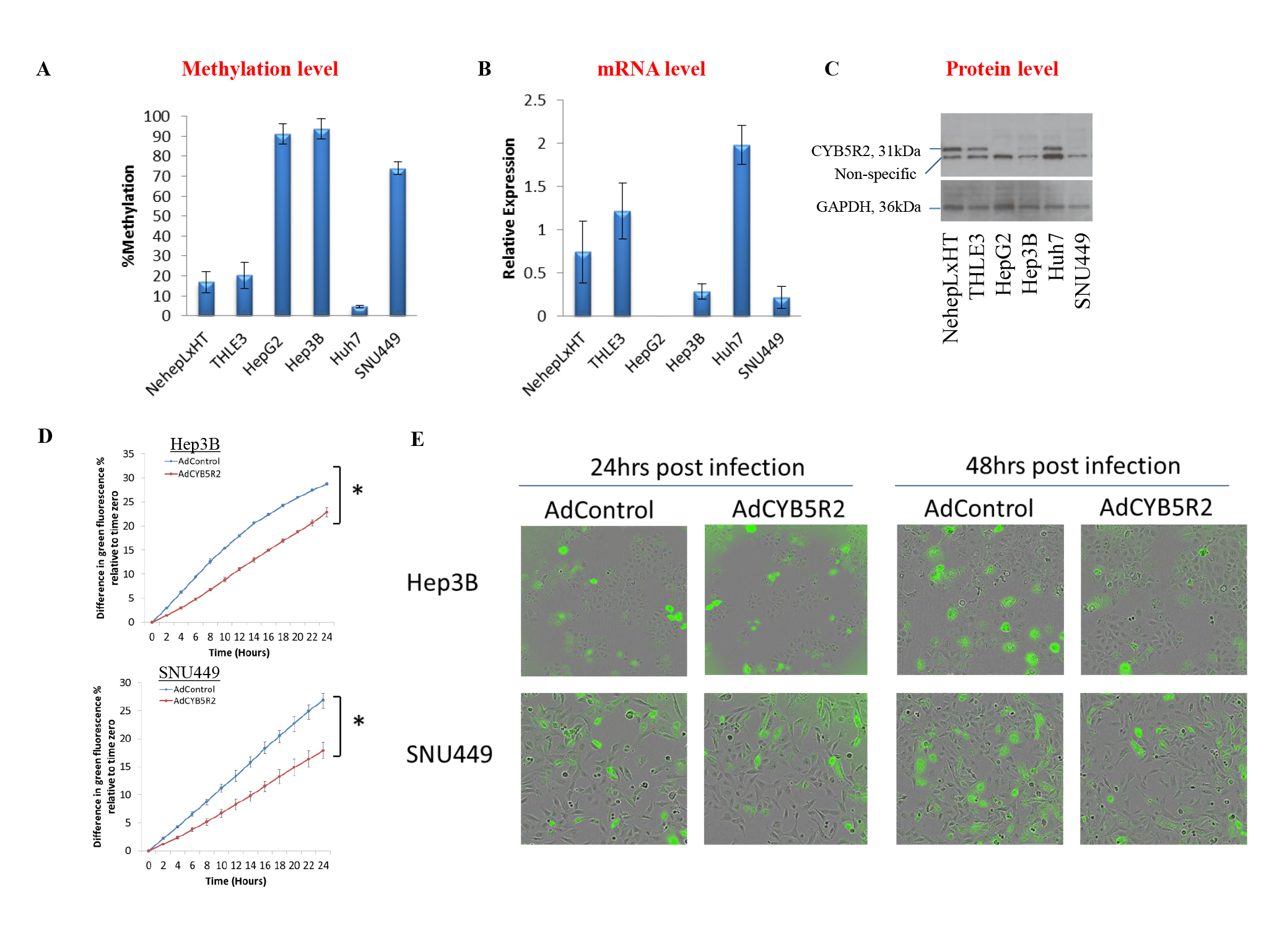

Supplement: Figure S5 — Characterization of CYB5R2 in liver cell lines. Experimental validation of (A) methylation levels, (B) transcript levels, (C) protein levels of CYB5R2 in respective liver cell lines. (D) Cells infected with adenoviral vector carrying control and CYB5R2 gene were monitored under microscope and images were captured every 2 hours to track their proliferation rate based on the surface area of zsGreen fluorescence. Y-axis is the difference in zsGreen area between time zero and the time when the next image was taken; X-axis is the number of hours after 24 hours post infection. *t-test, p-value<0.05. (E) Representative cell images at 24 and 48 hours post infection. (TIF) [file pone.0104158.s005.tif]
