# Supplementary material for: Methylation Profiles Reveal Distinct Subgroup of Hepatocellular Carcinoma Patients with Poor Prognosis
Source: PLoS One. 2014 Aug 5;9(8):e104158. doi: 10.1371/journal.pone.0104158 (PMC4122406; doi:10.1371/journal.pone.0104158)
Supplement: Table S1 — Primers used in pyrosequencing. (PDF) [file pone.0104158.s006.pdf]

Table S1. Primers used in pyrosequencing.

Gene Symbol	Forward Primer	Reverse Primer	Sequencing Primer	Annealing Temperature (^{o}C)
CYB5R2	GATTAGGTTTAGGGTAGG	Biotin-CCTAACCCCTTCCCTACACA	TGAGGTATGTAGAATGAATATA	57
CYP11B1	GGGTAAAGGTAGAGGTGTGTATG	Biotin-ACAACAACCTCAACCACCTATT	TGTAAAGGGTATAGGTATTG	55
GSTP1	TTGTTTTGTGAAGYGGGTGTG	Biotin-TTTCCCTCTTTCCCAAATCC	GGTTTTAGGGAATTTTT	55
PKDREJ	AGGAGGAAGGTTTAGGTTAGG	Biotin-ACCTCTACTTCCCCCATA	GTAAGTAGAGGTAGTATTAG	55
SH3YL1	TTGGGTTTGGGAGTTTTTTGAGAT	Biotin-AAATAAATCCCCCAATACTCACTACTAC	TTTGAGGGAGAAGGTTTGA	55
SPDY1	Biotin-GGGTTGGAGGGAGATTTTAGAG	AAACTAAAACCCTACCTTCTCATCT	ACTAAAACCCTACCTTCTCATCTAA	55
SPINT2	TGAGGGTAGTTGAGTGT	Biotin-ATACCTAAATCTACTCCTCACTC	TAAGGGAAGGGTGGTAGG	55
SPRR3	ATGGTTGAAGTGGATATGGAAATAATAA	Biotin-AATTCACTTATTCTACACCATCC	AATAATAATAGGTATTTTTGGTT	59
TSPYL5	Biotin-GAGGTTATAGTTTAGGGGGAGT	ACCCTTCACAATACCAAAATCTC	CAAACTAACCCCAAATAAAATAACC	55
TUBB6	GGAATAGGGTTTAGTTAAAGTGATTATAGG	Biotin-ACAATCATTCCTTTCCACCACA	GGGTTGGATTAGAAAATGAATATT	59
ZNF154	AGGTGTGTTTATAGGATTAGAGATAGTAG	Biotin-AAACAACCATCCCTATCCCAAACCT	AGTAGAGAGTAGAGTTGGGGTTA	57
