# Supplementary material for: Methylation Profiles Reveal Distinct Subgroup of Hepatocellular Carcinoma Patients with Poor Prognosis
Source: PLoS One. 2014 Aug 5;9(8):e104158. doi: 10.1371/journal.pone.0104158 (PMC4122406; doi:10.1371/journal.pone.0104158)
Supplement: Table S2 — Primers used for quantitative real-time PCR. (PDF) [file pone.0104158.s007.pdf]

Table S2. Primers used for quantitative real time PCR.

**Primer Name**	**Primer Sequence**	**Annealing Temperature (^{o}C)**
B_ACTIN_RT_F	AAAGACCTGTACGCCAACAC	59
B_ACTIN_RT_R	GTCATACTCCTGCTTGCTGAT	
CYB5R2_RT_F	GAACATGAAAATCGGGGAGA	59
CYB5R2_RT_R	TGTCTGGTTGGCAAAGATGA	
GSTP1_RT_F	ATCAGGGCCAGAGCTGGA	59
GSTP1_RT_R	ATAGGCAGGAGGCTTTGAGTGA	
SH3YL1_RT_F	GCAGGCGTGTCTTTAGAAGG	59
SH3YL1_RT_R	CTCCCTTGCTGCTTTTCTTG	
SPINT2_RT_F	AACAGCAATAATTACCTGACC	59
SPINT2_RT_R	AAGGATGCACGGCAAGGC	
