# Supplementary material for: Methylation Profiles Reveal Distinct Subgroup of Hepatocellular Carcinoma Patients with Poor Prognosis
Source: PLoS One. 2014 Aug 5;9(8):e104158. doi: 10.1371/journal.pone.0104158 (PMC4122406; doi:10.1371/journal.pone.0104158)
Supplement: Table S3 — 170 differentially methylated CpG loci that were selected in Consensus Hierarchical Clustering with feature selection. 20 out of 170 genes have differential expression between tumor and adjacent non-tumorous tissues. (PDF) [file pone.0104158.s008.pdf]

Table S3. 170 differentially methylated CpG loci that were selected in Consensus Hierarchical Clustering with feature selection. 20 out of 170 genes have differential expression between tumor and adjacent non-tumorous samples.

No.	Probeset ID	SYMBOL	Mean Difference (Δβ)	FDR adjusted P-value	Expression Fold Change (T/NT)	FDR adjusted P-value
1	cg21376883	ACTN2	0.10	1.28E-03	1.26	2.31E-03
2	cg27050763	AHCTF1	-0.21	4.27E-08	1.20	2.20E-04
3	cg18809289	ALOX5	0.20	4.23E-09	-1.38	3.08E-08
4	cg08690031	CDCA7	0.18	4.30E-06	1.46	3.49E-06
5	cg05755779	COLEC10	-0.16	1.06E-06	-1.85	2.41E-17
6	cg09009111	EMILIN2	0.29	6.94E-11	1.25	1.92E-02
7	cg18661868	FES	0.20	3.88E-09	-1.68	2.21E-09
8	cg17100322	FYN	0.23	6.52E-07	-1.31	1.45E-06
9	cg22129364	GPX7	0.22	2.71E-08	1.40	6.13E-07
10	cg26808784	H19	-0.27	2.49E-13	-2.75	5.24E-06
11	cg06638433	IGF2BP1	0.22	1.06E-08	1.31	4.17E-05
12	cg07862358	MYO10	0.30	6.02E-12	-1.30	9.20E-11
13	cg23681213	PNMA1	0.21	4.71E-08	1.28	5.81E-05
14	cg20276750	PPM1M	0.15	3.87E-04	1.20	1.26E-03
15	cg03262773	RAD54L	0.13	1.72E-03	1.58	1.22E-13
16	cg02589695	RASSF5	0.15	2.20E-04	-1.22	1.52E-05
17	cg22511633	TBL2	0.20	5.80E-09	1.42	1.52E-13
18	cg02545192	TERT	0.27	4.26E-11	1.39	1.09E-05
19	cg20267005	TYROBP	-0.12	3.24E-04	-1.31	8.93E-03
20	cg24680602	ZNF232	0.21	7.31E-08	1.31	3.02E-10
21	cg20359349	APR2	-0.13	3.64E-05		
22	cg07241568	ABO	0.20	5.82E-09		
23	cg09079275	ADAM19	0.14	5.16E-04		
24	cg04988423	ALX4	0.19	4.40E-06		
25	cg11260848	ALX4	0.12	6.80E-04		
26	cg07473175	AMIGO2	0.32	1.24E-14		
27	cg13640200	AMIGO2	0.32	4.04E-13		
28	cg09966445	AMPH	-0.28	3.80E-12		
29	cg16192029	ANKRD7	-0.45	4.97E-23		
30	cg14785449	ASNS	0.24	9.30E-10		
31	cg09911342	ASZ1	-0.25	2.36E-13		
32	cg26349773	ATP6V0A4	-0.17	1.41E-06		
33	cg18236477	ATP8A2	0.28	1.70E-10		
34	cg11507178	BCL9L	0.15	8.46E-05		
35	cg16778903	BRUNOL6	0.46	1.09E-20		
36	cg15516226	BTNL9	-0.36	1.41E-18		
37	cg12281657	C14orf50	0.40	4.82E-20		
38	cg27394486	C15orf2	-0.37	3.49E-16		
39	cg17978274	C15orf32	-0.34	4.62E-19		
40	cg12796229	C18orf43	0.29	6.32E-11		
41	cg04405541	C20orf114	-0.18	2.55E-07		
42	cg01671881	C20orf71	-0.15	3.08E-07		
43	cg07611334	C20orf98	0.30	3.52E-10		
44	cg08713365	C20orf98	0.19	1.51E-07		
45	cg07080358	C2orf32	0.20	2.67E-08		
46	cg04532952	CA4	0.21	4.26E-09		
47	cg21003606	CALN1	-0.33	1.96E-18		
48	cg24517042	CAV3	-0.27	6.36E-15		
49	cg08124722	CCL7	-0.31	8.51E-22		
50	cg04590978	CCNJ	0.40	4.29E-19		
51	cg15743985	CD22	-0.18	9.91E-08		
52	cg21640749	CD300LF	-0.35	4.99E-19		
53	cg07973967	CD79B	-0.11	4.79E-03		
54	cg12038710	CDH17	-0.33	7.31E-17		
55	cg08424423	CDSN	-0.34	1.41E-18		
56	cg08080029	CHD5	0.18	9.06E-06		
57	cg21148892	CLEC4F	-0.28	2.88E-15		
58	cg22241124	CNGA3	-0.23	1.60E-13		
59	cg00290506	CNIH3	0.30	2.43E-11		
60	cg19596755	CPT1A	0.18	7.67E-06		
61	cg21604803	CPT1C	0.25	1.99E-12		
62	cg03451296	CRIP2	0.17	1.78E-04		
63	cg10938286	CST2	-0.37	1.53E-22		
64	cg24875415	CST4	-0.32	1.04E-17		
65	cg07824742	DBH	-0.19	1.76E-10		
66	cg22605063	DBN1	0.24	7.69E-09		
67	cg15481539	DEFA5	-0.25	5.57E-15		
68	cg24890043	DKFZP566N034	0.22	1.33E-06		

69	cg16128363	DLGAP1	-0.25	2.20E-12
70	cg23753610	DNAHL1	-0.36	2.92E-21
71	cg18242139	ELAVL4	-0.25	6.46E-14
72	cg00027083	EPB41L3	0.14	1.58E-03
73	cg04289385	ETV7	0.19	3.66E-06
74	cg24176563	EYA4	0.22	5.08E-08
75	cg26656135	EYA4	0.20	3.44E-07
76	cg13338132	FAM71C	-0.22	5.94E-09
77	cg16516400	FAM89A	0.23	1.97E-09
78	cg07846167	FBLIM1	0.12	2.83E-03
79	cg27062617	FHOD1	0.19	2.43E-06
80	cg15648315	FLJ26443	-0.14	6.60E-06
81	cg03044435	FLJ35816	-0.31	2.52E-18
82	cg22777952	FOXB1	0.21	2.70E-08
83	cg03663715	FOXD1	0.25	3.98E-10
84	cg00661485	FOXI1	-0.36	6.53E-20
85	cg17503456	FOXL2	0.32	1.00E-12
86	cg18047970	GADL1	-0.20	2.59E-09
87	cg04534765	GALR1	0.13	1.78E-03
88	cg20822628	GATA5	-0.19	2.22E-10
89	cg06954481	GBX2	0.29	2.01E-09
90	cg26970800	GIF	-0.28	6.25E-13
91	cg18538812	GIF	-0.20	7.78E-10
92	cg04037952	GPR148	-0.23	4.88E-12
93	cg04597449	GPR150	0.23	4.72E-12
94	cg26252167	GPR6	0.21	1.66E-07
95	cg24694549	GRIP1	-0.27	2.85E-15
96	cg14859460	GRM6	0.13	1.88E-03
97	cg08766149	GZMB	-0.38	6.62E-22
98	cg17718302	HIST1H3J	0.48	1.69E-24
99	cg20899053	HLXB9	0.26	4.60E-09
100	cg24417499	HPCA	0.19	2.99E-08
101	cg26589285	IL29	-0.24	8.32E-13
102	cg03963198	IRX4	0.31	8.51E-13
103	cg00243313	IRX4	0.19	4.75E-07
104	cg14614211	IRXL1	0.21	1.61E-07
105	cg11845202	K5B	-0.27	6.96E-13
106	cg17255302	K6HF	-0.28	8.98E-15
107	cg12144803	KALRN	-0.22	1.27E-11
108	cg24924779	KCNG1	0.20	2.22E-06
109	cg26750319	KCNQ1	-0.28	1.54E-14
110	cg04254916	KRT5	-0.29	2.87E-17
111	cg18370227	KRTHA3A	-0.29	4.23E-16
112	cg04618528	KRTHA6	-0.30	4.13E-18
113	cg24461814	KRTHB4	-0.30	3.66E-16
114	cg07846220	LAMA1	0.23	9.62E-06
115	cg02718531	LOC114984	0.16	3.06E-05
116	cg16456919	LRRN5	-0.11	1.80E-03
117	cg00233307	MAP4K1	0.22	4.64E-09
118	cg18971054	MGAM	-0.26	4.62E-13
119	cg23502772	MGC42105	0.24	1.57E-09
120	cg23771603	MYO3A	0.19	6.71E-06
121	cg08441170	MYO3A	0.10	5.31E-03
122	cg04882759	MYOCD	-0.27	1.84E-15
123	cg07271264	MYOD1	0.15	2.17E-04
124	cg18059223	NALP2	-0.17	3.72E-09
125	cg23290344	NEF3	0.35	2.52E-13
126	cg18267374	NEF3	0.30	6.79E-12
127	cg14958635	NEUROG1	0.19	3.60E-06
128	cg22571530	NFASC	0.19	6.85E-07
129	cg05973262	NOTCH4	-0.22	9.39E-11
130	cg26845838	OACT2	0.30	1.79E-11
131	cg11360149	OR2V2	-0.28	1.93E-14
132	cg11487705	OXCT1	0.30	2.35E-11
133	cg18902090	PCDHAC1	0.19	9.97E-07
134	cg05436658	PRKCB1	0.33	3.53E-13
135	cg19769182	PRRT2	0.16	1.68E-06
136	cg24989962	PTGDR	0.29	2.03E-12
137	cg14037665	PXDN	0.18	4.17E-06
138	cg12164282	PXDN	0.14	1.73E-03
139	cg18410627	RALGPS1	0.30	8.08E-15
140	cg19332710	RIMS4	0.31	2.56E-09
141	cg15772361	SERPINB3	-0.28	3.31E-17

142	cg22946150	SH3GL3	0.17	1.17E-04
143	cg18986165	SIGLEC12	-0.35	2.05E-19
144	cg25437385	SLC35F3	0.26	1.25E-08
145	cg22445920	SLC36A3	-0.22	1.23E-11
146	cg02919422	SOX17	0.20	7.16E-07
147	cg10983208	SPOCK2	0.23	6.58E-09
148	cg20339230	ST8SIA2	0.24	5.02E-09
149	cg15873301	SYN2	0.23	1.79E-09
150	cg12277666	TDRD5	0.23	5.74E-09
151	cg02915837	TEAD4	0.14	2.75E-04
152	cg20488657	TFF3	-0.22	1.19E-09
153	cg01186777	TNFSF9	0.26	1.00E-09
154	cg19642007	TNNT3	-0.32	7.05E-19
155	cg15120497	TRH	-0.19	1.09E-07
156	cg07533148	TRIM58	0.34	1.29E-11
157	cg13628514	TRPV4	0.16	3.10E-05
158	cg06437862	TUBA2	-0.31	7.36E-14
159	cg23517605	TUBB2B	0.15	9.12E-06
160	cg20052718	TWIST1	0.22	1.28E-10
161	cg26312150	TWIST1	0.19	5.01E-09
162	cg05241571	UNQ467	-0.27	1.70E-11
163	cg00333226	UNQ739	0.15	2.02E-06
164	cg17162024	UNQ9433	0.22	2.76E-08
165	cg18886444	USP4	0.13	3.80E-04
166	cg24662718	VAV3	0.31	2.85E-13
167	cg18349835	VIPR2	0.32	8.29E-12
168	cg14667273	VWA1	0.13	1.48E-03
169	cg22021786	WFDC8	-0.27	1.33E-12
170	cg22637941	WISP1	-0.13	1.40E-03
