# Supplementary material for: Methylation Profiles Reveal Distinct Subgroup of Hepatocellular Carcinoma Patients with Poor Prognosis
Source: PLoS One. 2014 Aug 5;9(8):e104158. doi: 10.1371/journal.pone.0104158 (PMC4122406; doi:10.1371/journal.pone.0104158)
Supplement: Table S4 — Correlation between tumor subgroups and clinicopathological parameters in HCC samples. Fisher’s exact test was used to test the correlation between tumor subgroups and clinicopathological parameters. (PDF) [file pone.0104158.s009.pdf]

Table S4. Correlation between tumor subgroups and clinicopathological parameters in HCC samples. Fisher's exact test was used to test the correlation between tumor subgroups and clinicopathological parameters.

Parameter	Available Data	Group-1	Group-2	Group-3	p-value[a]
Age at diagnosis (Median=65.3)					0.55
≥65 years old	59	9	7	15	
<65 years old		12	4	12	
Gender					0.87
Male	59	19	10	23	
Female		2	1	4	
HBV status					0.73
postive	59	12	6	18	
negative		9	5	9	
Tumor size					1.00
≥5 cm	59	12	6	15	
<5 cm		9	5	12	
Differentiation (Edmonson)					0.32
I,II	59	11	7	10	
III,IV		10	4	17	
TNM staging					0.25
1	58	13	3	16	
2,3		8	7	11	
Cirrhosis					0.59
Absent	58	12	6	19	
Present		8	5	8	
Tumor Multifocality					1.00
Absent	55	15	8	22	
Present		3	2	5	
Tumor Encapsulation					0.27
Absent	53	10	5	20	
Present		8	4	6	
AFP level					0.85
≥100ng/ml	51	6	2	7	
<100ng/ml		13	8	15	

[a]Fisher's Exact Test
