# Supplementary material for: Methylation Profiles Reveal Distinct Subgroup of Hepatocellular Carcinoma Patients with Poor Prognosis
Source: PLoS One. 2014 Aug 5;9(8):e104158. doi: 10.1371/journal.pone.0104158 (PMC4122406; doi:10.1371/journal.pone.0104158)
Supplement: Table S5 — 4416 differentially methylated CpG loci between tumors and adjacent non-tumorous tissues. (PDF) [file pone.0104158.s010.pdf]

Table S5. 4416 differentially methylated CpG loci between tumors and adjacent non-tumorous tissues.

No.	Probeset ID	SYMBOL	Mean Difference (Δβ)	FDR adjusted P-value
1	cg22568540	A1BG	-0.16	4.83E-07
2	cg27653134	A2ML1	-0.17	4.51E-12
3	cg03490200	A2ML1	-0.24	2.34E-13
4	cg09382492	AANAT	-0.14	7.58E-08
5	cg05292376	AATK	-0.17	1.56E-10
6	cg00949442	ABCA3	0.52	8.68E-36
7	cg02331561	ABCA3	0.10	1.79E-02
8	cg21643191	ABCB5	-0.16	2.17E-08
9	cg11429658	ABCC13	-0.12	9.67E-06
10	cg25781162	ABCG5	-0.12	4.61E-05
11	cg08453096	ABCG5	-0.13	4.06E-06
12	cg13984181	ABHD7	0.37	2.48E-16
13	cg15156367	ABHD7	-0.12	1.26E-07
14	cg05488632	ABHD9	0.45	3.45E-33
15	cg25839227	ABI3	-0.13	1.18E-05
16	cg21400896	ABI3	-0.19	1.25E-10
17	cg05026186	ABLIM3	-0.12	1.75E-17
18	cg07241568	ABO	0.20	5.82E-09
19	cg11879188	ABO	-0.21	7.61E-11
20	cg25374854	ABR	0.15	2.48E-06
21	cg15603885	ABRA	-0.18	1.46E-08
22	cg07005767	ABRA	-0.28	6.14E-18
23	cg03805442	ACCN1	-0.12	6.27E-10
24	cg02812142	ACMSD	-0.16	1.36E-15
25	cg10266490	ACOT11	0.20	3.00E-17
26	cg26780333	ACOT4	0.14	4.39E-08
27	cg08101264	ACOT8	0.13	4.53E-04
28	cg17947599	ACOXL	-0.11	9.98E-07
29	cg07967308	ACP5	-0.10	4.52E-06
30	cg00400028	ACPL2	0.18	1.26E-07
31	cg15782391	ACPT	-0.21	2.62E-14
32	cg27257408	ACSBG1	-0.12	6.22E-08
33	cg27087809	ACSBG1	-0.13	5.60E-11
34	cg08256781	ACSBG2	-0.17	5.57E-12
35	cg13849691	ACSL5	-0.11	5.58E-04
36	cg15988792	ACSS1	0.23	7.10E-10
37	cg13547644	ACTA1	0.21	4.10E-07
38	cg20025656	ACTA1	0.12	3.17E-04
39	cg19517525	ACTG1	0.35	2.93E-11
40	cg23173910	ACTG2	-0.16	3.85E-11
41	cg08572611	ACTL6B	0.30	1.23E-10
42	cg14088161	ACTL6B	-0.10	1.25E-06
43	cg19863405	ACTL7B	-0.18	1.80E-11
44	cg19685066	ACTL8	-0.30	9.37E-16
45	cg21376883	ACTN2	0.10	1.28E-03

46	cg16159313	ACY1	-0.17	2.47E-06
47	cg11096993	ACY3	-0.12	1.42E-07
48	cg11668923	ADAM12	0.15	3.13E-04
49	cg23566335	ADAM18	-0.22	2.41E-14
50	cg09079275	ADAM19	0.14	5.16E-04
51	cg09314766	ADAM2	-0.19	8.75E-14
52	cg03716937	ADAM2	-0.25	3.05E-16
53	cg05997860	ADAM21	-0.12	6.16E-08
54	cg02998425	ADAM21	-0.19	1.46E-15
55	cg16778809	ADAM23	0.18	1.61E-06
56	cg16411152	ADAM29	-0.23	5.59E-16
57	cg14561282	ADAM29	-0.26	8.69E-19
58	cg22333888	ADAM33	0.12	1.12E-06
59	cg16786703	ADAM8	0.37	2.50E-17
60	cg19730092	ADAMTS14	-0.11	8.99E-05
61	cg02564733	ADAMTS15	0.10	3.03E-04
62	cg23727043	ADAMTS7	0.19	2.05E-10
63	cg01260219	ADAMTS8	-0.16	3.76E-09
64	cg16714091	ADAMTSL1	0.10	7.65E-04
65	cg17644208	ADCY3	0.11	2.27E-06
66	cg12265829	ADCY4	0.21	1.03E-07
67	cg16761581	ADCY4	0.18	3.70E-08
68	cg13878010	ADCY5	0.37	4.51E-16
69	cg13384396	ADCY5	0.33	7.78E-13
70	cg07376535	ADCYAP1	0.15	5.48E-07
71	cg13626881	ADORA1	-0.12	1.94E-11
72	cg21949305	ADORA2A	0.22	1.16E-13
73	cg02947253	ADORA3	-0.16	2.45E-08
74	cg17963840	ADRA1A	0.14	6.57E-05
75	cg11934695	ADRA1D	0.17	1.99E-06
76	cg21542793	ADRA2B	0.37	2.47E-20
77	cg10235817	ADRA2C	0.37	4.07E-25
78	cg14826456	ADRB1	0.34	2.94E-19
79	cg17619823	ADRB3	0.25	1.30E-09
80	cg02126753	AEBP1	0.15	4.32E-07
81	cg15957394	AFAP	0.26	2.50E-08
82	cg19564367	AFAP	0.22	6.67E-07
83	cg11376198	AFAR3	-0.20	1.30E-11
84	cg21201572	AGR2	-0.16	7.72E-08
85	cg19125606	AGT	-0.11	4.56E-08
86	cg22647018	AGT	-0.14	2.53E-08
87	cg26637069	AGTRL1	-0.11	3.65E-04
88	cg16967583	AGXT	-0.10	1.85E-10
89	cg27050763	AHCTF1	-0.21	4.27E-08
90	cg07361385	AHSG	-0.17	6.81E-09
91	cg11003133	AIM2	-0.12	1.89E-05
92	cg26770882	AIPL1	-0.16	9.61E-09
93	cg18876487	AIRE	-0.19	1.65E-12
94	cg20959866	AJAP1	0.15	7.99E-05
95	cg17525406	AJAP1	0.13	7.57E-04

96	cg11714502	AK1	-0.11	2.35E-05
97	cg11630242	AKAP10	0.23	1.08E-10
98	cg01555431	AKAP12	0.17	1.83E-05
99	cg15083233	AKAP2	0.21	4.35E-11
100	cg19176447	AKAP2	0.17	5.40E-11
101	cg07892051	AKAP3	-0.11	2.27E-08
102	cg04254119	AKAP3	-0.13	1.14E-08
103	cg13801416	AKR1B1	0.40	3.56E-15
104	cg18416881	AKR1B1	0.29	6.33E-13
105	cg11693019	AKR1B10	-0.17	2.08E-12
106	cg11314684	AKT3	-0.14	2.56E-10
107	cg22637834	AKT3	-0.19	1.40E-14
108	cg03365437	ALDH1A2	0.26	1.61E-10
109	cg00930873	ALDH1A2	0.25	6.79E-10
110	cg19177941	ALDH1A3	0.26	2.36E-12
111	cg21631409	ALDH3B2	-0.17	2.88E-24
112	cg06367117	ALDOC	0.16	3.36E-04
113	cg18277754	ALK	0.10	1.86E-04
114	cg03760483	ALOX12	0.24	2.18E-14
115	cg03742272	ALOX12B	-0.25	1.47E-16
116	cg00501366	ALOX12B	-0.26	2.02E-22
117	cg15843823	ALOX15	0.17	2.53E-06
118	cg18809289	ALOX5	0.20	4.23E-09
119	cg20645065	ALPL	0.21	6.46E-07
120	cg13605579	ALPP	-0.15	1.19E-09
121	cg11052143	ALS2CR11	0.28	3.34E-28
122	cg15780361	ALS2CR11	0.13	3.42E-11
123	cg14144305	ALX4	0.21	2.63E-09
124	cg26365854	ALX4	0.19	7.71E-08
125	cg04988423	ALX4	0.19	4.40E-06
126	cg15817236	ALX4	0.13	1.40E-05
127	cg11260848	ALX4	0.12	6.80E-04
128	cg04549333	ALX4	0.11	7.44E-05
129	cg06630567	AMBP	-0.11	5.00E-05
130	cg22296149	AMH	-0.12	8.34E-07
131	cg07473175	AMIGO2	0.32	1.24E-14
132	cg13640200	AMIGO2	0.32	4.04E-13
133	cg08035082	AMPD3	-0.13	1.37E-07
134	cg09966445	AMPH	-0.28	3.80E-12
135	cg26507477	AMY1A	-0.11	3.69E-17
136	cg21919219	AMY1A	-0.20	7.10E-15
137	cg22215192	AMY2A	-0.15	9.44E-10
138	cg26540515	ANGPT4	-0.23	4.95E-14
139	cg19531130	ANGPTL5	0.17	3.37E-11
140	cg04508649	ANGPTL7	0.24	1.25E-21
141	cg26984624	ANK1	-0.16	1.40E-11
142	cg02735486	ANK2	-0.12	1.26E-04
143	cg08321346	ANKMY1	0.35	1.18E-25
144	cg27504117	ANKMY1	0.15	1.05E-13
145	cg26381783	ANKRD15	0.33	6.79E-11

146	cg00344709	ANKRD21	-0.17	3.01E-10
147	cg18493238	ANKRD30A	-0.17	9.31E-11
148	cg19219503	ANKRD30A	-0.19	6.36E-09
149	cg19948393	ANKRD33	0.14	7.34E-08
150	cg19974223	ANKRD33	0.12	1.13E-08
151	cg00625425	ANKRD38	-0.12	1.85E-04
152	cg21435336	ANKRD41	0.11	1.94E-06
153	cg04446579	ANKRD45	0.23	1.31E-14
154	cg15883716	ANKRD45	0.22	6.08E-14
155	cg11075556	ANKRD7	-0.21	1.54E-16
156	cg16192029	ANKRD7	-0.45	4.97E-23
157	cg13003163	ANP32D	-0.12	4.02E-11
158	cg15711744	ANP32D	-0.25	6.27E-15
159	cg13042288	ANPEP	-0.11	1.32E-05
160	cg20437604	ANXA9	-0.18	1.84E-13
161	cg14451276	AOAH	0.11	1.09E-05
162	cg09472203	AP3B2	0.25	2.33E-08
163	cg13140464	AP3B2	-0.15	9.36E-11
164	cg07490776	AP3M2	0.13	3.53E-04
165	cg12044210	APBA2	-0.15	1.83E-07
166	cg21917349	APBA2	-0.15	2.87E-07
167	cg16970232	APC	0.42	2.56E-20
168	cg21634602	APC	0.41	4.04E-17
169	cg20311501	APC	0.35	1.61E-18
170	cg24332422	APC	0.32	2.19E-16
171	cg15020645	APC	0.26	4.63E-11
172	cg01240931	APC	0.12	6.22E-08
173	cg19264571	APCDD1	0.18	4.22E-13
174	cg16222568	APEG1	-0.22	5.80E-13
175	cg10062065	APEG1	-0.27	4.35E-17
176	cg17207590	APH1B	0.22	4.13E-12
177	cg19995014	APLP1	0.11	2.48E-04
178	cg01053621	APOA2	-0.18	6.13E-14
179	cg02157083	APOA5	0.14	2.65E-07
180	cg24309555	APOB	-0.11	1.98E-09
181	cg16879115	APOBEC1	-0.11	4.15E-11
182	cg22375610	APOBEC2	-0.15	1.58E-10
183	cg22954818	APOBEC3A	-0.18	2.03E-15
184	cg07186138	APOBEC3C	0.17	1.52E-08
185	cg26022401	APOBEC3G	0.14	1.44E-06
186	cg27436184	APOC2	-0.11	1.90E-05
187	cg04048249	APOC3	-0.12	1.58E-09
188	cg20359349	APR2	-0.13	3.64E-05
189	cg20176648	AQP1	-0.12	9.73E-06
190	cg20713492	AQP10	-0.12	4.00E-06
191	cg01447817	AQP12A	-0.14	1.77E-06
192	cg06356454	AQP12A	-0.14	4.47E-07
193	cg07047653	AQP2	-0.10	1.74E-08
194	cg12650635	AQP2	-0.18	1.54E-09
195	cg08594695	AQP4	-0.12	1.65E-08

196	cg23855989	AQP5	0.13	2.58E-07
197	cg24352499	AQP6	-0.12	3.10E-17
198	cg13246269	AQP7	-0.14	5.89E-15
199	cg07327347	AQP8	-0.12	2.69E-05
200	cg09954385	ARHGAP8	0.26	9.13E-11
201	cg10925082	ARHGDIB	-0.14	5.50E-09
202	cg03050522	ARHGEF1	0.15	2.71E-05
203	cg14681767	ARHGEF10	0.14	1.39E-04
204	cg24919884	ARHGEF16	-0.10	2.58E-04
205	cg27377450	ARHGEF18	-0.12	5.41E-13
206	cg02981853	ARHGEF18	-0.20	1.31E-11
207	cg18669381	ARHGEF19	-0.11	2.18E-10
208	cg11469778	ARHGEF4	-0.12	5.08E-04
209	cg24697329	ARHGEF4	-0.15	1.51E-06
210	cg00557354	ARHGEF7	0.13	1.57E-03
211	cg16427670	ARHGEF7	0.12	2.05E-04
212	cg18084554	ARID3A	0.11	3.56E-05
213	cg11673092	ARMC3	0.12	2.51E-04
214	cg02512226	ARMC4	0.13	1.46E-07
215	cg17942553	ARNT	-0.11	2.77E-07
216	cg20775959	ARNT2	0.14	7.01E-05
217	cg17788682	ARNT2	0.11	1.39E-03
218	cg14365123	ARPM2	-0.26	8.05E-14
219	cg25384157	ARRDC1	0.15	3.93E-07
220	cg08558340	ARS2	0.22	2.21E-11
221	cg21407055	ART1	-0.19	4.03E-11
222	cg25221254	ASAH3	-0.25	1.18E-14
223	cg26159905	ASB10	-0.13	1.18E-11
224	cg21038703	ASB16	-0.12	8.43E-07
225	cg19949550	ASB2	-0.15	1.35E-06
226	cg26847490	ASB4	-0.12	4.71E-08
227	cg11554605	ASB4	-0.15	1.47E-06
228	cg12182525	ASB5	-0.16	3.05E-15
229	cg06263495	ASCL2	0.38	4.08E-19
230	cg14785449	ASNS	0.24	9.30E-10
231	cg01245656	ASNS	0.22	8.24E-09
232	cg16540704	ASZ1	-0.22	2.25E-14
233	cg09911342	ASZ1	-0.25	2.36E-13
234	cg27383362	ATAD3C	-0.13	3.98E-08
235	cg24027342	ATM	-0.11	2.16E-24
236	cg10734665	ATP10A	-0.11	8.51E-04
237	cg08828036	ATP10A	-0.12	4.78E-06
238	cg14001035	ATP10A	-0.17	1.15E-10
239	cg17260954	ATP10A	-0.18	4.85E-13
240	cg19930802	ATP10A	-0.24	1.36E-19
241	cg26062856	ATP10A	-0.25	5.39E-16
242	cg20119871	ATP10A	-0.30	7.09E-20
243	cg11015241	ATP10A	-0.34	1.71E-24
244	cg12582965	ATP10A	-0.36	1.25E-18
245	cg13337662	ATP12A	-0.20	7.23E-11

246	cg09580336	ATP1A1	0.25	3.76E-19
247	cg07051960	ATP1A3	-0.12	4.47E-12
248	cg06517798	ATP1A4	-0.25	1.16E-17
249	cg01914242	ATP2A3	0.16	7.98E-06
250	cg06123346	ATP4A	-0.16	4.76E-22
251	cg06811800	ATP4B	-0.22	2.58E-16
252	cg08995424	ATP5G2	0.31	1.89E-16
253	cg03665605	ATP5J2	-0.17	7.43E-14
254	cg02491878	ATP6V0A4	-0.13	3.78E-07
255	cg26349773	ATP6V0A4	-0.17	1.41E-06
256	cg05483509	ATP6V0C	0.29	7.27E-17
257	cg01248426	ATP6V0D2	-0.16	9.59E-11
258	cg00319692	ATP6V0D2	-0.22	2.65E-15
259	cg06946880	ATP6V1B1	-0.14	1.71E-07
260	cg07052880	ATP6V1B1	-0.19	1.04E-10
261	cg27485921	ATP6V1E2	-0.11	2.89E-07
262	cg18236477	ATP8A2	0.28	1.70E-10
263	cg12111714	ATP8A2	0.22	4.38E-12
264	cg04335339	ATRNL1	0.15	1.95E-07
265	cg02028524	ATXN3	-0.24	1.62E-13
266	cg15001381	AXIN1	0.12	2.45E-03
267	cg16176379	AYTL1	0.17	6.69E-06
268	cg19465374	AZGP1	-0.10	3.55E-04
269	cg12019109	AZGP1	-0.16	2.06E-12
270	cg16608652	B3GALT2	0.15	2.25E-07
271	cg12716838	B3GALT3	0.26	1.19E-08
272	cg26709720	B3GALT5	-0.17	4.55E-12
273	cg20557104	B3GALT7	0.17	3.35E-11
274	cg11038843	B3GAT1	0.11	7.93E-04
275	cg27049761	B3GNT4	0.21	1.11E-07
276	cg17701886	B3GNT5	0.16	6.81E-06
277	cg02238826	B3GNT5	0.16	4.33E-06
278	cg11065385	B4GALNT3	0.14	1.56E-06
279	cg05769161	B4GALNT3	0.14	1.09E-06
280	cg16004226	B4GALNT4	0.18	5.39E-06
281	cg02930996	B4GALT6	0.28	2.82E-17
282	cg07027513	B4GALT6	0.28	9.51E-17
283	cg10918202	BAALC	0.24	1.41E-07
284	cg25906419	BACE2	0.15	2.14E-05
285	cg09143663	BACH1	-0.13	2.67E-06
286	cg26287988	BAGE	-0.13	2.86E-12
287	cg07973461	BAGE	-0.23	6.46E-19
288	cg20994561	BAI1	-0.27	1.24E-17
289	cg07679836	BAK1	0.19	4.07E-09
290	cg00332153	BANK1	0.19	1.15E-06
291	cg20073553	BAPX1	0.38	9.83E-23
292	cg12622986	BARHL1	0.13	3.26E-06
293	cg17241310	BARHL2	0.28	8.12E-10
294	cg23496260	BASP1	0.14	2.27E-06
295	cg10006582	BBS5	0.14	1.20E-06

296	cg21475402	BCAN	0.37	3.18E-17
297	cg18060199	BCAR3	0.25	1.63E-08
298	cg18917378	BCAS1	-0.12	1.77E-07
299	cg10764357	BCAT1	0.12	1.03E-04
300	cg12584718	BCDIN3	0.11	3.28E-05
301	cg22166290	BCL11A	0.12	6.83E-05
302	cg21602520	BCL2	0.37	2.07E-15
303	cg17602451	BCL2	0.31	4.59E-11
304	cg11507178	BCL9L	0.15	8.46E-05
305	cg24789424	BDH	-0.14	1.13E-07
306	cg02214188	BDH2	-0.11	3.45E-09
307	cg10528989	BDKRB1	-0.11	6.74E-06
308	cg20383064	BFSP2	-0.14	3.42E-09
309	cg25249068	BFSP2	-0.17	5.94E-09
310	cg26453588	BIK	0.23	5.28E-17
311	cg17237881	BIRC7	-0.17	2.40E-13
312	cg09377486	BIRC8	-0.10	6.83E-07
313	cg24454579	BIRC8	-0.18	3.30E-11
314	cg03860768	BLK	-0.26	2.40E-20
315	cg02226939	BLMH	0.14	3.46E-06
316	cg17571291	BLVRA	0.11	1.53E-04
317	cg23587532	BM88	-0.16	8.95E-09
318	cg14310034	BMP4	0.44	6.79E-23
319	cg03447931	BMP6	0.23	1.49E-07
320	cg07479491	BMP6	0.11	6.69E-06
321	cg26516759	BMP7	0.12	3.36E-04
322	cg03664992	BMP8A	0.29	1.39E-12
323	cg07082331	BMP8B	0.18	1.12E-06
324	cg17561435	BMPER	0.17	3.40E-06
325	cg18952647	BNC1	0.28	1.91E-11
326	cg19988449	BNC1	0.23	1.32E-09
327	cg10398682	BNC1	0.20	3.47E-09
328	cg21762589	BNIP3	0.12	3.14E-11
329	cg18742893	BOC	0.12	8.69E-05
330	cg17560332	BOLL	0.36	1.21E-22
331	cg10124201	BOLL	0.10	2.58E-04
332	cg14115346	BPHL	-0.12	3.43E-13
333	cg08254089	BPI	-0.15	4.52E-16
334	cg15494458	BPI	-0.16	8.45E-14
335	cg10968815	BPIL1	-0.13	1.54E-07
336	cg13696012	BPIL1	-0.13	4.53E-06
337	cg27195224	BPIL2	-0.18	7.24E-16
338	cg18223379	BPIL3	-0.19	1.61E-12
339	cg02309431	BPIL3	-0.22	9.29E-13
340	cg14732540	BRDT	-0.10	2.11E-03
341	cg00307685	BRDT	-0.19	4.55E-12
342	cg16313343	BRF1	0.22	1.72E-07
343	cg01468621	BRSK2	0.17	2.94E-05
344	cg14021073	BRSK2	0.15	3.08E-07
345	cg21801378	BRUNOL6	0.48	6.85E-23

346	cg16778903	BRUNOL6	0.46	1.09E-20
347	cg01532103	BSG	0.11	9.12E-03
348	cg14615807	BSND	-0.13	1.60E-12
349	cg19971655	BSND	-0.14	2.02E-12
350	cg14603345	BTBD3	-0.20	4.21E-11
351	cg21291985	BTBD4	-0.14	8.63E-07
352	cg23034818	BTBD6	0.19	1.02E-05
353	cg09450238	BTBD6	0.12	6.71E-07
354	cg19302283	BTC	-0.10	1.97E-12
355	cg18204685	BTD	-0.14	9.76E-08
356	cg14380517	BTG3	0.18	1.19E-06
357	cg23211240	BTG4	0.17	4.92E-05
358	cg01192900	BTG4	0.14	1.18E-05
359	cg22879515	BTG4	0.13	1.40E-06
360	cg25391023	BTNL2	-0.11	3.31E-10
361	cg24024214	BTNL8	-0.16	9.44E-12
362	cg15516226	BTNL9	-0.36	1.41E-18
363	cg20624391	BVES	0.13	3.23E-07
364	cg22182666	BZRAP1	-0.14	4.75E-08
365	cg16063112	C10orf107	0.13	1.99E-05
366	cg15227982	C10orf26	0.14	1.11E-07
367	cg09405083	C10orf26	0.10	2.57E-05
368	cg10548978	C10orf27	-0.15	2.25E-09
369	cg01560871	C10orf27	-0.28	5.60E-21
370	cg08397758	C10orf33	0.21	1.30E-08
371	cg03852570	C10orf33	0.20	1.19E-07
372	cg05942970	C10orf39	-0.20	5.38E-10
373	cg06985415	C10orf39	-0.23	3.72E-11
374	cg13985639	C10orf65	0.10	2.04E-04
375	cg08427977	C10orf72	-0.15	1.31E-09
376	cg22045288	C10orf91	-0.14	2.76E-14
377	cg04126866	C10orf99	-0.11	1.49E-09
378	cg01558777	C10orf99	-0.23	7.64E-20
379	cg03805684	C11orf10	-0.17	4.98E-18
380	cg01643624	C11orf16	-0.18	1.03E-12
381	cg07747336	C11orf38	-0.14	8.31E-17
382	cg23743472	C11orf38	-0.30	4.31E-22
383	cg25943276	C11orf39	-0.26	1.56E-16
384	cg02477931	C11orf39	-0.26	3.56E-18
385	cg16270990	C11orf44	-0.13	2.83E-06
386	cg13125510	C11orf44	-0.20	7.71E-17
387	cg19310430	C11orf45	0.13	3.31E-05
388	cg01335367	C12orf34	0.13	3.83E-09
389	cg02351381	C12orf34	0.13	5.54E-09
390	cg14738823	C12orf46	-0.17	2.88E-15
391	cg13435792	C12orf46	-0.22	3.41E-12
392	cg24276491	C14orf11	-0.18	7.56E-11
393	cg22247240	C14orf115	-0.17	2.23E-07
394	cg20022541	C14orf152	-0.31	4.29E-17
395	cg14912575	C14orf162	0.15	3.61E-09

396	cg12281657	C14orf50	0.40	4.82E-20
397	cg04536575	C14orf50	0.11	5.09E-05
398	cg00239071	C14orf68	0.21	2.91E-19
399	cg27394486	C15orf2	-0.37	3.49E-16
400	cg25455753	C15orf32	-0.28	2.08E-24
401	cg17978274	C15orf32	-0.34	4.62E-19
402	cg11911951	C16orf28	-0.13	5.48E-07
403	cg23617760	C16orf44	-0.25	2.15E-11
404	cg23093496	C16orf54	0.12	7.76E-07
405	cg18017908	C17orf76	0.13	2.44E-06
406	cg03686067	C18orf14	-0.10	1.17E-07
407	cg15081561	C18orf14	-0.20	5.71E-13
408	cg00729275	C18orf16	-0.10	1.92E-05
409	cg10054857	C18orf20	-0.10	3.99E-06
410	cg01722450	C18orf20	-0.14	5.67E-10
411	cg24300924	C18orf43	0.41	2.73E-17
412	cg12796229	C18orf43	0.29	6.32E-11
413	cg12334759	C19orf19	-0.23	4.34E-19
414	cg20973210	C19orf35	0.26	8.17E-16
415	cg08047907	C1orf114	0.22	5.40E-09
416	cg21852292	C1orf115	0.21	4.71E-10
417	cg01119135	C1orf116	-0.15	3.64E-09
418	cg15459773	C1orf124	-0.12	4.27E-06
419	cg04636557	C1orf124	-0.18	7.69E-11
420	cg05352668	C1orf127	-0.16	4.42E-10
421	cg18908499	C1orf150	-0.13	1.36E-08
422	cg05767404	C1orf150	-0.19	3.60E-20
423	cg09440340	C1orf161	-0.12	3.55E-08
424	cg01267315	C1orf164	0.13	3.48E-05
425	cg15731815	C1orf188	0.11	3.79E-06
426	cg25182523	C1orf24	-0.12	1.21E-08
427	cg12588301	C1orf42	-0.28	1.97E-19
428	cg11750883	C1orf42	-0.30	6.47E-20
429	cg08460435	C1orf59	0.33	2.70E-23
430	cg08887581	C1orf64	-0.12	9.43E-05
431	cg24525573	C1orf64	-0.15	6.52E-09
432	cg13449778	C1orf76	0.18	3.51E-07
433	cg05421688	C1orf76	0.11	8.12E-06
434	cg18870231	C1orf87	-0.11	4.97E-07
435	cg21926138	C1orf88	0.16	5.28E-07
436	cg11524446	C1orf94	-0.12	3.61E-04
437	cg00108454	C1QA	-0.10	6.48E-12
438	cg25995460	C1QA	-0.14	5.20E-08
439	cg22477971	C1QB	-0.25	6.96E-21
440	cg03941108	C1QB	-0.27	2.08E-19
441	cg11393848	C1QC	-0.11	8.71E-19
442	cg00136477	C1QC	-0.16	1.43E-14
443	cg13818573	C1QL1	0.17	3.61E-05
444	cg21023001	C1QR1	-0.27	1.11E-18
445	cg19138960	C1QR1	-0.29	2.78E-17

446	cg24844534	C1QTNF1	0.13	1.52E-04
447	cg26090652	C1QTNF5	0.33	7.45E-17
448	cg20483374	C1QTNF5	0.20	3.47E-08
449	cg04369341	C20orf100	0.27	3.53E-12
450	cg11319389	C20orf100	0.26	1.14E-10
451	cg04405541	C20orf114	-0.18	2.55E-07
452	cg22789545	C20orf114	-0.21	1.45E-12
453	cg01265637	C20orf141	-0.11	2.11E-09
454	cg19470701	C20orf149	0.15	1.44E-04
455	cg11154879	C20orf151	-0.14	1.62E-08
456	cg16796951	C20orf152	-0.11	2.56E-08
457	cg24691461	C20orf160	-0.10	2.29E-08
458	cg11801374	C20orf160	-0.27	2.53E-19
459	cg26111757	C20orf185	-0.18	1.49E-09
460	cg16483466	C20orf186	-0.22	2.02E-12
461	cg24122922	C20orf39	-0.20	1.82E-12
462	cg06668073	C20orf42	0.12	1.53E-04
463	cg11113534	C20orf70	-0.14	8.71E-07
464	cg01671881	C20orf71	-0.15	3.08E-07
465	cg27132814	C20orf79	-0.11	6.35E-10
466	cg20998885	C20orf79	-0.24	2.01E-16
467	cg15310873	C20orf85	-0.10	1.31E-03
468	cg12813792	C20orf85	-0.10	1.73E-08
469	cg21663722	C20orf91	-0.22	7.11E-20
470	cg07611334	C20orf98	0.30	3.52E-10
471	cg08713365	C20orf98	0.19	1.51E-07
472	cg06940107	C21orf100	-0.14	4.66E-12
473	cg10569414	C21orf121	-0.11	4.98E-04
474	cg23178308	C21orf124	0.18	1.09E-04
475	cg13520715	C21orf129	-0.15	2.36E-07
476	cg27544190	C21orf63	0.13	5.40E-05
477	cg21968796	C21orf70	0.10	8.10E-08
478	cg12003230	C21orf84	0.11	6.42E-10
479	cg23732182	C21orf84	-0.11	1.66E-04
480	cg10756887	C22orf15	0.17	1.57E-12
481	cg14127659	C22orf23	-0.14	2.28E-08
482	cg13438834	C22orf8	0.16	2.84E-05
483	cg06575572	C22orf8	0.14	1.53E-04
484	cg14419187	C2orf21	0.13	6.67E-05
485	cg11084611	C2orf27	-0.16	2.01E-15
486	cg07080358	C2orf32	0.20	2.67E-08
487	cg03338189	C2orf4	0.18	2.40E-08
488	cg22751696	C3orf14	0.10	2.31E-05
489	cg08145590	C3orf15	0.10	9.08E-03
490	cg02765820	C3orf22	-0.16	2.85E-13
491	cg07084746	C3orf27	-0.14	2.12E-10
492	cg22068211	C3orf27	-0.20	1.24E-14
493	cg02100848	C3orf32	-0.14	7.89E-08
494	cg04757243	C3orf36	-0.11	3.65E-07
495	cg18989919	C3orf36	-0.18	4.87E-11

496	cg05570980	C3orf52	0.22	3.62E-10
497	cg06807989	C3orf52	0.17	1.63E-06
498	cg18919097	C3orf57	0.14	5.22E-05
499	cg07109801	C3orf60	-0.20	5.31E-14
500	cg20835282	C3orf62	0.25	2.94E-07
501	cg07758574	C3orf62	0.14	1.13E-07
502	cg05659947	C4BPB	-0.19	1.69E-18
503	cg22197830	C5orf14	-0.15	4.81E-07
504	cg14722162	C5orf20	-0.17	1.19E-12
505	cg13349425	C5orf20	-0.27	8.14E-22
506	cg10257049	C5orf4	0.12	5.88E-04
507	cg05799317	C6orf118	-0.25	5.64E-18
508	cg24549507	C6orf145	0.20	7.22E-07
509	cg16150435	C6orf15	-0.13	6.52E-09
510	cg00463577	C6orf150	0.27	3.17E-09
511	cg09527362	C6orf150	0.13	3.58E-03
512	cg04600618	C6orf206	0.51	3.09E-30
513	cg01344171	C6orf206	0.19	9.09E-18
514	cg23973566	C6orf32	-0.11	1.97E-05
515	cg12818699	C6orf32	-0.20	4.96E-11
516	cg23216015	C7orf16	-0.15	5.08E-17
517	cg02564523	C7orf19	0.15	3.25E-04
518	cg10833091	C7orf23	0.15	1.51E-08
519	cg04999691	C7orf29	0.22	1.37E-13
520	cg22298208	C7orf34	-0.13	3.78E-09
521	cg04612566	C8orf31	-0.19	3.48E-12
522	cg15642035	C8ORFK32	-0.12	2.11E-05
523	cg06276653	C8ORFK32	-0.20	6.47E-11
524	cg00755043	C9orf121	0.36	4.95E-19
525	cg15785720	C9orf140	0.14	1.78E-04
526	cg01126560	C9orf142	0.11	5.48E-04
527	cg06484397	C9orf19	0.13	5.59E-05
528	cg17509612	C9orf78	0.10	1.55E-04
529	cg17191715	CA1	-0.14	5.82E-09
530	cg18503260	CA13	0.14	4.61E-06
531	cg18674980	CA3	0.34	1.03E-15
532	cg22289837	CA3	0.21	8.50E-11
533	cg04532952	CA4	0.21	4.26E-09
534	cg18743730	CA4	-0.12	1.32E-06
535	cg11667117	CA5A	-0.10	1.30E-04
536	cg12343082	CA5A	-0.14	1.65E-07
537	cg11258532	CA7	-0.19	1.38E-15
538	cg00983899	CABP2	-0.15	9.85E-13
539	cg18138484	CABP2	-0.17	5.33E-09
540	cg21518947	CABP4	-0.18	1.26E-14
541	cg09422355	CABP5	-0.29	2.46E-19
542	cg18534730	CABP5	-0.29	1.49E-20
543	cg20256494	CABP7	0.25	3.73E-09
544	cg11956146	CABYR	0.12	1.73E-03
545	cg19304410	CACHD1	0.30	2.23E-14

546	cg20876010	CACHD1	0.16	9.80E-08
547	cg25519930	CACNA1C	0.10	2.17E-03
548	cg01942127	CACNA1D	0.15	4.54E-06
549	cg11262815	CACNA1G	0.22	2.86E-09
550	cg05942574	CACNA1G	0.15	5.06E-10
551	cg18454685	CACNA1G	0.14	1.56E-04
552	cg00095526	CACNA1S	-0.14	4.21E-08
553	cg06379754	CACNA2D1	0.14	5.69E-05
554	cg20089715	CACNB1	-0.14	3.19E-15
555	cg04721098	CACNG3	-0.17	1.56E-18
556	cg06226384	CACNG5	-0.13	3.29E-18
557	cg22495124	CACNG5	-0.31	4.97E-17
558	cg11128808	CACNG6	-0.26	4.88E-15
559	cg09947274	CAD	-0.10	3.06E-04
560	cg06295856	CALCA	0.24	1.47E-10
561	cg09068492	CALCA	0.24	2.58E-10
562	cg14348532	CALCA	0.14	1.79E-06
563	cg10467022	CALCA	0.13	5.46E-06
564	cg22183706	CALCA	0.11	3.53E-06
565	cg01971122	CALCA	0.10	2.98E-05
566	cg06938878	CALCB	-0.18	1.74E-10
567	cg23239396	CALCR	0.14	1.20E-05
568	cg11828089	CALN1	-0.28	6.43E-17
569	cg21003606	CALN1	-0.33	1.96E-18
570	cg05497616	CAMK4	0.14	6.44E-05
571	cg13797282	CARD10	-0.13	3.09E-05
572	cg18321354	CARD11	-0.24	3.58E-15
573	cg05187322	CARD14	-0.17	1.95E-10
574	cg26683005	CARD8	0.13	3.10E-08
575	cg13802966	CASP1	-0.13	2.68E-16
576	cg16745604	CASP10	-0.10	3.87E-07
577	cg01999333	CASP14	-0.13	1.94E-09
578	cg09243021	CASP2	0.16	3.07E-09
579	cg17092637	CASP4	-0.15	7.00E-09
580	cg23410113	CASP8	-0.10	2.65E-22
581	cg09547777	CASQ1	-0.14	1.43E-09
582	cg18942631	CASQ2	-0.11	7.95E-10
583	cg05190718	CASQ2	-0.27	2.49E-22
584	cg17453778	CASR	0.13	7.23E-08
585	cg04759439	CAST1	0.24	3.27E-08
586	cg08365982	CAV3	-0.23	1.85E-14
587	cg24517042	CAV3	-0.27	6.36E-15
588	cg13745346	CBFA2T3	-0.21	4.23E-14
589	cg22780475	CBLC	-0.10	1.46E-03
590	cg15869642	CBLN1	-0.17	1.43E-13
591	cg25993718	CBLN4	0.11	2.37E-03
592	cg14564494	CBR3	0.19	8.83E-12
593	cg22633722	CBS	0.21	6.88E-08
594	cg02849695	CCDC19	0.30	7.83E-19
595	cg10051054	CCDC27	-0.20	9.21E-12

596	cg00891278	CCDC37	0.42	7.40E-36
597	cg20312228	CCDC37	0.23	7.42E-19
598	cg06468695	CCDC42	-0.12	3.56E-08
599	cg20099806	CCDC47	-0.11	6.46E-14
600	cg20131968	CCDC47	-0.16	1.48E-19
601	cg24287460	CCDC48	-0.17	1.43E-11
602	cg02498063	CCDC60	0.12	1.45E-10
603	cg02620769	CCDC65	0.29	2.24E-12
604	cg08958015	CCDC65	0.20	1.16E-08
605	cg11715966	CCDC8	-0.15	9.34E-08
606	cg15248035	CCIN	-0.10	1.37E-05
607	cg03724463	CCK	0.15	1.21E-06
608	cg04353483	CCKAR	-0.11	1.08E-07
609	cg15928132	CCKAR	-0.17	8.88E-12
610	cg17118262	CCL1	-0.14	3.46E-08
611	cg20556988	CCL1	-0.22	4.43E-16
612	cg24870391	CCL11	-0.26	2.24E-21
613	cg02706575	CCL13	-0.13	1.67E-11
614	cg24615251	CCL13	-0.16	1.05E-10
615	cg07881407	CCL14	0.11	1.94E-15
616	cg05766474	CCL16	-0.15	8.31E-06
617	cg08214029	CCL18	-0.24	1.18E-18
618	cg09425228	CCL20	-0.13	9.04E-08
619	cg21643045	CCL20	-0.31	2.68E-26
620	cg05556717	CCL26	-0.13	1.94E-06
621	cg12943082	CCL26	-0.14	1.11E-06
622	cg15005385	CCL3L1	-0.16	3.09E-08
623	cg25659818	CCL4	-0.20	1.25E-13
624	cg08124722	CCL7	-0.31	8.51E-22
625	cg01636591	CCL8	-0.12	1.66E-06
626	cg27000831	CCL8	-0.14	4.45E-14
627	cg16422907	CCNA1	0.19	4.80E-07
628	cg13060997	CCNA1	0.12	1.58E-04
629	cg09637363	CCND1	0.10	3.79E-04
630	cg12382902	CCND2	0.20	2.17E-08
631	cg00888007	CCND2	0.15	2.11E-06
632	cg16994506	CCND2	0.14	2.79E-06
633	cg02765328	CCND2	0.11	2.04E-04
634	cg25425078	CCND2	0.11	1.37E-05
635	cg08069899	CCND2	0.11	1.41E-04
636	cg06361108	CCNF	0.16	1.03E-05
637	cg04590978	CCNJ	0.40	4.29E-19
638	cg22864416	CCNJ	0.35	3.56E-11
639	cg09509673	CCR10	0.14	3.26E-15
640	cg04111761	CCR3	-0.13	5.63E-12
641	cg22022041	CCR9	-0.14	1.71E-10
642	cg16386080	CCRK	0.24	7.79E-14
643	cg12832565	CD160	0.11	3.99E-10
644	cg05436231	CD164L2	0.31	1.21E-13
645	cg14102807	CD19	-0.16	2.00E-11

646	cg07597976	CD19	-0.21	3.77E-16
647	cg07950803	CD1A	-0.30	6.98E-30
648	cg15952487	CD1B	-0.14	4.05E-17
649	cg17399166	CD1D	-0.18	8.50E-07
650	cg12200412	CD1E	-0.14	3.97E-23
651	cg01618851	CD209	-0.15	3.97E-10
652	cg15743985	CD22	-0.18	9.91E-08
653	cg13164537	CD226	0.14	9.89E-09
654	cg00350296	CD248	0.15	5.28E-10
655	cg17677397	CD300C	-0.18	8.80E-14
656	cg14173969	CD300C	-0.22	4.62E-15
657	cg04995095	CD300E	-0.23	2.13E-29
658	cg10523494	CD300LB	-0.11	8.54E-17
659	cg06407137	CD300LB	-0.19	8.09E-13
660	cg15374234	CD300LF	-0.24	9.61E-15
661	cg21640749	CD300LF	-0.35	4.99E-19
662	cg10129493	CD33	-0.32	1.12E-25
663	cg19591881	CD34	0.19	3.70E-08
664	cg15046675	CD37	0.10	1.39E-06
665	cg26043257	CD38	0.28	2.18E-10
666	cg17640322	CD44	0.11	1.72E-05
667	cg13311440	CD48	-0.16	8.26E-09
668	cg22325572	CD53	-0.13	4.24E-09
669	cg07380416	CD6	-0.20	2.04E-08
670	cg09902130	CD6	-0.24	5.70E-10
671	cg18557145	CD72	-0.24	1.18E-14
672	cg12971694	CD72	-0.29	5.07E-27
673	cg05921699	CD79A	-0.12	2.58E-10
674	cg25729716	CD79B	-0.10	9.95E-07
675	cg07973967	CD79B	-0.11	4.79E-03
676	cg21572897	CD80	-0.15	2.61E-12
677	cg04387658	CD86	-0.12	6.60E-07
678	cg17108819	CD8A	0.33	1.32E-12
679	cg02170525	CD8A	0.15	6.68E-08
680	cg26850754	CD8B1	0.25	6.29E-11
681	cg13164309	CDA	-0.21	7.79E-24
682	cg00750606	CDA	-0.23	2.88E-22
683	cg05469695	CDC42EP3	0.11	2.95E-04
684	cg08690031	CDCA7	0.18	4.30E-06
685	cg24975222	CDCP1	0.33	5.67E-14
686	cg17655614	CDH1	0.17	1.23E-08
687	cg11667754	CDH1	0.11	2.13E-04
688	cg19854301	CDH13	-0.20	1.49E-14
689	cg16777782	CDH13	-0.22	9.65E-14
690	cg02168291	CDH13	-0.22	7.71E-15
691	cg14792480	CDH16	-0.12	7.07E-07
692	cg20987610	CDH17	-0.23	8.76E-13
693	cg12038710	CDH17	-0.33	7.31E-17
694	cg03504701	CDH18	-0.39	2.13E-29
695	cg18509435	CDH20	-0.12	8.13E-11

696	cg04640913	CDH22	-0.11	1.12E-07
697	cg20895028	CDH26	-0.15	2.50E-09
698	cg24607535	CDH26	-0.19	6.63E-14
699	cg17733331	CDH3	0.10	5.77E-04
700	cg22319147	CDH5	-0.23	5.17E-12
701	cg08872742	CDH5	-0.25	1.23E-21
702	cg04155793	CDK10	-0.11	1.22E-07
703	cg07471052	CDK3	-0.13	9.13E-05
704	cg07658718	CDK5R1	0.13	1.53E-04
705	cg01920829	CDK5R2	0.17	4.67E-06
706	cg16077929	CDKL1	-0.12	4.37E-06
707	cg24432073	CDKL2	0.55	3.32E-34
708	cg14988503	CDKL2	0.49	2.94E-26
709	cg20919799	CDKN1C	0.34	6.17E-12
710	cg23548479	CDKN1C	0.34	2.63E-11
711	cg23146358	CDKN1C	0.27	5.52E-11
712	cg17738133	CDKN1C	0.17	1.71E-07
713	cg04548378	CDKN1C	0.14	6.82E-06
714	cg05559445	CDKN1C	0.14	1.64E-05
715	cg09099744	CDKN2A	0.59	2.40E-32
716	cg10895543	CDKN2A	0.45	1.94E-26
717	cg07752420	CDKN2A	0.43	1.29E-24
718	cg12840719	CDKN2A	0.33	6.50E-22
719	cg11653709	CDKN2A	0.28	6.78E-22
720	cg10210238	CDKN2B	0.28	3.26E-14
721	cg06421800	CDKN2B	0.18	3.59E-17
722	cg08390209	CDKN2B	0.11	1.01E-06
723	cg04675937	CDKN2B	0.11	2.32E-08
724	cg24735489	CDSN	-0.28	9.69E-20
725	cg08424423	CDSN	-0.34	1.41E-18
726	cg02055963	CDX2	0.19	9.92E-08
727	cg01424107	CDX2	0.12	2.99E-05
728	cg14831798	CDYL	-0.10	3.68E-06
729	cg09600520	CDYL	-0.14	1.48E-07
730	cg20657383	CEACAM1	-0.12	2.64E-07
731	cg23181133	CEACAM3	-0.10	2.55E-11
732	cg10237469	CEACAM4	-0.10	7.45E-17
733	cg21505334	CEACAM5	-0.27	6.03E-20
734	cg21126943	CEACAM6	-0.12	8.22E-15
735	cg26813458	CEACAM6	-0.33	4.38E-25
736	cg19623751	CEACAM7	-0.28	2.31E-25
737	cg07297178	CEACAM7	-0.30	3.66E-24
738	cg06475327	CEACAM8	-0.15	1.99E-08
739	cg02039171	CEBPE	-0.10	2.30E-11
740	cg18137704	CECR6	0.17	2.09E-10
741	cg06268694	CELSR1	0.38	7.05E-18
742	cg15879316	CELSR1	0.10	1.64E-04
743	cg06621358	CELSR3	0.31	3.94E-19
744	cg09736162	CELSR3	0.20	2.41E-05
745	cg05052633	CENTA2	0.21	2.71E-07

746	cg04856858	CENTG2	0.20	1.74E-09
747	cg24768561	CENTG2	0.17	7.81E-15
748	cg09425611	CES1	-0.12	4.82E-07
749	cg01474260	CESK1	-0.15	7.81E-12
750	cg07443748	CESK1	-0.16	5.12E-10
751	cg26738010	CETN1	-0.17	7.01E-11
752	cg17741572	CFB	-0.14	3.62E-07
753	cg17204100	CFC1	-0.30	1.41E-17
754	cg24974599	CFH	0.11	3.45E-09
755	cg25509184	CFTR	0.33	1.14E-17
756	cg07981495	CGA	-0.11	1.64E-06
757	cg00534856	CGB	-0.17	2.58E-15
758	cg20376767	CGB1	-0.13	1.74E-09
759	cg24908058	CGB5	-0.22	3.08E-20
760	cg03192551	CGI-69	0.30	5.38E-11
761	cg22740783	CGREF1	0.16	3.83E-04
762	cg23410627	CH25H	0.21	2.57E-08
763	cg06818777	CHAD	0.18	3.44E-12
764	cg06958829	CHAD	0.18	8.26E-12
765	cg13854874	CHAF1B	0.15	5.40E-05
766	cg08080029	CHD5	0.18	9.06E-06
767	cg00282347	CHD5	0.15	8.51E-06
768	cg00338702	CHFR	0.10	1.83E-03
769	cg12422450	CHGA	0.19	4.72E-07
770	cg15379858	ChGn	-0.22	2.50E-11
771	cg10045881	CHI3L2	-0.18	4.07E-11
772	cg21472642	CHN2	-0.11	4.75E-06
773	cg24130010	CHODL	0.15	7.29E-06
774	cg06319346	CHRFAM7A	-0.26	2.29E-22
775	cg13530039	CHRM1	-0.18	1.12E-13
776	cg04953015	CHRNA2	-0.10	1.60E-04
777	cg00318573	CHRNA4	0.14	1.94E-09
778	cg08912400	CHRNA4	0.12	6.29E-04
779	cg00367281	CHRNB3	-0.12	3.78E-07
780	cg27554782	CHRNB4	0.18	2.16E-07
781	cg11695684	CHRND	-0.10	6.33E-06
782	cg03853987	CHST10	0.34	3.17E-14
783	cg00995327	CHST2	0.24	1.01E-09
784	cg19731268	CHST3	0.23	1.68E-08
785	cg00840403	CHST4	0.15	3.76E-12
786	cg12827555	CHX10	0.11	4.95E-04
787	cg26112639	CIAS1	-0.18	1.54E-14
788	cg21991396	CIAS1	-0.29	8.44E-27
789	cg10313673	CILP2	0.28	3.21E-09
790	cg10669058	CILP2	0.17	1.87E-06
791	cg13316424	CIZ1	-0.11	4.00E-08
792	cg05786809	CKB	0.19	8.28E-06
793	cg20444256	CKM	-0.24	3.99E-22
794	cg00410921	CKMT1B	-0.11	3.24E-08
795	cg10978355	CKMT2	0.34	1.39E-16

796	cg07173760	CLC	-0.14	9.19E-12
797	cg13693652	CLCA2	-0.19	4.23E-17
798	cg21660130	CLCNKB	-0.13	6.36E-09
799	cg08418978	CLDN10	-0.16	2.64E-08
800	cg20449692	CLDN11	0.29	1.10E-08
801	cg11099899	CLDN14	-0.11	5.20E-06
802	cg02512860	CLDN15	-0.21	3.73E-08
803	cg13792279	CLDN17	-0.30	6.38E-24
804	cg10784090	CLDN18	-0.12	5.71E-07
805	cg17298704	CLDN18	-0.19	1.23E-10
806	cg15544036	CLDN4	0.13	2.26E-04
807	cg07384961	CLDN6	0.28	3.00E-12
808	cg10282491	CLDN9	0.16	3.45E-07
809	cg11905488	CLEC12A	-0.11	5.65E-08
810	cg20645058	CLEC12B	-0.21	1.21E-11
811	cg14473145	CLEC14A	0.21	6.82E-14
812	cg13354523	CLEC1A	-0.12	1.29E-05
813	cg21048669	CLEC3A	-0.12	8.33E-08
814	cg21057494	CLEC3B	-0.18	2.11E-12
815	cg22194129	CLEC4C	-0.12	2.19E-07
816	cg09546307	CLEC4D	-0.24	1.83E-16
817	cg14162076	CLEC4D	-0.34	9.87E-26
818	cg22984277	CLEC4E	-0.11	3.73E-16
819	cg18199266	CLEC4F	-0.12	7.23E-08
820	cg21148892	CLEC4F	-0.28	2.88E-15
821	cg04816348	CLEC4G	-0.14	1.40E-06
822	cg16626670	CLEC4G	-0.21	1.05E-12
823	cg21372914	CLEC4M	-0.19	2.90E-11
824	cg18463686	CLEC5A	-0.17	7.79E-14
825	cg20098659	CLEC9A	-0.13	3.15E-08
826	cg17469479	CLEC9A	-0.16	7.38E-09
827	cg06432655	CLIPR-59	0.29	2.80E-21
828	cg25107791	CLPS	-0.24	4.03E-17
829	cg07251788	CLTCL1	-0.18	1.08E-08
830	cg06933965	CMKLR1	-0.12	4.19E-06
831	cg00298951	CMKLR1	-0.27	1.69E-17
832	cg01683883	CMTM2	0.34	1.64E-16
833	cg23297477	CMTM3	0.30	6.19E-10
834	cg00174500	CMTM5	-0.19	7.80E-11
835	cg10560038	CMTM5	-0.22	1.40E-13
836	cg01617750	CMTM8	-0.11	2.66E-04
837	cg11500727	CNFN	-0.13	4.22E-07
838	cg19000186	CNGA1	-0.17	4.57E-11
839	cg15954792	CNGA3	-0.19	2.39E-12
840	cg22241124	CNGA3	-0.23	1.60E-13
841	cg16480209	CNGB1	-0.15	9.38E-09
842	cg00290506	CNIH3	0.30	2.43E-11
843	cg01968793	CNNM1	0.13	1.46E-04
844	cg23276695	CNR1	-0.14	2.47E-12
845	cg03732545	CNTN2	0.16	8.52E-14

846	cg10503138	CNTN4	-0.24	4.11E-23
847	cg05209917	CNTN6	-0.15	2.83E-10
848	cg07028533	CNTNAP2	0.18	3.06E-08
849	cg16254309	CNTNAP2	0.16	1.51E-05
850	cg25383242	CNTNAP4	-0.19	2.70E-13
851	cg06793062	CNTNAP4	-0.22	1.32E-10
852	cg17031727	COG2	0.19	4.14E-10
853	cg10143146	COL11A2	0.24	1.70E-10
854	cg08009622	COL12A1	0.12	1.04E-05
855	cg16907566	COL14A1	0.23	1.93E-14
856	cg20503329	COL15A1	-0.14	1.25E-06
857	cg20546002	COL23A1	0.26	1.55E-11
858	cg10730712	COL23A1	0.21	1.25E-07
859	cg01291404	COL2A1	0.22	1.84E-08
860	cg21513553	COL6A2	0.12	1.59E-03
861	cg00573606	COL6A3	-0.20	2.44E-11
862	cg11846236	COL7A1	0.35	1.29E-18
863	cg18931815	COL8A2	-0.12	6.87E-04
864	cg14579051	COL9A1	-0.14	2.04E-08
865	cg21789545	COL9A1	-0.21	2.26E-19
866	cg05755779	COLEC10	-0.16	1.06E-06
867	cg09949775	COMP	0.18	2.67E-05
868	cg15926585	COMT	-0.17	5.50E-08
869	cg24831427	COQ3	-0.11	9.16E-06
870	cg20015855	CORO2B	-0.10	2.69E-05
871	cg08290628	CORO2B	-0.16	1.17E-12
872	cg13623690	COTL1	0.23	3.23E-09
873	cg22677246	COTL1	0.10	4.73E-06
874	cg09027725	COX4I2	-0.15	9.32E-12
875	cg24335895	COX7A1	0.11	1.35E-08
876	cg27651218	COX8C	-0.10	6.91E-10
877	cg14761227	CPA1	-0.23	1.61E-13
878	cg22213042	CPA2	-0.11	1.14E-05
879	cg24060938	CPA2	-0.15	1.14E-08
880	cg24290574	CPA3	-0.11	9.29E-07
881	cg13424229	CPA3	-0.14	3.18E-07
882	cg00845900	CPA4	-0.20	3.70E-12
883	cg02855426	CPA5	-0.11	5.28E-16
884	cg21750887	CPA6	-0.21	1.73E-09
885	cg19885761	CPLX2	0.19	9.08E-07
886	cg22776578	CPLX2	0.10	2.09E-04
887	cg16673198	CPNE4	-0.21	4.63E-17
888	cg21716693	CPNE5	0.10	1.77E-03
889	cg16444968	CPNE7	0.25	2.22E-10
890	cg07690018	CPNE7	0.12	2.13E-04
891	cg16191009	CPNE9	0.17	1.31E-19
892	cg20051033	CPNE9	0.16	1.13E-06
893	cg03731898	CPO	-0.16	1.44E-09
894	cg06194186	CPO	-0.23	1.11E-19
895	cg19596755	CPT1A	0.18	7.67E-06

896	cg00983520	CPT1B	0.11	1.57E-03
897	cg21604803	CPT1C	0.25	1.99E-12
898	cg14726637	CR1	0.22	3.01E-11
899	cg11200929	CRABP1	0.23	2.33E-10
900	cg17133183	CRABP1	0.12	3.19E-05
901	cg23337754	CRABP1	0.12	4.18E-05
902	cg03258472	CRB3	0.24	2.35E-17
903	cg20329958	CRH	0.13	2.62E-07
904	cg08929103	CRHR1	-0.21	6.39E-11
905	cg04922810	CRHR2	0.24	6.47E-11
906	cg14896516	CRHR2	0.22	8.12E-08
907	cg02000005	CRIP1	0.15	4.45E-04
908	cg03451296	CRIP2	0.17	1.78E-04
909	cg01410472	CRISPLD1	0.20	1.56E-09
910	cg23606079	CRISPLD2	0.12	4.01E-06
911	cg07207789	CRISPLD2	-0.17	3.69E-11
912	cg22680204	CRMP1	0.33	5.06E-23
913	cg03544320	CRMP1	0.29	3.07E-14
914	cg16713808	CRNN	-0.25	5.62E-12
915	cg19370451	CRNN	-0.33	1.41E-22
916	cg10977115	CRTAM	-0.12	5.87E-10
917	cg22512531	CRTAM	-0.13	1.95E-08
918	cg22386774	CRYGA	-0.18	4.03E-11
919	cg12073779	CRYGD	0.12	2.10E-04
920	cg04569804	CSF1R	-0.10	2.81E-05
921	cg02196805	CSF2	-0.12	1.06E-09
922	cg13259290	CSF2	-0.13	2.90E-12
923	cg21209356	CSF2RB	-0.14	1.91E-05
924	cg21432842	CSF3	-0.16	4.40E-09
925	cg09088576	CSF3R	0.14	7.75E-09
926	cg11880211	CSH1	-0.16	2.22E-15
927	cg24498554	CSH1	-0.23	1.11E-15
928	cg27178345	CSH2	-0.14	1.21E-14
929	cg09585781	CSH2	-0.18	1.32E-17
930	cg01668383	CSNK1A1L	-0.23	1.12E-14
931	cg25306170	CSNK1A1L	-0.27	1.80E-19
932	cg06952310	CSPG3	-0.22	1.35E-18
933	cg13118849	CSPG3	-0.35	4.91E-22
934	cg15988232	CSPG5	0.10	1.22E-03
935	cg19216731	CSRP3	-0.20	8.90E-21
936	cg14704941	CSRP3	-0.28	4.91E-21
937	cg23660082	CST1	-0.13	8.72E-14
938	cg08430598	CST1	-0.30	4.43E-18
939	cg17928268	CST11	-0.24	3.16E-23
940	cg06489008	CST11	-0.25	4.10E-17
941	cg01237132	CST2	-0.12	3.65E-12
942	cg10938286	CST2	-0.37	1.53E-22
943	cg25965576	CST4	-0.10	2.15E-07
944	cg24875415	CST4	-0.32	1.04E-17
945	cg02882813	CST5	-0.26	3.17E-15

946	cg27655855	CST9L	-0.16	2.25E-09
947	cg15210427	CST9L	-0.21	9.37E-10
948	cg14540150	CTBP2	0.15	9.99E-06
949	cg04233664	CTBP2	0.10	1.43E-03
950	cg26952662	CTHRC1	0.36	3.71E-13
951	cg08460026	CTLA4	-0.12	1.29E-07
952	cg15043057	CTPS	0.32	2.32E-19
953	cg21686987	CTRB1	-0.13	1.74E-12
954	cg23064554	CTRC	-0.11	5.96E-09
955	cg21478437	CTSE	-0.12	5.72E-09
956	cg24355048	CTSG	-0.17	9.49E-19
957	cg24777950	CTSG	-0.20	2.20E-10
958	cg13234643	CTSL2	0.19	5.33E-06
959	cg02936872	CTSL2	0.19	5.56E-06
960	cg01623438	CTSZ	0.11	6.58E-11
961	cg27603796	CTTNBP2	0.20	2.83E-10
962	cg15777781	CUGBP2	-0.18	3.47E-10
963	cg00168942	CX40.1	-0.20	4.41E-13
964	cg23900225	CX40.1	-0.23	2.99E-14
965	cg08947964	CX62	-0.16	4.80E-09
966	cg00744433	CXADR	-0.11	2.65E-06
967	cg00499822	CXCL12	-0.13	1.23E-03
968	cg18618334	CXCL12	-0.14	6.60E-04
969	cg10088985	CXCL5	0.15	5.21E-07
970	cg22670329	CXCL6	0.14	1.14E-07
971	cg21859434	CXCR4	0.12	1.12E-05
972	cg14204735	CYB561	0.19	1.38E-07
973	cg03826976	CYB5R2	0.30	3.13E-25
974	cg06885524	CYBRD1	0.13	1.20E-04
975	cg17040807	CYGB	0.17	4.58E-12
976	cg21301440	CYGB	0.15	3.84E-07
977	cg09120035	CYP11B1	-0.45	8.68E-36
978	cg10500909	CYP11B2	-0.20	1.96E-16
979	cg09201719	CYP17A1	-0.11	1.45E-05
980	cg14694011	CYP19A1	-0.10	1.96E-07
981	cg04968473	CYP1A2	0.13	4.55E-09
982	cg09207718	CYP1A2	-0.19	2.38E-11
983	cg18956481	CYP24A1	0.31	3.79E-16
984	cg04884908	CYP26B1	0.13	2.50E-04
985	cg20322977	CYP26C1	0.17	1.55E-11
986	cg00436603	CYP2E1	-0.10	2.51E-05
987	cg13364756	CYP3A43	-0.15	1.78E-08
988	cg16689634	CYP4X1	0.32	3.67E-19
989	cg13158571	CYP4X1	0.31	8.92E-18
990	cg10751811	CYP4Z1	-0.12	1.15E-10
991	cg14404298	CYP8B1	-0.11	7.00E-09
992	cg21979032	CYP8B1	-0.16	3.40E-08
993	cg00512031	CYTL1	-0.28	7.67E-19
994	cg14837165	D4ST1	0.29	1.43E-08
995	cg06710648	DAB1	0.13	5.15E-07

996	cg00848728	DAB1	0.11	2.60E-04
997	cg05684891	DAB2IP	0.48	1.49E-33
998	cg07981910	DAB2IP	0.14	9.42E-11
999	cg13060154	DAB2IP	0.13	3.09E-14
1000	cg15686608	DAB2IP	0.12	1.01E-11
1001	cg08128768	DAB2IP	0.12	6.31E-08
1002	cg27650175	DAB2IP	0.11	7.19E-10
1003	cg07817783	DAP	0.18	7.51E-07
1004	cg23165541	DAPK2	-0.18	8.24E-11
1005	cg23507131	DARC	-0.21	3.52E-19
1006	cg18552413	DARC	-0.23	2.80E-13
1007	cg25020204	DBH	-0.18	1.38E-11
1008	cg07824742	DBH	-0.19	1.76E-10
1009	cg22605063	DBN1	0.24	7.69E-09
1010	cg23983884	DBN1	0.14	5.19E-10
1011	cg17470143	DCAMKL1	-0.13	1.22E-09
1012	cg02624705	DCC	0.11	1.81E-04
1013	cg18572014	DCC	-0.14	3.00E-29
1014	cg13396068	DCD	-0.19	1.12E-11
1015	cg25372195	DCD	-0.28	7.83E-20
1016	cg16306115	DCDC2	0.43	3.82E-18
1017	cg04515001	DCDC2	0.36	3.83E-15
1018	cg04517429	DCHS2	0.11	1.09E-05
1019	cg07903860	DCLRE1C	0.12	2.43E-03
1020	cg06785429	DCUN1D1	0.12	2.24E-08
1021	cg18055007	DDAH2	0.17	1.48E-07
1022	cg11155265	DDEFL1	0.15	1.57E-08
1023	cg22740835	DDR2	0.14	8.91E-17
1024	cg17188169	DDX43	-0.15	4.38E-06
1025	cg14757492	DDX49	-0.23	4.05E-16
1026	cg04761824	DEC1	-0.15	2.12E-14
1027	cg07294541	DEF6	0.13	1.98E-03
1028	cg14960043	DEF6	0.12	2.31E-03
1029	cg10758292	DEFA1	-0.19	1.46E-14
1030	cg17267907	DEFA1	-0.25	1.80E-19
1031	cg22218909	DEFA3	-0.25	1.40E-17
1032	cg19304352	DEFA4	-0.12	2.56E-07
1033	cg15481539	DEFA5	-0.25	5.57E-15
1034	cg27212977	DEFA6	-0.19	1.87E-13
1035	cg25214366	DEFB103A	-0.20	1.83E-13
1036	cg14120879	DEFB105A	-0.19	3.49E-14
1037	cg08753553	DEFB106A	-0.14	1.71E-10
1038	cg05810550	DEFB106A	-0.16	1.08E-14
1039	cg03014957	DEFB118	-0.18	1.74E-19
1040	cg20312687	DEFB118	-0.21	2.77E-20
1041	cg18462653	DEFB119	-0.17	8.21E-16
1042	cg26063872	DEFB123	-0.24	3.87E-18
1043	cg19241311	DEFB123	-0.29	6.10E-19
1044	cg08088390	DEFB125	-0.15	6.12E-15
1045	cg00466436	DEFB126	-0.13	1.65E-12

1046	cg20305726	DEFB126	-0.22	5.05E-20
1047	cg00769470	DEFB129	-0.15	4.36E-11
1048	cg18239253	DEFB32	-0.14	2.71E-08
1049	cg22478614	DEFB4	-0.23	4.38E-25
1050	cg02658251	DEFB4	-0.27	2.84E-22
1051	cg23213217	DEGS1	-0.17	4.29E-07
1052	cg08307816	DEGS2	0.33	1.67E-14
1053	cg00890257	DEGS2	0.31	4.88E-11
1054	cg24641737	DENND2D	0.16	2.78E-08
1055	cg00619207	DENND2D	0.11	3.96E-06
1056	cg24473633	DENND3	0.23	3.66E-09
1057	cg26259363	DES	-0.14	6.63E-10
1058	cg01344452	DGKE	0.37	1.07E-10
1059	cg06277657	DGKI	0.35	9.63E-17
1060	cg06577005	DHDDS	0.17	4.81E-09
1061	cg15974053	DHRS10	-0.14	6.86E-06
1062	cg26588422	DHX33	0.10	5.72E-05
1063	cg00217795	DIO2	-0.11	1.03E-05
1064	cg04623955	DIO3	0.28	3.92E-12
1065	cg21808053	DIRAS3	-0.21	5.09E-11
1066	cg10942056	DISP1	-0.12	1.30E-05
1067	cg06595693	DISP2	0.18	4.92E-08
1068	cg17983307	DKFZp434B1231	-0.18	2.17E-14
1069	cg17886204	DKFZp434I1020	0.38	8.27E-22
1070	cg19394737	DKFZp434O0527	-0.12	2.05E-08
1071	cg13379763	DKFZp434O0527	-0.21	4.96E-15
1072	cg17757055	DKFZP564O0823	0.13	1.15E-04
1073	cg24890043	DKFZP566N034	0.22	1.33E-06
1074	cg13216057	DKK3	0.21	1.03E-07
1075	cg21589115	DKKL1	0.10	1.19E-02
1076	cg23881725	DLEC1	0.30	2.90E-17
1077	cg00807586	DLEC1	0.20	5.78E-08
1078	cg16986720	DLG2	-0.23	2.19E-15
1079	cg16128363	DLGAP1	-0.25	2.20E-12
1080	cg09276978	DLGAP4	-0.29	1.04E-18
1081	cg17412258	DLK1	0.18	3.44E-07
1082	cg16652259	DLX1	0.24	2.59E-09
1083	cg15236866	DLX1	0.11	1.50E-06
1084	cg03657766	DLX2	0.18	8.26E-07
1085	cg01169726	DLX5	0.37	1.07E-20
1086	cg00503840	DLX5	0.36	2.10E-30
1087	cg27016494	DLX5	0.35	2.25E-20
1088	cg20080624	DLX5	0.34	5.79E-16
1089	cg13462129	DLX5	0.31	1.65E-16
1090	cg18873386	DLX5	0.30	7.35E-19
1091	cg11500797	DLX5	0.29	1.98E-21
1092	cg16924616	DLX5	0.18	1.53E-05
1093	cg12041387	DLX5	0.13	8.12E-08
1094	cg06911084	DLX5	0.13	6.68E-05
1095	cg00096922	DLX5	0.12	1.27E-06

1096	cg13344740	DLX5	0.11	9.03E-05
1097	cg20120491	DMC1	0.11	2.15E-05
1098	cg13191049	DMN	0.34	3.03E-10
1099	cg05881135	DMN	0.19	4.45E-06
1100	cg11530960	DMRT2	0.13	1.59E-06
1101	cg25957124	DNAH3	0.21	2.48E-11
1102	cg03503295	DNAH5	-0.19	1.74E-12
1103	cg06799664	DNAHL1	-0.26	2.44E-17
1104	cg23753610	DNAHL1	-0.36	2.92E-21
1105	cg23579062	DNAI1	0.15	1.40E-17
1106	cg08578641	DNAI1	0.15	5.07E-09
1107	cg11856697	DNAI2	-0.18	1.43E-13
1108	cg23366752	DNAJA4	-0.11	5.00E-07
1109	cg07061145	DNAJA4	-0.16	2.29E-13
1110	cg15712267	DNAJB1	-0.11	2.42E-04
1111	cg11695266	DNAJB6	0.16	3.09E-06
1112	cg16953612	DNAJB8	-0.20	2.18E-12
1113	cg03022541	DNAJB8	-0.27	3.60E-21
1114	cg02067021	DNAJC5B	-0.11	3.40E-04
1115	cg13760253	DNAJC5B	-0.17	2.70E-11
1116	cg12619509	DNASE1L2	0.16	3.95E-05
1117	cg23391785	DNM3	0.54	1.51E-32
1118	cg21629895	DNMT3A	-0.13	6.50E-09
1119	cg24949488	DNTT	-0.26	8.82E-24
1120	cg22807700	DNTTIP2	-0.16	4.64E-09
1121	cg21233722	DOCK2	0.22	4.95E-09
1122	cg26117023	DOK1	0.45	2.56E-22
1123	cg20965815	DPCR1	-0.13	3.16E-09
1124	cg04645843	DPCR1	-0.24	4.49E-19
1125	cg10922280	DPEP2	-0.10	7.95E-07
1126	cg04774694	DPEP2	-0.13	2.40E-24
1127	cg11516377	DPH1	0.14	3.58E-06
1128	cg26738880	DPP6	-0.14	3.90E-12
1129	cg06872381	DPPA3	-0.11	1.70E-08
1130	cg08284151	DPPA3	-0.12	2.57E-10
1131	cg20774846	DPYS	-0.11	1.45E-13
1132	cg07295678	DPYSL4	0.23	2.02E-08
1133	cg12109455	DPYSL4	0.16	7.40E-06
1134	cg16112129	DRD1	0.24	6.40E-08
1135	cg17307280	DRD1	0.13	1.12E-04
1136	cg21965997	DRD1IP	0.21	3.40E-08
1137	cg12758687	DRD2	0.26	5.28E-13
1138	cg06825142	DRD4	0.31	4.78E-09
1139	cg09936561	DRD5	0.28	7.33E-16
1140	cg21279601	DRD5	0.25	1.47E-11
1141	cg04180953	DSC1	-0.15	1.06E-07
1142	cg18356799	DSC1	-0.16	6.47E-10
1143	cg11832722	DSC3	0.26	2.24E-09
1144	cg15439862	DSC3	0.23	2.96E-09
1145	cg19703610	DSCAML1	0.22	1.74E-08

1146	cg00544557	DSCR10	-0.17	6.75E-11
1147	cg13460409	DSCR6	0.14	9.17E-08
1148	cg01262913	DSCR9	0.16	4.72E-07
1149	cg02668581	DSU	0.39	8.43E-14
1150	cg12620499	DSU	0.22	1.70E-06
1151	cg18719571	DTNBP1	-0.17	5.53E-09
1152	cg05732530	DUSP2	0.10	2.29E-03
1153	cg01148741	DUSP2	0.10	2.57E-03
1154	cg18070061	DUSP4	0.33	1.85E-12
1155	cg13983578	DUSP4	0.16	3.81E-06
1156	cg25751295	DUSP4	0.12	6.25E-05
1157	cg18396533	DYDC1	0.14	3.12E-16
1158	cg10281478	DYNC1I1	0.11	1.14E-04
1159	cg06270401	DYRK4	-0.12	6.01E-07
1160	cg09418321	DYRK4	-0.13	1.11E-09
1161	cg08433095	DYX1C1	-0.18	1.83E-12
1162	cg16593081	DYX1C1	-0.18	3.28E-11
1163	cg04101379	DZIP1	0.12	2.35E-04
1164	cg00756058	DZIP1	0.11	1.50E-03
1165	cg05056120	EBF	0.29	2.67E-17
1166	cg16592658	EBI3	-0.19	2.37E-15
1167	cg06819200	ECE1	0.35	1.75E-11
1168	cg25431974	ECEL1	0.14	1.49E-12
1169	cg02932167	ECEL1	0.10	1.45E-08
1170	cg24428042	ECHDC3	0.13	2.87E-03
1171	cg10885338	ECRG4	0.10	1.90E-04
1172	cg10521852	EDG4	0.34	2.85E-24
1173	cg06501070	EDG7	-0.15	1.27E-10
1174	cg24574111	EDG7	-0.16	1.03E-09
1175	cg27238470	EDN2	-0.11	1.35E-05
1176	cg21163415	EDN3	-0.19	2.38E-16
1177	cg23316360	EDNRB	0.17	3.87E-07
1178	cg22463915	EEF1A2	0.15	2.52E-06
1179	cg22836229	EFCAB1	0.32	7.75E-13
1180	cg11868900	EFCAB1	0.22	1.38E-09
1181	cg27513764	EFCAB3	-0.24	4.26E-21
1182	cg02899772	EFCBP2	0.12	2.07E-02
1183	cg20786074	EFEMP1	0.15	8.21E-08
1184	cg15230273	EFNA5	0.20	2.33E-07
1185	cg24929737	EFNB2	0.39	7.32E-18
1186	cg27226949	EFNB2	0.30	2.83E-13
1187	cg01013324	EFNB3	0.14	1.56E-05
1188	cg11460029	EFNB3	0.13	5.51E-05
1189	cg07197059	EFS	-0.18	6.14E-08
1190	cg24818418	EGF	-0.14	7.66E-11
1191	cg13379236	EGF	-0.15	2.40E-09
1192	cg05037688	EGFL7	-0.30	8.34E-23
1193	cg24236938	EGFL9	-0.10	8.85E-05
1194	cg19355190	EGR2	0.17	6.82E-06
1195	cg25811575	EGR3	0.12	4.63E-04

1196	cg18414381	EHF	0.10	4.60E-05
1197	cg16019273	EIF2C4	-0.10	5.50E-04
1198	cg10541755	EIF5A2	0.30	5.21E-11
1199	cg26209058	EIF5A2	0.18	1.91E-07
1200	cg01963696	ELA2	-0.15	3.55E-09
1201	cg25332298	ELAVL3	0.18	4.71E-07
1202	cg18242139	ELAVL4	-0.25	6.46E-14
1203	cg19439399	ELOVL4	0.16	1.57E-06
1204	cg13297865	ELOVL4	0.16	1.12E-05
1205	cg08981777	ELSPBP1	-0.12	9.14E-12
1206	cg27019278	EMCN	-0.22	3.02E-15
1207	cg09009111	EMILIN2	0.29	6.94E-11
1208	cg14133708	EMILIN2	0.12	7.46E-07
1209	cg18938204	EMILIN3	0.15	9.42E-06
1210	cg15746620	EMR3	-0.10	1.14E-11
1211	cg15552238	EMR3	-0.16	6.56E-24
1212	cg21351994	EMX1	0.11	1.44E-03
1213	cg19358493	EMX2	0.15	1.47E-06
1214	cg24910675	ENG	0.13	8.42E-07
1215	cg16112050	ENOSF1	-0.10	3.02E-03
1216	cg04006554	ENPP5	0.13	2.03E-05
1217	cg08493463	ENPP6	-0.11	4.81E-06
1218	cg17348429	ENPP7	-0.14	1.31E-07
1219	cg26087862	ENSA	-0.15	4.20E-20
1220	cg17018517	ENTPD2	0.20	5.07E-09
1221	cg17264618	ENTPD3	0.34	1.57E-22
1222	cg17200465	ENTPD3	0.30	8.21E-27
1223	cg12858460	EOMES	0.18	1.00E-06
1224	cg15540820	EOMES	0.13	2.96E-05
1225	cg14324838	EPB41L1	-0.13	2.55E-03
1226	cg20993403	EPB41L1	-0.13	4.12E-06
1227	cg00027083	EPB41L3	0.14	1.58E-03
1228	cg15146752	EPHA2	0.11	4.06E-03
1229	cg18055394	EPHA3	-0.12	2.52E-12
1230	cg03776464	EPHA4	0.25	7.27E-10
1231	cg18420965	EPHA5	0.10	5.53E-04
1232	cg08001895	EPHA7	0.16	4.54E-08
1233	cg00044245	EPHA7	0.11	5.22E-08
1234	cg16382256	EPHB3	0.13	1.17E-05
1235	cg02593766	EPN3	-0.24	1.60E-21
1236	cg08575537	EPO	-0.13	4.54E-08
1237	cg24477567	EPOR	0.14	9.35E-10
1238	cg27105123	EPS8L1	-0.12	2.99E-14
1239	cg00491404	EPS8L3	-0.11	1.38E-05
1240	cg00515905	EPS8L3	-0.19	5.48E-10
1241	cg07115820	EPX	-0.16	1.68E-09
1242	cg02989940	ERAF	-0.22	2.46E-12
1243	cg20637307	ERBB4	0.11	8.56E-04
1244	cg21349901	ERVK6	-0.12	6.94E-24
1245	cg20451680	ESM1	-0.12	4.29E-04

1246	cg07233761	ESM1	-0.15	4.58E-14
1247	cg13066963	ESPN	0.22	1.71E-10
1248	cg00655307	ESR1	0.22	3.35E-08
1249	cg07671949	ESR1	0.22	2.90E-09
1250	cg20627916	ESR1	0.17	9.94E-09
1251	cg11251858	ESR1	0.16	8.48E-05
1252	cg11059483	ESR2	0.18	5.03E-07
1253	cg16792632	ESR2	0.14	1.22E-05
1254	cg26298099	ESRRB	-0.23	5.85E-13
1255	cg07864297	ESRRB	-0.24	3.53E-18
1256	cg04289385	ETV7	0.19	3.66E-06
1257	cg03278643	EVC2	-0.13	1.53E-04
1258	cg23352695	EVI2A	0.11	2.16E-05
1259	cg27626299	EVX1	0.31	5.07E-15
1260	cg18397523	EVX1	0.19	2.59E-09
1261	cg24176563	EYA4	0.22	5.08E-08
1262	cg26656135	EYA4	0.20	3.44E-07
1263	cg21296676	EYA4	0.17	5.92E-07
1264	cg20286200	EYA4	0.16	3.13E-08
1265	cg01805282	EYA4	0.14	4.41E-04
1266	cg20330472	EYA4	0.12	2.54E-05
1267	cg07327468	EYA4	0.11	2.12E-08
1268	cg26608199	F10	-0.16	8.86E-09
1269	cg21367957	F10	-0.20	2.04E-10
1270	cg06625767	F12	-0.12	9.34E-06
1271	cg12332316	F12	-0.14	3.56E-07
1272	cg20199333	F2	-0.14	2.33E-07
1273	cg11591325	F2R	0.36	6.23E-18
1274	cg24573501	F2RL1	0.11	4.06E-06
1275	cg08241785	F2RL2	0.11	6.91E-08
1276	cg25256723	F5	-0.14	2.56E-15
1277	cg24269657	F7	-0.13	3.13E-06
1278	cg19910382	FABP1	-0.12	2.62E-06
1279	cg13599007	FABP6	-0.13	1.46E-05
1280	cg22861316	FABP6	-0.36	4.39E-24
1281	cg06781209	FADS2	0.22	1.64E-06
1282	cg25936385	FAIM2	-0.14	4.88E-10
1283	cg21453309	FAM101A	-0.17	3.26E-08
1284	cg23639989	FAM105A	0.19	6.90E-08
1285	cg01794265	FAM105A	0.16	1.59E-04
1286	cg21279865	FAM106A	-0.10	9.42E-13
1287	cg12285118	FAM107B	-0.16	1.05E-12
1288	cg02876062	FAM107B	-0.28	1.79E-21
1289	cg04450876	FAM112B	-0.26	1.69E-16
1290	cg20576002	FAM112B	-0.30	2.50E-15
1291	cg05596756	FAM113B	0.12	2.23E-07
1292	cg03569637	FAM113B	0.11	3.02E-07
1293	cg15842430	FAM12B	-0.19	1.93E-11
1294	cg04536922	FAM13A1	0.16	6.52E-09
1295	cg13921352	FAM19A4	0.12	1.03E-03

1296	cg07895149	FAM26B	0.13	1.84E-04
1297	cg09155001	FAM26C	-0.20	6.13E-16
1298	cg03158400	FAM3B	0.16	8.85E-05
1299	cg16352283	FAM46B	0.28	2.52E-16
1300	cg06073471	FAM55C	0.28	4.82E-12
1301	cg15646782	FAM55C	0.19	5.98E-08
1302	cg25186143	FAM57A	0.13	4.46E-04
1303	cg13271951	FAM57B	0.11	1.48E-06
1304	cg07078114	FAM69B	0.24	1.10E-15
1305	cg10531372	FAM71A	-0.11	1.65E-11
1306	cg14376424	FAM71B	-0.22	3.16E-14
1307	cg13338132	FAM71C	-0.22	5.94E-09
1308	cg12864581	FAM77C	-0.22	4.56E-14
1309	cg12998491	FAM78A	0.46	9.60E-22
1310	cg17936488	FAM78A	0.11	1.88E-05
1311	cg16534233	FAM80A	0.18	3.32E-05
1312	cg13199589	FAM80A	0.14	7.27E-05
1313	cg21771250	FAM83F	-0.12	4.47E-04
1314	cg03954858	FAM83F	-0.23	1.07E-16
1315	cg16516400	FAM89A	0.23	1.97E-09
1316	cg04443324	FAT2	-0.30	4.50E-18
1317	cg07846167	FBLIM1	0.12	2.83E-03
1318	cg15143643	FBLN1	-0.14	2.28E-08
1319	cg04337944	FBLN1	-0.17	2.92E-09
1320	cg16604516	FBLN2	0.21	6.99E-08
1321	cg18671950	FBN1	0.33	3.09E-15
1322	cg27223047	FBN2	0.24	3.24E-11
1323	cg25084878	FBN2	0.23	7.10E-13
1324	cg01934790	FBXL16	0.13	8.03E-06
1325	cg02699167	FBXL2	0.21	8.36E-09
1326	cg02630888	FBXL5	-0.16	4.41E-07
1327	cg08820801	FBXO17	-0.15	6.33E-08
1328	cg01680762	FBXO25	-0.11	5.55E-06
1329	cg20723355	FBXO39	0.22	7.33E-05
1330	cg02613386	FBXO39	0.21	5.59E-05
1331	cg07125166	FBXW12	-0.11	3.94E-08
1332	cg03112869	FBXW12	-0.15	7.48E-10
1333	cg13853198	FCER1G	0.10	3.59E-10
1334	cg12387247	FCER2	-0.12	1.07E-06
1335	cg03221619	FCER2	-0.20	1.63E-17
1336	cg19103704	FCGBP	-0.10	1.06E-09
1337	cg24030627	FCGBP	-0.26	8.01E-13
1338	cg19464944	FCGR1A	-0.14	1.14E-20
1339	cg10904672	FCGR1A	-0.15	1.33E-16
1340	cg22202141	FCGR3A	-0.12	9.77E-07
1341	cg00974864	FCGR3B	-0.11	3.03E-04
1342	cg04567009	FCGR3B	-0.12	1.59E-06
1343	cg17357062	FCN1	-0.26	3.77E-22
1344	cg06244417	FCN1	-0.29	2.34E-22
1345	cg26164184	FCN2	-0.21	4.88E-16

1346	cg11884243	FCN2	-0.26	3.24E-13
1347	cg11921829	FCRL2	-0.12	2.22E-10
1348	cg25259754	FCRL3	-0.15	2.13E-29
1349	cg03329572	FCRL5	-0.32	1.83E-21
1350	cg26015133	FCRLM2	-0.18	2.37E-17
1351	cg27495845	FCRLM2	-0.18	6.51E-12
1352	cg18661868	FES	0.20	3.88E-09
1353	cg24888049	FES	0.10	1.02E-04
1354	cg08221207	FEZ1	0.13	3.79E-06
1355	cg03782727	FFAR1	-0.15	2.16E-12
1356	cg06090864	FFAR1	-0.15	9.70E-13
1357	cg15479752	FFAR2	-0.21	3.49E-12
1358	cg02017155	FFAR3	-0.22	3.78E-13
1359	cg15512851	FGD2	-0.11	1.01E-08
1360	cg19954000	FGF1	-0.18	3.63E-11
1361	cg08816023	FGF1	-0.20	2.14E-14
1362	cg16293656	FGF17	0.20	1.09E-08
1363	cg15774153	FGF19	0.37	6.73E-12
1364	cg26096837	FGF19	0.22	1.33E-16
1365	cg17214107	FGF2	0.12	3.06E-08
1366	cg24030449	FGF20	0.19	8.14E-08
1367	cg14605021	FGF20	0.11	1.03E-03
1368	cg17264470	FGF21	-0.18	3.15E-08
1369	cg22189019	FGF22	0.11	2.86E-05
1370	cg18384168	FGF22	0.11	9.52E-05
1371	cg23219570	FGF23	-0.11	1.07E-07
1372	cg21902327	FGF6	-0.27	5.36E-19
1373	cg01731341	FGF6	-0.30	6.77E-18
1374	cg23504246	FGF7	-0.13	6.44E-08
1375	cg16584573	FGF8	0.13	1.35E-04
1376	cg13929970	FGFBP1	-0.15	1.33E-10
1377	cg08722122	FGFR1	-0.15	1.65E-06
1378	cg01871995	FGL1	-0.12	5.68E-09
1379	cg12271671	FGL2	0.15	1.76E-09
1380	cg27223827	FHL2	-0.11	8.47E-05
1381	cg10635061	FHL2	-0.14	1.91E-08
1382	cg23978322	FHL5	-0.15	3.72E-10
1383	cg27062617	FHOD1	0.19	2.43E-06
1384	cg23065097	FKBP1B	0.15	8.24E-09
1385	cg13401339	FKBP1B	0.15	2.09E-04
1386	cg19145398	FKHL18	-0.10	2.38E-04
1387	cg08331840	FKSG2	-0.12	3.23E-08
1388	cg26390526	FLG	-0.24	1.39E-13
1389	cg13447818	FLG	-0.26	2.33E-12
1390	cg03602500	FLJ00060	-0.30	1.26E-25
1391	cg25033144	FLJ00060	-0.38	1.40E-24
1392	cg19566405	FLJ10260	0.14	2.68E-08
1393	cg11644586	FLJ10786	-0.11	2.64E-03
1394	cg04301614	FLJ11017	0.13	6.14E-08
1395	cg19843036	FLJ11155	0.17	8.00E-06

1396	cg01485998	FLJ12505	0.31	2.21E-13
1397	cg03455458	FLJ12505	0.27	1.68E-10
1398	cg27383956	FLJ13265	-0.12	3.12E-06
1399	cg13391638	FLJ13576	0.19	4.86E-08
1400	cg19786920	FLJ14001	0.27	6.95E-11
1401	cg17178888	FLJ14166	0.13	5.16E-05
1402	cg11808874	FLJ14668	-0.19	1.96E-10
1403	cg07412254	FLJ14816	-0.12	3.60E-06
1404	cg17204557	FLJ14816	-0.15	1.61E-06
1405	cg08924430	FLJ20032	0.18	3.71E-06
1406	cg07360692	FLJ20032	0.13	8.90E-06
1407	cg07717632	FLJ20245	0.34	8.10E-19
1408	cg03005261	FLJ20647	0.26	8.65E-11
1409	cg02955988	FLJ21106	-0.11	2.94E-12
1410	cg03616357	FLJ21159	0.40	1.78E-18
1411	cg25484904	FLJ21511	0.28	1.13E-15
1412	cg26538442	FLJ21736	-0.10	3.81E-04
1413	cg19229991	FLJ22655	-0.13	3.65E-09
1414	cg10971790	FLJ22688	0.17	1.00E-07
1415	cg14958018	FLJ25169	-0.18	6.68E-12
1416	cg15903395	FLJ25369	-0.10	1.44E-06
1417	cg08245789	FLJ25421	-0.20	8.38E-13
1418	cg22029275	FLJ25477	0.21	6.22E-10
1419	cg24169915	FLJ25773	-0.13	1.32E-20
1420	cg06906435	FLJ25773	-0.23	6.09E-23
1421	cg25569462	FLJ25801	-0.10	3.23E-05
1422	cg15648315	FLJ26443	-0.14	6.60E-06
1423	cg26815414	FLJ26443	-0.18	7.24E-09
1424	cg13126790	FLJ27255	-0.11	1.50E-06
1425	cg04329382	FLJ27365	0.20	3.94E-09
1426	cg19298821	FLJ27505	-0.15	1.15E-09
1427	cg11682508	FLJ30313	-0.23	2.80E-13
1428	cg05396987	FLJ30834	0.16	3.70E-08
1429	cg19018097	FLJ30934	0.23	2.62E-10
1430	cg04435377	FLJ31568	-0.17	1.03E-08
1431	cg00393585	FLJ31659	0.17	1.62E-09
1432	cg19987219	FLJ32011	-0.11	3.25E-07
1433	cg02245378	FLJ32447	0.18	3.10E-08
1434	cg14159672	FLJ32569	0.22	4.60E-09
1435	cg14893161	FLJ32569	0.15	3.47E-06
1436	cg00433406	FLJ32784	-0.20	7.21E-10
1437	cg03113824	FLJ32926	-0.19	3.22E-12
1438	cg18854045	FLJ33534	-0.24	1.54E-13
1439	cg12491659	FLJ33641	-0.15	5.39E-11
1440	cg26331247	FLJ33706	-0.15	1.93E-12
1441	cg18201198	FLJ33708	-0.26	4.94E-18
1442	cg12001148	FLJ33790	0.17	2.05E-08
1443	cg16547529	FLJ33790	0.12	2.41E-07
1444	cg14236389	FLJ33860	-0.29	3.60E-24
1445	cg09076077	FLJ33860	-0.30	2.98E-22

1446	cg21039679	FLJ35695	-0.15	5.05E-11
1447	cg03044435	FLJ35816	-0.31	2.52E-18
1448	cg18604842	FLJ36046	-0.11	3.40E-10
1449	cg04086012	FLJ36180	-0.28	4.10E-20
1450	cg01430430	FLJ37078	-0.13	8.46E-08
1451	cg18289156	FLJ37357	0.15	1.51E-04
1452	cg21269934	FLJ37478	0.37	5.36E-23
1453	cg25875213	FLJ37549	0.38	4.87E-15
1454	cg01703884	FLJ37587	-0.10	4.66E-06
1455	cg26292028	FLJ37587	-0.10	1.03E-08
1456	cg24580001	FLJ37970	-0.17	9.85E-17
1457	cg25784308	FLJ38377	0.11	2.18E-04
1458	cg13297249	FLJ38379	-0.15	3.09E-17
1459	cg16173109	FLJ38379	-0.20	9.69E-15
1460	cg22158923	FLJ39237	-0.13	2.84E-07
1461	cg08349806	FLJ39599	0.13	5.12E-05
1462	cg03464655	FLJ39822	0.10	6.49E-03
1463	cg03213216	FLJ40125	0.30	4.58E-19
1464	cg00116838	FLJ40125	0.28	2.54E-16
1465	cg21930712	FLJ40235	-0.17	1.12E-15
1466	cg24469977	FLJ40288	-0.16	1.58E-09
1467	cg07409200	FLJ40919	-0.10	8.26E-09
1468	cg08782122	FLJ42291	-0.10	1.63E-17
1469	cg03734874	FLJ42486	0.18	2.82E-08
1470	cg00107187	FLJ42486	0.12	1.63E-04
1471	cg00297584	FLJ42957	0.15	7.37E-06
1472	cg08301503	FLJ43752	0.14	2.39E-05
1473	cg04329454	FLJ43752	0.10	4.26E-03
1474	cg21045388	FLJ43806	-0.19	1.50E-17
1475	cg17657618	FLJ43806	-0.22	5.35E-18
1476	cg23580945	FLJ43826	-0.18	4.47E-13
1477	cg18777554	FLJ44112	-0.18	1.66E-11
1478	cg25032865	FLJ44186	-0.12	5.07E-12
1479	cg17398003	FLJ44216	0.10	1.62E-05
1480	cg24217877	FLJ44674	-0.21	5.19E-19
1481	cg13897627	FLJ44674	-0.30	7.22E-27
1482	cg21306775	FLJ44881	-0.12	1.34E-08
1483	cg05364569	FLJ45202	-0.17	8.42E-17
1484	cg26776077	FLJ45202	-0.26	7.39E-20
1485	cg18354594	FLJ45717	-0.13	1.62E-05
1486	cg15207953	FLJ45832	-0.14	2.62E-14
1487	cg22954265	FLJ45832	-0.16	1.16E-16
1488	cg10832945	FLJ45964	-0.12	1.05E-08
1489	cg17977362	FLJ45964	-0.24	3.06E-09
1490	cg04765277	FLJ45983	0.14	2.33E-05
1491	cg19282452	FLJ46230	-0.20	1.32E-14
1492	cg08579995	FLJ46230	-0.27	5.24E-17
1493	cg12682367	FLJ46358	-0.23	9.33E-22
1494	cg07440877	FLJ46358	-0.23	1.33E-11
1495	cg25538571	FLJ46365	-0.20	1.12E-08

1496	cg03167883	FLJ46365	-0.20	1.35E-15
1497	cg25093045	FLJ46481	-0.22	1.99E-22
1498	cg00958560	FLJ46481	-0.30	4.36E-23
1499	cg03782453	FLJ90575	0.30	4.69E-27
1500	cg09111484	FLJ90579	-0.12	3.56E-07
1501	cg22367264	FLJ90579	-0.13	1.23E-08
1502	cg25044651	FLJ90650	0.30	8.23E-12
1503	cg15489294	FLJ90650	0.16	3.71E-05
1504	cg02661879	FLNC	0.25	1.11E-08
1505	cg17410236	FLRT2	0.14	1.79E-04
1506	cg07017374	FLT3	0.25	8.33E-10
1507	cg00489401	FLT4	0.35	8.43E-14
1508	cg04945541	FMNL1	0.26	2.66E-17
1509	cg18063149	FMO3	-0.11	6.52E-06
1510	cg25778166	FMO3	-0.12	2.74E-06
1511	cg04587829	FN3K	-0.12	1.32E-04
1512	cg26750747	FOLR1	-0.11	1.42E-10
1513	cg18818531	FOSL1	0.13	4.50E-06
1514	cg08583049	FOXB1	0.35	2.16E-18
1515	cg22777952	FOXB1	0.21	2.70E-08
1516	cg04504095	FOXC1	0.20	6.58E-07
1517	cg23867624	FOXC1	0.11	4.13E-03
1518	cg13958614	FOXD1	0.28	1.74E-16
1519	cg03663715	FOXD1	0.25	3.98E-10
1520	cg15868302	FOXD2	0.14	8.55E-17
1521	cg22815110	FOXD3	0.38	6.14E-18
1522	cg16708281	FOXD4L1	0.19	3.08E-16
1523	cg07485588	FOXD4L4	0.13	4.42E-13
1524	cg19292008	FOXE1	0.18	5.89E-07
1525	cg18815943	FOXE3	0.53	1.82E-22
1526	cg25971347	FOXF1	0.15	4.02E-05
1527	cg08045570	FOXF2	0.13	5.18E-04
1528	cg10300684	FOXG1B	0.21	1.01E-09
1529	cg02681442	FOXG1B	0.16	6.10E-08
1530	cg19233472	FOXI1	-0.17	1.97E-07
1531	cg00661485	FOXI1	-0.36	6.53E-20
1532	cg14312526	FOXL2	0.36	2.91E-14
1533	cg17503456	FOXL2	0.32	1.00E-12
1534	cg10213812	FOXN1	-0.14	3.89E-12
1535	cg05755354	FRMD4A	-0.21	5.05E-12
1536	cg25464840	FRMD4A	-0.23	3.86E-15
1537	cg16046465	FRMD5	0.32	4.35E-12
1538	cg06206628	FRMPD2	-0.14	1.17E-08
1539	cg20308679	FRZB	0.26	5.82E-12
1540	cg19568591	FRZB	0.15	9.89E-06
1541	cg25176823	FSCN1	0.34	8.09E-21
1542	cg25902889	FSD1	0.18	3.43E-17
1543	cg27420123	FSHB	-0.19	3.43E-24
1544	cg17327492	FSHR	-0.28	1.16E-22
1545	cg04963951	FTMT	-0.19	1.08E-13

1546	cg11234457	FTMT	-0.24	3.20E-17
1547	cg00796728	FUT4	0.16	4.07E-06
1548	cg12631737	FUT4	0.11	2.19E-04
1549	cg00579402	FUT6	0.10	9.98E-04
1550	cg10225525	FUT8	-0.15	3.47E-09
1551	cg25894551	FXYD2	-0.14	8.86E-06
1552	cg25081201	FXYD4	-0.10	4.34E-10
1553	cg12619162	FXYD4	-0.23	1.30E-14
1554	cg17682828	FXYD7	0.14	2.53E-05
1555	cg17100322	FYN	0.23	6.52E-07
1556	cg25228126	FZD2	0.18	5.58E-09
1557	cg21975377	FZD6	0.11	9.75E-04
1558	cg12618251	FZD7	0.15	4.84E-05
1559	cg20692569	FZD9	0.28	1.24E-24
1560	cg18438300	FZD9	-0.15	7.79E-13
1561	cg07903918	GABBR2	0.11	9.52E-04
1562	cg24387380	GABRA5	-0.14	2.66E-11
1563	cg07592353	GABRA6	-0.15	8.43E-14
1564	cg24244000	GABRG3	-0.12	2.04E-07
1565	cg27592112	GABRG3	-0.21	2.53E-13
1566	cg23181170	GABRP	-0.13	4.88E-10
1567	cg21652012	GABRP	-0.18	1.95E-07
1568	cg18047970	GADL1	-0.20	2.59E-09
1569	cg15946807	GADL1	-0.22	5.81E-11
1570	cg21238818	GAL3ST3	0.27	6.57E-16
1571	cg01725199	GALNT12	0.38	5.59E-14
1572	cg05593325	GALNT12	0.20	2.53E-09
1573	cg05714219	GALNT14	0.32	4.22E-13
1574	cg21549904	GALNT7	0.19	1.11E-05
1575	cg13255190	GALNT8	-0.12	3.52E-14
1576	cg14920334	GALNT8	-0.15	2.55E-09
1577	cg11091262	GALNTL5	-0.13	5.89E-09
1578	cg00662556	GALR1	0.14	2.50E-04
1579	cg04534765	GALR1	0.13	1.78E-03
1580	cg02293044	GAS2L1	-0.10	5.25E-05
1581	cg26420196	GAS6	0.13	5.00E-04
1582	cg22471346	GAS7	0.36	5.05E-13
1583	cg06490988	GATA2	0.17	6.93E-06
1584	cg06230736	GATA3	0.29	1.18E-09
1585	cg19172575	GATA4	0.12	2.00E-08
1586	cg01546563	GATA4	0.11	6.18E-05
1587	cg09923671	GATA5	-0.14	1.58E-10
1588	cg16542081	GATA5	-0.15	1.12E-12
1589	cg14519000	GATA5	-0.17	7.45E-13
1590	cg04132607	GATA5	-0.19	3.74E-18
1591	cg20822628	GATA5	-0.19	2.22E-10
1592	cg09864990	GATA5	-0.23	1.05E-19
1593	cg17740305	GBP5	-0.12	2.12E-24
1594	cg06954481	GBX2	0.29	2.01E-09
1595	cg23095584	GBX2	0.24	6.12E-13

1596	cg25462303	GCET2	-0.16	4.98E-18
1597	cg15001372	GCK	-0.13	4.70E-10
1598	cg02844545	GCM2	0.16	6.31E-07
1599	cg22289360	GCNT1	0.29	1.72E-12
1600	cg09885086	GCNT1	0.23	1.51E-09
1601	cg23877385	GCNT3	-0.12	3.82E-06
1602	cg00509616	Gcom1	0.30	4.66E-12
1603	cg04448487	GDAP1L1	0.37	1.61E-18
1604	cg17810944	GDAP1L1	0.11	5.56E-04
1605	cg26090660	GDEP	-0.12	4.77E-16
1606	cg02255004	GDEP	-0.18	1.28E-10
1607	cg07773116	GDF10	0.11	5.69E-03
1608	cg21115977	GDF2	-0.18	8.85E-10
1609	cg07378350	GDF5	-0.17	3.24E-10
1610	cg07715201	GDNF	0.10	6.07E-04
1611	cg03297731	GDPD3	-0.18	5.80E-09
1612	cg11452221	GEFT	0.12	4.58E-05
1613	cg02364642	GEFT	0.12	5.53E-06
1614	cg09038914	GFAP	-0.13	1.07E-07
1615	cg20125091	GFI1	0.28	9.79E-13
1616	cg22341104	GFI1	0.22	8.67E-07
1617	cg00194146	GFOD1	0.25	1.92E-08
1618	cg18602314	GFPT2	0.21	7.62E-09
1619	cg21937886	GFPT2	0.18	1.85E-06
1620	cg09350274	GFRA3	0.28	2.28E-14
1621	cg02990033	GFRA4	-0.15	5.19E-10
1622	cg13424446	GGTLA4	-0.14	1.41E-19
1623	cg19882093	GGTLA4	-0.14	6.84E-13
1624	cg13204181	GH1	-0.23	6.66E-13
1625	cg05131835	GH2	-0.19	6.62E-14
1626	cg04459030	GHRH	-0.22	1.38E-15
1627	cg11812218	GHSR	0.13	5.78E-06
1628	cg18538812	GIF	-0.20	7.78E-10
1629	cg26970800	GIF	-0.28	6.25E-13
1630	cg13043509	GIMAP5	0.17	6.39E-09
1631	cg01827098	GIMAP7	-0.15	1.73E-06
1632	cg20551517	GIP	-0.15	1.31E-06
1633	cg12428416	GJA3	-0.21	9.55E-15
1634	cg07070934	GJA4	0.12	1.91E-04
1635	cg11054936	GJB2	-0.12	2.51E-13
1636	cg15554401	GJB4	-0.17	1.10E-10
1637	cg15750705	GJB4	-0.22	8.46E-16
1638	cg01333788	GJB5	-0.14	2.92E-07
1639	cg08612871	GJB5	-0.15	1.96E-10
1640	cg22377389	GJB6	0.25	2.31E-09
1641	cg03473518	GJB6	0.18	5.49E-07
1642	cg17738194	GK2	-0.22	8.05E-25
1643	cg14078518	GK2	-0.25	1.17E-17
1644	cg13877895	GKN1	-0.10	1.27E-04
1645	cg06941093	GLIS1	-0.11	2.60E-08

1646	cg26781150	GLOXD1	0.26	1.41E-09
1647	cg10164640	GLOXD1	0.21	1.25E-07
1648	cg25014318	GLP1R	0.11	4.64E-04
1649	cg01056568	GLP2R	-0.10	4.25E-07
1650	cg20261915	GLP2R	-0.17	5.71E-10
1651	cg15522719	GLS	0.10	1.42E-03
1652	cg14313310	GLT25D2	0.18	2.33E-07
1653	cg16791686	GLTSCR2	-0.21	6.44E-18
1654	cg02436686	GMFG	-0.13	2.99E-08
1655	cg00057593	GML	-0.17	2.85E-12
1656	cg07711097	GML	-0.31	2.60E-25
1657	cg14917512	GNA11	0.37	4.97E-23
1658	cg17301902	GNA14	0.32	6.50E-17
1659	cg14371329	GNAL	-0.18	4.00E-11
1660	cg25983380	GNAS	0.22	4.93E-20
1661	cg00943909	GNAS	-0.11	1.43E-05
1662	cg01565918	GNAS	-0.11	8.72E-08
1663	cg05558390	GNAS	-0.11	2.99E-04
1664	cg17414107	GNAS	-0.11	5.91E-06
1665	cg20582984	GNAS	-0.12	9.47E-07
1666	cg14203179	GNAS	-0.12	2.89E-05
1667	cg14597908	GNAS	-0.12	7.68E-06
1668	cg03606258	GNAS	-0.15	1.27E-07
1669	cg06324048	GNAS	-0.15	4.86E-12
1670	cg25268451	GNAS	-0.16	8.69E-08
1671	cg16833551	GNAS	-0.19	4.49E-10
1672	cg07284407	GNAS	-0.23	6.31E-14
1673	cg21988465	GNAS	-0.26	2.60E-16
1674	cg17483510	GNB4	0.37	2.69E-23
1675	cg09997760	GNB4	0.19	1.04E-06
1676	cg26764244	GNG12	0.10	9.66E-05
1677	cg09649610	GNG4	0.20	4.79E-08
1678	cg17839611	GNGT2	-0.27	2.36E-13
1679	cg24751129	GNMT	-0.20	2.42E-10
1680	cg04267184	GNRH2	0.29	4.87E-23
1681	cg27219973	GNRHR	-0.14	1.03E-10
1682	cg01714513	GOLGA8A	-0.11	2.15E-10
1683	cg24852661	GOLPH2	0.13	1.18E-04
1684	cg24798047	GOLT1A	-0.12	1.30E-10
1685	cg07359545	GP1BB	-0.12	8.07E-04
1686	cg24987706	GPA33	-0.13	5.38E-07
1687	cg06665322	GPA33	-0.13	5.36E-07
1688	cg12137206	GPAM	0.20	6.77E-08
1689	cg24965984	GPAM	0.16	1.59E-05
1690	cg18691434	GPC2	0.40	5.48E-23
1691	cg05662500	GPD1L	0.14	3.32E-04
1692	cg14023451	GPLD1	-0.16	6.66E-11
1693	cg22932819	GPNMB	-0.10	1.12E-05
1694	cg17274742	GPNMB	-0.14	1.26E-08
1695	cg19132372	GPR1	-0.23	1.87E-14

1696	cg12014417	GPR109A	-0.18	2.23E-19
1697	cg03311899	GPR109A	-0.25	1.17E-20
1698	cg15447486	GPR109B	-0.15	2.64E-12
1699	cg05384917	GPR109B	-0.25	6.54E-14
1700	cg09069593	GPR114	-0.22	3.81E-17
1701	cg23613030	GPR115	-0.16	1.24E-10
1702	cg18841952	GPR115	-0.21	6.03E-20
1703	cg14045072	GPR116	-0.17	4.35E-07
1704	cg15726245	GPR12	-0.18	2.03E-11
1705	cg21607649	GPR123	-0.11	4.70E-10
1706	cg20011352	GPR124	0.19	1.04E-07
1707	cg26514492	GPR132	-0.23	4.89E-11
1708	cg24995381	GPR141	-0.34	1.92E-19
1709	cg22377428	GPR142	-0.16	2.17E-09
1710	cg03803009	GPR142	-0.29	1.21E-21
1711	cg04037952	GPR148	-0.23	4.88E-12
1712	cg04597449	GPR150	0.23	4.72E-12
1713	cg02320454	GPR150	0.16	2.52E-06
1714	cg18741908	GPR160	0.28	1.79E-20
1715	cg26575445	GPR160	0.18	9.44E-08
1716	cg17805404	GPR162	0.14	1.15E-07
1717	cg21870884	GPR25	0.29	1.56E-15
1718	cg04983977	GPR25	0.12	1.98E-20
1719	cg13562542	GPR27	0.38	1.41E-13
1720	cg22631938	GPR27	0.23	2.76E-12
1721	cg12970081	GPR32	-0.22	5.17E-16
1722	cg07432969	GPR35	-0.20	5.94E-18
1723	cg13097816	GPR35	-0.24	2.77E-12
1724	cg26026726	GPR42	-0.18	9.30E-10
1725	cg23661676	GPR42	-0.23	8.62E-14
1726	cg22253945	GPR45	-0.22	3.66E-14
1727	cg21399079	GPR45	-0.22	4.41E-11
1728	cg26252167	GPR6	0.21	1.66E-07
1729	cg12800028	GPR6	0.11	9.25E-04
1730	cg10189695	GPR78	0.21	8.36E-08
1731	cg27634151	GPR83	0.24	1.02E-12
1732	cg21969640	GPR84	-0.22	2.42E-12
1733	cg10298815	GPR88	0.12	5.65E-04
1734	cg14934821	GPSM1	-0.10	6.05E-06
1735	cg13237829	GPSN2	0.14	2.24E-09
1736	cg15983520	GPT	-0.12	2.95E-08
1737	cg06613840	GPX1	-0.12	7.34E-08
1738	cg18849169	GPX3	-0.18	7.03E-14
1739	cg16557944	GPX7	0.28	3.82E-08
1740	cg22129364	GPX7	0.22	2.71E-08
1741	cg15165084	GRAMD3	0.19	6.56E-04
1742	cg08704509	GRAMD3	0.14	9.36E-05
1743	cg25712380	GRAP2	-0.16	7.86E-11
1744	cg04034767	GRASP	0.45	4.36E-23
1745	cg22566906	GRASP	-0.11	9.50E-05

1746	cg17740645	GRB7	-0.12	1.54E-05
1747	cg03684977	GRB7	-0.15	1.48E-06
1748	cg18145505	GREM1	0.29	7.09E-13
1749	cg21296230	GREM1	0.19	1.41E-06
1750	cg16155382	GRHL3	0.22	7.80E-10
1751	cg15050111	GRHL3	0.22	7.45E-10
1752	cg15425280	GRIA2	0.13	2.59E-05
1753	cg09555879	GRIK5	-0.32	9.25E-22
1754	cg09414535	GRIP1	-0.15	5.60E-11
1755	cg24694549	GRIP1	-0.27	2.85E-15
1756	cg21710060	GRK1	-0.11	2.09E-08
1757	cg09034896	GRK1	-0.14	1.27E-05
1758	cg15550350	GRK7	-0.17	1.62E-13
1759	cg26424956	GRM4	-0.27	6.09E-17
1760	cg01962826	GRM4	-0.40	4.71E-24
1761	cg14859460	GRM6	0.13	1.88E-03
1762	cg02946850	GRM8	-0.26	4.88E-16
1763	cg19224837	GSC	0.10	4.03E-04
1764	cg26599006	GSCL	0.32	1.66E-12
1765	cg26609631	GSH1	0.27	1.01E-08
1766	cg03070194	GSTM2	0.39	1.81E-24
1767	cg16670497	GSTM2	0.28	3.43E-17
1768	cg04987894	GSTM5	0.16	3.97E-08
1769	cg25250998	GSTO2	0.17	2.19E-04
1770	cg15410903	GSTO2	0.11	2.94E-02
1771	cg02659086	GSTP1	0.39	7.81E-18
1772	cg04920951	GSTP1	0.39	7.19E-21
1773	cg09038676	GSTP1	0.29	5.39E-13
1774	cg11566244	GSTP1	0.11	3.36E-05
1775	cg06003187	GUCA2A	-0.20	1.10E-10
1776	cg23984434	GUCY1A2	0.20	2.25E-06
1777	cg02210887	GUCY1A3	-0.11	4.87E-08
1778	cg13274254	GULP1	0.20	1.09E-08
1779	cg24938727	GUP1	0.17	4.14E-07
1780	cg00407150	GUP1	-0.13	1.05E-10
1781	cg09841009	GYPA	-0.30	1.65E-21
1782	cg16636571	GYPB	-0.11	4.77E-12
1783	cg27214365	GYPB	-0.31	7.22E-23
1784	cg13143729	GYPE	-0.12	8.54E-15
1785	cg16998872	GYPE	-0.30	2.65E-22
1786	cg06118312	GZMA	-0.16	4.65E-14
1787	cg08766149	GZMB	-0.38	6.62E-22
1788	cg22228134	GZMH	-0.21	7.47E-16
1789	cg04705866	GZMK	-0.11	2.13E-08
1790	cg18338021	GZMM	-0.14	6.52E-09
1791	cg02657360	H19	-0.11	8.89E-09
1792	cg23977670	H19	-0.12	7.97E-10
1793	cg10602543	H19	-0.14	2.27E-07
1794	cg25852472	H19	-0.15	1.91E-07
1795	cg17769238	H19	-0.17	1.94E-09

1796	cg11492040	H19	-0.18	3.05E-09
1797	cg26808784	H19	-0.27	2.49E-13
1798	cg00745543	H1T2	-0.24	1.95E-15
1799	cg00601486	H1T2	-0.38	1.42E-24
1800	cg24628744	H2AFY	0.13	3.57E-13
1801	cg01550148	H2AFY	0.11	4.94E-14
1802	cg15783800	HAK	-0.14	1.57E-06
1803	cg19721889	HAND1	0.12	8.59E-05
1804	cg01580681	HAND2	0.19	8.67E-07
1805	cg06434428	HAPLN1	-0.18	1.55E-09
1806	cg14449575	HAPLN4	0.15	3.34E-15
1807	cg13300756	HAS1	-0.32	8.19E-21
1808	cg14141399	HAS1	-0.39	1.07E-20
1809	cg17484237	HAVCR2	-0.18	1.77E-20
1810	cg20609368	HBD	-0.24	5.17E-21
1811	cg08970694	HBE1	-0.19	6.57E-26
1812	cg01598642	HBG1	-0.29	9.91E-23
1813	cg09839960	HBII-438B	-0.20	1.42E-16
1814	cg07703401	HBQ1	0.43	5.92E-22
1815	cg21960110	HBZ	-0.14	9.27E-07
1816	cg04623837	HCG9	0.34	9.32E-17
1817	cg23244913	HCG9	0.31	2.53E-17
1818	cg00141162	HCLS1	0.20	1.14E-10
1819	cg06005396	HCN2	0.17	3.02E-10
1820	cg02860342	HCN4	0.17	1.49E-08
1821	cg17063201	HCRTR2	-0.23	4.55E-15
1822	cg24385322	HDAC3	0.26	4.19E-10
1823	cg10414058	HDAC3	0.25	1.55E-14
1824	cg00746981	HDGF	0.25	6.55E-17
1825	cg21070087	HECW1	-0.18	9.84E-12
1826	cg17628717	HECW1	-0.25	4.35E-17
1827	cg17605084	HEM1	-0.11	1.25E-05
1828	cg27592318	HEMGN	-0.18	1.11E-09
1829	cg02397514	HES4	0.24	4.08E-07
1830	cg24127874	HES6	0.20	6.85E-08
1831	cg04700814	HEXIM1	0.13	3.45E-08
1832	cg12866859	HEXIM1	0.11	7.69E-06
1833	cg25462291	HEYL	0.25	2.91E-09
1834	cg06589885	HFE2	-0.16	3.59E-11
1835	cg08005849	HGF	-0.10	2.13E-06
1836	cg07364841	HGFAC	0.19	2.82E-15
1837	cg02879662	HIF3A	0.13	1.90E-05
1838	cg07123548	HIPK4	-0.15	3.05E-16
1839	cg14652095	HIST1H1A	0.13	4.44E-13
1840	cg17711541	HIST1H2AC	0.11	1.39E-06
1841	cg20520725	HIST1H2AE	0.35	1.27E-23
1842	cg24358465	HIST1H2AG	0.17	2.50E-04
1843	cg10046620	HIST1H2AI	0.31	1.41E-13
1844	cg03221914	HIST1H2AJ	0.19	1.43E-16
1845	cg17050972	HIST1H2AJ	0.18	1.71E-07

1846	cg02367951	HIST1H2AK	0.12	2.41E-12
1847	cg21146268	HIST1H2BE	0.12	7.37E-06
1848	cg21663122	HIST1H2BH	0.35	6.44E-21
1849	cg14776962	HIST1H2BH	0.22	1.55E-14
1850	cg02192855	HIST1H2BI	0.25	2.20E-11
1851	cg27059238	HIST1H2BI	0.11	1.89E-10
1852	cg08797106	HIST1H3A	0.16	2.81E-16
1853	cg24784109	HIST1H3D	0.11	2.61E-09
1854	cg05414338	HIST1H3F	0.27	8.92E-12
1855	cg06254453	HIST1H3F	0.23	6.29E-11
1856	cg02909790	HIST1H3G	0.39	1.05E-22
1857	cg17718302	HIST1H3J	0.48	1.69E-24
1858	cg17965019	HIST1H3J	0.39	2.11E-18
1859	cg08260959	HIST1H4F	0.38	5.52E-22
1860	cg05159188	HIST1H4F	0.22	4.57E-09
1861	cg17651821	HIST1H4L	0.15	6.60E-04
1862	cg14415300	HIST2H2AA	0.28	3.05E-18
1863	cg14743462	HIST3H2A	0.28	8.69E-13
1864	cg00143998	HIST3H2BB	0.16	4.28E-08
1865	cg14541950	HIST3H2BB	0.14	1.23E-06
1866	cg06323290	HK1	-0.11	3.91E-05
1867	cg24107142	HK2	-0.25	1.18E-19
1868	cg18638581	HK2	-0.26	1.30E-17
1869	cg14709481	HK3	-0.17	3.30E-13
1870	cg14166009	HKR1	0.22	9.69E-12
1871	cg12024906	HKR1	0.20	2.92E-05
1872	cg20879959	HLA-A	0.13	1.82E-05
1873	cg16097079	HLA-C	0.11	3.71E-05
1874	cg16899306	HLA-DQB2	-0.19	2.76E-14
1875	cg04345908	HLA-DQB2	-0.20	2.22E-17
1876	cg25764570	HLA-DRA	-0.17	5.49E-11
1877	cg10746737	HLA-DRB5	-0.17	4.50E-09
1878	cg17475918	HLA-E	0.15	1.57E-04
1879	cg21529533	HLA-G	0.21	8.18E-09
1880	cg04219321	HLF	0.18	2.08E-05
1881	cg20899053	HLXB9	0.26	4.60E-09
1882	cg03020951	HMGA1	0.35	7.78E-21
1883	cg10212621	HMGCS2	-0.11	9.27E-08
1884	cg22718139	HMGCS2	-0.12	5.04E-05
1885	cg13176979	HMGN3	0.12	3.92E-05
1886	cg23834593	HNF4A	-0.14	2.57E-10
1887	cg22062068	HNRNPG-T	-0.13	5.89E-06
1888	cg00363813	HOM-TES-103	-0.20	1.44E-09
1889	cg03700462	HOXA1	0.13	1.03E-06
1890	cg15760840	HOXA11	0.16	2.03E-07
1891	cg17950095	HOXA11	0.12	6.91E-04
1892	cg06397837	HOXA13	0.28	3.80E-14
1893	cg10883303	HOXA13	0.27	6.32E-07
1894	cg26069745	HOXA2	0.22	1.63E-08
1895	cg09871315	HOXA2	0.17	3.49E-06

1896	cg25426743	HOXA3	0.11	5.07E-08
1897	cg24169822	HOXA4	0.19	1.32E-08
1898	cg04317399	HOXA4	0.13	1.44E-04
1899	cg02248486	HOXA5	0.12	3.27E-03
1900	cg04265576	HOXA6	0.21	8.81E-07
1901	cg23432345	HOXA7	0.31	2.27E-12
1902	cg26511321	HOXA7	0.22	1.73E-10
1903	cg26521404	HOXA9	0.38	1.31E-15
1904	cg01354473	HOXA9	0.33	2.51E-16
1905	cg07778029	HOXA9	0.32	1.35E-14
1906	cg27009703	HOXA9	0.26	1.09E-12
1907	cg01381846	HOXA9	0.26	9.82E-15
1908	cg25047280	HOXA9	0.23	2.70E-10
1909	cg07823492	HOXB1	-0.12	3.63E-04
1910	cg25882366	HOXB2	0.13	8.27E-05
1911	cg09313705	HOXB2	0.11	3.35E-12
1912	cg21460081	HOXB4	0.22	6.78E-15
1913	cg08089301	HOXB4	0.13	8.41E-05
1914	cg15539420	HOXB8	0.11	2.14E-03
1915	cg26153631	HOXC10	0.13	1.43E-06
1916	cg07123069	HOXC11	0.13	1.74E-05
1917	cg15700739	HOXC5	0.14	3.51E-08
1918	cg19001226	HOXD1	0.12	3.99E-04
1919	cg16632715	HOXD11	0.23	2.31E-09
1920	cg19325985	HOXD13	0.15	1.63E-07
1921	cg00005847	HOXD3	0.13	1.13E-05
1922	cg00767581	HOXD4	0.11	1.00E-03
1923	cg12127282	HOXD4	0.11	1.61E-03
1924	cg15520279	HOXD8	0.44	6.56E-20
1925	cg10957151	HOXD9	0.11	1.23E-04
1926	cg24417499	HPCA	0.19	2.99E-08
1927	cg11147193	HPCAL1	-0.12	3.47E-09
1928	cg00979348	HPSE	0.10	6.15E-06
1929	cg27016307	HRC	-0.12	7.49E-08
1930	cg14837082	HRH1	-0.11	2.47E-12
1931	cg14345676	HRH2	-0.14	6.33E-11
1932	cg20277670	HRH2	-0.32	2.54E-21
1933	cg08186362	HRH3	0.25	3.02E-08
1934	cg10605520	HRH3	0.12	1.57E-04
1935	cg20373326	HSD17B2	-0.10	1.29E-06
1936	cg26359240	HSDL1	-0.14	6.84E-13
1937	cg15127733	HSPA12B	0.11	1.31E-05
1938	cg09458237	HSPA12B	-0.23	4.07E-16
1939	cg27120999	HSPA2	0.38	1.27E-24
1940	cg24642523	HSPA2	0.34	2.82E-18
1941	cg01520924	HSPA2	0.14	1.56E-03
1942	cg16319578	HSPA2	0.13	3.28E-04
1943	cg13817266	HSPA6	0.13	6.93E-08
1944	cg22189286	HSPB8	-0.20	1.14E-10
1945	cg05346140	HSPB9	-0.12	6.45E-04

1946	cg02968557	HSPB9	-0.14	5.72E-05
1947	cg12582959	HSU79303	0.10	2.12E-03
1948	cg15846641	HTLF	0.14	7.52E-05
1949	cg06545504	HTN1	-0.11	6.78E-06
1950	cg20887241	HTR1D	-0.10	4.90E-09
1951	cg12775613	HTR1F	-0.15	2.76E-09
1952	cg06096336	HTR2B	0.10	1.81E-04
1953	cg06531741	HTR3B	-0.14	1.98E-06
1954	cg18271969	HTR3C	-0.18	1.03E-10
1955	cg19815720	HTR3C	-0.19	1.58E-11
1956	cg14483391	HTR3D	-0.11	1.05E-05
1957	cg04200192	HTR3D	-0.25	6.21E-16
1958	cg26153642	HTR3E	-0.14	1.44E-07
1959	cg06291867	HTR7	0.42	2.14E-24
1960	cg09495977	HTRA3	0.13	1.29E-04
1961	cg14757296	HYAL1	-0.12	2.16E-24
1962	cg00840516	HYAL2	0.16	1.65E-13
1963	cg15560337	HYAL4	-0.14	4.25E-07
1964	cg09794131	HYDIN	0.17	1.54E-07
1965	cg20977864	HYDIN	0.14	1.52E-08
1966	cg24125648	HYPK	0.14	1.27E-05
1967	cg05434957	ICA1	0.10	2.95E-04
1968	cg07918509	ICAM2	-0.11	1.47E-04
1969	cg06855803	ICAM3	0.11	1.57E-06
1970	cg14145194	ICAM3	0.10	2.96E-06
1971	cg00468146	ID4	0.20	1.89E-07
1972	cg14967066	IFITM1	0.17	1.03E-05
1973	cg11959435	IFNA1	-0.18	3.08E-17
1974	cg01906717	IFNA13	-0.10	1.14E-09
1975	cg21023114	IFNA14	-0.16	1.09E-15
1976	cg01074640	IFNA17	-0.29	3.66E-22
1977	cg07373172	IFNA21	-0.15	8.29E-12
1978	cg19982860	IFNA21	-0.30	2.20E-24
1979	cg23029519	IFNA4	-0.11	4.96E-11
1980	cg15669228	IFNA8	-0.18	1.17E-19
1981	cg05126264	IFNB1	-0.25	4.11E-25
1982	cg26227465	IFNG	-0.13	2.16E-07
1983	cg11390468	IFT57	0.15	9.60E-06
1984	cg22375192	IGF1R	0.49	4.76E-25
1985	cg02807948	IGF2	-0.12	5.82E-10
1986	cg22956483	IGF2	-0.14	5.11E-06
1987	cg13756879	IGF2	-0.16	4.60E-05
1988	cg11005826	IGF2AS	-0.11	9.99E-05
1989	cg04112019	IGF2AS	-0.13	3.16E-05
1990	cg16817891	IGF2AS	-0.17	6.77E-10
1991	cg20792294	IGF2AS	-0.19	9.28E-07
1992	cg10501065	IGF2AS	-0.22	8.83E-11
1993	cg06638433	IGF2BP1	0.22	1.06E-08
1994	cg18234011	IGF2BP2	0.14	3.31E-04
1995	cg01747665	IGFALS	0.13	4.03E-08

1996	cg05982504	IGFALS	-0.13	9.31E-06
1997	cg22467567	IGFBP5	0.10	1.46E-05
1998	cg03876618	IGFBP7	0.23	1.08E-10
1999	cg02415431	IGLL1	-0.12	2.23E-06
2000	cg10494770	IGLL1	-0.12	1.87E-10
2001	cg14458615	IGSF11	-0.12	2.63E-06
2002	cg20530056	IKBKE	0.10	1.67E-04
2003	cg17067005	IL10	-0.20	4.05E-11
2004	cg01697865	IL10RA	-0.21	1.09E-13
2005	cg26661481	IL10RA	-0.25	8.46E-14
2006	cg27416067	IL12RB2	0.29	8.36E-15
2007	cg09837803	IL16	-0.19	1.02E-11
2008	cg14366598	IL17E	-0.17	5.84E-20
2009	cg07258507	IL17E	-0.27	3.35E-21
2010	cg15095327	IL17RE	0.12	2.20E-04
2011	cg07832674	IL17RE	0.10	1.27E-06
2012	cg16749930	IL18BP	0.16	2.44E-09
2013	cg05916196	IL19	-0.12	1.15E-09
2014	cg00839584	IL1A	0.14	2.21E-14
2015	cg21378206	IL1F5	-0.16	9.51E-12
2016	cg02229946	IL1F7	-0.13	5.01E-13
2017	cg10479672	IL1F8	-0.19	1.58E-14
2018	cg08861115	IL1F9	-0.12	1.61E-06
2019	cg01103730	IL20	-0.12	7.81E-05
2020	cg04481779	IL20RA	0.30	3.53E-18
2021	cg22487322	IL20RA	0.11	6.83E-11
2022	cg02656594	IL21R	-0.11	6.54E-09
2023	cg26333641	IL22	-0.23	1.38E-15
2024	cg00294382	IL23A	0.20	1.43E-16
2025	cg06796611	IL24	-0.13	1.70E-10
2026	cg23909633	IL24	-0.15	1.12E-10
2027	cg17982671	IL28B	-0.16	9.10E-11
2028	cg11270633	IL28B	-0.18	1.43E-16
2029	cg27412902	IL29	-0.10	2.62E-07
2030	cg26589285	IL29	-0.24	8.32E-13
2031	cg17983064	IL3	-0.10	2.75E-07
2032	cg11212312	IL3	-0.20	1.73E-14
2033	cg10159529	IL5RA	-0.27	2.48E-19
2034	cg21004129	IL8RA	-0.12	1.02E-07
2035	cg13519373	IL8RA	-0.18	3.73E-18
2036	cg13739417	IL8RB	-0.18	2.64E-11
2037	cg14150666	IL8RB	-0.19	2.89E-14
2038	cg08463485	ILDR1	0.18	4.62E-08
2039	cg11726593	IMPA1	-0.10	2.47E-13
2040	cg24127989	IMPDH1	0.17	7.77E-06
2041	cg15740366	IMPDH1	0.16	4.36E-05
2042	cg26083396	IMPDH1	0.15	7.49E-04
2043	cg05089059	IMPDH1	0.12	2.29E-04
2044	cg25764191	INA	0.41	1.97E-20
2045	cg23642747	INA	0.35	7.08E-20

2046	cg25928444	INHA	-0.16	2.01E-10
2047	cg08640498	INHBC	-0.10	1.74E-06
2048	cg22630748	INHBE	-0.10	7.85E-05
2049	cg02309273	INPP5B	0.16	3.66E-06
2050	cg10784030	INPP5B	0.13	2.78E-06
2051	cg18285951	INPP5E	0.29	5.34E-09
2052	cg03366382	INS	-0.18	4.22E-13
2053	cg13993218	INS	-0.31	7.24E-21
2054	cg25336198	INS	-0.35	9.17E-21
2055	cg00613255	INS	-0.35	1.43E-18
2056	cg01413516	INSL3	-0.11	4.22E-04
2057	cg12067261	INSL4	-0.20	3.45E-14
2058	cg19297688	INSL4	-0.21	1.17E-11
2059	cg04979933	INSL5	-0.11	9.65E-08
2060	cg07531356	INSL6	-0.18	5.32E-09
2061	cg11830061	INSL6	-0.20	2.53E-12
2062	cg07850604	INSM2	0.30	1.59E-12
2063	cg10670077	INSR	-0.13	3.42E-06
2064	cg14940420	IQCF2	-0.14	9.49E-16
2065	cg10691387	IQCF2	-0.20	6.54E-19
2066	cg19418515	IQCH	-0.11	1.79E-18
2067	cg20395892	IRAK3	0.24	3.44E-12
2068	cg07914866	IRAK3	0.19	4.52E-08
2069	cg12741420	IRF4	0.27	5.05E-14
2070	cg25344367	IRGC	-0.16	1.21E-13
2071	cg15433631	IRX2	0.24	2.99E-07
2072	cg03963198	IRX4	0.31	8.51E-13
2073	cg00243313	IRX4	0.19	4.75E-07
2074	cg05266781	IRX5	0.26	6.70E-11
2075	cg05362516	IRX5	0.18	1.50E-05
2076	cg02161900	IRXL1	0.22	6.24E-09
2077	cg14614211	IRXL1	0.21	1.61E-07
2078	cg00392257	ISG20L2	-0.16	1.37E-07
2079	cg02833725	ISG20L2	-0.16	1.10E-06
2080	cg02077702	ISLR	-0.11	4.22E-05
2081	cg11102782	ISYNA1	0.44	2.10E-24
2082	cg09374949	ISYNA1	0.27	2.88E-11
2083	cg00445824	ISYNA1	0.25	8.73E-09
2084	cg17749520	ITGA2B	-0.12	1.45E-10
2085	cg10488476	ITGA3	0.16	2.65E-05
2086	cg25870420	ITGA9	0.21	1.31E-07
2087	cg02164442	ITGAD	-0.12	7.86E-05
2088	cg24167037	ITGB2	-0.19	1.30E-08
2089	cg19812619	ITGB7	-0.13	6.34E-10
2090	cg17864730	ITGB8	0.11	6.50E-05
2091	cg03386869	ITGBL1	-0.13	2.65E-07
2092	cg06134964	ITIH1	-0.15	9.22E-08
2093	cg26099316	ITIH3	-0.10	3.41E-05
2094	cg01259619	ITPKB	0.11	2.86E-04
2095	cg00495491	ITPR2	-0.13	1.69E-25

2096	cg12650926	ITPR3	0.15	7.78E-06
2097	cg16400825	ITPR3	0.12	3.56E-04
2098	cg09582042	ITR	0.16	1.21E-11
2099	cg25082710	IVL	-0.14	8.03E-15
2100	cg05440289	IVL	-0.27	1.90E-23
2101	cg10234985	IZUMO1	0.22	8.36E-15
2102	cg01519742	JAKMIP1	0.28	6.07E-09
2103	cg13168683	JAKMIP1	0.16	3.49E-06
2104	cg03382304	JAM2	0.11	5.82E-04
2105	cg11344614	JAM2	-0.19	7.92E-14
2106	cg11520395	JMJD4	-0.15	1.67E-11
2107	cg08949296	JPH1	0.13	1.95E-04
2108	cg11845202	K5B	-0.27	6.96E-13
2109	cg07265310	K5B	-0.29	3.16E-24
2110	cg20256783	K6HF	-0.26	1.03E-21
2111	cg17255302	K6HF	-0.28	8.98E-15
2112	cg07017706	K6IRS3	-0.30	1.42E-20
2113	cg13928961	K6IRS3	-0.33	6.69E-23
2114	cg20200335	K6IRS4	-0.23	3.53E-23
2115	cg08126211	KAAG1	0.35	7.31E-17
2116	cg10430690	KALRN	-0.10	1.31E-07
2117	cg12144803	KALRN	-0.22	1.27E-11
2118	cg16842214	KBTBD5	0.14	1.26E-04
2119	cg00412805	KBTBD5	-0.14	6.30E-06
2120	cg07830847	KCNA10	-0.27	9.29E-19
2121	cg26162582	KCNA6	0.15	1.09E-06
2122	cg26450866	KCNA7	0.11	1.47E-04
2123	cg23873703	KCNAB1	-0.13	2.92E-04
2124	cg15423862	KCNAB1	-0.16	1.13E-07
2125	cg19814116	KCNAB2	-0.17	5.99E-08
2126	cg20890210	KCNB2	0.13	1.84E-06
2127	cg27409364	KCNC1	0.38	2.14E-34
2128	cg06572160	KCNC3	0.30	5.35E-23
2129	cg05675373	KCNC4	0.41	7.58E-25
2130	cg23189044	KCNE3	0.17	5.38E-06
2131	cg02595219	KCNE3	0.12	9.93E-04
2132	cg24924779	KCNG1	0.20	2.22E-06
2133	cg27553955	KCNG3	0.28	1.14E-23
2134	cg25101056	KCNG4	-0.19	7.05E-18
2135	cg20406482	KCNH4	0.18	1.35E-06
2136	cg23559331	KCNH4	0.14	7.25E-06
2137	cg07092725	KCNH5	-0.21	1.23E-17
2138	cg24997944	KCNH8	0.10	1.22E-05
2139	cg08422599	KCNIP1	0.15	1.33E-07
2140	cg12833011	KCNIP1	-0.26	1.93E-18
2141	cg04997967	KCNIP2	0.17	7.17E-05
2142	cg14481339	KCNJ1	-0.19	1.06E-14
2143	cg05101920	KCNJ10	0.16	4.55E-08
2144	cg05768141	KCNJ10	0.13	1.22E-05
2145	cg22642718	KCNJ12	-0.22	2.84E-18

2146	cg18248112	KCNJ15	0.11	2.61E-05
2147	cg03993463	KCNJ15	-0.13	1.38E-09
2148	cg20066612	KCNJ9	-0.19	2.97E-12
2149	cg10935723	KCNK10	-0.14	8.66E-12
2150	cg27320127	KCNK12	0.38	8.06E-20
2151	cg02136132	KCNK13	0.12	1.69E-05
2152	cg14948436	KCNK16	-0.22	3.56E-14
2153	cg01669948	KCNK16	-0.25	6.18E-17
2154	cg02611419	KCNK17	-0.17	2.78E-07
2155	cg07637239	KCNK18	-0.16	2.71E-13
2156	cg01643580	KCNK3	0.19	1.10E-07
2157	cg05782445	KCNK3	0.16	1.49E-09
2158	cg17834752	KCNK9	0.26	2.35E-13
2159	cg04688368	KCNMA1	0.13	2.63E-05
2160	cg01495509	KCNMB1	-0.10	2.01E-09
2161	cg22646937	KCNMB1	-0.16	2.85E-25
2162	cg25211252	KCNMB3	-0.18	7.10E-11
2163	cg10778619	KCNMB4	0.17	5.70E-06
2164	cg04833845	KCNN4	-0.19	9.63E-17
2165	cg19779211	KCNQ1	0.34	1.99E-18
2166	cg08303146	KCNQ1	0.32	3.42E-14
2167	cg17575811	KCNQ1	0.17	6.60E-07
2168	cg17820828	KCNQ1	-0.10	6.69E-03
2169	cg01734338	KCNQ1	-0.11	7.52E-09
2170	cg16778148	KCNQ1	-0.12	5.57E-10
2171	cg16609872	KCNQ1	-0.13	2.21E-07
2172	cg20751395	KCNQ1	-0.19	1.09E-09
2173	cg04719766	KCNQ1	-0.19	2.23E-13
2174	cg17229197	KCNQ1	-0.21	9.69E-15
2175	cg08007665	KCNQ1	-0.22	2.53E-13
2176	cg26879282	KCNQ1	-0.24	6.33E-16
2177	cg26750319	KCNQ1	-0.28	1.54E-14
2178	cg12949760	KCNQ1	-0.28	3.77E-22
2179	cg01530101	KCNQ1DN	0.12	1.45E-04
2180	cg09998591	KCNQ1DN	-0.16	1.39E-08
2181	cg18153124	KCNQ3	0.11	1.07E-04
2182	cg00630164	KCNQ4	0.23	1.74E-11
2183	cg24687051	KCNQ5	0.13	8.76E-05
2184	cg05373457	KCNS2	0.30	9.17E-15
2185	cg20673481	KCNS3	0.15	5.32E-05
2186	cg10539808	KCTD1	-0.27	2.59E-15
2187	cg12280407	KCTD12	0.23	2.53E-09
2188	cg22229142	KCTD17	-0.10	1.91E-06
2189	cg12300353	KCTD8	0.12	4.85E-04
2190	cg14323109	KDR	0.12	4.84E-05
2191	cg02507952	KIAA0125	-0.17	2.07E-12
2192	cg13105904	KIAA0323	0.21	1.09E-05
2193	cg11880010	KIAA0367	-0.12	2.74E-06
2194	cg18530324	KIAA0427	0.14	1.43E-05
2195	cg17729667	KIAA0980	0.25	1.12E-09

2196	cg09088834	KIAA0980	0.22	5.17E-09
2197	cg10337772	KIAA1143	0.12	2.55E-04
2198	cg16797831	KIAA1324	0.28	3.22E-16
2199	cg26045220	KIAA1324	0.20	5.97E-08
2200	cg05730092	KIAA1446	-0.11	9.24E-06
2201	cg09173897	KIAA1622	0.14	9.34E-09
2202	cg02867079	KIAA1822	0.14	4.71E-05
2203	cg06834875	KIAA1822	0.10	2.42E-03
2204	cg20582779	KIAA1822L	-0.13	1.89E-06
2205	cg18172186	KIAA1913	-0.23	5.76E-16
2206	cg26781575	KIAA1946	0.12	8.98E-06
2207	cg19795898	KIAA1984	-0.20	7.56E-13
2208	cg15613048	KIF17	0.37	8.93E-15
2209	cg07596494	KIF3C	0.10	4.54E-05
2210	cg04270799	KIF5A	0.22	3.04E-09
2211	cg18628483	KIF5A	-0.16	2.85E-10
2212	cg18221897	KIR2DL1	-0.17	3.02E-17
2213	cg22039846	KIR2DL1	-0.26	1.82E-24
2214	cg24654350	KIR3DL1	-0.24	2.03E-17
2215	cg15627025	KIR3DL1	-0.28	3.32E-25
2216	cg23404467	KIR3DL3	-0.15	1.02E-08
2217	cg11237817	KIR3DL3	-0.23	1.80E-19
2218	cg26870566	KIRREL	-0.19	3.77E-11
2219	cg09479015	KIRREL2	0.10	2.18E-04
2220	cg05606799	KISS1	-0.11	3.64E-05
2221	cg23282559	KL	0.23	1.10E-08
2222	cg03883348	KLC3	0.14	3.53E-05
2223	cg04528819	KLF14	0.28	6.15E-11
2224	cg18202456	KLF17	-0.13	2.83E-10
2225	cg26842024	KLF2	0.13	2.59E-03
2226	cg07309102	KLF4	0.13	1.61E-03
2227	cg13507429	KLHDC7A	-0.12	4.29E-08
2228	cg18533225	KLHDC7B	0.16	2.15E-11
2229	cg20349377	KLHL1	-0.15	8.72E-15
2230	cg20425130	KLHL6	0.11	6.25E-07
2231	cg26415633	KLK1	-0.10	4.07E-10
2232	cg11846956	KLK10	0.13	1.08E-04
2233	cg08818784	KLK10	0.12	1.04E-05
2234	cg23894058	KLK12	-0.15	6.38E-11
2235	cg05242523	KLK14	-0.13	1.33E-05
2236	cg12683641	KLK14	-0.16	1.53E-15
2237	cg04744379	KLK15	-0.12	1.19E-11
2238	cg26149550	KLK15	-0.25	4.16E-16
2239	cg07947016	KLK2	-0.21	1.08E-25
2240	cg17687962	KLK3	-0.26	7.30E-19
2241	cg10078829	KLK4	-0.15	1.22E-14
2242	cg15096123	KLK4	-0.22	8.88E-16
2243	cg04349727	KLK5	-0.19	8.92E-16
2244	cg27583102	KLK5	-0.21	5.05E-11
2245	cg21256656	KLK6	-0.25	1.13E-16

2246	cg18967533	KLK6	-0.25	5.78E-21
2247	cg01144251	KLK9	-0.25	4.93E-20
2248	cg22468123	KLP1	0.17	2.13E-04
2249	cg20047055	KLRC1	-0.23	2.09E-19
2250	cg21474838	KLRD1	-0.19	4.21E-12
2251	cg17405586	KRT1	-0.21	1.48E-23
2252	cg02756698	KRT12	-0.15	9.99E-06
2253	cg10742225	KRT13	-0.11	1.28E-10
2254	cg13350783	KRT13	-0.30	6.11E-19
2255	cg11068096	KRT14	-0.20	7.81E-21
2256	cg01602596	KRT14	-0.21	1.52E-16
2257	cg11983245	KRT15	-0.25	3.04E-16
2258	cg23128056	KRT15	-0.28	1.58E-17
2259	cg24457403	KRT16	-0.17	8.54E-07
2260	cg27478659	KRT16	-0.27	4.30E-24
2261	cg00214794	KRT17	-0.15	8.39E-20
2262	cg18982568	KRT1B	-0.16	5.17E-13
2263	cg05921581	KRT1B	-0.20	8.91E-13
2264	cg25124433	KRT20	-0.17	2.70E-10
2265	cg18901980	KRT25A	-0.29	1.31E-18
2266	cg22983092	KRT25A	-0.30	2.47E-17
2267	cg04484789	KRT25B	-0.22	1.20E-19
2268	cg16488522	KRT25C	-0.15	2.28E-12
2269	cg10173723	KRT25C	-0.20	2.32E-14
2270	cg00463848	KRT2A	-0.23	7.39E-22
2271	cg16405957	KRT2A	-0.25	2.69E-16
2272	cg20862119	KRT2B	-0.27	3.49E-15
2273	cg11465372	KRT2B	-0.31	7.49E-18
2274	cg12610744	KRT4	-0.12	9.79E-11
2275	cg17667972	KRT4	-0.13	4.57E-05
2276	cg23645091	KRT5	-0.24	5.05E-14
2277	cg04254916	KRT5	-0.29	2.87E-17
2278	cg11471401	KRT6A	-0.16	4.94E-13
2279	cg12795208	KRT6B	-0.22	3.94E-14
2280	cg13181284	KRT6B	-0.30	8.29E-20
2281	cg16112157	KRT6C	-0.14	2.44E-12
2282	cg00213044	KRT6C	-0.24	4.62E-23
2283	cg08136806	KRT6E	-0.10	1.92E-06
2284	cg26577529	KRT6E	-0.17	1.70E-10
2285	cg01853981	KRT6IRS	-0.21	1.31E-16
2286	cg23767977	KRT6IRS	-0.28	5.67E-17
2287	cg23696712	KRT6L	-0.27	4.64E-16
2288	cg09522147	KRT7	0.12	2.17E-04
2289	cg07007400	KRT7	0.11	6.62E-05
2290	cg10328573	KRT9	-0.19	1.44E-15
2291	cg02784874	KRT9	-0.28	4.50E-25
2292	cg17778867	KRTAP10-8	-0.11	1.67E-09
2293	cg07014174	KRTAP11-1	-0.18	1.35E-15
2294	cg27071517	KRTAP13-1	-0.27	2.31E-23
2295	cg16431978	KRTAP13-3	-0.20	2.71E-23

2296	cg14062083	KRTAP13-4	-0.21	5.67E-19
2297	cg15606663	KRTAP15-1	-0.19	4.17E-22
2298	cg16812893	KRTAP15-1	-0.32	3.83E-29
2299	cg26499286	KRTAP17-1	-0.11	1.68E-13
2300	cg16242770	KRTAP17-1	-0.26	3.45E-15
2301	cg04297093	KRTAP19-3	-0.20	1.74E-10
2302	cg06748315	KRTAP19-3	-0.20	6.43E-17
2303	cg07374637	KRTAP19-5	-0.25	1.11E-17
2304	cg06324671	KRTAP19-7	-0.25	1.19E-17
2305	cg26565975	KRTAP20-1	-0.26	1.13E-15
2306	cg06222800	KRTAP21-1	-0.15	1.06E-14
2307	cg27456885	KRTAP22-1	-0.13	5.20E-12
2308	cg23205633	KRTAP4-2	-0.25	5.47E-15
2309	cg14087150	KRTAP4-4	-0.22	2.01E-19
2310	cg03029616	KRTAP6-1	-0.27	3.08E-19
2311	cg19306866	KRTAP6-2	-0.23	1.99E-19
2312	cg24423088	KRTAP8-1	-0.35	1.01E-19
2313	cg11710560	KRTAP9-3	-0.28	5.84E-20
2314	cg11618577	KRTCAP3	0.24	2.90E-22
2315	cg00063144	KRTHA1	-0.26	2.78E-15
2316	cg21230133	KRTHA1	-0.36	2.38E-24
2317	cg08611714	KRTHA2	-0.27	1.43E-23
2318	cg23674788	KRTHA2	-0.34	8.55E-24
2319	cg18370227	KRTHA3A	-0.29	4.23E-16
2320	cg15983005	KRTHA3A	-0.38	3.97E-25
2321	cg02580606	KRTHA3B	-0.28	1.07E-20
2322	cg14533138	KRTHA3B	-0.39	1.23E-26
2323	cg06074920	KRTHA4	-0.17	4.29E-10
2324	cg25545210	KRTHA4	-0.38	7.42E-26
2325	cg00384537	KRTHA5	-0.15	3.78E-12
2326	cg09538582	KRTHA5	-0.20	7.65E-16
2327	cg02780988	KRTHA6	-0.26	2.39E-21
2328	cg04618528	KRTHA6	-0.30	4.13E-18
2329	cg22139563	KRTHA8	-0.12	1.41E-08
2330	cg13696887	KRTHB2	-0.17	1.29E-09
2331	cg24926042	KRTHB2	-0.23	3.00E-15
2332	cg19258973	KRTHB3	-0.24	1.76E-16
2333	cg16791508	KRTHB3	-0.26	6.66E-18
2334	cg18153060	KRTHB4	-0.27	8.18E-17
2335	cg24461814	KRTHB4	-0.30	3.66E-16
2336	cg06132342	KRTHB5	-0.26	6.71E-18
2337	cg21518208	KRTHB5	-0.30	1.49E-19
2338	cg16911220	KRTHB6	-0.12	9.78E-09
2339	cg07643942	LACRT	-0.13	1.69E-09
2340	cg10125195	LACRT	-0.25	7.08E-20
2341	cg00616135	LACTB	0.11	4.34E-05
2342	cg26956535	LAG3	0.10	1.10E-07
2343	cg06238491	LAIR1	-0.10	4.22E-04
2344	cg20320468	LAIR1	-0.21	3.26E-16
2345	cg04000821	LAIR2	-0.17	4.32E-17

2346	cg00269932	LAIR2	-0.26	1.93E-16
2347	cg01726767	LALBA	-0.16	7.18E-11
2348	cg14338548	LALBA	-0.25	1.84E-15
2349	cg07846220	LAMA1	0.23	9.62E-06
2350	cg23696949	LAMC2	0.21	6.90E-09
2351	cg26491484	LAMC3	-0.17	8.04E-09
2352	cg12732155	LAPTM5	-0.11	1.50E-03
2353	cg01318557	LAT2	-0.27	3.38E-10
2354	cg12022621	LAX1	-0.16	1.41E-14
2355	cg14696820	LCE1A	-0.23	9.18E-22
2356	cg21434954	LCE1B	-0.23	5.60E-21
2357	cg08878744	LCE1B	-0.27	9.33E-27
2358	cg24304714	LCE1C	-0.13	2.09E-17
2359	cg15531099	LCE1D	-0.28	6.94E-26
2360	cg21065959	LCE1E	-0.22	1.04E-20
2361	cg23413307	LCE1F	-0.34	5.83E-25
2362	cg25098401	LCE2B	-0.19	1.93E-21
2363	cg03960217	LCE2C	-0.20	6.38E-24
2364	cg21312148	LCE2D	-0.15	1.59E-08
2365	cg26135325	LCE3A	-0.14	7.32E-07
2366	cg03741352	LCE3C	-0.26	1.47E-21
2367	cg20676475	LCE3D	-0.16	1.57E-12
2368	cg23110514	LCE3E	-0.11	4.03E-08
2369	cg21846488	LCE4A	-0.23	6.67E-18
2370	cg05248781	LCE5A	-0.34	1.79E-17
2371	cg10248727	LCN1	-0.20	6.52E-21
2372	cg23815000	LCN1	-0.25	8.96E-16
2373	cg11873854	LCN6	-0.15	6.48E-15
2374	cg17127769	LCP2	-0.11	2.59E-05
2375	cg03368758	LDB2	-0.11	1.93E-05
2376	cg12008118	LDB3	-0.12	7.46E-10
2377	cg14813083	LDHAL6A	0.12	1.50E-05
2378	cg07915343	LDHAL6A	0.11	4.79E-09
2379	cg06437004	LDHB	0.49	5.31E-32
2380	cg03243946	LDHB	0.34	2.22E-15
2381	cg08418111	LDHC	0.13	7.46E-06
2382	cg20661303	LEFTY2	-0.17	2.13E-13
2383	cg12782180	LEP	0.25	3.57E-14
2384	cg19594666	LEP	0.11	1.81E-03
2385	cg20270599	LEPREL1	0.11	4.05E-05
2386	cg08958913	LGALS14	-0.11	2.20E-10
2387	cg01192952	LGALS14	-0.15	2.75E-11
2388	cg19099850	LGALS3	0.45	2.17E-15
2389	cg17403875	LGALS3	0.41	2.97E-14
2390	cg14870271	LGALS3BP	0.12	3.46E-05
2391	cg11105610	LGALS3BP	0.12	4.82E-08
2392	cg06394229	LGALS4	-0.12	6.40E-05
2393	cg00532335	LGI1	-0.18	2.20E-14
2394	cg04833050	LHFPL2	0.11	1.41E-03
2395	cg22660578	LHX1	0.28	2.59E-13

2396	cg07109287	LHX2	0.10	2.13E-02
2397	cg20300246	LHX3	0.16	1.03E-05
2398	cg25680829	LHX4	0.14	7.76E-06
2399	cg06866657	LHX6	0.16	1.27E-08
2400	cg19539004	LIF	-0.14	2.61E-09
2401	cg01796228	LIFR	-0.21	1.67E-18
2402	cg25384595	LILRA1	-0.22	2.63E-24
2403	cg19486673	LILRA2	-0.18	1.61E-16
2404	cg00705255	LILRA3	-0.15	1.88E-30
2405	cg13733733	LILRA3	-0.23	8.49E-16
2406	cg20542190	LILRA4	-0.12	2.79E-18
2407	cg01204985	LILRA4	-0.26	2.76E-16
2408	cg06392096	LILRA5	-0.12	7.73E-30
2409	cg00727947	LILRA5	-0.26	2.19E-18
2410	cg05248470	LILRB2	-0.11	1.13E-09
2411	cg22456522	LILRB3	-0.20	2.57E-16
2412	cg00113020	LILRB4	-0.14	2.02E-09
2413	cg05922591	LILRB4	-0.26	3.82E-15
2414	cg20649991	LILRB5	-0.27	8.01E-19
2415	cg08684473	LILRB5	-0.29	8.92E-18
2416	cg02988947	LIMD2	0.19	1.80E-13
2417	cg05592398	LIMK1	0.18	1.16E-08
2418	cg00585790	LIMS1	0.15	3.45E-10
2419	cg01733599	LIPC	-0.11	5.03E-06
2420	cg24447890	LMAN1L	-0.10	9.20E-06
2421	cg11822932	LMO2	-0.10	1.27E-06
2422	cg09358725	LMO2	-0.11	4.12E-06
2423	cg22227965	LMO7	-0.11	3.18E-10
2424	cg09636671	LMOD1	0.20	1.09E-06
2425	cg09404633	LMOD1	0.11	1.24E-03
2426	cg24650501	LMX1A	0.10	1.88E-05
2427	cg25999867	LOC112937	0.15	1.94E-06
2428	cg19674669	LOC112937	0.11	7.94E-04
2429	cg02718531	LOC114984	0.16	3.06E-05
2430	cg24272559	LOC116123	-0.19	1.94E-11
2431	cg07580827	LOC120379	-0.11	4.03E-10
2432	cg21624282	LOC122258	-0.17	1.77E-12
2433	cg05461841	LOC124220	-0.14	9.80E-14
2434	cg26259865	LOC124220	-0.19	3.79E-12
2435	cg26687173	LOC126248	0.12	3.35E-18
2436	cg05719902	LOC136306	0.17	3.54E-13
2437	cg24506604	LOC144501	-0.14	1.58E-07
2438	cg15660498	LOC148137	0.11	2.09E-04
2439	cg14681629	LOC153684	0.10	3.72E-05
2440	cg06186808	LOC161247	0.22	3.61E-16
2441	cg17561452	LOC161931	-0.17	2.36E-13
2442	cg07595943	LOC161931	-0.23	1.08E-13
2443	cg11456838	LOC202459	-0.17	2.80E-12
2444	cg25650811	LOC223075	-0.13	6.85E-13
2445	cg04992673	LOC253012	-0.24	6.92E-13

2446	cg11608424	LOC253012	-0.24	1.48E-13
2447	cg22131172	LOC283487	-0.16	2.08E-10
2448	cg01605783	LOC284837	-0.15	2.97E-08
2449	cg00546897	LOC284837	-0.26	1.19E-16
2450	cg16983159	LOC340061	0.16	1.39E-08
2451	cg07434382	LOC348174	-0.17	4.30E-19
2452	cg15149938	LOC348645	-0.17	7.37E-11
2453	cg01870826	LOC389458	0.35	5.24E-18
2454	cg18401406	LOC400120	-0.17	4.15E-08
2455	cg00881370	LOC55908	0.17	2.99E-07
2456	cg15309006	LOC63928	0.15	1.07E-04
2457	cg27011193	LOC654342	0.12	9.48E-12
2458	cg11012046	LOC89944	0.26	4.95E-12
2459	cg11781389	LOC89944	0.22	4.66E-12
2460	cg17761453	LOR	-0.24	1.00E-15
2461	cg27626424	LOR	-0.31	2.18E-20
2462	cg02548238	LOX	0.17	4.42E-05
2463	cg22836153	LOX	0.10	2.54E-04
2464	cg08918749	LPL	0.11	1.33E-03
2465	cg24489015	LPO	-0.15	1.92E-12
2466	cg20654468	LPXN	0.26	7.19E-18
2467	cg23587449	LRAT	0.19	4.35E-08
2468	cg23303074	LRAT	0.13	7.05E-06
2469	cg20580177	LRFN3	-0.13	2.12E-05
2470	cg20358834	LRFN4	0.35	5.23E-14
2471	cg08700306	LRP3	-0.11	5.64E-06
2472	cg25201363	LRPAP1	0.12	1.11E-07
2473	cg02121427	LRRC15	-0.12	8.46E-09
2474	cg26838900	LRRC15	-0.25	1.32E-15
2475	cg20442697	LRRC17	0.12	8.97E-12
2476	cg07514381	LRRC2	-0.16	6.99E-13
2477	cg17833578	LRRC21	-0.12	6.89E-10
2478	cg17827767	LRRC21	-0.27	4.01E-23
2479	cg23089840	LRRC3	0.17	3.86E-11
2480	cg09610963	LRRC37A	-0.11	6.10E-20
2481	cg18020749	LRRC37A	-0.13	1.33E-16
2482	cg15087147	LRRC4	0.18	9.78E-14
2483	cg10576828	LRRC7	-0.25	2.56E-18
2484	cg21401794	LRRC8C	0.13	1.01E-04
2485	cg16456919	LRRN5	-0.11	1.80E-03
2486	cg01909245	LSP1	-0.20	9.92E-12
2487	cg26158194	LSP1	-0.23	1.05E-11
2488	cg14324675	LST1	-0.17	7.53E-09
2489	cg17709873	LTA	-0.20	6.91E-13
2490	cg20542800	LTB4R	0.10	1.76E-04
2491	cg15784615	LTBR	-0.11	3.61E-04
2492	cg11394785	LTC4S	0.13	4.72E-08
2493	cg16361890	LTC4S	0.11	6.50E-09
2494	cg15261665	LTF	0.12	1.13E-04
2495	cg27494383	LTK	0.19	3.73E-10

2496	cg07654934	LXN	0.28	4.75E-20
2497	cg08263647	LXN	0.16	2.73E-16
2498	cg07572435	LY6D	-0.27	2.33E-20
2499	cg05800321	LY6D	-0.28	3.10E-18
2500	cg27557796	LY6G6C	-0.16	3.76E-09
2501	cg10107725	LY75	0.18	3.37E-10
2502	cg23995753	LY75	0.14	3.19E-04
2503	cg02212836	LY86	-0.17	3.02E-07
2504	cg01367992	LY9	-0.15	5.42E-10
2505	cg18920397	LY9	-0.22	1.94E-10
2506	cg23180489	LYNX1	0.18	2.29E-13
2507	cg04311964	LYPD2	-0.13	3.25E-11
2508	cg15014458	LYPD3	0.33	1.98E-30
2509	cg25340403	LYPD3	0.21	1.50E-26
2510	cg24116028	LYPD4	-0.14	1.07E-09
2511	cg21044104	LYZL4	-0.27	6.87E-19
2512	cg16826286	LYZL6	-0.19	9.62E-13
2513	cg26917999	LZTS1	-0.18	3.41E-10
2514	cg08385610	LZTS1	-0.23	1.46E-15
2515	cg12029639	MAB21L1	-0.15	2.43E-11
2516	cg05093686	MAB21L1	-0.23	8.10E-15
2517	cg22233974	MACF1	0.11	2.79E-10
2518	cg26365553	MADD	0.15	1.36E-03
2519	cg02497758	MAFB	-0.13	7.68E-11
2520	cg14535518	MAG	-0.15	1.36E-14
2521	cg05055150	MAG	-0.15	5.45E-10
2522	cg01678091	MAGEL2	-0.14	3.46E-08
2523	cg02982734	MAGEL2	-0.14	6.70E-12
2524	cg04014889	MAGEL2	-0.16	3.98E-10
2525	cg21325760	MAGEL2	-0.17	2.90E-11
2526	cg20537629	MAGI2	0.12	4.32E-05
2527	cg01615704	MALL	0.15	6.24E-05
2528	cg08880153	MAP1A	-0.20	5.28E-13
2529	cg07380496	MAP1B	0.29	1.07E-12
2530	cg02001410	MAP1B	0.17	1.59E-08
2531	cg19601328	MAP3K14	0.14	7.07E-13
2532	cg15639951	MAP3K14	0.14	2.05E-04
2533	cg00233307	MAP4K1	0.22	4.64E-09
2534	cg15679651	MAP4K1	0.21	4.21E-12
2535	cg12727374	MAP6D1	0.45	1.11E-20
2536	cg03705396	MAP6D1	0.43	3.93E-26
2537	cg24155668	MAPK13	0.13	1.63E-04
2538	cg22892110	MAPK15	0.40	1.39E-23
2539	cg03568064	MAPK8IP1	0.26	9.17E-08
2540	cg17271365	MARCH1	-0.20	5.63E-21
2541	cg07259382	MARCH1	-0.22	1.79E-17
2542	cg25842356	MARCH3	0.14	1.06E-11
2543	cg11009736	MARCO	-0.18	1.77E-18
2544	cg02431964	MARCO	-0.28	2.44E-19
2545	cg24063382	MAS1L	-0.21	7.64E-20

2546	cg21831174	MASP1	0.14	2.44E-14
2547	cg13833831	MATK	-0.17	3.46E-09
2548	cg10039928	MATN2	-0.10	4.70E-07
2549	cg14448104	MATN4	-0.18	4.80E-09
2550	cg27418851	MBL2	-0.12	3.40E-07
2551	cg12555907	MBP	0.12	2.71E-08
2552	cg11564670	MC2R	-0.20	1.49E-15
2553	cg19226099	MC3R	-0.26	1.08E-20
2554	cg02037013	MC4R	-0.19	8.87E-15
2555	cg21096399	MCAM	0.16	3.10E-05
2556	cg03365354	MCAM	0.13	5.99E-04
2557	cg11668844	MCF2L	0.12	1.49E-04
2558	cg27239157	MCF2L2	0.25	1.77E-12
2559	cg23752923	MCOLN3	0.14	2.16E-07
2560	cg08223748	MEF2C	0.11	7.21E-06
2561	cg21514871	MEFV	0.14	4.55E-08
2562	cg02490034	MEST	-0.11	1.25E-07
2563	cg08077673	MEST	-0.11	2.33E-06
2564	cg09872616	MEST	-0.11	4.39E-08
2565	cg09059945	MEST	-0.11	2.83E-10
2566	cg01888566	MEST	-0.15	2.05E-08
2567	cg11027330	METRN	0.13	1.28E-05
2568	cg08477744	MFAP2	-0.13	1.56E-10
2569	cg13030582	MFAP4	0.10	1.50E-05
2570	cg06256735	MFAP5	-0.19	1.95E-16
2571	cg15815843	MFAP5	-0.27	1.31E-20
2572	cg18452324	MFNG	0.12	4.70E-04
2573	cg15075718	MFRP	-0.16	6.25E-07
2574	cg19560971	MFRP	-0.16	1.41E-15
2575	cg17952262	MFSD7	0.22	2.52E-13
2576	cg24693053	MFSD7	0.20	2.71E-14
2577	cg18971054	MGAM	-0.26	4.62E-13
2578	cg18344063	MGAT4C	-0.18	7.55E-13
2579	cg03862760	MGC10911	-0.14	1.07E-16
2580	cg20687462	MGC10992	0.11	1.56E-04
2581	cg12108912	MGC10993	-0.18	5.52E-14
2582	cg05937237	MGC11257	-0.10	2.20E-10
2583	cg26608667	MGC11257	-0.14	3.42E-09
2584	cg21517055	MGC11271	0.15	4.04E-06
2585	cg12960248	MGC11335	0.12	1.71E-07
2586	cg05731779	MGC13034	-0.19	1.60E-12
2587	cg15905124	MGC13034	-0.24	5.68E-19
2588	cg20346726	MGC13040	0.14	2.16E-06
2589	cg00202702	MGC13040	0.10	5.92E-05
2590	cg18798750	MGC14289	0.29	1.02E-10
2591	cg00042156	MGC16291	-0.18	1.57E-09
2592	cg04726446	MGC16372	0.42	6.99E-26
2593	cg10883621	MGC16372	0.34	6.70E-23
2594	cg11277230	MGC26744	0.11	7.63E-05
2595	cg21087137	MGC26856	0.11	6.07E-06

2596	cg22496683	MGC27016	-0.12	2.99E-05
2597	cg02992596	MGC27016	-0.26	5.73E-15
2598	cg26790059	MGC27121	-0.11	1.66E-05
2599	cg18750756	MGC29671	-0.21	2.20E-10
2600	cg01138020	MGC29671	-0.23	2.68E-13
2601	cg04731384	MGC3101	-0.23	6.51E-15
2602	cg12452946	MGC33302	0.11	4.56E-11
2603	cg15557833	MGC33302	0.10	2.27E-09
2604	cg24642468	MGC33367	-0.18	1.37E-17
2605	cg07804582	MGC33367	-0.19	2.07E-11
2606	cg18931750	MGC33407	-0.12	1.40E-07
2607	cg00775197	MGC33600	0.12	1.14E-05
2608	cg21553524	MGC33926	0.15	1.72E-05
2609	cg19540408	MGC35154	-0.14	1.35E-15
2610	cg11784281	MGC35154	-0.17	1.09E-12
2611	cg14074117	MGC35212	-0.11	4.66E-06
2612	cg25072962	MGC35295	-0.22	3.30E-22
2613	cg23499129	MGC35308	0.22	6.03E-10
2614	cg10261191	MGC39545	0.12	4.98E-12
2615	cg15658426	MGC39633	0.19	2.99E-12
2616	cg24400943	MGC39681	-0.18	7.80E-11
2617	cg07484450	MGC39681	-0.22	8.51E-13
2618	cg24546463	MGC39715	0.21	6.63E-11
2619	cg17199658	MGC39715	0.16	1.09E-08
2620	cg23502772	MGC42105	0.24	1.57E-09
2621	cg05174079	MGC42630	-0.15	4.01E-15
2622	cg22543128	MGC42630	-0.19	4.89E-16
2623	cg14759043	MGC4266	0.13	2.03E-03
2624	cg06154597	MGC4618	-0.18	6.11E-11
2625	cg00448720	MGC48915	-0.14	1.01E-07
2626	cg12594641	MGC52057	-0.11	5.71E-08
2627	cg14086122	MGC7036	-0.14	2.28E-08
2628	cg00411097	MGC9712	-0.12	5.40E-10
2629	cg06194808	MGC9712	-0.16	9.90E-08
2630	cg20377673	MIF	0.12	2.00E-03
2631	cg06534422	MIF	0.12	8.56E-03
2632	cg15147516	MIXL1	0.42	2.20E-20
2633	cg18354264	MIXL1	0.12	7.56E-05
2634	cg21030400	MKNK2	0.22	8.91E-16
2635	cg20769842	MKRN3	-0.12	8.51E-10
2636	cg23234999	MKRN3	-0.20	2.65E-15
2637	cg03969797	MKRN3	-0.20	2.17E-14
2638	cg17169998	MLC1	-0.15	7.16E-07
2639	cg20182358	MLF1	0.31	1.49E-17
2640	cg20226764	MLN	-0.17	4.22E-13
2641	cg08332212	MLN	-0.19	6.07E-10
2642	cg07935568	MLNR	0.40	1.30E-17
2643	cg02620013	MLNR	0.34	1.43E-21
2644	cg14026971	MLSTD2	0.22	1.14E-08
2645	cg24243265	MLZE	-0.16	1.14E-10

2646	cg16575408	MMP1	-0.19	6.61E-10
2647	cg10599444	MMP14	-0.11	1.92E-05
2648	cg26705553	MMP25	0.16	3.85E-07
2649	cg12493906	MMP26	-0.35	4.10E-32
2650	cg06259570	MMP27	-0.11	1.59E-06
2651	cg18113270	MMP3	-0.18	1.03E-08
2652	cg20925811	MMP9	-0.11	7.53E-05
2653	cg19368582	MMRN2	-0.17	1.84E-13
2654	cg25119415	MNDA	-0.24	1.50E-30
2655	cg19382714	MOG	-0.20	3.07E-14
2656	cg03589001	MORF4L1	0.14	3.35E-09
2657	cg07570142	MOXD1	0.26	1.31E-10
2658	cg25473396	MPG	-0.11	7.61E-06
2659	cg13828047	MPI	0.13	1.82E-07
2660	cg06312477	MPN2	-0.11	5.10E-11
2661	cg20342082	MPN2	-0.13	5.21E-07
2662	cg09421562	MPO	-0.11	8.96E-07
2663	cg04988978	MPO	-0.13	2.13E-10
2664	cg03557733	MPP2	0.18	5.86E-05
2665	cg07112154	MPP2	0.15	2.63E-05
2666	cg17749384	MPP7	-0.12	6.88E-06
2667	cg22933847	MRGPRF	0.24	8.49E-16
2668	cg23001457	MRGPRX1	-0.22	2.08E-24
2669	cg24252809	MRGPRX1	-0.28	3.82E-20
2670	cg13062935	MRGPRX2	-0.13	2.52E-05
2671	cg16446783	MRGPRX4	-0.11	1.09E-07
2672	cg20275133	MRPL20	0.12	1.84E-11
2673	cg24541550	MRVI1	-0.16	9.91E-08
2674	cg24365867	MRVI1	-0.19	1.08E-09
2675	cg06806711	MS4A1	-0.21	5.97E-16
2676	cg07221454	MS4A10	-0.15	6.69E-10
2677	cg23307338	MS4A12	-0.18	1.45E-12
2678	cg10414946	MS4A2	-0.10	4.08E-07
2679	cg17173423	MS4A3	-0.21	2.84E-18
2680	cg03634982	MS4A4A	-0.12	6.72E-07
2681	cg06066303	MS4A5	-0.27	3.43E-18
2682	cg04353769	MS4A6A	-0.13	1.21E-08
2683	cg18343292	MS4A7	-0.14	1.00E-21
2684	cg10853416	MS4A7	-0.34	7.94E-27
2685	cg23710218	MSC	0.25	1.50E-14
2686	cg08244028	MSH3	-0.12	2.91E-06
2687	cg06100324	MSLN	-0.17	3.21E-11
2688	cg17501569	MSLN	-0.22	1.56E-14
2689	cg16303562	MSR1	-0.22	3.34E-27
2690	cg01668126	MSR1	-0.23	2.40E-18
2691	cg03332271	MST1R	0.17	2.85E-07
2692	cg15696781	MSTO1	0.17	2.58E-07
2693	cg20588069	MSX1	0.22	1.31E-09
2694	cg24840099	MSX1	0.21	2.64E-10
2695	cg03843978	MSX1	0.19	1.18E-17

2696	cg20891301	MSX1	0.17	1.17E-15
2697	cg03199651	MSX1	0.16	5.59E-08
2698	cg20161179	MSX1	0.16	8.04E-07
2699	cg09748975	MSX1	0.15	5.56E-17
2700	cg06094150	MT1B	-0.10	2.42E-06
2701	cg16158681	MT3	0.35	1.94E-17
2702	cg22655224	MT3	0.10	5.04E-04
2703	cg01015871	MT4	-0.17	1.92E-11
2704	cg24633648	MTAC2D1	0.11	2.31E-06
2705	cg15737319	MTHFD2	0.37	8.62E-21
2706	cg17687883	MTHFD2	0.22	1.23E-08
2707	cg24620905	MTMR11	-0.11	6.69E-10
2708	cg06039392	MTNR1A	0.10	1.84E-04
2709	cg15842276	MTNR1B	0.23	2.11E-27
2710	cg03087937	MUC15	-0.15	7.83E-08
2711	cg16215361	MUC15	-0.19	1.90E-11
2712	cg01584473	MUC17	-0.18	2.00E-13
2713	cg07873488	MUC17	-0.21	4.56E-17
2714	cg00644033	MUC3B	-0.13	1.62E-10
2715	cg15589427	MUC4	-0.24	5.37E-17
2716	cg03818682	MUC5AC	-0.19	2.95E-18
2717	cg03609102	MUC5B	-0.17	1.57E-12
2718	cg22407504	MUC5B	-0.21	7.30E-13
2719	cg22289115	MUCDHL	-0.14	1.35E-06
2720	cg21130374	MX2	-0.12	1.64E-04
2721	cg04828792	MX2	-0.27	5.47E-17
2722	cg05832051	MYADM	0.40	5.02E-20
2723	cg26207503	MYF5	0.27	2.03E-18
2724	cg21126707	MYF5	0.11	2.34E-04
2725	cg26711820	MYF6	0.10	1.24E-05
2726	cg23400451	MYH4	-0.11	2.79E-07
2727	cg21301148	MYH6	-0.10	1.99E-13
2728	cg14541311	MYH6	-0.30	1.03E-19
2729	cg01429391	MYH7	-0.11	8.45E-06
2730	cg12339029	MYL1	-0.24	3.60E-21
2731	cg24353217	MYL2	-0.13	8.96E-07
2732	cg10591174	MYL2	-0.14	6.47E-11
2733	cg15439078	MYL3	-0.11	3.34E-15
2734	cg05294455	MYL4	-0.14	3.52E-12
2735	cg23595927	MYL5	-0.21	5.59E-15
2736	cg23370883	MYL7	-0.14	1.18E-14
2737	cg09786221	MYL9	0.35	2.11E-12
2738	cg21671476	MYL9	0.17	9.51E-11
2739	cg04376312	MYLK	0.16	1.10E-06
2740	cg03731616	MYLK	0.11	1.81E-04
2741	cg19961522	MYLK2	-0.10	1.07E-08
2742	cg08860143	MYLPF	-0.11	5.61E-09
2743	cg07862358	MYO10	0.30	6.02E-12
2744	cg19796273	MYO10	0.22	6.47E-11
2745	cg25013838	MYO18B	-0.12	6.81E-06

2746	cg23771603	MYO3A	0.19	6.71E-06
2747	cg08441170	MYO3A	0.10	5.31E-03
2748	cg23287547	MYO5A	0.14	2.11E-06
2749	cg04882759	MYOCD	-0.27	1.84E-15
2750	cg18555440	MYOD1	0.40	6.16E-25
2751	cg07271264	MYOD1	0.15	2.17E-04
2752	cg24322623	MYOD1	0.13	5.39E-06
2753	cg10677144	MYOM1	-0.14	1.32E-05
2754	cg25070010	MYRIP	0.12	4.66E-04
2755	cg10071275	MYT1	-0.14	3.35E-08
2756	cg16772207	MYT1	-0.19	1.16E-10
2757	cg17894008	NACAL	-0.17	3.87E-08
2758	cg18484189	NALP10	-0.19	5.73E-15
2759	cg20311730	NALP10	-0.29	9.15E-23
2760	cg03789934	NALP11	-0.12	1.54E-15
2761	cg25866075	NALP12	-0.16	1.48E-09
2762	cg07042144	NALP12	-0.22	4.66E-16
2763	cg25203856	NALP14	-0.19	2.48E-14
2764	cg02347487	NALP14	-0.26	8.56E-18
2765	cg16106497	NALP2	-0.11	3.12E-04
2766	cg18059223	NALP2	-0.17	3.72E-09
2767	cg14930706	NALP4	-0.10	2.44E-06
2768	cg26264314	NALP5	-0.21	7.75E-12
2769	cg05023116	NALP5	-0.25	7.31E-17
2770	cg14876043	NALP7	-0.13	1.53E-09
2771	cg08728865	NALP7	-0.20	3.72E-12
2772	cg22190114	NALP8	-0.17	5.65E-15
2773	cg08475088	NALP9	-0.28	3.89E-19
2774	cg15752043	NANOS1	0.23	7.09E-06
2775	cg14916079	NANOS1	0.21	4.88E-12
2776	cg15537850	NAPRT1	-0.13	2.03E-07
2777	cg19136075	NBL1	-0.13	2.61E-09
2778	cg07131544	NCR2	-0.18	3.88E-10
2779	cg04303901	NCRMS	-0.11	7.64E-05
2780	cg00689010	NCSTN	-0.13	5.87E-07
2781	cg13828758	NDN	-0.12	1.84E-03
2782	cg01989224	NDN	-0.16	6.60E-11
2783	cg18081258	NDRG2	0.34	6.83E-22
2784	cg00687686	NDRG4	0.15	1.07E-05
2785	cg01536400	NDUFA8	-0.14	1.13E-10
2786	cg18192417	NEBL	0.28	3.82E-10
2787	cg24625388	NEBL	0.13	6.44E-05
2788	cg23290344	NEF3	0.35	2.52E-13
2789	cg18267374	NEF3	0.30	6.79E-12
2790	cg02994956	NEFH	0.28	3.11E-18
2791	cg16042149	NEFH	0.15	1.19E-05
2792	cg04645342	NEGR1	0.12	1.27E-04
2793	cg12978308	NEIL1	0.15	1.30E-04
2794	cg12600197	NEIL1	0.12	3.98E-04
2795	cg27065979	NEK3	-0.10	1.15E-05

2796	cg21838334	NES	0.14	4.12E-05
2797	cg02755525	NETO2	0.52	1.17E-22
2798	cg11634198	NEUROD2	0.16	2.85E-06
2799	cg26312920	NEUROD6	-0.16	6.89E-17
2800	cg11554507	NEUROD6	-0.24	1.63E-19
2801	cg14958635	NEUROG1	0.19	3.60E-06
2802	cg09134003	NEUROG2	0.14	1.64E-05
2803	cg22289810	NF1	0.12	2.85E-04
2804	cg03017264	NFAM1	-0.16	3.82E-15
2805	cg22571530	NFASC	0.19	6.85E-07
2806	cg07986525	NFE2L3	0.20	3.13E-06
2807	cg15501381	NGB	0.10	1.40E-04
2808	cg20059312	NGEF	-0.15	8.38E-07
2809	cg17129388	NGFR	-0.12	5.28E-05
2810	cg12529228	NHLH1	-0.11	3.83E-09
2811	cg22427279	NHLH2	-0.11	6.84E-13
2812	cg22881914	NID2	0.40	4.95E-17
2813	cg00739120	NIFIE14	0.23	1.70E-11
2814	cg00237010	NINJ2	0.16	4.10E-11
2815	cg15105703	NIP	0.30	1.32E-16
2816	cg10201668	NIP	0.17	3.61E-08
2817	cg10126923	NKG7	-0.17	1.30E-07
2818	cg10539507	NKX6-1	0.12	2.05E-04
2819	cg09260089	NKX6-2	0.49	3.79E-25
2820	cg08441806	NKX6-2	0.38	2.85E-22
2821	cg15660077	NLGN2	0.12	6.87E-06
2822	cg08109815	NMBR	0.29	5.70E-19
2823	cg18676162	NME4	0.11	7.22E-03
2824	cg13707560	NME5	0.20	9.42E-07
2825	cg18250832	NMUR1	0.13	1.49E-15
2826	cg03914397	NMUR2	-0.28	7.58E-28
2827	cg02196655	NOL10	0.13	6.34E-04
2828	cg06722216	NOL4	0.11	4.06E-05
2829	cg00273124	NOLA2	-0.14	1.98E-05
2830	cg23896545	NOMO1	0.15	2.59E-09
2831	cg21006686	NOS1	-0.18	3.43E-13
2832	cg03842617	NOS3	-0.20	2.62E-10
2833	cg25007250	NOS3	-0.25	1.41E-16
2834	cg17498321	NOTCH3	0.13	1.85E-04
2835	cg14700707	NOTCH4	-0.15	2.29E-06
2836	cg05973262	NOTCH4	-0.22	9.39E-11
2837	cg18488855	NOVA1	0.13	7.75E-06
2838	cg21461100	NOVA2	0.14	1.80E-05
2839	cg17063929	NOX4	0.10	9.92E-03
2840	cg04837071	NOXA1	-0.14	1.26E-07
2841	cg09628601	NPAS1	0.10	1.99E-06
2842	cg22134325	NPAS4	0.25	4.17E-15
2843	cg23186643	NPB	0.12	2.16E-03
2844	cg21628553	NPBWR2	-0.20	4.17E-11
2845	cg06255227	NPBWR2	-0.27	6.71E-15

2846	cg26581729	NPDC1	-0.15	6.80E-06
2847	cg07792737	NPIP	-0.12	2.33E-20
2848	cg05839235	NPR3	0.28	2.15E-10
2849	cg00548268	NPTX2	0.28	1.58E-15
2850	cg12799895	NPTX2	0.20	1.15E-07
2851	cg05158615	NPY	0.39	6.09E-22
2852	cg12614105	NPY	0.20	2.79E-09
2853	cg18438777	NPY5R	0.11	7.21E-04
2854	cg19194454	NQO1	0.13	2.85E-03
2855	cg06650260	NR0B2	-0.11	1.39E-04
2856	cg24580782	NR0B2	-0.11	3.42E-04
2857	cg10055471	NR0B2	-0.13	1.08E-05
2858	cg12613344	NR1H3	0.20	1.46E-16
2859	cg00554250	NR1H3	0.11	1.85E-06
2860	cg06277277	NR1I3	-0.13	4.21E-10
2861	cg25214346	NR1I3	-0.16	3.62E-11
2862	cg03958979	NR2E1	0.36	2.41E-21
2863	cg19697981	NR2E1	0.22	4.74E-11
2864	cg07890954	NR2E3	-0.11	3.06E-08
2865	cg18860847	NR2E3	-0.16	3.68E-14
2866	cg03533058	NR4A1	-0.13	8.99E-08
2867	cg05194726	NRIP2	0.15	3.50E-07
2868	cg00830029	NRIP2	0.14	4.37E-05
2869	cg25511429	NRN1	0.29	8.60E-15
2870	cg22367989	NRP2	0.28	9.06E-13
2871	cg16279786	NRXN1	-0.10	6.52E-07
2872	cg02554564	NTF3	-0.11	1.11E-07
2873	cg04740359	NTF3	-0.13	1.11E-05
2874	cg08108641	NTNG2	-0.16	3.55E-08
2875	cg14384532	NTRK3	0.21	8.07E-10
2876	cg08888956	NTS	-0.15	5.74E-12
2877	cg14871138	NTSR1	-0.20	3.72E-11
2878	cg21252483	NUCB1	0.13	1.48E-16
2879	cg21435394	NUDT14	0.11	1.07E-04
2880	cg04322134	NUDT2	-0.12	6.52E-21
2881	cg13285447	NUMBL	0.16	4.01E-04
2882	cg15827031	NUP155	0.20	3.30E-08
2883	cg07986773	NUP50	-0.15	3.22E-05
2884	cg08532057	NUPL1	0.15	4.46E-06
2885	cg07586911	NYD-SP18	-0.16	7.42E-09
2886	cg22182945	NYD-SP26	-0.17	1.62E-10
2887	cg16418329	NYD-SP26	-0.20	9.15E-12
2888	cg26845838	OACT2	0.30	1.79E-11
2889	cg03853151	OAT	0.20	2.78E-05
2890	cg15092802	OBP2A	-0.22	2.62E-18
2891	cg08539093	OBP2B	-0.22	9.34E-18
2892	cg15538820	OBP2B	-0.32	3.12E-20
2893	cg12902039	OCA2	0.16	3.13E-07
2894	cg22152667	OCM	-0.18	1.33E-11
2895	cg10335112	ODF3	-0.23	7.12E-24

2896	cg16225091	ODF3	-0.28	1.28E-19
2897	cg22168676	OFCC1	-0.15	2.62E-09
2898	cg07408740	OGDH	-0.11	3.41E-06
2899	cg21387281	OGFR	0.12	3.72E-12
2900	cg08268099	OLFM1	-0.24	6.35E-20
2901	cg00208967	OLFM2	0.23	2.82E-15
2902	cg10246520	OLFM4	-0.28	6.89E-18
2903	cg18506679	OLFML2A	-0.16	1.31E-12
2904	cg25853078	OPCML	-0.10	1.73E-07
2905	cg09261262	OPN4	-0.20	3.39E-19
2906	cg19869139	OPRD1	-0.16	2.00E-08
2907	cg09970593	OPRD1	-0.19	1.41E-13
2908	cg05657520	OPTC	-0.18	5.33E-10
2909	cg22951794	OR10A5	-0.35	3.05E-22
2910	cg22593486	OR10H1	-0.14	1.12E-12
2911	cg12513379	OR10H2	-0.11	4.73E-14
2912	cg24926780	OR10H2	-0.14	5.79E-13
2913	cg25843439	OR10H3	-0.14	6.04E-11
2914	cg04737405	OR12D2	-0.11	2.82E-11
2915	cg21414251	OR12D2	-0.17	8.03E-19
2916	cg03608577	OR12D3	-0.25	4.27E-14
2917	cg20856834	OR12D3	-0.25	4.20E-19
2918	cg16678925	OR1A2	-0.14	3.93E-15
2919	cg02721374	OR1D2	-0.18	2.62E-16
2920	cg22510822	OR1E2	-0.12	3.13E-07
2921	cg07879977	OR1F1	-0.14	2.76E-05
2922	cg27622610	OR1G1	-0.20	8.74E-14
2923	cg14598387	OR1N1	-0.13	6.74E-12
2924	cg16473288	OR1N1	-0.14	3.08E-10
2925	cg17026542	OR2A4	-0.12	1.32E-07
2926	cg24264578	OR2C1	-0.12	1.08E-10
2927	cg13397379	OR2C3	-0.32	4.20E-22
2928	cg08634024	OR2F1	-0.26	1.83E-16
2929	cg09931793	OR2K2	-0.15	7.09E-10
2930	cg27341860	OR2L13	-0.25	4.89E-16
2931	cg05071677	OR2V2	-0.15	5.92E-10
2932	cg11360149	OR2V2	-0.28	1.93E-14
2933	cg14014613	OR3A1	-0.21	4.27E-14
2934	cg05674036	OR3A3	-0.14	3.75E-10
2935	cg06353345	OR51B4	-0.18	8.51E-21
2936	cg25890048	OR5I1	-0.21	1.21E-17
2937	cg13410437	OR5P2	-0.31	2.14E-20
2938	cg01469547	OR5P3	-0.34	9.91E-23
2939	cg14258236	OR5V1	-0.19	6.35E-10
2940	cg24076830	OR5V1	-0.20	2.02E-12
2941	cg05112299	OR7A17	-0.14	2.58E-19
2942	cg02124291	OR7A5	-0.18	1.39E-12
2943	cg06639544	OR7A5	-0.20	1.27E-16
2944	cg24992780	OR7C1	-0.16	1.79E-10
2945	cg03544379	OR7C2	-0.24	6.36E-22

2946	cg14620221	OR8B8	-0.23	3.07E-12
2947	cg16612699	OR8B8	-0.25	3.05E-13
2948	cg00969271	ORM2	-0.13	1.01E-05
2949	cg16408565	ORM2	-0.18	5.71E-07
2950	cg07873128	OSBPL5	-0.11	5.76E-09
2951	cg06131936	OSBPL5	-0.12	8.40E-08
2952	cg11219178	OSBPL5	-0.15	6.23E-10
2953	cg12514506	OSBPL5	-0.16	8.98E-08
2954	cg25852715	OSBPL5	-0.17	1.72E-11
2955	cg21098323	OSCAR	0.14	5.39E-10
2956	cg16029760	OSTbeta	0.32	1.11E-19
2957	cg26900154	OTOF	-0.11	3.96E-05
2958	cg27305303	OTOF	-0.26	1.59E-19
2959	cg09222115	OTOS	-0.12	2.53E-06
2960	cg18790143	OTOS	-0.20	2.18E-09
2961	cg01421985	OTUD7	-0.13	4.39E-19
2962	cg09558502	OVGP1	-0.15	6.23E-15
2963	cg20909686	OVOL1	0.37	8.69E-20
2964	cg13496736	OVOL1	0.32	2.70E-13
2965	cg20315951	OVOL1	0.19	4.97E-09
2966	cg03681481	OVOL1	0.10	1.34E-04
2967	cg08590939	OXCT1	0.38	5.30E-23
2968	cg11487705	OXCT1	0.30	2.35E-11
2969	cg04144788	OXCT2	0.16	6.03E-06
2970	cg26955850	OXT	0.13	8.08E-05
2971	cg23391006	OXTR	0.12	9.61E-06
2972	cg26697117	P2RX1	-0.11	3.41E-07
2973	cg10287137	P2RY2	-0.16	3.60E-07
2974	cg06637774	P2RY6	0.13	4.51E-04
2975	cg15903837	P2RY6	-0.11	1.18E-10
2976	cg05671350	P4HA3	0.18	1.54E-08
2977	cg03924115	P518	0.11	2.47E-12
2978	cg25022327	P53AIP1	-0.22	8.86E-12
2979	cg00094319	PABPC3	-0.14	4.65E-10
2980	cg23043245	PACSIN1	0.26	2.32E-14
2981	cg23239444	PACSIN1	0.17	2.65E-06
2982	cg17502536	PADI1	-0.19	2.36E-10
2983	cg22193702	PADI1	-0.25	7.30E-15
2984	cg12858514	PADI3	-0.15	3.88E-09
2985	cg01459162	PADI3	-0.19	9.33E-13
2986	cg03264414	PAEP	-0.23	5.66E-14
2987	cg01055695	PAEP	-0.36	5.27E-24
2988	cg06958211	PAK6	-0.11	1.62E-09
2989	cg11896271	PANX2	0.14	9.55E-04
2990	cg08191915	PANX3	-0.16	9.19E-10
2991	cg03885639	PANX3	-0.17	2.09E-14
2992	cg10994126	PAPPA2	-0.15	8.30E-10
2993	cg09191232	PAPSS1	0.15	2.17E-08
2994	cg13644052	PAQR4	-0.24	3.02E-16
2995	cg13117105	PAQR5	0.10	1.42E-03

2996	cg24323031	PAQR8	0.14	9.24E-05
2997	cg26301689	PAQR9	-0.19	1.90E-09
2998	cg15426734	PARD6A	0.27	7.74E-15
2999	cg07937272	PARP12	0.14	5.41E-09
3000	cg09712066	PART1	-0.13	2.37E-06
3001	cg26861460	PARVG	-0.15	8.81E-13
3002	cg19863740	PARVG	-0.18	7.08E-14
3003	cg19345602	PATE	-0.11	9.18E-13
3004	cg10377274	PATE	-0.32	1.29E-23
3005	cg24401441	PAX1	-0.13	3.85E-10
3006	cg08886154	PAX4	-0.26	9.45E-30
3007	cg16514843	PAX4	-0.29	3.07E-23
3008	cg25242557	PAX6	0.12	7.07E-04
3009	cg00509670	PAX9	0.13	4.76E-05
3010	cg26620157	PAX9	0.12	5.14E-06
3011	cg19996355	PBX4	0.37	2.87E-21
3012	cg23158811	PBX4	0.30	2.29E-16
3013	cg07685034	PCBP3	-0.14	6.68E-12
3014	cg23272214	PCBP3	-0.25	8.01E-17
3015	cg04797496	PCDH12	-0.15	6.48E-15
3016	cg20366906	PCDH8	0.26	2.17E-13
3017	cg20514617	PCDHA9	-0.12	1.46E-08
3018	cg12629325	PCDHAC1	0.30	1.88E-10
3019	cg18902090	PCDHAC1	0.19	9.97E-07
3020	cg07899016	PCDHB12	-0.11	2.62E-09
3021	cg11368643	PCDHB15	0.11	6.22E-04
3022	cg02260587	PCDHB2	0.10	1.18E-05
3023	cg11647681	PCDHGA12	0.29	8.13E-17
3024	cg07730329	PCDHGA12	0.29	2.64E-16
3025	cg00911351	PCDHGB4	0.14	1.95E-06
3026	cg23563234	PCDHGB7	0.40	4.71E-24
3027	cg14011639	PCDHGB7	0.26	2.84E-15
3028	cg15361590	PCDHGC4	0.28	1.85E-13
3029	cg12145907	PCDHGC4	0.13	1.78E-07
3030	cg17108383	PCDHGC5	-0.10	1.54E-04
3031	cg00943245	PCDHGC5	-0.12	1.09E-06
3032	cg16101800	PCK1	-0.11	1.78E-05
3033	cg13662243	PCMT1	-0.12	5.87E-10
3034	cg26777475	PCOLCE	-0.10	3.96E-05
3035	cg26563737	PCOLN3	-0.19	3.21E-14
3036	cg02910574	PCOLN3	-0.23	3.27E-14
3037	cg00895324	PCP4	-0.25	1.10E-25
3038	cg09786257	PCSK1	0.27	5.52E-26
3039	cg23187653	PCSK1	0.21	1.33E-10
3040	cg20249919	PCSK6	-0.12	1.52E-05
3041	cg18096388	PDCD1	-0.14	2.09E-15
3042	cg00795812	PDCD1	-0.26	2.47E-12
3043	cg24133115	PDE10A	0.17	1.60E-05
3044	cg01431114	PDE10A	0.12	1.31E-03
3045	cg13899108	PDE4C	0.19	3.18E-18

3046	cg17861230	PDE4C	0.17	7.40E-06
3047	cg11935147	PDE4DIP	0.35	5.28E-33
3048	cg18915143	PDE6A	-0.11	1.46E-06
3049	cg05078019	PDE6B	-0.14	3.06E-05
3050	cg12435792	PDE6B	-0.21	2.01E-10
3051	cg12572827	PDE6H	-0.16	5.53E-12
3052	cg09447105	PDE6H	-0.27	1.03E-17
3053	cg00516481	PDE9A	0.11	9.46E-04
3054	cg12727795	PDGFRB	0.19	3.13E-11
3055	cg27108154	PDHA2	-0.25	3.06E-15
3056	cg17725968	PDHA2	-0.31	6.86E-23
3057	cg22171829	PDK4	-0.11	4.15E-08
3058	cg14444710	PDPK1	-0.22	4.84E-16
3059	cg14944362	PDYN	-0.13	1.40E-09
3060	cg11245384	PDYN	-0.16	3.14E-08
3061	cg13019092	PDZK1	-0.15	1.53E-10
3062	cg10321723	PDZK1	-0.18	6.58E-12
3063	cg22705225	PDZK3	0.11	1.17E-04
3064	cg14473924	PDZRN3	0.13	1.53E-04
3065	cg20352371	PDZRN4	-0.18	3.17E-11
3066	cg15417244	PDZRN4	-0.25	6.27E-16
3067	cg21099488	PELI2	0.10	3.56E-04
3068	cg04598121	PENK	0.19	5.72E-09
3069	cg06546607	PEPD	0.14	3.63E-06
3070	cg25514503	PER3	-0.10	1.32E-04
3071	cg27446233	PFKM	-0.11	3.74E-06
3072	cg08465862	PFKP	0.24	5.06E-10
3073	cg10247268	PFKP	0.19	2.14E-08
3074	cg19890739	Pfs2	0.11	4.47E-06
3075	cg11957986	PGA5	-0.15	7.66E-11
3076	cg09750183	PGA5	-0.20	2.02E-14
3077	cg26057752	PGAM2	0.16	1.46E-05
3078	cg17391877	PGC	-0.12	3.98E-07
3079	cg12855851	PGC	-0.15	4.12E-07
3080	cg01892689	PGCP	-0.18	3.05E-14
3081	cg12841020	PGF	0.10	1.75E-03
3082	cg09299388	PGK2	-0.26	7.50E-16
3083	cg02806777	PGLYRP1	0.20	4.49E-10
3084	cg06275635	PGLYRP3	-0.18	1.21E-12
3085	cg09448880	PGLYRP3	-0.30	9.98E-25
3086	cg11442717	PHF10	0.10	7.74E-04
3087	cg01775414	PHF21B	0.15	1.32E-05
3088	cg19759064	PHKG1	-0.11	5.33E-05
3089	cg18722841	PHOX2A	0.16	7.64E-07
3090	cg08876932	PHOX2A	0.10	2.72E-05
3091	cg04893119	PI15	-0.10	2.32E-04
3092	cg02442161	PI3	-0.22	4.31E-20
3093	cg11635563	PIG38	-0.14	8.26E-09
3094	cg23166362	PIK3CD	0.12	3.78E-06
3095	cg13453139	PIK3R5	-0.25	3.03E-13

3096	cg09528351	PIK3R5	-0.25	8.20E-15
3097	cg15542496	PIP	-0.11	1.64E-05
3098	cg26628847	PIP	-0.16	5.26E-10
3099	cg19690214	PIP3-E	-0.14	2.59E-12
3100	cg10082841	PIP5KL1	0.12	1.45E-04
3101	cg17599586	PISD	0.23	5.18E-05
3102	cg16759954	PITPNM3	-0.12	8.81E-08
3103	cg24495017	PITX1	0.25	7.55E-11
3104	cg14681055	PITX3	0.30	1.23E-10
3105	cg22882178	PITX3	0.23	1.33E-08
3106	cg10365880	PKD2L1	-0.19	2.95E-11
3107	cg11377136	PKDREJ	0.46	1.19E-35
3108	cg02280309	PKLR	-0.16	1.12E-12
3109	cg02510853	PKMYT1	0.22	1.04E-20
3110	cg02044879	PLA2G12B	-0.13	6.76E-06
3111	cg21820890	PLA2G12B	-0.16	3.72E-07
3112	cg22220722	PLA2G2A	-0.17	1.86E-09
3113	cg11037787	PLA2G2A	-0.20	4.18E-14
3114	cg14321743	PLA2G2D	-0.21	1.99E-13
3115	cg07142319	PLA2G2D	-0.23	4.15E-15
3116	cg16825000	PLA2G2E	-0.19	7.43E-14
3117	cg16087263	PLA2G2F	-0.13	7.84E-10
3118	cg11933267	PLA2G2F	-0.18	4.95E-11
3119	cg21274025	PLA2G3	-0.18	2.27E-08
3120	cg15228639	PLA2G4E	-0.16	8.48E-14
3121	cg18630040	PLA2G7	0.31	2.20E-10
3122	cg04367351	PLA2R1	0.11	8.03E-07
3123	cg16483916	PLAC2	0.26	1.97E-11
3124	cg04993257	PLAC2	0.24	2.48E-14
3125	cg12091331	PLAT	0.15	4.33E-07
3126	cg06521280	PLAU	0.21	3.29E-12
3127	cg15120942	PLCD1	0.18	7.67E-08
3128	cg09480837	PLCE1	-0.13	2.90E-04
3129	cg21522797	PLCG2	-0.13	2.62E-09
3130	cg02833180	PLCL1	-0.20	6.16E-14
3131	cg25573386	PLCZ1	-0.13	9.52E-08
3132	cg12613383	PLD5	0.15	8.29E-06
3133	cg17641252	PLEK2	0.13	1.13E-05
3134	cg20141916	PLEK2	0.11	6.46E-04
3135	cg21581873	PLEKHA6	0.18	1.23E-10
3136	cg27549944	PLEKHA6	0.16	8.42E-11
3137	cg25288155	PLEKHB1	-0.13	1.52E-05
3138	cg02254407	PLEKHB1	-0.20	9.42E-09
3139	cg12072973	PLEKHO1	0.26	2.26E-07
3140	cg15083227	PLEKHQ1	0.13	9.75E-06
3141	cg14003512	PLGLB2	-0.13	9.18E-13
3142	cg06240124	PLK1	0.12	3.94E-03
3143	cg25527547	PLOD3	-0.13	1.91E-07
3144	cg26220350	PLTP	0.25	2.69E-11
3145	cg11846968	PLUNC	-0.18	9.99E-17

3146	cg14601284	PLXDC1	0.13	2.17E-05
3147	cg06084117	PLXNA4B	-0.27	2.61E-16
3148	cg17356112	PLXNA4B	-0.28	2.32E-19
3149	cg09837648	PLXNB1	0.17	2.04E-10
3150	cg24770850	PLXND1	0.21	2.12E-07
3151	cg13415207	PMAIP1	0.28	6.02E-12
3152	cg12530080	PMCHL1	-0.11	3.96E-10
3153	cg03242666	PMP22	-0.13	2.43E-09
3154	cg11812202	PNLIP	-0.21	7.00E-15
3155	cg27600794	PNLIPRP1	-0.20	4.99E-14
3156	cg11310496	PNLIPRP2	-0.11	2.13E-07
3157	cg16272420	PNLIPRP2	-0.26	2.48E-20
3158	cg23681213	PNMA1	0.21	4.71E-08
3159	cg02154186	PNMA2	0.33	6.43E-14
3160	cg12894984	PNMT	-0.11	1.39E-04
3161	cg19391527	PNOC	-0.11	5.12E-09
3162	cg23092823	PODN	0.29	1.24E-18
3163	cg16028753	PODN	0.17	5.89E-10
3164	cg04739570	PODXL2	0.25	3.00E-14
3165	cg19593572	POLR3E	0.14	4.49E-09
3166	cg16302441	POMC	0.13	5.65E-07
3167	cg16739580	POP2	-0.13	6.35E-09
3168	cg19363546	POU2F3	0.16	4.12E-06
3169	cg21243096	POU3F1	0.39	4.91E-24
3170	cg17791651	POU3F1	0.27	2.63E-12
3171	cg20291049	POU3F3	0.34	1.82E-17
3172	cg08097882	POU4F1	0.46	1.25E-20
3173	cg15604467	POU4F1	0.26	3.48E-11
3174	cg24199834	POU4F2	0.31	6.84E-17
3175	cg13262687	POU4F2	0.17	1.68E-09
3176	cg03251857	PPAPDC3	-0.19	5.63E-10
3177	cg20357806	PPBP	-0.14	7.76E-09
3178	cg14724265	PPEF2	-0.12	2.41E-09
3179	cg24920358	PPIE	0.15	6.33E-05
3180	cg09350141	PPM1F	0.14	6.82E-05
3181	cg20276750	PPM1M	0.15	3.87E-04
3182	cg24834740	PPP1R16B	0.16	4.77E-06
3183	cg27377213	PPP1R16B	0.11	1.10E-05
3184	cg19280968	PPP1R2	0.23	1.40E-07
3185	cg11164400	PPP1R9A	0.20	5.26E-08
3186	cg16872560	PPP1R9A	0.14	6.59E-05
3187	cg19221959	PPP1R9A	0.11	1.31E-03
3188	cg07867360	PPP2R2C	-0.18	1.02E-08
3189	cg07038400	PPP2R3A	0.31	8.36E-25
3190	cg15765694	PPP3R2	-0.14	6.31E-11
3191	cg02602411	PPP3R2	-0.22	6.90E-14
3192	cg12585943	PPT2	0.29	6.88E-19
3193	cg17329164	PPT2	0.24	9.20E-14
3194	cg01543654	PRAC	0.23	1.16E-13
3195	cg12374721	PRAC	0.15	8.51E-04

3196	cg05208878	PRAME	-0.18	1.52E-13
3197	cg18807515	PRAMEF2	-0.11	1.32E-06
3198	cg27345534	PRB2	-0.12	3.74E-13
3199	cg04520391	PRB2	-0.18	2.51E-13
3200	cg22692158	PRB4	-0.15	3.52E-12
3201	cg14076161	PRB4	-0.22	8.80E-19
3202	cg09699159	PRDM11	-0.16	6.33E-12
3203	cg20227165	PRDM11	-0.28	3.19E-24
3204	cg01295203	PRDM14	0.41	7.25E-22
3205	cg08528984	PRDM16	0.12	6.39E-04
3206	cg12379145	PRDM2	-0.16	2.66E-14
3207	cg25402049	PRDM2	-0.18	1.20E-12
3208	cg19324313	PRDM2	-0.20	8.84E-16
3209	cg02374486	PRF1	-0.11	3.21E-06
3210	cg09914304	PRF1	-0.14	1.44E-07
3211	cg17342283	PRG1	-0.11	5.50E-05
3212	cg20684549	PRG2	0.12	1.30E-04
3213	cg15640375	PRG2	0.11	8.38E-04
3214	cg15357945	PRG2	-0.21	2.99E-17
3215	cg24653967	PRH1	0.17	1.99E-11
3216	cg14502651	PRH2	0.14	8.28E-08
3217	cg12493160	PRKACG	-0.16	2.94E-09
3218	cg13577076	PRKAR1B	0.33	9.28E-18
3219	cg13705707	PRKAR2B	0.12	1.13E-05
3220	cg05436658	PRKCB1	0.33	3.53E-13
3221	cg24250393	PRKCB1	0.12	4.10E-05
3222	cg18959478	PRKCDBP	0.12	3.29E-04
3223	cg18392783	PRKCDBP	0.11	1.27E-04
3224	cg02282237	PRKCH	0.14	4.97E-07
3225	cg21794225	PRKD1	0.13	1.95E-04
3226	cg09019938	PRKG1	0.11	4.22E-04
3227	cg16744741	PRKG2	-0.31	7.94E-27
3228	cg09906458	PRND	-0.11	4.01E-09
3229	cg09425898	PRNT	-0.21	1.60E-10
3230	cg26718585	PROC	-0.12	6.90E-06
3231	cg19462352	PROCA1	0.12	8.96E-05
3232	cg26806924	PROCR	0.19	2.04E-09
3233	cg08555612	PROK2	0.11	7.63E-04
3234	cg24626923	PROKR1	-0.13	1.06E-09
3235	cg20576510	PROM1	-0.18	6.50E-10
3236	cg00930194	PROP1	-0.12	2.53E-10
3237	cg21478490	PROP1	-0.17	4.88E-11
3238	cg25734864	PROZ	-0.13	9.45E-06
3239	cg09595479	PRPH	0.21	5.73E-07
3240	cg24059075	PRPH	-0.12	2.90E-09
3241	cg00911873	PRPS1L1	-0.17	4.77E-12
3242	cg21264055	PRR3	0.19	2.39E-07
3243	cg04273431	PRR3	0.11	2.88E-05
3244	cg23240895	PRR5	-0.19	9.47E-08
3245	cg19769182	PRRT2	0.16	1.68E-06

3246	cg12878228	PRSS1	-0.21	8.57E-21
3247	cg26504906	PRSS16	-0.20	1.05E-10
3248	cg04958389	PRSS2	-0.30	9.15E-23
3249	cg23214764	PRSS23	0.12	1.59E-04
3250	cg13439730	PRSS8	0.10	4.18E-04
3251	cg07073964	PRSSL1	-0.21	4.86E-13
3252	cg09134726	PRTN3	-0.17	4.81E-10
3253	cg20281815	PSCA	-0.17	1.28E-10
3254	cg13446199	PSCA	-0.22	3.04E-14
3255	cg13548361	PSD	0.12	2.84E-07
3256	cg08093398	PSF1	-0.10	2.09E-08
3257	cg25477904	PSG1	-0.11	1.67E-13
3258	cg25839766	PSG1	-0.34	2.11E-26
3259	cg04127255	PSG2	-0.14	1.07E-11
3260	cg07745725	PSG3	-0.13	6.79E-14
3261	cg13434203	PSG3	-0.17	1.93E-16
3262	cg23264413	PSG4	-0.12	2.03E-11
3263	cg01078689	PSG5	-0.10	4.23E-16
3264	cg15586352	PSG5	-0.15	1.39E-16
3265	cg11151665	PSG6	-0.10	2.86E-13
3266	cg23610820	PSG9	-0.11	3.77E-15
3267	cg16890093	PSMB8	0.10	6.04E-05
3268	cg12703269	PSTPIP1	-0.16	1.01E-11
3269	cg24989962	PTGDR	0.29	2.03E-12
3270	cg09516965	PTGDR	0.11	5.83E-03
3271	cg00563932	PTGDS	-0.17	1.65E-10
3272	cg06738602	PTGER2	0.23	7.68E-09
3273	cg25823578	PTGER2	0.17	4.34E-06
3274	cg26672426	PTGES	-0.14	4.09E-06
3275	cg17683775	PTGES	-0.16	3.51E-08
3276	cg03752628	PTGFRN	-0.12	1.21E-08
3277	cg13986130	PTGS2	0.19	2.08E-11
3278	cg15774283	PTGS2	0.12	1.43E-05
3279	cg24816298	PTH	-0.16	3.63E-12
3280	cg25057743	PTHR2	0.34	1.32E-16
3281	cg08747889	PTK7	0.32	1.12E-12
3282	cg21663580	PTK7	0.21	9.48E-11
3283	cg18675600	PTP4A3	-0.19	4.04E-13
3284	cg23868119	PTPLA	0.13	6.55E-05
3285	cg02222362	PTPN20B	0.12	4.84E-05
3286	cg22335340	PTPN6	0.11	1.13E-05
3287	cg04956511	PTPN6	-0.10	3.13E-07
3288	cg09440243	PTPRD	-0.14	1.22E-08
3289	cg14859417	PTPRE	-0.12	4.48E-04
3290	cg06971096	PTPRN	0.16	2.58E-09
3291	cg18601426	PTPRN2	0.24	4.62E-14
3292	cg05119831	PTPRN2	0.11	3.99E-06
3293	cg10646402	PTPRO	0.14	7.32E-06
3294	cg11843304	PTPRO	-0.15	3.80E-17
3295	cg00614413	PTPRS	-0.15	3.82E-22

3296	cg27299588	PTPRS	-0.22	2.16E-11
3297	cg16003238	PUNC	0.10	3.40E-04
3298	cg09863066	PVALB	-0.13	5.70E-15
3299	cg02978737	PVALB	-0.23	5.25E-16
3300	cg22580353	PVR	-0.15	2.11E-12
3301	cg10832166	PWCR1	-0.11	8.07E-08
3302	cg13994177	PWCR1	-0.17	7.11E-13
3303	cg26389232	PWCR1	-0.17	2.46E-16
3304	cg14037665	PXDN	0.18	4.17E-06
3305	cg12164282	PXDN	0.14	1.73E-03
3306	cg09587549	PYCARD	0.26	2.47E-08
3307	cg05361373	PYCARD	0.21	2.59E-07
3308	cg15247144	PYCARD	0.18	4.81E-07
3309	cg12100791	PYCARD	0.11	1.03E-02
3310	cg19202384	PYCR1	-0.15	6.66E-10
3311	cg19850348	PYGO1	0.27	4.66E-12
3312	cg23412777	PYGO1	-0.14	2.28E-06
3313	cg16029957	QPCT	0.14	3.35E-05
3314	cg08812504	QSCN6	0.24	5.10E-08
3315	cg08755040	QSCN6	0.14	3.12E-07
3316	cg23018448	QTRT1	-0.13	2.68E-09
3317	cg01255591	R3HDML	-0.20	8.39E-13
3318	cg10149836	RAB11FIP4	0.21	3.85E-10
3319	cg04764624	RAB11FIP4	0.20	2.41E-09
3320	cg26029902	RAB22A	0.30	1.96E-18
3321	cg00848461	RAB24	-0.15	3.39E-09
3322	cg09243900	RAB25	0.17	1.38E-20
3323	cg19580810	RAB25	0.13	1.09E-15
3324	cg17982102	RAB31	0.36	3.68E-18
3325	cg04274487	RAB31	0.15	2.26E-07
3326	cg21237418	RAB34	0.13	4.10E-04
3327	cg15379633	RAB36	0.30	1.16E-18
3328	cg13894021	RAB36	0.12	7.29E-09
3329	cg12448933	RAB37	0.19	7.69E-08
3330	cg05090725	RAB39	0.26	6.30E-09
3331	cg08179907	RAB39	0.16	2.41E-07
3332	cg05554936	RAB3D	0.40	6.88E-14
3333	cg20780953	RAB3D	0.25	9.47E-08
3334	cg06646021	RAB4A	-0.12	2.05E-06
3335	cg22373112	RAB5C	0.18	3.94E-11
3336	cg11513856	RABL5	-0.12	3.52E-12
3337	cg18265887	RAC2	0.16	1.17E-06
3338	cg03262773	RAD54L	0.13	1.72E-03
3339	cg00278366	RAD9B	-0.15	1.86E-05
3340	cg11494699	RAG1	-0.14	1.60E-09
3341	cg15982419	RALBP1	0.15	9.46E-06
3342	cg18410627	RALGPS1	0.30	8.08E-15
3343	cg08433538	RALGPS1	0.10	2.09E-03
3344	cg14436761	RAMP2	0.10	1.10E-04
3345	cg02417264	RAMP3	-0.12	1.29E-09

3346	cg02902770	RARRES2	-0.12	1.14E-08
3347	cg10274908	RASA4	0.15	6.81E-05
3348	cg05749161	RASA4	0.15	8.45E-05
3349	cg10319505	RASEF	0.22	1.17E-06
3350	cg16154416	RASGRF1	0.19	4.71E-08
3351	cg15156078	RASGRF1	0.14	1.55E-05
3352	cg09952204	RASGRF2	0.35	1.21E-14
3353	cg14170423	RASGRP2	0.14	1.05E-07
3354	cg01109219	RASGRP3	-0.11	3.98E-06
3355	cg26750002	RASIP1	0.11	1.85E-04
3356	cg10604168	RASL12	0.33	5.55E-20
3357	cg21554552	RASSF1	0.24	3.08E-17
3358	cg00777121	RASSF1	0.15	4.42E-12
3359	cg08047457	RASSF1	0.15	2.65E-11
3360	cg19614321	RASSF2	0.15	1.27E-06
3361	cg08617916	RASSF5	0.16	3.02E-04
3362	cg02589695	RASSF5	0.15	2.20E-04
3363	cg01860753	RASSF5	0.14	6.19E-04
3364	cg17558126	RASSF5	0.13	5.88E-04
3365	cg25058957	RAXL1	-0.22	2.71E-16
3366	cg25002911	RB1	-0.14	8.28E-27
3367	cg06914598	RBAK	0.26	2.75E-26
3368	cg15821095	RBM15B	-0.24	6.10E-16
3369	cg18565355	RBM35A	0.18	2.62E-07
3370	cg14916213	RBM4	0.12	1.09E-03
3371	cg25488206	RBM9	0.15	3.27E-09
3372	cg00615377	RBM9	0.15	5.33E-09
3373	cg23363832	RBP1	0.28	2.43E-10
3374	cg13099330	RBP1	0.25	8.62E-10
3375	cg06543018	RBP1	0.19	1.46E-06
3376	cg27457941	RBP1	0.16	2.32E-06
3377	cg12497564	RBP1	0.13	3.11E-03
3378	cg24594997	RBP1	0.13	4.42E-05
3379	cg11027570	RBP1	0.12	2.03E-03
3380	cg24053375	RBP3	-0.22	1.99E-13
3381	cg17964955	RBP3	-0.24	1.48E-19
3382	cg24441911	RBP5	-0.16	3.55E-07
3383	cg04378886	RCN3	0.12	3.83E-09
3384	cg04720358	RCV1	-0.11	5.27E-09
3385	cg24357161	RCV1	-0.23	3.75E-14
3386	cg25661884	RDH8	-0.15	5.79E-10
3387	cg18140857	RDHE2	-0.26	1.14E-19
3388	cg15169162	RDS	-0.16	9.98E-14
3389	cg12717594	RECK	0.19	4.23E-06
3390	cg27264345	REEP2	0.12	3.91E-05
3391	cg05828624	REG1A	-0.22	6.21E-19
3392	cg07841014	REG1B	-0.30	1.69E-22
3393	cg27342801	REG3A	-0.21	1.54E-11
3394	cg00918005	REG3G	-0.23	1.71E-18
3395	cg16158220	REGL	-0.17	1.72E-11

3396	cg02868123	REGL	-0.30	6.00E-22
3397	cg05556020	REN	-0.15	1.06E-09
3398	cg19205533	RERG	0.22	1.71E-14
3399	cg03028472	RERG	0.12	2.45E-04
3400	cg20692181	RETN	-0.12	2.81E-13
3401	cg14659547	RETNLB	-0.31	8.51E-22
3402	cg13789236	RFK	0.11	1.13E-09
3403	cg22825979	RFP2	0.15	1.30E-04
3404	cg15303841	RFPL1	-0.21	3.73E-17
3405	cg00406844	RFPL3	-0.22	8.49E-16
3406	cg13614181	RGC32	0.27	1.18E-15
3407	cg02148642	RGPD5	-0.20	7.84E-19
3408	cg24660086	RGR	-0.21	7.39E-19
3409	cg04041960	RGS10	0.40	3.95E-20
3410	cg19653161	RGS10	0.23	1.42E-08
3411	cg24317255	RGS17	0.19	4.81E-09
3412	cg20315136	RGS2	0.11	2.19E-03
3413	cg21303386	RGS7	0.17	5.40E-08
3414	cg25020850	RGS8	-0.22	9.18E-14
3415	cg15595739	RGS9	-0.14	3.68E-07
3416	cg19862344	RHAG	-0.15	4.25E-09
3417	cg10523019	RHBDD1	0.13	3.30E-04
3418	cg24653181	RHCG	0.22	9.20E-08
3419	cg10453365	RHCG	0.12	4.68E-04
3420	cg03998173	RHEB	0.11	2.47E-03
3421	cg13180098	RHO	-0.13	6.12E-09
3422	cg09088508	RHOF	0.16	5.97E-06
3423	cg00804392	RHOH	0.13	1.51E-07
3424	cg18771300	RHOJ	-0.12	4.59E-07
3425	cg05135288	RHOT2	0.10	5.18E-04
3426	cg18180155	RIBC2	0.10	8.30E-04
3427	cg09847584	RIMBP2	-0.27	1.33E-19
3428	cg19332710	RIMS4	0.31	2.56E-09
3429	cg20822579	RIPK3	0.26	3.12E-16
3430	cg04711324	RIT2	-0.36	4.68E-21
3431	cg19287277	RKHD3	0.14	1.09E-04
3432	cg10408410	RLBP1	-0.12	8.82E-09
3433	cg00055233	RLN1	0.11	1.37E-06
3434	cg20789691	RLN1	-0.17	7.53E-11
3435	cg03679581	RLN2	0.11	1.02E-06
3436	cg05958352	RNASE1	-0.11	2.33E-04
3437	cg13718960	RNASE1	-0.17	4.88E-13
3438	cg26191951	RNASE2	-0.11	5.84E-11
3439	cg15910079	RNASE3	-0.11	8.36E-13
3440	cg07525077	RNASE3	-0.11	3.77E-11
3441	cg06720660	RNASE6	-0.16	3.87E-08
3442	cg05270634	RND2	0.27	5.14E-12
3443	cg00948524	RNF135	0.33	8.97E-17
3444	cg09754413	RNF182	0.15	2.04E-05
3445	cg04603184	RNF182	0.14	6.25E-07

3446	cg04994456	RNF186	-0.10	1.02E-03
3447	cg05439368	RNF36	0.12	3.92E-07
3448	cg25766046	ROR2	0.24	1.16E-09
3449	cg25112191	RORC	-0.18	1.85E-13
3450	cg26555310	RPE65	-0.12	6.66E-09
3451	cg11724759	RPE65	-0.15	9.58E-15
3452	cg13102585	RPIP8	0.32	1.69E-24
3453	cg07441272	RPL39L	0.30	7.36E-12
3454	cg07693270	RPL39L	0.18	3.17E-09
3455	cg18279742	RPS2	-0.14	1.12E-10
3456	cg19428417	RRAD	0.13	2.23E-07
3457	cg26709950	RRAD	0.12	2.15E-04
3458	cg25635500	RRP22	0.22	1.45E-08
3459	cg16612562	RRP22	0.19	5.45E-21
3460	cg01777397	RSNL2	0.15	9.74E-04
3461	cg05358404	RTEL1	0.13	2.29E-10
3462	cg00689340	RTKN	-0.16	2.15E-10
3463	cg08749917	RTP1	-0.12	4.10E-07
3464	cg26536164	RTP3	-0.16	3.06E-07
3465	cg06377278	RUNX3	0.47	1.83E-18
3466	cg00117172	RUNX3	0.35	8.94E-12
3467	cg22737001	RUNX3	0.24	2.96E-09
3468	cg14645650	RUNX3	-0.12	1.46E-09
3469	cg04757093	RUNX3	-0.13	9.93E-14
3470	cg18231267	RUNX3	-0.16	4.98E-10
3471	cg25178645	RUNX3	-0.16	9.70E-11
3472	cg27360282	RUNX3	-0.17	4.48E-11
3473	cg02723372	RUNX3	-0.21	6.50E-17
3474	cg14182690	RUNX3	-0.24	5.33E-13
3475	cg10364513	RXRG	0.20	7.68E-08
3476	cg19764418	RYR2	-0.14	5.48E-07
3477	cg20847746	S100A1	-0.16	1.07E-09
3478	cg10795646	S100A10	-0.10	1.04E-04
3479	cg02813121	S100A12	-0.36	7.10E-26
3480	cg06615154	S100A3	-0.20	6.72E-10
3481	cg07426848	S100A3	-0.30	1.75E-17
3482	cg17250929	S100A5	-0.19	1.21E-11
3483	cg24898863	S100A8	-0.11	5.83E-20
3484	cg20070090	S100A8	-0.21	1.09E-20
3485	cg07039113	S100A9	-0.16	3.68E-06
3486	cg07685786	S100B	-0.11	4.48E-06
3487	cg08205865	SAC	-0.12	4.60E-13
3488	cg08118311	SALL3	0.33	3.28E-14
3489	cg15191648	SALL3	0.27	3.25E-10
3490	cg06303238	SALL4	0.12	2.24E-05
3491	cg21122774	SARDH	-0.14	5.70E-08
3492	cg23887396	SARM1	0.26	2.32E-14
3493	cg09025324	SART2	0.28	3.17E-17
3494	cg17981339	SBEM	-0.12	5.36E-07
3495	cg27160701	SBEM	-0.13	3.58E-09

3496	cg23680518	SBSN	-0.21	1.41E-13
3497	cg27050793	SBSN	-0.23	3.17E-17
3498	cg17866455	SCAND2	-0.11	2.09E-07
3499	cg05106502	SCAP1	-0.19	1.54E-08
3500	cg14785479	SCARF2	0.17	9.37E-06
3501	cg22886089	SCG3	0.14	1.34E-08
3502	cg01772980	SCGB1D1	-0.23	3.78E-21
3503	cg13916742	SCGB1D1	-0.28	4.29E-19
3504	cg10848367	SCGB1D2	-0.12	2.36E-13
3505	cg26041297	SCGB1D2	-0.15	1.21E-20
3506	cg08459368	SCGB2A1	-0.24	1.96E-16
3507	cg14741922	SCGB2A2	-0.13	8.39E-09
3508	cg22862656	SCGB2A2	-0.24	5.52E-15
3509	cg14472601	SCGB3A1	-0.23	2.08E-12
3510	cg19530885	SCGB3A2	-0.16	3.41E-10
3511	cg16954341	SCGN	0.17	2.88E-08
3512	cg15457899	SCN3B	0.30	9.04E-14
3513	cg13765785	SCN3B	0.16	5.62E-07
3514	cg23722792	SCN4A	-0.12	4.17E-08
3515	cg13694749	SCN4A	-0.35	1.37E-20
3516	cg27560864	SCN4B	0.14	1.77E-07
3517	cg25995212	SCN7A	-0.20	7.21E-14
3518	cg26215727	SCNN1A	0.11	9.67E-07
3519	cg13365761	SCNN1G	0.17	8.44E-06
3520	cg01857260	SCRL	0.16	2.34E-08
3521	cg09697795	SCRL	0.12	5.58E-05
3522	cg10639440	SCRN1	0.22	1.60E-08
3523	cg01200060	SCRT2	0.18	3.12E-09
3524	cg15250797	SCTR	0.21	2.79E-10
3525	cg01897036	SCTR	-0.11	8.60E-05
3526	cg26219051	SCUBE1	0.28	1.38E-15
3527	cg01081263	SCUBE2	0.17	2.40E-07
3528	cg19237879	SCUBE2	0.16	1.19E-05
3529	cg00347904	SCUBE3	0.33	8.91E-16
3530	cg10876928	SDC4	-0.14	3.36E-09
3531	cg06352750	SDPR	0.12	1.89E-03
3532	cg09426307	SEC14L3	-0.11	1.05E-07
3533	cg20831708	SEC31L2	-0.13	6.82E-04
3534	cg02787991	SECTM1	0.45	2.20E-24
3535	cg10249734	SECTM1	0.18	1.18E-13
3536	cg24497819	SELPLG	0.11	2.55E-06
3537	cg08097657	SEMA3B	0.11	3.62E-07
3538	cg24816455	SEMA3B	-0.25	5.59E-18
3539	cg14911395	SEMA3B	-0.34	5.56E-23
3540	cg07640473	SEMA3F	0.21	2.38E-06
3541	cg22496652	SEMA4D	-0.11	1.62E-18
3542	cg03702236	SEMA5A	0.17	1.39E-05
3543	cg12928668	SEMA6A	0.12	1.00E-04
3544	cg14093936	SEMA7A	0.10	2.33E-06
3545	cg02311163	SEMG2	-0.19	6.80E-18

3546	cg03312792	SEMG2	-0.27	9.85E-22
3547	cg03135598	SEPW1	0.11	1.39E-03
3548	cg12078929	SERHL	0.40	4.35E-25
3549	cg03855656	SERHL	0.17	3.91E-13
3550	cg19663795	SEPT1	0.13	3.89E-06
3551	cg05788638	SERPINA10	-0.16	4.58E-08
3552	cg08495878	SERPINA4	-0.11	2.20E-04
3553	cg10025865	SERPINA6	-0.10	8.25E-05
3554	cg11435943	SERPINB12	-0.19	1.42E-18
3555	cg18121684	SERPINB13	-0.13	9.87E-08
3556	cg24691255	SERPINB2	-0.21	1.15E-12
3557	cg10533434	SERPINB3	-0.19	1.21E-20
3558	cg15772361	SERPINB3	-0.28	3.31E-17
3559	cg24546942	SERPINB4	-0.20	9.51E-18
3560	cg15422147	SERPINB5	-0.17	1.73E-12
3561	cg01568736	SERPINB7	-0.28	2.47E-20
3562	cg01770400	SERPINC1	-0.17	2.72E-09
3563	cg02523400	SERPIND1	-0.12	1.04E-05
3564	cg27596068	SERPINH1	-0.12	3.50E-11
3565	cg04809136	SF3B14	0.53	1.49E-33
3566	cg03421300	SFN	-0.11	1.54E-06
3567	cg11354906	SFRP2	0.14	4.20E-07
3568	cg20019546	SFRP4	0.27	4.17E-12
3569	cg19166347	SFRP4	0.14	2.00E-06
3570	cg06692050	SFRP5	0.14	3.07E-07
3571	cg11206634	SFT2D3	-0.11	9.22E-04
3572	cg26394380	SFTPB	-0.16	7.11E-12
3573	cg08458487	SFTPD	-0.15	3.70E-11
3574	cg03600318	SFTPD	-0.15	1.01E-13
3575	cg04582295	SGCA	-0.21	5.89E-14
3576	cg23653712	SGCB	0.16	2.80E-06
3577	cg04435420	SGCD	-0.15	4.75E-09
3578	cg03811411	SGCD	-0.20	9.52E-09
3579	cg07126559	SGCG	-0.21	6.96E-16
3580	cg17463527	SGK2	-0.12	1.32E-09
3581	cg21685427	SGK2	-0.16	5.44E-09
3582	cg15787039	SGNE1	0.28	3.78E-28
3583	cg16884042	SGPP2	0.13	2.87E-05
3584	cg26379475	SH2D1B	-0.12	8.83E-08
3585	cg12499211	SH2D2A	-0.12	6.21E-04
3586	cg20839149	SH2D2A	-0.17	3.39E-09
3587	cg01414934	SH3BGRL3	0.11	2.24E-05
3588	cg08822227	SH3BP2	0.24	3.70E-21
3589	cg07991621	SH3BP2	0.11	6.24E-11
3590	cg22946150	SH3GL3	0.17	1.17E-04
3591	cg07816074	SH3TC1	-0.12	9.47E-06
3592	cg21825027	SH3YL1	0.56	3.83E-29
3593	cg11801011	SHANK1	-0.21	5.14E-12
3594	cg24824840	SHANK1	-0.29	4.71E-19
3595	cg04396791	SHANK2	0.14	4.39E-11

3596	cg10362475	SHANK2	0.12	1.31E-10
3597	cg18581445	SHD	0.19	4.18E-07
3598	cg09381003	SHKBP1	0.27	5.74E-14
3599	cg21022395	SHOC2	0.12	4.70E-07
3600	cg06156376	SHOX2	0.18	7.54E-06
3601	cg17191178	SHOX2	0.17	8.00E-09
3602	cg18149919	SIAHBP1	-0.23	4.96E-13
3603	cg03597525	SIGLEC10	-0.22	2.54E-16
3604	cg03283694	SIGLEC11	-0.13	1.81E-12
3605	cg24311282	SIGLEC11	-0.24	4.85E-14
3606	cg12530021	SIGLEC12	-0.17	3.57E-14
3607	cg18986165	SIGLEC12	-0.35	2.05E-19
3608	cg01193293	SIGLEC7	-0.23	6.96E-20
3609	cg23458892	SIGLEC7	-0.28	7.25E-22
3610	cg04164824	SIGLEC9	-0.14	9.40E-18
3611	cg13407883	SIGLEC9	-0.30	3.92E-21
3612	cg11540692	SIM1	0.25	3.86E-15
3613	cg26668713	SIPA1	0.10	1.11E-02
3614	cg09577651	SIRPB1	-0.18	1.09E-15
3615	cg11061975	SIRPB2	-0.28	2.83E-21
3616	cg25737664	SIRPD	-0.29	8.13E-20
3617	cg03310469	SIX2	0.14	2.96E-05
3618	cg13163729	SIX3	0.13	2.13E-06
3619	cg19456540	SIX6	0.24	6.27E-08
3620	cg02794695	SLA	-0.18	1.41E-07
3621	cg08996413	SLA	-0.27	2.15E-21
3622	cg19283196	SLC10A4	0.36	1.67E-19
3623	cg08209133	SLC10A4	0.27	8.11E-14
3624	cg07719512	SLC11A1	-0.13	1.98E-08
3625	cg18854666	SLC11A1	-0.13	1.35E-07
3626	cg14930674	SLC12A1	-0.26	8.72E-16
3627	cg23656386	SLC12A4	0.14	8.21E-08
3628	cg02005755	SLC13A4	-0.18	6.73E-12
3629	cg17589341	SLC14A1	-0.11	1.64E-07
3630	cg21992250	SLC15A3	0.33	3.14E-15
3631	cg01172899	SLC16A14	0.12	3.28E-04
3632	cg14417329	SLC16A3	0.23	3.53E-13
3633	cg27619475	SLC16A5	0.15	1.04E-12
3634	cg09300114	SLC16A5	0.12	2.96E-09
3635	cg11879514	SLC16A6	0.17	2.27E-07
3636	cg20439022	SLC16A8	0.22	4.32E-20
3637	cg21627181	SLC17A4	-0.12	3.30E-05
3638	cg06836849	SLC17A8	0.16	1.01E-07
3639	cg13857100	SLC18A1	-0.13	1.02E-10
3640	cg00512279	SLC18A2	0.11	1.29E-04
3641	cg14008883	SLC18A3	0.20	1.95E-08
3642	cg06192619	SLC1A6	-0.18	8.12E-15
3643	cg16377872	SLC1A6	-0.20	2.45E-13
3644	cg27292431	SLC22A1	0.18	6.85E-10
3645	cg09326702	SLC22A11	-0.12	9.52E-06

3646	cg22334665	SLC22A16	0.30	1.52E-14
3647	cg18521925	SLC22A16	-0.11	2.69E-07
3648	cg01557297	SLC22A17	0.18	2.26E-06
3649	cg21019522	SLC22A18	0.16	4.51E-16
3650	cg19906550	SLC22A18	0.15	3.79E-19
3651	cg02719634	SLC22A18AS	-0.12	1.79E-05
3652	cg08999895	SLC22A18AS	-0.27	2.14E-17
3653	cg25313204	SLC22A3	-0.15	3.67E-04
3654	cg12439773	SLC22A6	-0.12	3.59E-08
3655	cg12348970	SLC24A2	-0.30	1.35E-23
3656	cg00888479	SLC24A3	0.11	5.82E-04
3657	cg01557989	SLC24A3	0.11	8.48E-05
3658	cg19409156	SLC24A5	-0.19	4.26E-13
3659	cg01497576	SLC24A5	-0.19	7.81E-10
3660	cg15275890	SLC25A17	-0.17	1.78E-10
3661	cg25152631	SLC25A36	0.26	1.29E-09
3662	cg25054311	SLC26A2	0.12	8.81E-04
3663	cg22294577	SLC26A3	-0.16	5.33E-11
3664	cg09492887	SLC26A5	0.25	5.66E-12
3665	cg15674432	SLC26A8	0.17	5.56E-11
3666	cg23036025	SLC27A5	-0.10	8.69E-08
3667	cg16107172	SLC27A5	-0.12	3.77E-05
3668	cg01993576	SLC29A1	-0.17	3.16E-08
3669	cg05168404	SLC29A4	-0.13	5.90E-06
3670	cg26133068	SLC2A11	0.11	5.71E-04
3671	cg05521696	SLC2A14	0.36	4.63E-21
3672	cg13323752	SLC2A14	0.31	2.54E-12
3673	cg17142134	SLC2A2	-0.15	1.93E-07
3674	cg24480859	SLC2A5	-0.13	4.73E-07
3675	cg24027679	SLC2A7	-0.15	2.63E-09
3676	cg08649013	SLC2A9	-0.13	2.85E-09
3677	cg23338195	SLC30A8	-0.14	1.26E-11
3678	cg10516359	SLC35C1	-0.22	3.48E-11
3679	cg25437385	SLC35F3	0.26	1.25E-08
3680	cg22445920	SLC36A3	-0.22	1.23E-11
3681	cg13265003	SLC37A1	-0.14	1.07E-11
3682	cg26550234	SLC39A12	-0.16	2.46E-11
3683	cg05254747	SLC39A14	-0.10	3.06E-04
3684	cg02192965	SLC3A1	-0.13	2.39E-06
3685	cg27336379	SLC44A5	-0.19	1.06E-09
3686	cg23280807	SLC5A11	-0.12	1.13E-03
3687	cg20092728	SLC5A12	-0.13	8.80E-09
3688	cg21578906	SLC5A4	-0.27	2.47E-17
3689	cg16232126	SLC5A7	0.11	2.70E-04
3690	cg22415472	SLC5A7	-0.12	2.95E-05
3691	cg16005224	SLC5A8	-0.11	4.86E-05
3692	cg09395732	SLC6A11	-0.19	3.97E-09
3693	cg03064067	SLC6A15	0.14	1.46E-05
3694	cg19421752	SLC6A18	-0.22	7.61E-21
3695	cg02064402	SLC6A18	-0.30	5.34E-19

3696	cg04970117	SLC6A20	0.28	2.02E-11
3697	cg05016953	SLC6A4	0.22	9.78E-10
3698	cg21291896	SLC6A6	0.24	3.33E-09
3699	cg13763232	SLC6A6	-0.12	1.98E-08
3700	cg19435264	SLC6A7	0.23	2.45E-09
3701	cg14424530	SLC7A1	0.10	6.06E-03
3702	cg06690548	SLC7A11	-0.22	1.33E-12
3703	cg26907768	SLC7A5	0.16	4.14E-09
3704	cg24734575	SLC7A9	-0.11	8.73E-06
3705	cg10807560	SLC8A1	-0.34	5.83E-25
3706	cg22123464	SLC8A2	0.19	1.75E-09
3707	cg02748539	SLC9A3	0.14	1.02E-05
3708	cg09210315	SLCO4A1	-0.10	3.49E-07
3709	cg27081230	SLIC1	-0.12	6.17E-09
3710	cg27431150	SLIC1	-0.20	3.23E-10
3711	cg18972811	SLIT2	0.17	9.38E-06
3712	cg09083627	SLITRK5	0.25	3.32E-09
3713	cg12237269	SLN	-0.17	1.04E-09
3714	cg07441143	SLURP1	-0.18	1.33E-12
3715	cg27508002	SMAD9	-0.17	1.23E-11
3716	cg16194715	SMAD9	-0.22	1.39E-11
3717	cg02827112	SMARCAD1	0.10	1.40E-03
3718	cg09542291	SMCP	-0.17	6.30E-11
3719	cg21948655	SMCP	-0.22	3.10E-13
3720	cg10556064	SMPD3	0.27	5.83E-25
3721	cg15201635	SMPD3	0.26	2.00E-11
3722	cg00891541	SMPD3	0.25	9.38E-24
3723	cg19297232	SMPD3	0.19	1.03E-19
3724	cg25629694	SMURF2	0.12	4.39E-08
3725	cg05403071	SNAI1	0.20	1.42E-07
3726	cg07576541	SNAPAP	-0.12	6.50E-06
3727	cg04747322	SNCAIP	0.10	2.94E-05
3728	cg09816471	SNN	0.16	3.84E-06
3729	cg22555495	SNRPN	-0.12	2.51E-06
3730	cg19803984	SNRPN	-0.15	3.88E-14
3731	cg16321029	SNRPN	-0.15	2.83E-13
3732	cg15585987	SNTG1	-0.11	8.66E-08
3733	cg20179697	SNX9	0.17	1.50E-09
3734	cg06220235	SOCS1	0.30	1.07E-08
3735	cg04797323	SOCS2	0.35	2.82E-12
3736	cg23412850	SOCS2	0.33	2.11E-11
3737	cg11738543	SOCS2	0.27	1.20E-09
3738	cg22125370	SOCS2	0.18	2.38E-07
3739	cg11225410	SOCS2	0.10	8.27E-05
3740	cg27637521	SOCS3	0.13	1.12E-02
3741	cg22264436	SOST	-0.20	2.24E-12
3742	cg14256699	SOST	-0.27	1.27E-14
3743	cg22303211	SOX1	0.19	2.85E-15
3744	cg20008332	SOX11	0.13	2.84E-04
3745	cg08432727	SOX11	0.11	4.91E-04

3746	cg03811478	SOX14	0.21	2.22E-15
3747	cg16428251	SOX14	0.13	9.69E-05
3748	cg02919422	SOX17	0.20	7.16E-07
3749	cg15105987	SOX2	0.20	1.67E-15
3750	cg19063972	SOX21	0.20	5.57E-07
3751	cg11208483	SOX21	0.18	4.94E-15
3752	cg06200339	SOX30	-0.10	1.26E-04
3753	cg21530890	SOX8	0.29	3.62E-12
3754	cg27210136	SP6	-0.20	2.00E-17
3755	cg23355492	SP6	-0.21	5.06E-13
3756	cg05836145	SP8	0.10	3.27E-05
3757	cg13334277	SPACA1	-0.12	6.28E-04
3758	cg26829529	SPACA3	-0.16	1.33E-22
3759	cg11161417	SPACA3	-0.23	4.37E-25
3760	cg15670863	SPACA4	-0.24	4.53E-17
3761	cg25107903	SPAG11	-0.15	5.72E-19
3762	cg19787037	SPAG11	-0.21	2.37E-25
3763	cg04624659	SPAG17	0.11	1.03E-05
3764	cg08642068	SPAG4L	-0.13	3.88E-07
3765	cg25802093	SPAG6	0.41	1.66E-21
3766	cg06908778	SPAG6	0.32	5.59E-15
3767	cg22724153	SPAM1	-0.13	5.10E-10
3768	cg08331313	SPARC	0.20	8.34E-11
3769	cg01216369	SPATA16	-0.15	2.86E-08
3770	cg06577725	SPATA16	-0.19	1.35E-11
3771	cg09022993	SPATA18	0.21	1.21E-10
3772	cg04141379	SPATA6	0.12	1.10E-04
3773	cg01407341	SPATA8	-0.19	2.26E-12
3774	cg02423618	SPATA8	-0.23	5.77E-22
3775	cg22970435	SPATS1	0.19	4.74E-07
3776	cg17240454	SPDEF	-0.10	1.62E-07
3777	cg04786857	SPDY1	0.56	1.18E-41
3778	cg06816106	SPDY1	0.38	6.62E-18
3779	cg18755783	SPG20	0.23	2.41E-09
3780	cg01917648	SPIC	-0.14	3.26E-12
3781	cg04577715	SPINK1	-0.20	4.66E-09
3782	cg04103317	SPINK4	-0.14	1.52E-10
3783	cg27488807	SPINK7	-0.10	5.28E-11
3784	cg23765993	SPINLW1	-0.13	9.30E-12
3785	cg04868764	SPINT1	0.32	8.05E-14
3786	cg26531804	SPINT1	0.28	5.89E-17
3787	cg15375239	SPINT2	0.50	6.56E-24
3788	cg13301014	SPINT2	0.30	2.22E-16
3789	cg13888886	SPO11	-0.18	7.46E-16
3790	cg12832649	SPOCK	0.16	2.32E-05
3791	cg10983208	SPOCK2	0.23	6.58E-09
3792	cg06101324	SPRR1A	-0.25	3.93E-30
3793	cg04505023	SPRR1A	-0.37	3.71E-27
3794	cg24884084	SPRR1B	-0.11	2.68E-13
3795	cg18780284	SPRR1B	-0.35	4.73E-30

3796	cg12891678	SPRR2D	-0.26	5.22E-33
3797	cg08555657	SPRR2E	-0.27	4.55E-31
3798	cg00152644	SPRR2E	-0.36	2.48E-28
3799	cg25856811	SPRR3	-0.33	8.10E-33
3800	cg04138756	SPRR3	-0.39	3.47E-30
3801	cg02202484	SPRR4	-0.15	6.73E-13
3802	cg08763351	SPRR4	-0.29	2.06E-17
3803	cg22036988	SPSB4	0.10	4.39E-04
3804	cg15403517	SRD5A2	0.31	1.77E-20
3805	cg19502744	SRD5A2	0.16	2.34E-03
3806	cg02399455	SRI	-0.12	4.82E-06
3807	cg03330516	SRMS	-0.25	3.58E-16
3808	cg22285621	SSH3	-0.10	7.48E-05
3809	cg27291231	SSNA1	-0.17	1.24E-09
3810	cg24509668	SSPN	0.10	3.25E-05
3811	cg02164046	SST	0.31	1.18E-14
3812	cg14297029	SSTR3	-0.16	2.07E-10
3813	cg10089145	ST14	0.17	4.61E-04
3814	cg15928398	ST6GAL1	0.20	2.33E-11
3815	cg25725843	ST6GAL2	0.16	2.20E-08
3816	cg08666623	ST6GALNAC2	0.15	1.83E-06
3817	cg21522303	ST6GALNAC2	0.12	1.17E-06
3818	cg26363196	ST6GALNAC3	0.17	4.47E-08
3819	cg20339230	ST8SIA2	0.24	5.02E-09
3820	cg19751300	ST8SIA5	0.36	6.68E-19
3821	cg23129478	ST8SIA5	0.15	7.02E-08
3822	cg17471928	STAC2	0.14	6.05E-06
3823	cg21309147	STAC2	0.13	4.96E-05
3824	cg08624068	STARD6	-0.14	9.45E-08
3825	cg03001305	STAT5A	0.12	2.04E-05
3826	cg00436282	STATH	-0.11	3.20E-06
3827	cg18320336	STEAP1	0.16	1.97E-10
3828	cg00564163	STEAP4	0.30	3.31E-14
3829	cg11271605	STEAP4	0.27	1.10E-12
3830	cg06782692	STK32C	0.36	6.41E-14
3831	cg07284558	STK32C	0.17	1.15E-06
3832	cg08788717	STK33	0.14	1.95E-06
3833	cg23326689	STMN2	0.12	1.81E-04
3834	cg08291098	STMN3	0.29	6.61E-16
3835	cg02130905	STMN4	-0.14	2.13E-10
3836	cg25762706	STMN4	-0.16	1.31E-10
3837	cg03712843	STOML3	-0.10	4.02E-09
3838	cg25061755	STON1	-0.18	9.61E-11
3839	cg10334928	STON2	0.20	4.62E-13
3840	cg06813842	STRC	-0.16	2.14E-14
3841	cg15301694	STRN3	-0.11	1.52E-05
3842	cg17328659	STUB1	0.12	1.29E-03
3843	cg06948294	STXBP6	0.14	1.21E-07
3844	cg00616129	STYK1	0.13	2.42E-05
3845	cg08088989	SUHW1	-0.17	7.86E-11

3846	cg18530748	SULT1A1	0.22	9.43E-07
3847	cg15415545	SULT1A3	0.40	9.03E-21
3848	cg19450025	SULT1A3	0.25	3.48E-14
3849	cg10648113	SULT1B1	-0.11	2.38E-18
3850	cg23163573	SULT1C1	-0.17	2.15E-16
3851	cg10236239	SULT1C2	0.14	1.48E-07
3852	cg19139729	SULT2A1	-0.10	6.71E-07
3853	cg00698688	SULT2B1	-0.13	1.11E-04
3854	cg05472874	SULT4A1	0.23	3.30E-08
3855	cg16500334	SUMO3	0.15	9.92E-05
3856	cg25307641	SURF2	-0.11	7.44E-08
3857	cg04230060	SUSD1	0.16	2.19E-10
3858	cg14862827	SUSD1	0.13	1.99E-04
3859	cg01705587	SV2A	0.13	1.00E-06
3860	cg18087477	SYCP1	0.15	1.46E-05
3861	cg17696091	SYCP1	-0.19	3.29E-17
3862	cg07347645	SYCP2	-0.15	1.22E-07
3863	cg14304761	SYK	0.19	7.95E-07
3864	cg02608019	SYK	0.16	1.97E-05
3865	cg23447996	SYK	0.15	9.92E-08
3866	cg05801648	SYK	0.15	1.24E-07
3867	cg10025443	SYK	0.14	3.13E-06
3868	cg15873301	SYN2	0.23	1.79E-09
3869	cg16894211	SYN3	-0.12	1.15E-06
3870	cg19713460	SYNGR1	0.16	6.92E-05
3871	cg15555014	SYNGR2	-0.10	1.71E-05
3872	cg02519806	SYNPO2	0.13	6.43E-04
3873	cg07127957	SYNPO2L	-0.13	3.10E-09
3874	cg00902195	SYT10	0.13	8.82E-06
3875	cg23950724	SYT10	0.10	8.50E-04
3876	cg05715649	SYT2	-0.11	3.00E-08
3877	cg12289045	SYT3	-0.10	4.27E-08
3878	cg20239740	SYT5	0.13	1.95E-05
3879	cg05368341	SYT6	0.11	2.45E-05
3880	cg01806928	SYT9	0.15	1.86E-06
3881	cg17188046	T	0.11	1.61E-03
3882	cg17829936	TAAR5	-0.19	7.42E-10
3883	cg23887102	TAAR6	-0.10	3.16E-06
3884	cg26033710	TACR1	-0.11	1.70E-08
3885	cg15278682	TACR2	-0.12	1.75E-06
3886	cg26984805	TACR2	-0.23	8.13E-11
3887	cg16076328	TACSTD1	0.39	1.97E-22
3888	cg16080552	TACSTD2	0.20	1.05E-10
3889	cg19797376	TAL1	0.26	3.94E-15
3890	cg23871659	TANK	0.12	1.22E-08
3891	cg18524091	TAS1R2	-0.14	9.04E-07
3892	cg21731286	TAS1R2	-0.14	2.18E-10
3893	cg12150401	TAS2R16	-0.25	6.16E-24
3894	cg25608041	TBC1D1	0.28	4.40E-27
3895	cg12718562	TBC1D21	-0.31	4.55E-22

3896	cg14532417	TBC1D3	-0.18	2.62E-19
3897	cg04901273	TBC1D3	-0.18	3.05E-22
3898	cg23350580	TBC1D3C	-0.21	7.79E-29
3899	cg02601403	TBC1D3C	-0.27	7.94E-27
3900	cg27574244	TBCD	0.27	1.38E-13
3901	cg22511633	TBL2	0.20	5.80E-09
3902	cg25548825	TBX10	-0.13	4.16E-10
3903	cg26422060	TBX10	-0.16	6.17E-10
3904	cg12163132	TBX2	0.25	6.39E-15
3905	cg13274713	TBX2	0.12	9.63E-04
3906	cg13870866	TBX20	0.20	9.02E-09
3907	cg02008154	TBX20	0.18	1.66E-07
3908	cg20209009	TBX21	0.31	1.97E-11
3909	cg26607785	TBX21	0.22	4.66E-12
3910	cg18536148	TBX4	0.45	7.89E-21
3911	cg03866607	TBX4	0.19	2.60E-18
3912	cg21907579	TBX5	0.13	7.17E-05
3913	cg14370448	TBX6	-0.12	8.15E-04
3914	cg01293143	TCEA2	-0.10	5.76E-04
3915	cg03943081	TCERG1L	0.25	5.67E-10
3916	cg16175725	TCF1	-0.11	3.21E-05
3917	cg06444781	TCF1	-0.14	4.59E-06
3918	cg14532519	TCF20	0.12	8.50E-13
3919	cg24215443	TCF21	0.20	2.89E-09
3920	cg24861272	TCF8	-0.11	4.78E-22
3921	cg14127336	TCL1A	-0.23	2.54E-17
3922	cg13771579	TCL1B	-0.13	1.10E-07
3923	cg27504299	TCL1B	-0.32	3.65E-22
3924	cg05023540	TCL6	-0.20	3.38E-35
3925	cg20018806	TCN1	-0.20	1.78E-18
3926	cg22407458	TCP11	0.10	1.01E-10
3927	cg24110050	TCTEX1D1	0.34	1.07E-23
3928	cg17819635	TCTEX1D1	0.28	1.01E-19
3929	cg08121954	TDO2	0.11	2.76E-05
3930	cg12277666	TDRD5	0.23	5.74E-09
3931	cg02915837	TEAD4	0.14	2.75E-04
3932	cg25829729	TEB1	-0.18	2.24E-12
3933	cg26530497	TEB1	-0.18	5.12E-13
3934	cg24813212	TEKT2	0.24	2.15E-10
3935	cg02016419	TEKT3	0.15	6.91E-05
3936	cg02545192	TERT	0.27	4.26E-11
3937	cg12439899	TFAP2A	0.24	1.81E-11
3938	cg25202471	TFAP2A	0.19	5.77E-09
3939	cg26372517	TFAP2E	0.11	2.02E-02
3940	cg02906238	TFCP2L1	0.34	2.04E-13
3941	cg18729973	TFF1	-0.22	1.19E-14
3942	cg02643667	TFF1	-0.25	4.96E-17
3943	cg11158374	TFF2	-0.27	5.35E-17
3944	cg04806409	TFF3	-0.16	1.14E-07
3945	cg20488657	TFF3	-0.22	1.19E-09

3946	cg23141855	TFPI2	0.11	3.26E-05
3947	cg10681065	TFR2	-0.11	1.19E-04
3948	cg15526708	TGFBR1	0.12	2.44E-06
3949	cg00563926	TGFBR3	0.13	1.46E-06
3950	cg27496506	TGM5	-0.23	1.28E-16
3951	cg23278885	TGM6	-0.24	2.33E-17
3952	cg05912121	TH	-0.18	3.41E-11
3953	cg08573687	TH	-0.26	4.17E-12
3954	cg13798289	THAP11	-0.16	2.32E-11
3955	cg18110483	THBS4	0.16	2.23E-07
3956	cg27465849	THEDC1	-0.20	1.23E-13
3957	cg18338296	THRSP	0.14	2.84E-05
3958	cg09174741	THSD1	-0.12	1.03E-21
3959	cg07965823	THSD3	0.28	3.68E-11
3960	cg06469542	THSD3	0.11	9.10E-04
3961	cg12508624	THY1	0.18	4.15E-07
3962	cg21633698	THY1	-0.13	4.76E-06
3963	cg04807655	TIAM1	0.15	1.45E-05
3964	cg15853125	TIAM1	0.12	7.71E-04
3965	cg20925955	TIAM2	-0.13	3.53E-06
3966	cg09837169	TIAM2	-0.14	5.49E-07
3967	cg18994063	TIMD4	-0.13	2.96E-10
3968	cg03870261	TIMM13	0.12	1.48E-03
3969	cg24080529	TIMP3	-0.16	2.10E-10
3970	cg14869028	TINAGL1	0.32	3.10E-18
3971	cg22855405	TINAGL1	0.30	1.42E-13
3972	cg27022827	TJP3	-0.17	3.47E-09
3973	cg05745457	TKTL2	-0.12	3.78E-08
3974	cg16413535	TKTL2	-0.19	3.79E-10
3975	cg15915418	TLE1	0.25	3.71E-12
3976	cg08794763	TLE6	-0.20	2.31E-11
3977	cg23181434	TLK2	-0.14	1.56E-12
3978	cg25720804	TLX3	0.53	1.66E-26
3979	cg25942450	TLX3	0.41	2.66E-22
3980	cg08124030	TM4SF1	0.11	1.31E-02
3981	cg23713520	TM4SF11	0.22	1.50E-08
3982	cg05556202	TM4SF19	-0.13	1.24E-06
3983	cg05445326	TM4SF19	-0.13	1.68E-07
3984	cg20683151	TM4SF20	-0.20	1.24E-12
3985	cg14696396	TM6SF1	0.19	4.69E-06
3986	cg20277416	TM7SF2	-0.11	5.15E-04
3987	cg03243506	TMC2	-0.19	5.20E-11
3988	cg19290962	TMC2	-0.28	2.50E-18
3989	cg25690265	TMC5	-0.12	1.07E-06
3990	cg19056418	TMC8	-0.13	6.85E-06
3991	cg03998348	TMCO5	-0.15	5.41E-12
3992	cg22775000	TMEFF1	0.33	1.01E-11
3993	cg15075170	TMEFF1	0.12	1.91E-03
3994	cg06825166	TMEM10	-0.25	9.00E-14
3995	cg14782678	TMEM102	-0.13	2.45E-04

3996	cg04482110	TMEM106A	0.12	1.44E-02
3997	cg10735607	TMEM109	-0.14	9.95E-06
3998	cg16708012	TMEM119	0.12	9.39E-12
3999	cg23886551	TMEM121	0.14	2.03E-05
4000	cg04355435	TMEM125	0.24	4.46E-11
4001	cg02089348	TMEM129	-0.17	5.45E-08
4002	cg21505886	TMEM129	-0.17	7.66E-08
4003	cg19372178	TMEM16G	-0.19	8.01E-17
4004	cg02672493	TMEM22	0.14	7.52E-04
4005	cg20001829	TMEM25	0.15	4.96E-06
4006	cg21706946	TMEM40	-0.14	1.52E-08
4007	cg03534410	TMEM40	-0.19	8.73E-13
4008	cg05385377	TMEM51	0.11	2.27E-03
4009	cg06179485	TMEM54	0.13	4.90E-07
4010	cg20955688	TMEM71	-0.12	1.61E-05
4011	cg27159719	TMEM71	-0.15	1.16E-13
4012	cg26457013	TMEM86B	-0.14	2.18E-05
4013	cg01076838	TMEM86B	-0.25	5.66E-17
4014	cg26912636	TMEPAI	-0.20	2.50E-14
4015	cg00138126	TMEPAI	-0.27	1.23E-21
4016	cg13486556	TMOD2	0.10	9.28E-04
4017	cg19686152	TMOD3	-0.10	1.15E-08
4018	cg24901042	TMPRSS2	0.14	1.03E-04
4019	cg11484872	TNF	-0.21	1.88E-17
4020	cg04425624	TNF	-0.24	1.32E-12
4021	cg20368904	TNFAIP2	0.15	1.57E-14
4022	cg26530341	TNFRSF10A	-0.15	1.02E-06
4023	cg27090216	TNFRSF10C	0.37	4.62E-15
4024	cg05636175	TNFRSF10C	0.27	6.27E-16
4025	cg14015044	TNFRSF10C	0.23	4.49E-09
4026	cg02938601	TNFRSF10C	0.18	2.64E-10
4027	cg16175263	TNFRSF10C	0.16	8.82E-09
4028	cg01407244	TNFRSF10C	0.11	9.40E-07
4029	cg18152830	TNFRSF13B	-0.19	7.23E-13
4030	cg23274244	TNFRSF13B	-0.22	2.82E-14
4031	cg00504595	TNFRSF19	-0.11	1.53E-13
4032	cg26189983	TNFRSF1B	0.10	6.82E-03
4033	cg23867494	TNFRSF4	-0.13	4.28E-05
4034	cg22335801	TNFRSF4	-0.20	1.19E-08
4035	cg08840010	TNFRSF9	-0.12	4.17E-11
4036	cg21094154	TNFSF11	-0.13	1.75E-08
4037	cg16517394	TNFSF4	0.14	8.83E-08
4038	cg10861599	TNFSF4	0.13	1.22E-07
4039	cg01186777	TNFSF9	0.26	1.00E-09
4040	cg21295911	TNFSF9	0.25	3.04E-10
4041	cg20950277	TNIP3	-0.16	1.19E-13
4042	cg01962086	TNK2	0.16	7.26E-06
4043	cg11688469	TNNI1	-0.10	2.94E-06
4044	cg12114524	TNNI1	-0.31	5.30E-22
4045	cg14672680	TNNI2	-0.14	3.15E-10

4046	cg18330203	TNNT2	-0.17	3.81E-08
4047	cg19642007	TNNT3	-0.32	7.05E-19
4048	cg13980719	TNP1	-0.10	5.95E-05
4049	cg10376763	TNP1	-0.14	3.52E-07
4050	cg10331038	TNR	-0.20	2.76E-14
4051	cg26050734	TNRC4	0.11	5.98E-06
4052	cg13823701	TNXB	-0.11	1.07E-07
4053	cg22424444	TOLLIP	-0.23	8.72E-15
4054	cg12188860	TOP1MT	-0.32	5.76E-20
4055	cg22105022	TOR2A	-0.10	1.30E-06
4056	cg04391111	TP73	0.23	6.28E-07
4057	cg00565688	TP73	0.12	9.40E-03
4058	cg27472295	TPBG	0.11	1.90E-05
4059	cg22484980	TPCN2	-0.11	1.03E-07
4060	cg17691309	TPM2	0.21	9.79E-10
4061	cg24070292	TPM2	0.11	1.71E-07
4062	cg24490338	TPM3	-0.13	4.48E-07
4063	cg10370591	TPO	-0.28	6.59E-26
4064	cg16016036	TPO	-0.32	7.46E-22
4065	cg27044702	TPSAB1	-0.13	2.51E-08
4066	cg05873268	TPSAB1	-0.21	3.60E-20
4067	cg08321330	TPSB2	-0.24	2.23E-20
4068	cg03776850	TPSD1	-0.13	2.82E-12
4069	cg01375871	TPSD1	-0.16	6.50E-17
4070	cg15551881	TRAF1	0.10	4.88E-06
4071	cg08804892	TRAK1	0.15	9.78E-10
4072	cg24877842	TRAK1	0.12	1.06E-11
4073	cg01667702	TRAPPC1	0.17	1.90E-10
4074	cg01837574	TRAPPC1	0.15	1.01E-10
4075	cg06196379	TREM1	-0.18	1.26E-23
4076	cg01980222	TREM2	-0.18	4.88E-13
4077	cg20095587	TREM2	-0.39	1.17E-26
4078	cg04454050	TREML1	-0.20	2.04E-11
4079	cg01564343	TREML1	-0.28	1.07E-19
4080	cg26928682	TREML2	-0.12	5.94E-07
4081	cg01009664	TRH	0.12	2.48E-04
4082	cg15120497	TRH	-0.19	1.09E-07
4083	cg26989103	TRHR	-0.13	1.40E-08
4084	cg22268164	TRHR	-0.19	2.56E-17
4085	cg12793610	TRIM2	0.15	2.69E-11
4086	cg13625403	TRIM29	-0.18	1.56E-13
4087	cg00679556	TRIM31	-0.23	2.67E-17
4088	cg10693071	TRIM36	0.14	1.43E-06
4089	cg09312149	TRIM36	0.13	2.91E-05
4090	cg12242338	TRIM42	-0.25	3.27E-16
4091	cg15495837	TRIM43	-0.17	1.01E-19
4092	cg02284188	TRIM49	-0.22	2.61E-25
4093	cg03882305	TRIM50C	0.18	6.48E-11
4094	cg04962134	TRIM51	-0.32	1.94E-33
4095	cg07533148	TRIM58	0.34	1.29E-11

4096	cg10273210	TRIM59	0.22	4.17E-08
4097	cg23704082	TRIM59	0.14	4.08E-05
4098	cg07485777	TRIM60	-0.17	1.76E-11
4099	cg16051685	TRIM63	-0.15	1.21E-08
4100	cg09079593	TRIM7	0.12	1.93E-04
4101	cg03361068	TRIM9	0.10	2.52E-04
4102	cg06493386	TRPA1	0.17	2.66E-07
4103	cg24794531	TRPC1	0.29	4.23E-12
4104	cg18474934	TRPC3	-0.15	1.31E-05
4105	cg06812844	TRPM2	-0.16	5.87E-11
4106	cg16832407	TRPM3	0.30	1.14E-18
4107	cg20555507	TRPM3	0.22	1.32E-10
4108	cg07882535	TRPM5	-0.17	9.44E-11
4109	cg11474811	TRPM5	-0.26	1.53E-20
4110	cg15746445	TRPM8	-0.18	1.81E-16
4111	cg21312090	TRPS1	0.14	2.12E-05
4112	cg19615059	TRPV4	0.18	1.61E-05
4113	cg13628514	TRPV4	0.16	3.10E-05
4114	cg14269477	TRPV5	-0.14	1.50E-10
4115	cg13675849	TRPV5	-0.23	2.09E-15
4116	cg02786019	TRPV6	-0.13	2.10E-06
4117	cg14153740	TRY1	-0.28	8.51E-22
4118	cg26197220	TSC22D1	0.30	2.75E-10
4119	cg07758904	TSCOT	0.18	1.02E-05
4120	cg02332073	TSGA13	-0.16	2.17E-18
4121	cg11058932	TSGA13	-0.20	3.31E-21
4122	cg15089387	TSLP	0.15	7.11E-06
4123	cg19875656	TSP50	-0.16	3.36E-07
4124	cg08831594	TSPAN15	0.28	2.63E-12
4125	cg12610070	TSPAN15	0.12	4.47E-04
4126	cg09390792	TSPAN18	-0.28	3.95E-18
4127	cg06791867	TSPAN18	-0.30	6.96E-19
4128	cg03614513	TSPAN2	0.22	5.83E-10
4129	cg06043114	TSPAN2	0.12	1.79E-05
4130	cg00041575	TSPAN32	-0.14	2.27E-08
4131	cg17627559	TSPAN32	-0.15	1.74E-06
4132	cg03116740	TSPAN4	-0.13	5.77E-08
4133	cg11173246	TSPAN4	-0.16	1.76E-09
4134	cg00622677	TSPAN5	0.11	1.27E-04
4135	cg15747595	TSPYL5	0.38	2.47E-39
4136	cg00186701	TSPYL5	0.22	1.57E-16
4137	cg16581199	TSSK1	-0.10	1.58E-06
4138	cg23973000	TSSK1	-0.16	2.29E-12
4139	cg02255609	TTC4	0.16	1.40E-05
4140	cg19584957	TTLL10	-0.21	8.42E-17
4141	cg08137716	TTLL6	0.15	1.16E-08
4142	cg15480475	TUB	-0.14	2.30E-09
4143	cg05492113	TUB	-0.21	1.40E-16
4144	cg22675150	TUBA2	-0.12	3.08E-07
4145	cg06437862	TUBA2	-0.31	7.36E-14

4146	cg03462055	TUBB2B	0.15	7.68E-06
4147	cg23517605	TUBB2B	0.15	9.12E-06
4148	cg15669092	TUBB3	0.13	1.54E-04
4149	cg13494498	TUBB3	0.11	8.45E-04
4150	cg15677294	TUBB4	-0.19	7.49E-10
4151	cg07307078	TUBB6	0.24	1.05E-09
4152	cg22467216	TUBB6	0.15	1.83E-06
4153	cg01036012	TUBB8	-0.13	5.06E-10
4154	cg07908874	TUBGCP2	-0.12	2.90E-04
4155	cg19353006	TUSC3	-0.21	1.14E-13
4156	cg20052718	TWIST1	0.22	1.28E-10
4157	cg26312150	TWIST1	0.19	5.01E-09
4158	cg23435746	TWSG1	0.15	6.22E-08
4159	cg06243393	TXNDC2	-0.11	1.48E-06
4160	cg05547500	TXNDC2	-0.12	5.21E-07
4161	cg12099032	TXNDC3	-0.15	8.04E-09
4162	cg15028436	TXNDC3	-0.20	1.33E-16
4163	cg16069910	TXNL6	-0.14	3.38E-06
4164	cg14366490	TXNL6	-0.18	4.93E-15
4165	cg20267005	TYROBP	-0.12	3.24E-04
4166	cg13578652	UBASH3A	-0.14	4.19E-06
4167	cg07326586	UBD	-0.10	3.72E-08
4168	cg15320474	UBD	-0.17	5.78E-10
4169	cg25421647	UBE2L3	0.14	3.20E-05
4170	cg25612480	UBE2V2	-0.34	2.04E-22
4171	cg20092036	UBE3B	0.11	6.66E-07
4172	cg20492912	UBE4A	-0.11	1.95E-06
4173	cg10241097	UBL4B	-0.17	2.10E-13
4174	cg24346637	UBL4B	-0.29	1.86E-17
4175	cg01369413	UBQLN3	-0.21	2.67E-12
4176	cg24715245	UCHL1	0.20	1.45E-07
4177	cg08319991	UCHL1	0.18	1.12E-09
4178	cg04527918	UCN	0.12	1.42E-04
4179	cg20028470	UCN	0.11	2.96E-04
4180	cg20587968	UCN3	-0.19	7.85E-12
4181	cg26644395	UCN3	-0.20	6.44E-18
4182	cg14433673	UCP2	0.38	8.72E-14
4183	cg05205664	UCP2	0.38	6.44E-14
4184	cg11811840	UGT1A1	-0.13	5.59E-06
4185	cg12385643	UGT1A6	-0.12	3.32E-06
4186	cg23317501	UGT3A1	0.13	4.49E-09
4187	cg07084163	UGT3A2	0.14	8.67E-10
4188	cg25892041	UGT8	-0.14	4.27E-09
4189	cg25589890	ULBP1	0.24	6.30E-10
4190	cg02409282	ULBP1	0.15	1.11E-06
4191	cg03985136	ULBP2	0.18	9.55E-06
4192	cg18418460	ULBP3	0.12	1.43E-04
4193	cg18071006	ULBP3	0.11	1.58E-04
4194	cg12324629	ULK2	0.18	3.70E-21
4195	cg25013053	UNC45B	-0.13	4.91E-09

4196	cg22346765	UNC5CL	-0.15	2.08E-12
4197	cg10725344	UNQ1940	-0.15	9.28E-12
4198	cg10574499	UNQ2446	0.25	4.56E-13
4199	cg17074151	UNQ2541	-0.14	3.44E-12
4200	cg16536450	UNQ2541	-0.21	1.84E-12
4201	cg12315311	UNQ3033	-0.14	1.66E-19
4202	cg01718139	UNQ3033	-0.18	1.98E-11
4203	cg09082921	UNQ3045	0.11	1.03E-04
4204	cg21712685	UNQ3045	0.10	2.66E-04
4205	cg10305797	UNQ467	-0.21	4.72E-09
4206	cg05241571	UNQ467	-0.27	1.70E-11
4207	cg03003745	UNQ473	-0.13	5.27E-09
4208	cg26222045	UNQ5810	-0.12	1.97E-05
4209	cg23444894	UNQ5810	-0.14	1.69E-08
4210	cg15944856	UNQ6411	-0.25	2.12E-18
4211	cg00333226	UNQ739	0.15	2.02E-06
4212	cg01072821	UNQ9391	-0.20	2.13E-13
4213	cg04057858	UNQ9391	-0.23	8.22E-16
4214	cg17162024	UNQ9433	0.22	2.76E-08
4215	cg10613381	UPB1	-0.11	4.07E-05
4216	cg02019333	UPK1B	-0.18	9.03E-11
4217	cg19384697	UPK3B	0.20	7.05E-13
4218	cg22995176	UPK3B	0.13	1.52E-07
4219	cg21307628	URB	-0.16	2.23E-10
4220	cg24088438	URP2	0.15	1.13E-07
4221	cg21289015	USH1C	0.11	3.84E-04
4222	cg24365013	USH3A	-0.13	4.93E-09
4223	cg06910100	USHBP1	-0.10	2.65E-06
4224	cg07499372	USP29	-0.11	1.14E-07
4225	cg16547341	USP29	-0.15	9.85E-07
4226	cg18886444	USP4	0.13	3.80E-04
4227	cg08742106	USP6	-0.18	1.88E-21
4228	cg12954718	USP6	-0.18	3.96E-20
4229	cg21273029	UST	0.13	3.76E-06
4230	cg09053680	UTF1	0.47	9.69E-20
4231	cg03755123	UTF1	0.18	1.40E-06
4232	cg10634115	UTS2R	0.14	2.37E-13
4233	cg20881054	VASH1	0.31	8.14E-15
4234	cg18489434	VASH1	0.27	2.70E-12
4235	cg26571739	VAV1	-0.13	1.53E-07
4236	cg13470920	VAV1	-0.17	1.76E-08
4237	cg24662718	VAV3	0.31	2.85E-13
4238	cg00263760	VAX1	0.11	1.33E-03
4239	cg02932669	VGCNL1	0.24	6.62E-11
4240	cg04084157	VGF	0.17	1.81E-07
4241	cg08097755	VGF	0.16	1.71E-08
4242	cg03160135	VGLL2	0.19	7.03E-08
4243	cg20916523	VHL	-0.12	3.31E-03
4244	cg16869108	VHL	-0.13	9.87E-04
4245	cg26113512	VILL	0.32	2.15E-18

4246	cg04660410	VILL	0.21	2.57E-07
4247	cg12874092	VIM	0.40	7.64E-18
4248	cg18349835	VIPR2	0.32	8.29E-12
4249	cg05523047	VLDLR	0.14	7.47E-05
4250	cg12457773	VMP	0.11	3.49E-05
4251	cg19554294	VN1R2	-0.11	2.03E-11
4252	cg20832020	VSIG9	-0.11	4.78E-08
4253	cg06151165	VSX1	0.18	6.38E-07
4254	cg04706338	VTN	-0.13	1.97E-08
4255	cg14667273	VWA1	0.13	1.48E-03
4256	cg27347104	VWF	-0.15	4.44E-11
4257	cg07744166	WASF3	0.12	1.08E-05
4258	cg17703554	WDR21C	-0.26	1.10E-25
4259	cg01587454	WDR21C	-0.28	6.41E-18
4260	cg07054641	WDR52	0.23	4.86E-17
4261	cg15044041	WDR52	0.18	1.55E-15
4262	cg24652919	WDR58	-0.15	8.16E-12
4263	cg21577049	WDR69	0.15	2.29E-07
4264	cg26128092	WDR8	0.29	1.98E-09
4265	cg19135761	WDR8	0.15	1.11E-07
4266	cg25964007	WDRPUH	0.14	1.63E-04
4267	cg04420907	WDRPUH	0.11	3.21E-04
4268	cg23865698	WFDC1	-0.27	1.91E-26
4269	cg00690280	WFDC10B	-0.13	2.50E-09
4270	cg22988566	WFDC10B	-0.30	5.45E-21
4271	cg18241647	WFDC12	-0.23	1.17E-14
4272	cg20485165	WFDC12	-0.26	3.36E-21
4273	cg09874776	WFDC13	-0.10	1.64E-05
4274	cg14288464	WFDC5	-0.15	1.13E-12
4275	cg24765446	WFDC6	-0.25	1.15E-24
4276	cg22021786	WFDC8	-0.27	1.33E-12
4277	cg12061127	WFDC9	-0.27	3.03E-14
4278	cg10478221	WFIKKN1	0.15	4.54E-09
4279	cg19427610	WIF1	0.12	2.54E-04
4280	cg22637941	WISP1	-0.13	1.40E-03
4281	cg03670238	WISP1	-0.15	6.44E-07
4282	cg20985014	WISP3	-0.12	1.57E-09
4283	cg20616414	WNK2	0.44	2.62E-24
4284	cg16158807	WNK2	0.31	5.72E-15
4285	cg05164634	WNT10B	0.10	1.90E-03
4286	cg21238457	WNT11	0.26	2.99E-15
4287	cg01830294	WNT2	0.26	6.03E-15
4288	cg15647515	WNT2	0.11	1.25E-04
4289	cg00625653	WNT7A	0.23	8.30E-10
4290	cg08603768	WNT8A	-0.12	8.50E-10
4291	cg26109803	WNT8B	-0.14	5.29E-10
4292	cg13641903	WT1	0.15	7.61E-10
4293	cg01442426	XCR1	-0.26	8.78E-17
4294	cg10947146	XKR6	0.30	3.71E-10
4295	cg09485593	XYLT1	-0.15	1.30E-07

4296	cg18342279	ZAR1	0.41	4.32E-20
4297	cg25298754	ZBED2	-0.15	6.92E-10
4298	cg24323726	ZBED2	-0.20	2.85E-09
4299	cg25101936	ZBTB16	0.13	5.05E-06
4300	cg13666729	ZBTB8	0.28	8.10E-12
4301	cg11619390	ZBTB8	0.14	3.61E-05
4302	cg10133777	ZD52F10	-0.14	2.52E-16
4303	cg18429742	ZDHHC11	-0.15	3.85E-08
4304	cg27654142	ZFP2	0.15	2.96E-05
4305	cg09339527	ZFP2	0.12	4.94E-03
4306	cg04862249	ZFP3	0.25	4.77E-10
4307	cg12680609	ZFP41	0.51	1.94E-33
4308	cg24641352	ZFP41	0.11	1.26E-04
4309	cg14456683	ZIC1	0.28	9.70E-23
4310	cg05073035	ZIC1	0.25	4.97E-13
4311	cg27519373	ZIM2	-0.14	2.40E-11
4312	cg00354258	ZIM2	-0.15	7.74E-09
4313	cg16519742	ZIM2	-0.16	4.22E-07
4314	cg09837943	ZIM2	-0.17	2.57E-13
4315	cg06244906	ZIM2	-0.27	6.44E-12
4316	cg03421687	ZMYND10	0.39	4.27E-13
4317	cg23208152	ZMYND10	0.35	9.37E-11
4318	cg06088032	ZMYND10	0.30	5.40E-09
4319	cg20881888	ZMYND10	0.27	5.54E-07
4320	cg24832140	ZNF114	0.14	1.78E-07
4321	cg13877915	ZNF132	0.14	8.56E-06
4322	cg19776201	ZNF132	0.12	5.26E-08
4323	cg08849574	ZNF134	0.17	1.48E-06
4324	cg16638540	ZNF135	0.25	1.22E-08
4325	cg13714039	ZNF14	0.11	2.67E-04
4326	cg13108181	ZNF141	0.18	2.63E-06
4327	cg21790626	ZNF154	0.58	2.86E-35
4328	cg08668790	ZNF154	0.52	6.60E-32
4329	cg12586262	ZNF160	0.17	1.57E-06
4330	cg03712038	ZNF160	0.14	5.41E-06
4331	cg06566994	ZNF167	0.12	2.74E-13
4332	cg09643544	ZNF177	0.31	1.38E-13
4333	cg05250458	ZNF177	0.13	1.20E-04
4334	cg11706111	ZNF19	-0.10	1.16E-05
4335	cg20880234	ZNF198	0.15	7.47E-06
4336	cg26014197	ZNF206	-0.12	2.41E-09
4337	cg20055101	ZNF206	-0.14	1.16E-08
4338	cg01614759	ZNF22	-0.13	1.72E-05
4339	cg10604333	ZNF222	0.11	6.80E-05
4340	cg13885201	ZNF23	0.19	4.32E-07
4341	cg24680602	ZNF232	0.21	7.31E-08
4342	cg04825431	ZNF239	-0.29	1.86E-13
4343	cg02286642	ZNF254	0.30	4.13E-18
4344	cg20176532	ZNF256	0.11	1.25E-03
4345	cg16636110	ZNF264	0.17	2.69E-15

4346	cg09429185	ZNF286	0.15	5.29E-07
4347	cg23423382	ZNF287	0.18	9.62E-08
4348	cg20850981	ZNF323	-0.17	1.02E-13
4349	cg00275232	ZNF342	0.12	5.84E-04
4350	cg10224037	ZNF354A	0.12	1.69E-04
4351	cg11538128	ZNF354C	0.19	9.72E-08
4352	cg04488521	ZNF354C	0.11	6.98E-04
4353	cg15591678	ZNF365	0.12	2.61E-05
4354	cg16449972	ZNF423	-0.20	2.26E-15
4355	cg02238504	ZNF423	-0.22	2.44E-17
4356	cg18129786	ZNF445	-0.32	7.90E-19
4357	cg18888520	ZNF447	0.14	5.33E-05
4358	cg23037403	ZNF454	0.18	2.73E-07
4359	cg01184522	ZNF496	0.33	2.93E-11
4360	cg18793806	ZNF514	0.17	2.82E-10
4361	cg18680834	ZNF536	-0.13	3.36E-13
4362	cg03555203	ZNF537	-0.21	1.45E-17
4363	cg08458170	ZNF537	-0.33	2.02E-19
4364	cg03975694	ZNF540	0.40	3.18E-25
4365	cg27389185	ZNF540	0.27	8.37E-19
4366	cg04391540	ZNF541	-0.12	1.94E-04
4367	cg25886284	ZNF545	0.29	1.45E-10
4368	cg07054095	ZNF549	0.14	9.86E-07
4369	cg06458239	ZNF549	0.13	2.11E-05
4370	cg16014085	ZNF553	0.23	2.48E-17
4371	cg05221167	ZNF560	0.27	4.00E-15
4372	cg04062391	ZNF560	0.20	2.25E-10
4373	cg23968383	ZNF572	-0.18	1.55E-06
4374	cg11719283	ZNF574	-0.13	8.63E-07
4375	cg16731240	ZNF577	0.13	2.93E-03
4376	cg24333473	ZNF597	0.12	3.88E-10
4377	cg12259537	ZNF606	0.19	2.05E-10
4378	cg07703337	ZNF610	-0.19	1.21E-13
4379	cg15062535	ZNF610	-0.20	1.08E-15
4380	cg25776555	ZNF614	0.10	8.77E-05
4381	cg17892556	ZNF625	0.28	1.61E-08
4382	cg18267381	ZNF659	0.12	4.93E-04
4383	cg22598028	ZNF660	0.34	3.93E-14
4384	cg05508084	ZNF667	0.25	1.65E-12
4385	cg03289872	ZNF667	0.13	6.80E-05
4386	cg23857226	ZNF671	0.18	9.43E-06
4387	cg19246110	ZNF671	0.14	1.04E-04
4388	cg18335068	ZNF677	0.10	1.69E-04
4389	cg10705251	ZNF683	-0.11	9.58E-07
4390	cg20845050	ZNF7	-0.12	1.60E-11
4391	cg18440048	ZNF70	0.12	1.28E-05
4392	cg02440177	ZNF702	0.33	2.98E-14
4393	cg07559730	ZNF702	0.16	1.55E-07
4394	cg13334054	ZNF80	-0.11	1.19E-14
4395	cg03109316	ZNF80	-0.37	2.87E-28

4396	cg04386405	ZNF83	0.20	1.25E-12
4397	cg07706362	ZNF91	0.11	4.15E-15
4398	cg07660236	ZNF96	0.25	3.17E-16
4399	cg02622316	ZNF96	0.17	2.47E-06
4400	cg06590610	ZNFN1A1	-0.22	2.09E-11
4401	cg03872376	ZP4	-0.22	3.74E-22
4402	cg27094076	ZPBP	0.12	1.90E-07
4403	cg27183007	ZPBP	0.12	1.38E-08
4404	cg21331821	ZSCAN1	0.13	1.81E-04
4405	cg27276456	ZSCAN4	-0.20	1.51E-13
4406	cg22262964		-0.13	5.01E-19
4407	cg14804557		-0.14	2.26E-12
4408	cg00718513		-0.14	3.99E-18
4409	cg11560645		-0.15	1.19E-08
4410	cg27348440		-0.15	8.79E-10
4411	cg16878021		-0.18	4.07E-13
4412	cg23663653		-0.20	1.03E-15
4413	cg23988567		-0.20	4.97E-16
4414	cg15051063		-0.22	1.58E-10
4415	cg10883352		-0.23	8.36E-13
4416	cg23984130		-0.25	4.51E-22
