# Supplementary material for: Methylation Profiles Reveal Distinct Subgroup of Hepatocellular Carcinoma Patients with Poor Prognosis
Source: PLoS One. 2014 Aug 5;9(8):e104158. doi: 10.1371/journal.pone.0104158 (PMC4122406; doi:10.1371/journal.pone.0104158)
Supplement: Table S6 — IPA results for top biological functions enriched in differentially methylated dataset. (PDF) [file pone.0104158.s011.pdf]

Table S6. IPA results for top biological functions enriched in differentially methylated dataset.

Category	B-H adjusted p-Value	No. of Molecules	Molecules
Cell-To-Cell Signaling and Interaction	1.28E-15	307	ADCYAP1,ADORA1,ADORA2A,ADORA3,ADRA1A,ADRA1D,ADRA2B,ADRA2C,AGT,AKAP10,AKAP12,AKT3,ALK,AMH,APLNR,APOC3,ARHGEF7,ASB2,ATM,BAI1,BCAR3,BSG,C1QA,CABP2,CALCA,CALCB,CASP1,CASP10,CASP2,CASP8,CASR,CCK,CCL1,CCL11,CCL13,CCL16,CCL18,CCL20,CCL26,CCL4,CCL7,CCL8,CCR10,CCR9,CD160,CD19,CD226,CD33,CD44,CD79B,CDC42EP3,CDKL2,CEACAM6,CELSR3,CFTR,CGA,CGB (includes others),CHRM1,CHRNA2,CHRNA4,CHRNB3,CHRNB4,CHRND,CHST4,CLEC1A,CLEC5A,CLTCL1,CMKLR1,CNGA3,CRABP1,CRH,CRHR1,CSF1R,CXCL12,CXCL5,CXCL6,CXCR1,CXCR2,CXCR4,CYTL1,DDR2,DEFB4A/DEFB4B,DKKL1,DOK1,EDN3,EDNRB,EFNB2,EFNB3,EFS,EGF,EMR3,EPHA2,ERBB4,ESR1,ESR2,F2,F2RL1,FCGR1A,FFAR1,FFAR2,FFAR3,FGF17,FGF20,FGF21,FGF6,FSHB,FSHR,GABBR2,GABRA5,GABRA6,GAS6,GATA3,GDF5,GDNF,GFRA3,GHRH,GHSR,GIP,GJA3,GNA11,GNA14,GNAL,GNG12,GNRH2,GNRHR,GPR1,GPR114,GPR115,GPR116,GPR12,GPR123,GPR124,GPR25,GPR27,GPR32,GPR35,GPR6,GRAP2,GRIA2,GUCY1A2,GULP1,HCAR3,HCLS1,HCN2,HGF,HRH1,HTR2B,HYAL2,IFITM1,IFNB1,IFNG,IGF1R,IGFALS,IL10RA,IL12RB2,IL3,IL36G,IL5RA,IMPA1,INHA,INS,INSL3,INSL4,INSR,ITPKB,KALRN,KCNIP1,KCNIP2,KLRC1,KLRD1,KRT17,LALBA,LAT2,LAX1,LGALS3BP,LIFR,LILRA1,LILRA2,LILRA3,LILRB2,LILRB3,LILRB4,LILRB5,LIMK1,LPAR2,LPXN,LRRN2,LTA,LTB4R,LTBR,LTK,MADD,MAGI2,MARCO,MC2R,MCAM,MIF,MKNK2,MLN,MLNR,MPP2,MS4A2,MST1R,NCR2,NFAM1,NLRP12,NMBR,NMUR1,NMUR2,NPB,NPBWR2,NPY5R,NR1I3,NR2E3,NR4A1,NRP2,NTF3,NTSR1,OPRD1,OR1D2,OR1E2,OXT,OXTR,P2RX1,PCDH8,PCSK1,PDCD1,PDGFRB,PDPK1,PENK,PGF,PLAU,PLXNB1,PNOC,POMC,PPP1R17,PRKAR2B,PRKCH,PRKD1,PRKG2,PSTPIP1,PTGER2,PTH,PTH2R,PTK7,PTPN6,RAC2,RALBP1,RASGRF1,RASGRP2,RCVRN,RGR,RHEB,RHO,RIPK3,RIT2,RND2,ROR2,RTKN,S100A9,SCTR,SECTM1,SEMA3B,SEMA5A,SFN,SFRP5,SH2D2A,SH3BP2,SH3GL3,SIPA1,SIRPB1,SNTG1,SRD5A2,SRI,SST,SSTR3,TAAR5,TAAR6,TACSTD2,TANK,TBX5,TGFBR1,TLE1,TLK2,TMEM102,TNF,TNFRSF10A,TNFRSF10C,TNFRSF13B,TNFSF4,TNFSF9,TOLLIP,TREM1,TRH,TRHR,TRIM13,TSPAN32,TYROBP,UCN,UTS2R,VIPR2,VLDLR,WISP1,WISP3,WNT8B,XCR1,ZP4
Cellular Movement	1.52E-15	337	ABI3,ADORA1,ADORA2A,ADORA3,AGR2,AGT,AJAP1,AKT3,ALOX12,ALPP,ANGPT4,ANPEP,APC,APLNR,ARHGAP8/PRR5-ARHGAP8,ARHGDIB,ARHGEF4,ATP1A4,BCAN,BCAR3,BDKRB1,BMP4,BMP6,BMP7,BSG,BTC,CABYR,CALCA,CARD10,CCK,CCL1,CCL11,CCL13,CCL14,CCL16,CCL18,CCL20,CCL26,CCL3L1/CCL3L3,CCL4,CCL7,CCL8,CCND1,CCR3,CD226,CD38,CD44,CD48,CD86,CDCP1,CDH1,CDH13,CDH3,CDH5,CDKN2A,CDX2,CEACAM1,CGB (includes others),CHFR,CHGA,CHN2,CHST2,CLCA2,CLDN4,CLRN1,CMKLR1,CMTM8,CNR1,COL7A1,CRMP1,CSF1R,CSF2,CSF3,CSF3R,CSH1/CSH2,CTBP2,CTSG,CTSZ,CXADR,CXCL12,CXCL5,CXCL6,CXCR1,CXCR2,CXCR4,DARC,DDR2,DEFA1 (includes others),DEFA5,DEFB103A/DEFB103B,DEFB4A/DEFB4B,DKK3,DNAJB6,DOCK2,DOK1,DSE,EBI3,EDN2,EDN3,EDNRB,EFNA5,EFNB2,EFNB3,EGF,ENG,EPHA2,EPHB3,EPO,EPOR,ERBB4,ESR1,ESR2,F10,F2,F2R,F2RL1,F7,FAM65B,FBLIM1,FBLN2,FBXL2,FCGR3A,FERMT1,FGF1,FGF2,FGF7,FGF8,FGFR1,FHL2,FHOD1,FLNC,FLT4,FOSL1,FOXC1,FOXD3,FSCN1,FYN,GATA3,GDF2,GDNF,GH1,GHSR,GNA11,GNAS,GPLD1,GPR132,GRB7,GZMB,HAS1,HAVCR2,HDGF,HES6,HGF,HLA-G,HOXA1,HOXA4,HOXA7,HOXA9,HTN1,HYAL2,ICAM2,IFNB1,IFNG,IGF1R,IGF2,IGF2BP1,IGFBP5,IL10,IL16,IL18BP,IL1A,IL20,IL21R,IL3,INS,INSR,ITGA3,ITGA9,ITGB2,ITGB8,KANK1,KCNMA1,KCNN4,KDR,KISS1,KL,KLF2,KLF4,KLK3,KRT16,KRT2,LAMC2,LEP,LGALS3,LIF,LIFR,LIMK1,LIMS1,LMO7,LOX,LPAR2,LPAR3,LRPAP1,LSP1,LTA,LTK,LY6D,MATK,MCAM,MCF2L,MIF,MMP14,MMP3,MMP9,MSR1,MST1R,MUC4,MYLK,MYO10,NCKAP1L,NDRG2,NES,NF1,NOS3,NOTCH4,NOX4,NR4A1,NRP2,OPRD1,P2RY2,PAEP,PCSK6,PDE4DIP,PDGFRB,PENK,PGF,PIK3CD,PIK3R5,PLA2G7,PLAU,PLCL1,PLXNB1,PLXND1,PMP22,PODN,PODXL2,PPBP,PPM1F,PRKCB,PRKD1,PRKG1,PROC,PROCR,PROK2,PTGDR,PTGS2,PTH,PTP4A3,PTPRO,PVR,RAC2,RALBP1,RARRES2,RASSF1,RECK,REN,RLN2,RNASE2,ROR2,RUNX3,S100A10,S100A12,S100A8,S100A9,S100B,SCGB3A1,SCNN1A,SCNN1G,SDC4,SELPLG,SEMA3F,SEMA4D,SERPINB3,SERPINB5,SERPINC1,SERPIND1,SFN,SFRP2,SFRP4,SFTPD,SH2D2A,SLC22A16,SLIT2,SNAI1,SOCS3,SPARC,SPDEF,SST,ST6GAL1,STMN3,SYK,SYNM,TACR1,TBX5,TFAP2A,TFF1,TFF2,TFF3,TGFBR1,THBS4,THY1,TIAM1,TIMP3,TLE6,TMOD3,TNF,TNFRSF1B,TNFSF11,TNFSF9,TNK2,TNP1,TP73,TPM3,TRH,TUBB2B,TWIST1,VASH1,VAV3,VHL,VIM,VTN,VWF,WASF3,WISP1,WNT11,ZBTB16,ZSCAN1
Cellular Function and Maintenance	7.88E-11	89	ADCYAP1,ATP1A4,BCL2,BSG,CACNA1C,CALCA,CALCB,CALCR,CASR,CCKAR,CCL1,CCL11,CCL13,CCL14,CCL16,CCL20,CCL3L1/CCL3L3,CCL4,CCL7,CCL8,CD19,CD38,CD8A,CEACAM3,CFTR,CHGA,CHRM1,CXCL12,CXCL5,CXCR4,DEFA1 (includes others),DEFA5,DEFB103A/DEFB103B,DEFB4A/DEFB4B,DRD5,EDN2,EGF,F2,F2R,F2RL1,F7,FCGR1A,FCGR3A,FCGR3B,FGF23,FKBP1B,FTMT,FXYD2,FYN,GCM2,GDNF,GLP1R,GPR132,HRH3,ITGB2,KCNMA1,KCNQ1,LAX1,LCP2,LPAR2,LPAR3,MMP1,MS4A1,NMUR2,OSCAR,P2RY2,PENK,PLCE1,PLCG2,PPBP,PROK2,RGS2,RHAG,RHCG,RYR2,SCGN,SLC11A1,SLC24A2,SLC8A1,TFR2,TNF,TPCN2,TRPA1,TRPC1,TRPC3,TRPM2,TRPV4,VWF,XCR1
Molecular Transport	4.00E-09	80	ADCYAP1,ADRA1A,AGT,APOC3,BDKRB1,CACNA1D,CALCR,CASR,CCK,CCL1,CCL3L1/CCL3L3,CCL4,CCL7,CCR10,CCR3,CCR9,CD8A,CSF2,CXCL12,CXCR1,CXCR2,CXCR4,EDNRB,EGF,EPO,F2,F2R,F2RL1,FCGR3A,FCGR3B,FFAR1,FGF1,FGF2,GH1,GNA11,GNRH2,HGF,HTR7,IFNG,IL3,INS,LAT2,LILRB4,LTA,MCAM,MLN,MRVI1,NMUR1,NMUR2,NOS3,NPY,OR3A1,OXT,OXTR,P2RY2,PLAU,PLCL1,POMC,PPBP,PRKCB,PRKG1,PRND,PROC,PTH,RASGRP3,RYR2,S100A1,S100A8,S100A9,SPINT2,TACR1,TNF,TNFSF11,TP73,TPSAB1/TPSB2,TRH,TRHR,TRPC1,TRPC3,TRPV4
Vitamin and Mineral Metabolism	4.00E-09	80	ADCYAP1,ADRA1A,AGT,APOC3,BDKRB1,CACNA1D,CALCR,CASR,CCK,CCL1,CCL3L1/CCL3L3,CCL4,CCL7,CCR10,CCR3,CCR9,CD8A,CSF2,CXCL12,CXCR1,CXCR2,CXCR4,EDNRB,EGF,EPO,F2,F2R,F2RL1,FCGR3A,FCGR3B,FFAR1,FGF1,FGF2,GH1,GNA11,GNRH2,HGF,HTR7,IFNG,IL3,INS,LAT2,LILRB4,LTA,MCAM,MLN,MRVI1,NMUR1,NMUR2,NOS3,NPY,OR3A1,OXT,OXTR,P2RY2,PLAU,PLCL1,POMC,PPBP,PRKCB,PRKG1,PRND,PROC,PTH,RASGRP3,RYR2,S100A1,S100A8,S100A9,SPINT2,TACR1,TNF,TNFSF11,TP73,TPSAB1/TPSB2,TRH,TRHR,TRPC1,TRPC3,TRPV4
Nucleic Acid Metabolism	5.86E-08	56	ADCY5,ADCYAP1,ADORA2A,ADRB1,AKAP12,APLP1,AQP1,BMP4,BMP6,BMP7,CALCA,CALCR,CASR,CGA,CGB (includes others),CRH,CRHR1,CXCR4,DRD1,DRD2,DRD4,F2,FSHR,GHRH,GIP,GLP1R,GLP2R,GNAS,GNRHR,GPR12,GPR6,GRM8,HRH3,HTR2B,IFNG,INS,INSL3,MC2R,MC3R,MC4R,MTNR1A,NOS3,NPY,POMC,PTGER2,PTGS2,PTH,PTH2R,RAMP2,RAMP3,RGS10,RGS17,RGS2,SCTR,SST,TNF
Small Molecule Biochemistry	5.86E-08	56	ADCY5,ADCYAP1,ADORA2A,ADRB1,AKAP12,APLP1,AQP1,BMP4,BMP6,BMP7,CALCA,CALCR,CASR,CGA,CGB (includes others),CRH,CRHR1,CXCR4,DRD1,DRD2,DRD4,F2,FSHR,GHRH,GIP,GLP1R,GLP2R,GNAS,GNRHR,GPR12,GPR6,GRM8,HRH3,HTR2B,IFNG,INS,INSL3,MC2R,MC3R,MC4R,MTNR1A,NOS3,NPY,POMC,PTGER2,PTGS2,PTH,PTH2R,RAMP2,RAMP3,RGS10,RGS17,RGS2,SCTR,SST,TNF

Cellular Growth and Proliferation	1.97E-07	588	ABCB5,ACSL5,ACTG1,ADAM12,ADAMTS15,ADAMTS8,ADCY3,ADCYAP1,ADRA1A,ADRA1D,AGT,AHSG,AIM2,AKAP12,AKR1B10,AKT3,ALDH1A2,ALK,ALOX12,ALOX15,ALOX5,AMH,ANPEP,APC,APCDD1,APLNR,AQP1,ARHGEF1,ASB2,ATM,ATP5G2,ATP8A2,AXIN1,BAI1,BAK1,BCAR3,BCAT1,BCL2,BDKRB1,BIK,BMP4,BMP6,BMP7,BMPER,BNC1,BNIP3,BPI,BRF1,BSG,BTC,BTG3,BTG4,C9orf78,CA3,CACNA1G,CALCA,CALCB,CALCR,CARD10,CASP1,CASP2,CASP8,CASR,CBFA2T3,CBS,CCDC8,CCKAR,CCL11,CCL14,CCL18,CCL20,CCL26,CCL3L1/CCL3L3,CCL4,CCND1,CCND2,CD160,CD19,CD1D,CD209,CD248,CD33,CD37,CD38,CD44,CD6,CD80,CD86,CD8A,CDA,CDCA7,CDCP1,CDH1,CDH13,CDH5,CDK10,CDK20,CDK3,CDK5R1,CDKN1C,CDKN2A,CDKN2B,CDX2,CEACAM1,CEACAM6,CEBPE,CGREF1,CHFR,CHGA,CHMP1A,CHN2,CHRM1,CLCA2,CNOT8,COL14A1,COL2A1,COL6A2,COL6A3,CR1,CRH,CRIP1,CSF1R,CSF2,CSF2RB,CSF3,CSF3R,CSH1/CSH2,CSRP3,CTBP2,CTLA4,CTSZ,CXADR,CXCL12,CXCL5,CXCR1,CXCR2,CXCR4,CYP19A1,DAB2IP,DAP,DAPK2,DBN1,DCC,DCD,DEFA1 (includes others),DEGS1,DEGS2,DES,DIRAS3,DKK3,DLEC1,DLK1,DLX5,DNAJB1,DPH1,DUSP2,DUSP4,EAPP,EBI3,EDN2,EDNRB,EFEMP1,EFNB2,EGF,EGR2,EGR3,EHF,EIF5A2,ELANE,EMILIN2,ENG,ENPP7,EPB41L3,EPCAM,EPHA2,EPHA5,EPHB3,EPO,EPOR,ERBB4,ESM1,ESR1,ESR2,ETV7,F12,F2,F2R,F2RL1,F7,FABP1,FABP6,FADS2,FBLN1,FBLN2,FBN1,FBN2,FBXL2,FCER2,FERMT1,FES,FGF1,FGF19,FGF2,FGF6,FGF7,FGF8,FGFBP1,FGFR1,FHL2,FKBP1B,FLT3,FLT4,FOLR1,FOSL1,FOXC1,FOXG1,FRZB,FSCN1,FTMT,FUT4,FUT8,FYN,FZD7,FZD9,GABRP,GALR1,GAS2L1,GAS6,GATA2,GATA4,GDF2,GDF5,GDNF,GFI1,GH1,GHRH,GJB2,GLP2R,GLTSCR2,GML,GNAS,GNG4,GNMT,GNRH2,GNRHR,GPLD1,GPNMB,GPR12,GPX1,GPX3,GRB7,GREM1,GSTP1,GZMB,H2AFY,HAND1,HAS1,HAVCR2,HDAC3,HDGF,HES6,HEXIM1,HGF,HGFAC,HK1,HK2,HMGA1,HNF4A,HOXA1,HOXA11,HOXA5,HOXA7,HOXA9,HOXC10,HPSE,HRH2,HSPB8,HTN1,HTR2B,HYAL1,HYAL2,ICAM3,IFNA17,IFNA8,IFNB1,IFNG,IFNL1,IFNL3,IGF1R,IGF2,IGF2BP1,IGFBP5,IGFBP7,IKBKE,IL10,IL10RA,IL12RB2,IL1A,IL22,IL23A,IL24,IL3,IL5RA,INHA,INS,INSL4,INSR,IRF4,ITGA2B,ITGA3,ITGB8,KCNK3,KCNMA1,KCNN4,KDR,KISS1,KLF2,KLF4,KLK3,KLK4,KRT14,KRT16,KRT2,LACRT,LAIR1,LAMC3,LEP,LEPREL1,LGALS3,LGALS4,LGI1,LIF,LIFR,LILRB2,LILRB4,LIMK1,LOX,LPAR2,LPAR3,LST1,LTA,LTBR,LTF,LTK,LY75,LY86,LZTS1,MADD,MAP3K14,MATK,MBP,MCAM,MEST,MIF,MMP14,MMP9,MNDA,MOG,MS4A1,MS4A2,MSH3,MSLN,MST1R,MSX1,MT3,MUC4,MYF6,MYL2,MYL9,MYOCD,NCKAP1L,NCSTN,NDN,NDRG2,NEIL1,NES,NF1,NGFR,NOS1,NOS3,NOTCH3,NOX4,NPY,NQO1,NR1H3,NR4A1,NRP2,NTRK3,NTS,OGFR,OXT,OXTR,PAEP,PAX9,PDCD1,PDGFRB,PDK4,PDPK1,PDXK,PDZK1,PENK,PFKP,PGF,PIK3CD,PLA2G3,PLAT,PLAU,PLCD1,PLCE1,PLCG2,PLK1,PLXNA4,PLXNB1,PMEPA1,PMP22,PODN,POMC,POU2F3,POU4F1,POU4F2,PRAME,PRDM14,PRDM16,PRG2,PRKACG,PRKAR2B,PRKCB,PRKCH,PRKD1,PRKG1,PROC,PROCR,PROK2,PRR5,PRSS2,PRTN3,PTGER2,PTGES,PTGS2,PTH,PTP4A3,PTPN6,PTPRE,PTPRN,PTPRO,PXDN,QPCT,QSOX1,RAB25,RAC2,RASSF1,RASSF5,RB1,RBFOX2,RBP3,RCVRN,RECK,REG1A,REG1B,REG3A,RERG,RETN,RHOJ,RLN2,RNASE3,ROR2,RRAD,RUNX3,RYR2,S100A10,S100B,SALL4,SCGB3A1,SCGN,SCTR,SCUBE3,SEMA3B,SEMA6A,SERPINB2,SERPINB4,SERPINB5,SERPINC1,SERPIND1,SERPINH1,SFN,SFRP2,SFRP4,SFRP5,SFTPD,SIPA1,SIX3,SLC12A4,SLC22A1,SLC22A18,SLC26A2,SLC26A3,SLC2A11,SLC7A11,SLC8A1,SLIT2,SLURP1,SMPD3,SNAI1,SOCS1,SOCS3,SOST,SOX11,SOX17,SOX2,SPAM1,SPARC,SPDYA,SPEG,SPINK7,SPINT2,SPRR3,SST,SSTR3,ST14,STAT5A,STMN3,STUB1,SULT2A1,SUMO3,SYK,SYNM,TACSTD2,TAL1,TBC1D3 (includes others),TBX2,TBX5,TCL1A,TERT,TFAP2A,TFF3,TFPI2,TGFBR1,TGFBR3,THBS4,TIAM1,TIGIT,TIMP3,TJP3,TMC8,TNF,TNFRSF10A,TNFRSF13B,TNFRSF19,TNFRSF1B,TNFRSF4,TNFRSF9,TNFSF4,TNFSF9,TNK2,TP73,TPO,TPSD1,TRH,TRPC1,TRPC3,TSLP,TUBB3,TWIST1,UBE2L3,UBE2V2,UCHL1,UCN,ULBP1,ULBP2,USP4,VASH1,VAV1,VAV3,VHL,VTN,WIF1,WISP1,WNK2,WNT10B,WNT2,WNT7A,WT1,ZBTB16,ZEB1,ZMYM2,ZP4,ZSCAN1,ZSCAN18
Cellular Development	2.43E-06	90	ANPEP,ATM,BCL2,BMP4,BSG,CALCB,CCL20,CCL4,CD160,CD19,CD1D,CD209,CD33,CD37,CD38,CD6,CD80,CD86,CD8A,CDKN1C,CDKN2A,CEACAM1,CR1,CSF1R,CSF2,CSF2RB,CSF3,CSF3R,CTLA4,CTSZ,CXCL12,EBI3,EPO,FCER2,FLT3,GATA2,GH1,HAVCR2,HMGA1,HOXA5,HOXA9,HRH2,IFNG,IGF2,IL10,IL10RA,IL23A,IL3,ITGA2B,KCNN4,KLF4,LAIR1,LEP,LGALS4,LILRB2,LILRB4,LST1,LTA,LY75,MBP,MIF,MNDA,MS4A1,NCKAP1L,PAEP,PDCD1,PTPN6,PTPRN,PTPRO,RAC2,RBP3,SFTPD,SOCS3,STAT5A,TCL1A,TERT,TIGIT,TNF,TNFRSF13B,TNFRSF1B,TNFRSF4,TNFRSF9,TNFSF9,TSLP,ULBP1,ULBP2,VAV3,ZBTB16,ZMYM2,ZP4
Cell Death and Survival	1.19E-05	30	CCL11,CCL13,CCL7,CCR3,CD1D,CD226,CD38,CD6,CEACAM1,CLEC12B,CSF2,FCGR1A,FCGR3B,HAVCR2,IFNG,ITGB2,KIR3DL1,KLRC1,KLRD1,LAIR1,NCR2,PRF1,PTGDR,PTPN6,SIGLEC7,SYK,TNF,ULBP1,ULBP2,ULBP3
