# Supplementary material for: Methylation Profiles Reveal Distinct Subgroup of Hepatocellular Carcinoma Patients with Poor Prognosis
Source: PLoS One. 2014 Aug 5;9(8):e104158. doi: 10.1371/journal.pone.0104158 (PMC4122406; doi:10.1371/journal.pone.0104158)
Supplement: Table S7 — 536 genes with aberrant methylation and associated change of expression. (PDF) [file pone.0104158.s012.pdf]

Table S7. 536 genes with aberrant methylation and associated change of expression.

No.	SYMBOL	Infinium Probeset ID	Methylation Difference (T-NT)	FDR adjusted p-value (Methylation)	Illumina probeset ID	Expression Fold Change (T/NT)	FDR adjusted p-value (Expression)
1	TSPYL5	cg15747595	0.38	2.47E-39	ILMN_1737972	-1.32	4.24E-08
2	LDHB	cg06437004	0.49	5.31E-32	ILMN_1728132	-1.22	2.47E-02
3	SH3YL1	cg21825027	0.56	3.83E-29	ILMN_1712231	-1.43	8.81E-09
4	CYB5R2	cg03826976	0.30	3.13E-25	ILMN_1739576	-1.34	9.62E-05
5	SPINT2	cg15375239	0.50	6.56E-24	ILMN_1800739	-1.77	9.87E-05
6	TCTEX1D1	cg24110050	0.34	1.07E-23	ILMN_1751868	-1.59	4.54E-07
7	GRASP	cg04034767	0.45	4.36E-23	ILMN_1705210	-1.48	4.69E-09
8	DOK1	cg26117023	0.45	2.56E-22	ILMN_1700086	-1.20	1.78E-04
9	KRTCAP3	cg11618577	0.24	2.90E-22	ILMN_1700728	-1.86	2.88E-11
10	PBX4	cg19996355	0.37	2.87E-21	ILMN_1700762	-1.21	3.03E-04
11	ULK2	cg12324629	0.18	3.70E-21	ILMN_1715482	-1.21	9.48E-07
12	GSTP1	cg04920951	0.39	7.19E-21	ILMN_1679809	-1.84	1.70E-08
13	SRD5A2	cg15403517	0.31	1.77E-20	ILMN_1788895	-3.85	1.18E-16
14	ADRA2B	cg21542793	0.37	2.47E-20	ILMN_1677868	-1.38	6.65E-13
15	PODN	cg23092823	0.29	1.24E-18	ILMN_1770800	-1.29	1.87E-04
16	RAB31	cg17982102	0.36	3.68E-18	ILMN_1660691	-1.24	7.13E-03
17	CFTR	cg25509184	0.33	1.14E-17	ILMN_1705813	-2.03	3.09E-11
18	GNA14	cg17301902	0.32	6.50E-17	ILMN_1686227	-1.36	8.36E-14
19	RNF135	cg00948524	0.33	8.97E-17	ILMN_1700660	-1.24	1.69E-07
20	NR1H3	cg12613344	0.20	1.46E-16	ILMN_1814022	-1.33	3.12E-08
21	HGFAC	cg07364841	0.19	2.82E-15	ILMN_1701468	-8.28	1.83E-22
22	WNT11	cg21238457	0.26	2.99E-15	ILMN_1662188	-1.36	3.79E-05
23	TNFAIP2	cg20368904	0.15	1.57E-14	ILMN_1727689	-1.22	3.72E-03
24	MASP1	cg21831174	0.14	2.44E-14	ILMN_1809537	-2.65	7.33E-23
25	LEP	cg12782180	0.25	3.57E-14	ILMN_2207505	-1.36	8.34E-06
26	GALNT14	cg05714219	0.32	4.22E-13	ILMN_1722524	-1.40	5.32E-12
27	APCDD1	cg19264571	0.18	4.22E-13	ILMN_1656951	-1.35	3.00E-04
28	SLC16A5	cg27619475	0.15	1.04E-12	ILMN_1755649	-1.37	9.52E-07
29	CD8A	cg17108819	0.33	1.32E-12	ILMN_1760374	-1.23	8.09E-04
30	ALDH1A3	cg19177941	0.26	2.36E-12	ILMN_1807439	-1.47	2.12E-06
31	SOCS2	cg04797323	0.35	2.82E-12	ILMN_1798926	-2.58	6.86E-14
32	SOX8	cg21530890	0.29	3.62E-12	ILMN_1789244	-1.27	7.02E-07
33	CHST4	cg00840403	0.15	3.76E-12	ILMN_1696590	-2.33	5.47E-12
34	CYGB	cg17040807	0.17	4.58E-12	ILMN_1758128	-1.59	3.69E-13
35	MYO10	cg07862358	0.30	6.02E-12	ILMN_2232712	-1.30	9.20E-11
36	CDKN1C	cg20919799	0.34	6.17E-12	ILMN_1718565	-1.35	1.09E-03
37	PTGS2	cg13986130	0.19	2.08E-11	ILMN_2054297	-2.50	6.04E-12
38	ST6GAL1	cg15928398	0.20	2.33E-11	ILMN_2384496	-1.32	6.44E-04
39	LRRC3	cg23089840	0.17	3.86E-11	ILMN_1795055	-1.33	3.32E-11
40	AKAP2	cg15083233	0.21	4.35E-11	ILMN_1675507	-1.40	1.87E-09
41	TMEM125	cg04355435	0.24	4.46E-11	ILMN_1685709	-1.22	1.04E-04
42	TACSTD2	cg16080552	0.20	1.05E-10	ILMN_1739001	-3.41	1.78E-11
43	HCLS1	cg00141162	0.20	1.14E-10	ILMN_1727402	-1.67	1.72E-05
44	SPATA18	cg09022993	0.21	1.21E-10	ILMN_1667948	-1.86	2.94E-15
45	ESPN	cg13066963	0.22	1.71E-10	ILMN_1806710	-1.39	2.46E-05
46	RBP1	cg23363832	0.28	2.43E-10	ILMN_1656837	-2.92	2.65E-10
47	HCN2	cg06005396	0.17	3.02E-10	ILMN_1659150	-1.21	1.53E-05
48	LTK	cg27494383	0.19	3.73E-10	ILMN_1799871	-1.29	1.71E-10
49	CD160	cg12832565	0.11	3.99E-10	ILMN_1742001	-1.32	3.98E-10
50	SLC22A1	cg27292431	0.18	6.85E-10	ILMN_1715742	-5.52	4.66E-16
51	TUBB6	cg07307078	0.24	1.05E-09	ILMN_1702636	-1.22	3.37E-03
52	SLC25A36	cg25152631	0.26	1.29E-09	ILMN_1766528	-1.20	6.72E-04
53	FGL2	cg12271671	0.15	1.76E-09	ILMN_1693009	-1.63	1.21E-08
54	SPG20	cg18755783	0.23	2.41E-09	ILMN_1801236	-1.27	2.57E-09

55	TCF21	cg24215443	0.20	2.89E-09	ILMN_1766712	-1.28	3.14E-09
56	ESR1	cg07671949	0.22	2.90E-09	ILMN_1678535	-2.73	2.20E-20
57	CFH	cg24974599	0.11	3.45E-09	ILMN_1810910	-1.56	8.03E-05
58	RCN3	cg04378886	0.12	3.83E-09	ILMN_1809850	-1.21	1.73E-05
59	FES	cg18661868	0.20	3.88E-09	ILMN_1693650	-1.68	2.21E-09
60	SLC7A5	cg26907768	0.16	4.14E-09	ILMN_1720373	-1.27	7.47E-03
61	ALOX5	cg18809289	0.20	4.23E-09	ILMN_1792150	-1.38	3.08E-08
62	UGT3A1	cg23317501	0.13	4.49E-09	ILMN_2133038	-1.29	9.30E-03
63	GIMAP5	cg13043509	0.17	6.39E-09	ILMN_1769383	-1.21	1.01E-02
64	GFPT2	cg18602314	0.21	7.62E-09	ILMN_1709674	-1.21	8.95E-04
65	CSF3R	cg09088576	0.14	7.75E-09	ILMN_2371280	-1.78	7.17E-10
66	HOXA4	cg24169822	0.19	1.32E-08	ILMN_3251370	-1.20	5.99E-05
67	COX7A1	cg24335895	0.11	1.35E-08	ILMN_1662419	-1.34	3.35E-04
68	HCN4	cg02860342	0.17	1.49E-08	ILMN_1790810	-1.51	2.96E-11
69	GFOD1	cg00194146	0.25	1.92E-08	ILMN_1778240	-1.36	5.79E-04
70	CH25H	cg23410627	0.21	2.57E-08	ILMN_1741021	-1.64	4.59E-05
71	HEXIM1	cg04700814	0.13	3.45E-08	ILMN_1685115	-1.45	4.04E-11
72	IGFALS	cg01747665	0.13	4.03E-08	ILMN_2188966	-7.58	1.93E-27
73	LRAT	cg23587449	0.19	4.35E-08	ILMN_1673491	-1.29	3.14E-14
74	KCNJ10	cg05101920	0.16	4.55E-08	ILMN_1808272	-1.23	8.17E-07
75	LGALS3BP	cg11105610	0.12	4.82E-08	ILMN_1659688	-1.27	3.82E-02
76	CBS	cg22633722	0.21	6.88E-08	ILMN_1804735	-2.05	4.36E-09
77	EFEMP1	cg20786074	0.15	8.21E-08	ILMN_1735877	-1.40	2.92E-02
78	RASGRP2	cg14170423	0.14	1.05E-07	ILMN_2375319	-1.37	2.01E-05
79	LAG3	cg26956535	0.10	1.10E-07	ILMN_1813338	-1.35	1.50E-05
80	CXCL6	cg22670329	0.14	1.14E-07	ILMN_2161577	-1.30	8.97E-03
81	ITGA9	cg25870420	0.21	1.31E-07	ILMN_1813117	-1.35	3.01E-13
82	SNAI1	cg05403071	0.20	1.42E-07	ILMN_1758086	-1.36	4.93E-09
83	BMP6	cg03447931	0.23	1.49E-07	ILMN_1747650	-1.24	1.62E-06
84	ID4	cg00468146	0.20	1.89E-07	ILMN_1721758	-1.29	3.56E-05
85	RRAD	cg19428417	0.13	2.23E-07	ILMN_2186137	-1.25	1.86E-05
86	APOA5	cg02157083	0.14	2.65E-07	ILMN_1754055	-3.80	1.42E-13
87	SFRP5	cg06692050	0.14	3.07E-07	ILMN_1790026	-1.47	9.17E-14
88	BRSK2	cg14021073	0.15	3.08E-07	ILMN_1708496	-1.23	8.08E-03
89	AEBP1	cg02126753	0.15	4.32E-07	ILMN_1736178	-1.71	9.91E-06
90	PLAT	cg12091331	0.15	4.33E-07	ILMN_1738742	-1.25	6.94E-03
91	VSX1	cg06151165	0.18	6.38E-07	ILMN_1755734	-1.21	2.89E-06
92	ALPL	cg20645065	0.21	6.46E-07	ILMN_1701603	-2.03	2.02E-09
93	FYN	cg17100322	0.23	6.52E-07	ILMN_2249920	-1.31	1.45E-06
94	BTBD6	cg09450238	0.12	6.71E-07	ILMN_1744725	-1.38	9.67E-06
95	ENG	cg24910675	0.13	8.42E-07	ILMN_1760778	-1.53	3.14E-07
96	HAND2	cg01580681	0.19	8.67E-07	ILMN_2064902	-1.36	2.04E-14
97	EOMES	cg12858460	0.18	1.00E-06	ILMN_1760509	-1.36	8.68E-05
98	RAC2	cg18265887	0.16	1.17E-06	ILMN_1709795	-1.36	1.22E-02
99	RASSF2	cg19614321	0.15	1.27E-06	ILMN_1812139	-1.31	5.98E-08
100	TGFBR3	cg00563926	0.13	1.46E-06	ILMN_1784287	-1.53	6.68E-06
101	SYT9	cg01806928	0.15	1.86E-06	ILMN_1728496	-1.23	7.01E-07
102	BASP1	cg23496260	0.14	2.27E-06	ILMN_1651826	-2.15	1.63E-10
103	ABR	cg25374854	0.15	2.48E-06	ILMN_1657045	-1.22	1.04E-05
104	SGCB	cg23653712	0.16	2.80E-06	ILMN_2194467	-1.28	1.12E-05
105	BMPER	cg17561435	0.17	3.40E-06	ILMN_3307916	-1.43	3.27E-14
106	FEZ1	cg08221207	0.13	3.79E-06	ILMN_1779071	-1.53	1.03E-09
107	RECK	cg12717594	0.19	4.23E-06	ILMN_2067269	-1.21	4.66E-03
108	TMEM25	cg20001829	0.15	4.96E-06	ILMN_1651745	-1.40	2.23E-08
109	ZBTB16	cg25101936	0.13	5.05E-06	ILMN_2305407	-1.74	7.60E-09
110	EGR2	cg19355190	0.17	6.82E-06	ILMN_1743199	-2.28	1.50E-10
111	TSLP	cg15089387	0.15	7.11E-06	ILMN_1662884	-1.36	1.11E-06
112	TNK2	cg01962086	0.16	7.26E-06	ILMN_1669703	-1.33	7.52E-07
113	CTBP2	cg14540150	0.15	9.99E-06	ILMN_1781031	-1.35	5.60E-09
114	WASF3	cg07744166	0.12	1.08E-05	ILMN_1810797	-1.25	1.23E-02
115	PRKAR2B	cg13705707	0.12	1.13E-05	ILMN_1684850	-1.23	4.58E-08

116	METRN	cg11027330	0.13	1.28E-05	ILMN_1712583	-1.34	5.39E-04
117	MFAP4	cg13030582	0.10	1.50E-05	ILMN_1766914	-4.51	3.78E-19
118	EFNB3	cg01013324	0.14	1.56E-05	ILMN_1695606	-1.30	3.31E-13
119	TRPV4	cg19615059	0.18	1.61E-05	ILMN_2362346	-1.28	5.26E-10
120	AKAP12	cg01555431	0.17	1.83E-05	ILMN_1684836	-1.27	6.20E-04
121	TMEM121	cg23886551	0.14	2.03E-05	ILMN_1723145	-1.39	7.93E-12
122	HLF	cg04219321	0.18	2.08E-05	ILMN_1722829	-1.81	6.34E-09
123	TDO2	cg08121954	0.11	2.76E-05	ILMN_1716859	-2.78	2.65E-09
124	OAT	cg03853151	0.20	2.78E-05	ILMN_1654441	-2.50	3.54E-09
125	THRSP	cg18338296	0.14	2.84E-05	ILMN_1794844	-4.68	7.50E-10
126	TFPI2	cg23141855	0.11	3.26E-05	ILMN_2068104	-1.78	2.18E-15
127	FOXF1	cg25971347	0.15	4.02E-05	ILMN_1680973	-1.23	4.59E-07
128	LEPREL1	cg20270599	0.11	4.05E-05	ILMN_1657373	-2.15	4.84E-20
129	NOL4	cg06722216	0.11	4.06E-05	ILMN_2087629	-1.29	1.25E-04
130	KCNS3	cg20673481	0.15	5.32E-05	ILMN_2175112	-1.24	7.59E-03
131	KRT7	cg07007400	0.11	6.62E-05	ILMN_2163723	-1.25	1.69E-03
132	OXT	cg26955850	0.13	8.08E-05	ILMN_1735124	-2.13	1.03E-13
133	GRAMD3	cg08704509	0.14	9.36E-05	ILMN_2065690	-1.28	1.18E-06
134	SEMA6A	cg12928668	0.12	1.00E-04	ILMN_1713529	-1.45	1.99E-06
135	TMPRSS2	cg24901042	0.14	1.03E-04	ILMN_1791123	-1.99	7.74E-10
136	CYBRD1	cg06885524	0.13	1.20E-04	ILMN_1712305	-1.46	9.52E-05
137	C1QTNF1	cg24844534	0.13	1.52E-04	ILMN_2380237	-2.10	2.56E-13
138	FLRT2	cg17410236	0.14	1.79E-04	ILMN_1769615	-1.23	1.82E-07
139	RASSF5	cg02589695	0.15	2.20E-04	ILMN_2362902	-1.22	1.52E-05
140	PELI2	cg21099488	0.10	3.56E-04	ILMN_1780132	-1.27	4.01E-05
141	CCND1	cg09637363	0.10	3.79E-04	ILMN_1688480	-1.30	6.40E-03
142	PRSS8	cg13439730	0.10	4.18E-04	ILMN_1796461	-1.71	3.18E-09
143	EGR3	cg25811575	0.12	4.63E-04	ILMN_1722781	-1.32	6.93E-07
144	PROK2	cg08555612	0.11	7.63E-04	ILMN_1775257	-1.48	3.85E-05
145	B3GAT1	cg11038843	0.11	7.93E-04	ILMN_1794072	-1.71	1.45E-13
146	COL6A2	cg21513553	0.12	1.59E-03	ILMN_1765017	-1.25	3.71E-08
147	KLF4	cg07309102	0.13	1.61E-03	ILMN_1779857	-1.40	3.56E-06
148	SDPR	cg06352750	0.12	1.89E-03	ILMN_1715991	-1.56	1.08E-08
149	RGS2	cg20315136	0.11	2.19E-03	ILMN_2197365	-2.34	8.19E-09
150	TMEM51	cg05385377	0.11	2.27E-03	ILMN_1674985	-1.22	2.49E-02
151	DUSP2	cg05732530	0.10	2.29E-03	ILMN_1712959	-1.31	2.66E-06
152	KLF2	cg26842024	0.13	2.59E-03	ILMN_1735930	-1.53	3.20E-07
153	EPHA2	cg15146752	0.11	4.06E-03	ILMN_1699354	-1.79	1.34E-14
154	TNFRSF1B	cg26189983	0.10	6.82E-03	ILMN_1764788	-1.40	1.09E-04
155	NME4	cg18676162	0.11	7.22E-03	ILMN_1800634	-1.21	1.89E-02
156	SOCS3	cg27637521	0.13	1.12E-02	ILMN_1781001	-2.13	3.34E-09
157	TMEM106A	cg04482110	0.12	1.44E-02	ILMN_3249244	-1.42	1.20E-07
158	LHX2	cg07109287	0.10	2.13E-02	ILMN_1807016	-2.25	2.85E-15
159	MNDA	cg25119415	-0.24	1.50E-30	ILMN_1738992	-1.40	2.95E-07
160	LILRA3	cg00705255	-0.15	1.88E-30	ILMN_1661631	-1.24	1.20E-07
161	WFDC1	cg23865698	-0.27	1.91E-26	ILMN_1660808	-1.32	3.89E-08
162	S100A12	cg02813121	-0.36	7.10E-26	ILMN_1748915	-1.39	5.82E-08
163	CEACAM6	cg26813458	-0.33	4.38E-25	ILMN_1712522	-1.20	4.16E-02
164	CDA	cg13164309	-0.21	7.79E-24	ILMN_1714592	-1.82	3.33E-12
165	HBG1	cg01598642	-0.29	9.91E-23	ILMN_1796678	-1.30	2.03E-05
166	FCN1	cg06244417	-0.29	2.34E-22	ILMN_1668063	-1.48	8.75E-07
167	KCNQ1	cg12949760	-0.28	3.77E-22	ILMN_1656079	-1.23	1.62E-07
168	FAM107B	cg02876062	-0.28	1.79E-21	ILMN_1758672	-1.29	8.69E-06
169	C1QB	cg22477971	-0.25	6.96E-21	ILMN_1796409	-1.63	5.41E-05
170	S100A8	cg20070090	-0.21	1.09E-20	ILMN_1729801	-2.91	3.27E-11
171	HAVCR2	cg17484237	-0.18	1.77E-20	ILMN_1693826	-1.21	1.41E-02
172	OLFM1	cg08268099	-0.24	6.35E-20	ILMN_1742025	-1.48	4.51E-08
173	TREML1	cg01564343	-0.28	1.07E-19	ILMN_1690783	-1.47	1.98E-11
174	MARCO	cg02431964	-0.28	2.44E-19	ILMN_1731503	-4.54	3.10E-25
175	LILRB5	cg20649991	-0.27	8.01E-19	ILMN_2339294	-1.62	8.62E-14
176	C1QC	cg11393848	-0.11	8.71E-19	ILMN_1785902	-1.47	2.52E-04

177	CTSG	cg24355048	-0.17	9.49E-19	ILMN_1680424	-1.26	5.04E-03
178	LIFR	cg01796228	-0.21	1.67E-18	ILMN_1709094	-1.48	1.92E-16
179	TCN1	cg20018806	-0.20	1.78E-18	ILMN_1768469	-1.26	2.39E-02
180	DEFA3	cg22218909	-0.25	1.40E-17	ILMN_2165289	-1.78	1.94E-08
181	ABLIM3	cg05026186	-0.12	1.75E-17	ILMN_1656940	-1.50	3.00E-07
182	PRG2	cg15357945	-0.21	2.99E-17	ILMN_1729314	-1.32	6.38E-06
183	TFF2	cg11158374	-0.27	5.35E-17	ILMN_1663919	-1.31	1.68E-02
184	MX2	cg04828792	-0.27	5.47E-17	ILMN_2231928	-1.29	1.80E-04
185	TMEM86B	cg01076838	-0.25	5.66E-17	ILMN_2049417	-1.62	6.96E-11
186	FAM83F	cg03954858	-0.23	1.07E-16	ILMN_1683231	-1.33	1.84E-12
187	TRPM8	cg15746445	-0.18	1.81E-16	ILMN_1661637	-2.60	4.70E-13
188	LILRB3	cg22456522	-0.20	2.57E-16	ILMN_1784884	-1.46	1.35E-05
189	CASP1	cg13802966	-0.13	2.68E-16	ILMN_1727762	-1.31	3.97E-08
190	CLEC4E	cg22984277	-0.11	3.73E-16	ILMN_1771664	-1.23	2.88E-04
191	FCN2	cg26164184	-0.21	4.88E-16	ILMN_1714057	-2.48	1.87E-22
192	GRM8	cg02946850	-0.26	4.88E-16	ILMN_1729212	-1.46	2.09E-07
193	PVALB	cg02978737	-0.23	5.25E-16	ILMN_2069224	-1.40	2.24E-17
194	GZMH	cg22228134	-0.21	7.47E-16	ILMN_1731233	-1.34	4.68E-05
195	ACMSD	cg02812142	-0.16	1.36E-15	ILMN_1727091	-1.65	3.96E-06
196	AQP7	cg13246269	-0.14	5.89E-15	ILMN_1738494	-1.36	1.03E-07
197	OVGP1	cg09558502	-0.15	6.23E-15	ILMN_1734542	-1.30	5.24E-04
198	GPR1	cg19132372	-0.23	1.87E-14	ILMN_2107004	-1.40	2.96E-08
199	CBFA2T3	cg13745346	-0.21	4.23E-14	ILMN_1657627	-1.26	1.37E-09
200	GZMA	cg06118312	-0.16	4.65E-14	ILMN_1779324	-1.30	1.01E-02
201	SIGLEC11	cg24311282	-0.24	4.85E-14	ILMN_1674593	-1.50	4.70E-13
202	SGCA	cg04582295	-0.21	5.89E-14	ILMN_1741007	-1.39	2.47E-09
203	GPR109B	cg05384917	-0.25	6.54E-14	ILMN_1677693	-1.20	6.00E-03
204	IL10RA	cg26661481	-0.25	8.46E-14	ILMN_1652825	-1.44	1.92E-05
205	SFTPD	cg03600318	-0.15	1.01E-13	ILMN_1768575	-1.30	4.39E-13
206	TMEM71	cg27159719	-0.15	1.16E-13	ILMN_1674402	-1.47	1.00E-06
207	CCL4	cg25659818	-0.20	1.25E-13	ILMN_1674563	-1.55	5.26E-11
208	DPYS	cg20774846	-0.11	1.45E-13	ILMN_1749324	-1.68	4.84E-05
209	H19	cg26808784	-0.27	2.49E-13	ILMN_2148527	-2.75	5.24E-06
210	GJB2	cg11054936	-0.12	2.51E-13	ILMN_1769388	-1.64	2.20E-06
211	HK3	cg14709481	-0.17	3.30E-13	ILMN_1670302	-1.49	9.68E-13
212	MLN	cg20226764	-0.17	4.22E-13	ILMN_1685858	-1.27	3.48E-13
213	CLEC4G	cg16626670	-0.21	1.05E-12	ILMN_2193817	-7.64	7.90E-32
214	AZGP1	cg12019109	-0.16	2.06E-12	ILMN_1797154	-2.55	1.40E-14
215	SCGB3A1	cg14472601	-0.23	2.08E-12	ILMN_1679666	-1.47	1.60E-04
216	C1QA	cg00108454	-0.10	6.48E-12	ILMN_1737918	-1.34	9.80E-04
217	AFAR3	cg11376198	-0.20	1.30E-11	ILMN_1702696	-1.42	1.32E-06
218	DBH	cg25020204	-0.18	1.38E-11	ILMN_1746220	-4.74	2.05E-23
219	CCDC48	cg24287460	-0.17	1.43E-11	ILMN_1751099	-1.21	7.10E-07
220	OSBPL5	cg25852715	-0.17	1.72E-11	ILMN_1802151	-1.24	1.17E-04
221	CYP1A2	cg09207718	-0.19	2.38E-11	ILMN_1683607	-13.70	7.93E-22
222	CRISPLD2	cg07207789	-0.17	3.69E-11	ILMN_1790689	-1.70	1.08E-06
223	IL10	cg17067005	-0.20	4.05E-11	ILMN_2073307	-1.46	1.28E-07
224	CNGA1	cg19000186	-0.17	4.57E-11	ILMN_1786353	-1.91	3.52E-10
225	DIRAS3	cg21808053	-0.21	5.09E-11	ILMN_2174215	-1.29	3.49E-09
226	NDN	cg01989224	-0.16	6.60E-11	ILMN_1692058	-1.30	5.05E-03
227	GPLD1	cg14023451	-0.16	6.66E-11	ILMN_1765419	-1.31	1.53E-02
228	SLC18A1	cg13857100	-0.13	1.02E-10	ILMN_2172890	-1.52	2.40E-11
229	PTGDS	cg00563932	-0.17	1.65E-10	ILMN_1664464	-2.04	5.35E-08
230	AGXT	cg16967583	-0.10	1.85E-10	ILMN_1709796	-2.35	1.67E-09
231	IGLL1	cg10494770	-0.12	1.87E-10	ILMN_2393765	-4.05	2.84E-10
232	GNMT	cg24751129	-0.20	2.42E-10	ILMN_1736238	-4.02	2.88E-13
233	TIMD4	cg18994063	-0.13	2.96E-10	ILMN_1750678	-2.24	3.23E-11
234	GABRP	cg23181170	-0.13	4.88E-10	ILMN_1689146	-1.29	6.72E-08
235	IGF2	cg02807948	-0.12	5.82E-10	ILMN_1699867	-2.46	3.37E-07
236	PROM1	cg20576510	-0.18	6.50E-10	ILMN_1786720	-1.52	1.68E-08
237	DES	cg26259363	-0.14	6.63E-10	ILMN_1698995	-1.21	2.30E-05

238	GDF2	cg21115977	-0.18	8.85E-10	ILMN_1760363	-1.48	2.56E-15
239	LILRB2	cg05248470	-0.11	1.13E-09	ILMN_1734234	-1.31	8.56E-10
240	ALPP	cg13605579	-0.15	1.19E-09	ILMN_1693789	-1.40	3.84E-06
241	RAMP3	cg02417264	-0.12	1.29E-09	ILMN_2065745	-1.78	3.08E-14
242	KCNJ15	cg03993463	-0.13	1.38E-09	ILMN_1675756	-1.21	1.80E-05
243	APOC3	cg04048249	-0.12	1.58E-09	ILMN_1722070	-1.71	6.01E-06
244	APOB	cg24309555	-0.11	1.98E-09	ILMN_1664024	-1.39	2.26E-05
245	PMP22	cg03242666	-0.13	2.43E-09	ILMN_1659312	-1.22	1.24E-04
246	PLCG2	cg21522797	-0.13	2.62E-09	ILMN_1815719	-1.34	1.27E-03
247	SDC4	cg10876928	-0.14	3.36E-09	ILMN_1663042	-1.72	1.51E-11
248	BDH2	cg02214188	-0.11	3.45E-09	ILMN_1673360	-1.59	2.23E-15
249	ARHGDIB	cg10925082	-0.14	5.50E-09	ILMN_1678143	-1.36	4.94E-04
250	FGL1	cg01871995	-0.12	5.68E-09	ILMN_2366192	-1.61	3.21E-03
251	CTRC	cg23064554	-0.11	5.96E-09	ILMN_1748730	-1.21	2.27E-05
252	AHSG	cg07361385	-0.17	6.81E-09	ILMN_1730625	-1.40	5.89E-04
253	CYP8B1	cg14404298	-0.11	7.00E-09	ILMN_1669802	-2.71	2.01E-06
254	PPBP	cg20357806	-0.14	7.76E-09	ILMN_1767281	-1.21	2.43E-07
255	LAMC3	cg26491484	-0.17	8.04E-09	ILMN_1688642	-1.52	1.83E-07
256	CD48	cg13311440	-0.16	8.26E-09	ILMN_2061043	-1.21	3.78E-02
257	RARRES2	cg02902770	-0.12	1.14E-08	ILMN_1810844	-1.33	2.17E-03
258	MS4A6A	cg04353769	-0.13	1.21E-08	ILMN_1797731	-2.29	1.24E-13
259	CYP3A43	cg13364756	-0.15	1.78E-08	ILMN_1673591	-3.11	8.47E-18
260	VTN	cg04706338	-0.13	1.97E-08	ILMN_1691127	-1.38	6.35E-06
261	GZMK	cg04705866	-0.11	2.13E-08	ILMN_1710734	-1.57	6.93E-07
262	CLDN10	cg08418978	-0.16	2.64E-08	ILMN_1737096	-1.43	1.45E-12
263	GPT	cg15983520	-0.12	2.95E-08	ILMN_1795257	-2.36	1.61E-13
264	GMFG	cg02436686	-0.13	2.99E-08	ILMN_1711617	-1.51	1.07E-07
265	ZDHHC11	cg18429742	-0.15	3.85E-08	ILMN_1694514	-1.57	3.92E-06
266	ATAD3C	cg27383362	-0.13	3.98E-08	ILMN_2231569	-1.21	2.11E-07
267	PDK4	cg22171829	-0.11	4.15E-08	ILMN_1684982	-2.25	3.86E-10
268	SERPINA10	cg05788638	-0.16	4.58E-08	ILMN_1793628	-1.85	7.87E-09
269	GUCY1A3	cg02210887	-0.11	4.87E-08	ILMN_2131177	-1.40	2.37E-11
270	SARDH	cg21122774	-0.14	5.70E-08	ILMN_1704973	-1.90	7.62E-16
271	EFS	cg07197059	-0.18	6.14E-08	ILMN_1765248	-1.23	7.75E-08
272	SLC27A5	cg23036025	-0.10	8.69E-08	ILMN_1725366	-2.87	8.09E-10
273	NR4A1	cg03533058	-0.13	8.99E-08	ILMN_1661178	-1.65	7.53E-15
274	ITIH1	cg06134964	-0.15	9.22E-08	ILMN_1755251	-1.78	1.00E-08
275	HMGCS2	cg10212621	-0.11	9.27E-08	ILMN_1815203	-1.71	2.36E-07
276	TNXB	cg13823701	-0.11	1.07E-07	ILMN_1684614	-1.62	2.73E-16
277	NTF3	cg02554564	-0.11	1.11E-07	ILMN_1809364	-2.00	2.17E-19
278	KCNAB1	cg15423862	-0.16	1.13E-07	ILMN_1744968	-1.26	3.59E-07
279	NKG7	cg10126923	-0.17	1.30E-07	ILMN_1682993	-1.38	9.15E-06
280	CA5A	cg12343082	-0.14	1.65E-07	ILMN_1731292	-2.03	1.45E-09
281	SLC2A2	cg17142134	-0.15	1.93E-07	ILMN_1755720	-1.90	9.03E-06
282	F2	cg20199333	-0.14	2.33E-07	ILMN_3249291	-1.65	1.82E-08
283	KCNK17	cg02611419	-0.17	2.78E-07	ILMN_1717702	-1.43	1.65E-11
284	LY86	cg02212836	-0.17	3.02E-07	ILMN_1807825	-1.27	8.17E-04
285	MBL2	cg27418851	-0.12	3.40E-07	ILMN_1762464	-3.52	4.14E-12
286	SLCO4A1	cg09210315	-0.10	3.49E-07	ILMN_1727200	-1.29	3.18E-05
287	RBP5	cg24441911	-0.16	3.55E-07	ILMN_2103024	-1.85	1.36E-07
288	F12	cg12332316	-0.14	3.56E-07	ILMN_1671766	-1.82	1.95E-06
289	CFB	cg17741572	-0.14	3.62E-07	ILMN_1774287	-1.35	4.94E-04
290	PLA2G12B	cg21820890	-0.16	3.72E-07	ILMN_1808487	-1.20	8.06E-03
291	AQP12A	cg06356454	-0.14	4.47E-07	ILMN_1757036	-1.52	1.47E-12
292	A1BG	cg22568540	-0.16	4.83E-07	ILMN_2055271	-1.21	1.14E-03
293	ORM2	cg16408565	-0.18	5.71E-07	ILMN_1731785	-1.63	2.76E-06
294	CD86	cg04387658	-0.12	6.60E-07	ILMN_1714602	-1.26	1.46E-03
295	SULT2A1	cg19139729	-0.10	6.71E-07	ILMN_1692983	-2.15	1.12E-06
296	FCGR3A	cg22202141	-0.12	9.77E-07	ILMN_1703679	-1.40	2.90E-10
297	CD79B	cg25729716	-0.10	9.95E-07	ILMN_2366212	-1.28	2.63E-05
298	COLEC10	cg05755779	-0.16	1.06E-06	ILMN_1662962	-1.85	2.41E-17

299	HSD17B2	cg20373326	-0.10	1.29E-06	ILMN_1808713	-2.78	2.34E-12
300	FCGR3B	cg04567009	-0.12	1.59E-06	ILMN_1728639	-1.32	5.75E-05
301	FGFR1	cg08722122	-0.15	1.65E-06	ILMN_1729369	-1.39	1.04E-11
302	GIMAP7	cg01827098	-0.15	1.73E-06	ILMN_1776678	-1.38	1.17E-05
303	ACY1	cg16159313	-0.17	2.47E-06	ILMN_1683883	-1.25	1.14E-02
304	FMO3	cg25778166	-0.12	2.74E-06	ILMN_2344283	-2.10	1.07E-05
305	KIAA0367	cg11880010	-0.12	2.74E-06	ILMN_1810628	-1.23	1.55E-04
306	F7	cg24269657	-0.13	3.13E-06	ILMN_1740559	-1.65	2.00E-09
307	S100A9	cg07039113	-0.16	3.68E-06	ILMN_1750974	-1.60	1.46E-03
308	ABCG5	cg08453096	-0.13	4.06E-06	ILMN_1716592	-1.29	1.13E-03
309	EPB41L1	cg20993403	-0.13	4.12E-06	ILMN_1716507	-1.25	2.49E-05
310	ACP5	cg07967308	-0.10	4.52E-06	ILMN_2078599	-1.36	4.37E-03
311	LIPC	cg01733599	-0.11	5.03E-06	ILMN_1812700	-2.21	4.67E-11
312	LRP3	cg08700306	-0.11	5.64E-06	ILMN_2127605	-1.45	2.55E-07
313	NPDC1	cg26581729	-0.15	6.80E-06	ILMN_2199313	-1.24	8.66E-09
314	PROC	cg26718585	-0.12	6.90E-06	ILMN_1687721	-1.69	2.03E-10
315	CCL16	cg05766474	-0.15	8.31E-06	ILMN_2045324	-1.29	1.94E-02
316	FXYD2	cg25894551	-0.14	8.86E-06	ILMN_1781045	-1.51	3.40E-09
317	PROZ	cg25734864	-0.13	9.45E-06	ILMN_1664565	-2.92	1.23E-13
318	SERPIND1	cg02523400	-0.12	1.04E-05	ILMN_1707975	-1.21	1.10E-02
319	NR0B2	cg10055471	-0.13	1.08E-05	ILMN_2220978	-2.02	5.76E-10
320	ANPEP	cg13042288	-0.11	1.32E-05	ILMN_1763837	-1.68	1.99E-08
321	EDN2	cg27238470	-0.11	1.35E-05	ILMN_1680814	-1.22	3.01E-05
322	PCSK6	cg20249919	-0.12	1.52E-05	ILMN_1768577	-1.45	7.19E-05
323	LDB2	cg03368758	-0.11	1.93E-05	ILMN_1800697	-1.43	1.09E-07
324	CYP2E1	cg00436603	-0.10	2.51E-05	ILMN_1665437	-5.04	5.97E-11
325	LCP2	cg17127769	-0.11	2.59E-05	ILMN_1658962	-1.33	3.05E-08
326	CSF1R	cg04569804	-0.10	2.81E-05	ILMN_1686623	-1.67	5.41E-07
327	ITIH3	cg26099316	-0.10	3.41E-05	ILMN_1757461	-1.22	3.98E-02
328	PCOLCE	cg26777475	-0.10	3.96E-05	ILMN_1707070	-1.81	1.12E-05
329	UPB1	cg10613381	-0.11	4.07E-05	ILMN_1678690	-1.65	1.60E-05
330	ISLR	cg02077702	-0.11	4.22E-05	ILMN_1747593	-1.45	3.20E-05
331	AMBP	cg06630567	-0.11	5.00E-05	ILMN_1695446	-1.47	1.43E-05
332	NGFR	cg17129388	-0.12	5.28E-05	ILMN_1752658	-1.51	2.20E-17
333	ITGAD	cg02164442	-0.12	7.86E-05	ILMN_1681945	-1.60	1.09E-08
334	SERPINA6	cg10025865	-0.10	8.25E-05	ILMN_2215418	-1.36	4.56E-03
335	TFR2	cg10681065	-0.11	1.19E-04	ILMN_1724738	-2.01	9.16E-12
336	PER3	cg25514503	-0.10	1.32E-04	ILMN_1660986	-1.29	1.07E-05
337	EVC2	cg03278643	-0.13	1.53E-04	ILMN_1762735	-2.11	1.30E-14
338	ANKRD38	cg00625425	-0.12	1.85E-04	ILMN_1776936	-1.52	4.68E-04
339	SLC39A14	cg05254747	-0.10	3.06E-04	ILMN_1764629	-1.66	8.58E-10
340	TYROBP	cg20267005	-0.12	3.24E-04	ILMN_1778977	-1.31	8.93E-03
341	CXCL12	cg18618334	-0.14	6.60E-04	ILMN_1791447	-5.25	9.64E-21
342	COL8A2	cg18931815	-0.12	6.87E-04	ILMN_2102330	-1.32	6.24E-07
343	ENOSF1	cg16112050	-0.10	3.02E-03	ILMN_1803676	-1.21	9.08E-04
344	SF3B14	cg04809136	0.53	1.49E-33	ILMN_1703720	1.39	5.22E-11
345	HIST1H2AE	cg20520725	0.35	1.27E-23	ILMN_1756849	1.26	3.19E-05
346	NETO2	cg02755525	0.52	1.17E-22	ILMN_1760849	1.46	1.89E-10
347	GPR160	cg18741908	0.28	1.79E-20	ILMN_1662846	1.24	3.33E-04
348	RASL12	cg10604168	0.33	5.55E-20	ILMN_1806403	1.33	2.13E-09
349	ATP1A1	cg09580336	0.25	3.76E-19	ILMN_1731783	1.44	3.13E-06
350	CELSR3	cg06621358	0.31	3.94E-19	ILMN_1691290	1.95	1.25E-13
351	COL7A1	cg11846236	0.35	1.29E-18	ILMN_1751161	1.49	6.84E-06
352	RAB22A	cg26029902	0.30	1.96E-18	ILMN_1786976	1.35	1.02E-07
353	EFNB2	cg24929737	0.39	7.32E-18	ILMN_1703852	1.34	1.18E-07
354	MSX1	cg03843978	0.19	1.18E-17	ILMN_1777397	1.44	3.27E-10
355	CDKN2B	cg06421800	0.18	3.59E-17	ILMN_2376723	1.31	3.88E-06
356	FOXD2	cg15868302	0.14	8.55E-17	ILMN_1789400	1.51	3.47E-10
357	LGALS3	cg19099850	0.45	2.17E-15	ILMN_1803788	1.37	1.10E-03
358	AKR1B1	cg13801416	0.40	3.56E-15	ILMN_1701731	1.57	1.02E-03
359	MSC	cg23710218	0.25	1.50E-14	ILMN_1741404	1.29	3.19E-03

360	HOXA13	cg06397837	0.28	3.80E-14	ILMN_1731349	1.72	9.82E-08
361	CLEC14A	cg14473145	0.21	6.82E-14	ILMN_2142185	1.28	1.17E-03
362	CTHRC1	cg26952662	0.36	3.71E-13	ILMN_1725090	1.84	6.61E-06
363	HIST3H2A	cg14743462	0.28	8.69E-13	ILMN_1779648	1.20	2.21E-04
364	APH1B	cg17207590	0.22	4.13E-12	ILMN_1767816	1.36	2.12E-05
365	RPL39L	cg07441272	0.30	7.36E-12	ILMN_1712413	1.30	1.50E-02
366	CBR3	cg14564494	0.19	8.83E-12	ILMN_1652237	1.27	3.22E-05
367	TMEM119	cg16708012	0.12	9.39E-12	ILMN_1738116	1.22	9.99E-03
368	ACTG1	cg19517525	0.35	2.93E-11	ILMN_2053178	1.23	6.08E-05
369	PDGFRB	cg12727795	0.19	3.13E-11	ILMN_1815057	1.31	1.80E-02
370	RAB5C	cg22373112	0.18	3.94E-11	ILMN_1769665	1.30	1.14E-05
371	NINJ2	cg00237010	0.16	4.10E-11	ILMN_1731745	1.36	3.48E-03
372	TERT	cg02545192	0.27	4.26E-11	ILMN_2373119	1.39	1.09E-05
373	EIF5A2	cg10541755	0.30	5.21E-11	ILMN_1735151	1.34	1.59E-05
374	EMILIN2	cg09009111	0.29	6.94E-11	ILMN_1697268	1.25	1.92E-02
375	PITX1	cg24495017	0.25	7.55E-11	ILMN_1674386	1.68	2.01E-06
376	SPARC	cg08331313	0.20	8.34E-11	ILMN_1796734	1.20	4.15E-02
377	STEAP1	cg18320336	0.16	1.97E-10	ILMN_1733094	1.37	2.74E-03
378	ZNF606	cg12259537	0.19	2.05E-10	ILMN_1681829	1.25	9.11E-08
379	ZNF514	cg18793806	0.17	2.82E-10	ILMN_2215211	1.25	2.79E-08
380	RAB11FIP4	cg10149836	0.21	3.85E-10	ILMN_1681467	1.28	1.06E-09
381	COG2	cg17031727	0.19	4.14E-10	ILMN_1776993	1.30	1.18E-08
382	PFKP	cg08465862	0.24	5.06E-10	ILMN_1805737	1.23	6.31E-03
383	ACSS1	cg15988792	0.23	7.10E-10	ILMN_1752269	1.25	1.58E-05
384	TPM2	cg17691309	0.21	9.79E-10	ILMN_1789196	1.43	7.37E-04
385	UCHL1	cg08319991	0.18	1.12E-09	ILMN_1757387	1.28	2.89E-02
386	BAK1	cg07679836	0.19	4.07E-09	ILMN_1805990	1.24	1.16E-08
387	DHDDS	cg06577005	0.17	4.81E-09	ILMN_2405642	1.24	3.32E-08
388	PARP12	cg07937272	0.14	5.41E-09	ILMN_1718558	1.56	2.77E-12
389	TBL2	cg22511633	0.20	5.80E-09	ILMN_1764489	1.42	1.52E-13
390	HLA-G	cg21529533	0.21	8.18E-09	ILMN_1656670	1.59	1.36E-07
391	IGF2BP1	cg06638433	0.22	1.06E-08	ILMN_1733807	1.31	4.17E-05
392	FLNC	cg02661879	0.25	1.11E-08	ILMN_1715748	1.39	1.02E-03
393	BCAR3	cg18060199	0.25	1.63E-08	ILMN_1763638	1.30	1.43E-05
394	PAPSS1	cg09191232	0.15	2.17E-08	ILMN_1781819	1.35	2.12E-08
395	GPX7	cg22129364	0.22	2.71E-08	ILMN_1726030	1.40	6.13E-07
396	SCGN	cg16954341	0.17	2.88E-08	ILMN_1789648	1.98	5.65E-05
397	CD34	cg19591881	0.19	3.70E-08	ILMN_2341229	2.22	3.02E-19
398	PNMA1	cg23681213	0.21	4.71E-08	ILMN_1770850	1.28	5.81E-05
399	HES6	cg24127874	0.20	6.85E-08	ILMN_1694268	1.65	9.71E-13
400	ZNF232	cg24680602	0.21	7.31E-08	ILMN_2139351	1.31	3.02E-10
401	STXBP6	cg06948294	0.14	1.21E-07	ILMN_1750912	1.78	1.97E-14
402	ACPL2	cg00400028	0.18	1.26E-07	ILMN_1706598	1.26	3.01E-05
403	PPP1R2	cg19280968	0.23	1.40E-07	ILMN_1683044	1.22	9.06E-04
404	DDAH2	cg18055007	0.17	1.48E-07	ILMN_1770787	1.31	4.31E-04
405	THBS4	cg18110483	0.16	2.23E-07	ILMN_1736078	2.25	1.33E-08
406	PRR3	cg21264055	0.19	2.39E-07	ILMN_1750167	1.21	2.30E-09
407	MSTO1	cg15696781	0.17	2.58E-07	ILMN_1707337	1.22	7.68E-08
408	DLK1	cg17412258	0.18	3.44E-07	ILMN_1652975	1.35	4.13E-02
409	THY1	cg12508624	0.18	4.15E-07	ILMN_1779875	2.24	3.81E-15
410	BTG3	cg14380517	0.18	1.19E-06	ILMN_1707339	1.39	3.49E-05
411	HIST1H2AC	cg17711541	0.11	1.39E-06	ILMN_1792689	1.38	5.39E-04
412	MYO5A	cg23287547	0.14	2.11E-06	ILMN_1698225	1.30	2.07E-05
413	EEF1A2	cg22463915	0.15	2.52E-06	ILMN_2108735	2.19	1.28E-05
414	DNAJB6	cg11695266	0.16	3.09E-06	ILMN_1793770	1.49	3.34E-09
415	NFE2L3	cg07986525	0.20	3.13E-06	ILMN_2049766	1.21	5.58E-07
416	CDCA7	cg08690031	0.18	4.30E-06	ILMN_1737184	1.46	3.49E-06
417	CTSL2	cg13234643	0.19	5.33E-06	ILMN_1748352	1.55	2.15E-06
418	HIST1H2BE	cg21146268	0.12	7.37E-06	ILMN_1687947	1.31	7.76E-07
419	TUBB2B	cg03462055	0.15	7.68E-06	ILMN_1680874	1.28	1.47E-04
420	IMPDH1	cg24127989	0.17	7.77E-06	ILMN_1676515	1.22	2.59E-03

421	RALBP1	cg15982419	0.15	9.46E-06	ILMN_1791840	1.30	1.96E-08
422	CCNF	cg06361108	0.16	1.03E-05	ILMN_1773119	1.37	8.54E-14
423	TTC4	cg02255609	0.16	1.40E-05	ILMN_1678140	1.23	1.44E-05
424	HLA-A	cg20879959	0.13	1.82E-05	ILMN_1671054	1.23	7.65E-03
425	COMP	cg09949775	0.18	2.67E-05	ILMN_1677636	1.37	3.15E-04
426	UBE2L3	cg25421647	0.14	3.20E-05	ILMN_1677877	1.27	1.19E-05
427	ARID3A	cg18084554	0.11	3.56E-05	ILMN_1670130	1.22	2.34E-02
428	C1QL1	cg13818573	0.17	3.61E-05	ILMN_1716957	1.20	1.00E-03
429	NES	cg21838334	0.14	4.12E-05	ILMN_1738147	1.28	3.78E-07
430	LOX	cg02548238	0.17	4.42E-05	ILMN_1695880	1.31	1.30E-04
431	CHAF1B	cg13854874	0.15	5.40E-05	ILMN_1674231	1.47	3.53E-08
432	MALL	cg01615704	0.15	6.24E-05	ILMN_2063168	1.31	2.60E-06
433	BCAT1	cg10764357	0.12	1.03E-04	ILMN_1766169	1.60	1.11E-06
434	DYNC1I1	cg10281478	0.11	1.14E-04	ILMN_1690397	1.25	3.17E-03
435	TSPAN5	cg00622677	0.11	1.27E-04	ILMN_1799028	1.30	1.79E-03
436	PRKCDBP	cg18392783	0.11	1.27E-04	ILMN_1793476	1.23	5.34E-06
437	CNNM1	cg01968793	0.13	1.46E-04	ILMN_1657750	1.25	7.08E-05
438	BLVRA	cg17571291	0.11	1.53E-04	ILMN_1691436	1.48	1.12E-04
439	TUBB3	cg15669092	0.13	1.54E-04	ILMN_1791726	1.20	5.58E-04
440	MAPK13	cg24155668	0.13	1.63E-04	ILMN_1749327	1.32	3.05E-03
441	NOTCH3	cg17498321	0.13	1.85E-04	ILMN_1658926	1.40	1.98E-11
442	IGF2BP2	cg18234011	0.14	3.31E-04	ILMN_1702447	1.61	1.61E-05
443	GPD1L	cg05662500	0.14	3.32E-04	ILMN_1694106	1.32	6.09E-04
444	AP3M2	cg07490776	0.13	3.53E-04	ILMN_1676946	1.46	1.87E-14
445	CGREF1	cg22740783	0.16	3.83E-04	ILMN_1774722	1.51	1.26E-10
446	PPM1M	cg20276750	0.15	3.87E-04	ILMN_1657810	1.20	1.26E-03
447	HOXD1	cg19001226	0.12	3.99E-04	ILMN_1717381	1.36	3.35E-05
448	ACOT8	cg08101264	0.13	4.53E-04	ILMN_1679600	1.25	7.31E-08
449	FZD6	cg21975377	0.11	9.75E-04	ILMN_1659297	1.21	6.02E-04
450	RBM4	cg14916213	0.12	1.09E-03	ILMN_1712455	1.35	2.06E-13
451	ACTN2	cg21376883	0.10	1.28E-03	ILMN_1654422	1.26	2.31E-03
452	LPL	cg08918749	0.11	1.33E-03	ILMN_1786444	1.25	5.31E-04
453	GLS	cg15522719	0.10	1.42E-03	ILMN_2188722	1.26	3.13E-05
454	RAD54L	cg03262773	0.13	1.72E-03	ILMN_1658027	1.58	1.22E-13
455	MIF	cg20377673	0.12	2.00E-03	ILMN_1807074	1.35	6.16E-04
456	RHEB	cg03998173	0.11	2.47E-03	ILMN_1657949	1.67	1.92E-13
457	HOXA5	cg02248486	0.12	3.27E-03	ILMN_1753613	1.23	1.88E-03
458	PLK1	cg06240124	0.12	3.94E-03	ILMN_1736176	1.37	1.47E-12
459	SLC7A1	cg14424530	0.10	6.06E-03	ILMN_1683859	1.22	7.33E-03
460	CCL20	cg21643045	-0.31	2.68E-26	ILMN_1657234	3.42	2.78E-09
461	WDR21C	cg17703554	-0.26	1.10E-25	ILMN_1768454	1.42	7.93E-04
462	PI3	cg02442161	-0.22	4.31E-20	ILMN_1693192	1.26	9.94E-06
463	TOP1MT	cg12188860	-0.32	5.76E-20	ILMN_2405628	1.37	2.36E-05
464	REG1A	cg05828624	-0.22	6.21E-19	ILMN_1802441	1.29	2.48E-02
465	CST1	cg08430598	-0.30	4.43E-18	ILMN_1753449	1.21	3.14E-02
466	SLC22A18AS	cg08999895	-0.27	2.14E-17	ILMN_1691048	1.44	1.17E-05
467	DDX49	cg14757492	-0.23	4.05E-16	ILMN_1762225	1.38	4.26E-07
468	PDPK1	cg14444710	-0.22	4.84E-16	ILMN_1653793	1.42	1.58E-10
469	RBM15B	cg15821095	-0.24	6.10E-16	ILMN_1673024	1.32	7.86E-12
470	CDH13	cg02168291	-0.22	7.71E-15	ILMN_1766925	1.76	2.64E-15
471	PGCP	cg01892689	-0.18	3.05E-14	ILMN_2058795	1.60	1.68E-08
472	ESM1	cg07233761	-0.15	4.58E-14	ILMN_2212878	2.34	4.58E-14
473	ATP5J2	cg03665605	-0.17	7.43E-14	ILMN_2307883	1.45	2.19E-11
474	ZNF323	cg20850981	-0.17	1.02E-13	ILMN_2377991	1.44	5.02E-07
475	RNASE1	cg13718960	-0.17	4.88E-13	ILMN_1795183	1.23	1.07E-02
476	ARHGEF18	cg27377450	-0.12	5.41E-13	ILMN_1664016	1.20	1.33E-03
477	HSDL1	cg26359240	-0.14	6.84E-13	ILMN_2112755	1.25	1.38E-07
478	OLFML2A	cg18506679	-0.16	1.31E-12	ILMN_1761425	1.33	1.24E-05
479	AKR1B10	cg11693019	-0.17	2.08E-12	ILMN_1672148	7.72	4.94E-11
480	PDZK1	cg10321723	-0.18	6.58E-12	ILMN_1708341	1.70	4.59E-08
481	COX4I2	cg09027725	-0.15	9.32E-12	ILMN_1815634	1.25	2.00E-12

482	REG3A	cg27342801	-0.21	1.54E-11	ILMN_1757504	1.24	1.82E-02
483	ZNF7	cg20845050	-0.12	1.60E-11	ILMN_2137066	1.57	5.57E-13
484	JMJD4	cg11520395	-0.15	1.67E-11	ILMN_1692896	1.25	4.93E-09
485	SERPINH1	cg27596068	-0.12	3.50E-11	ILMN_1751028	1.28	3.21E-05
486	ACTG2	cg23173910	-0.16	3.85E-11	ILMN_1795325	2.32	5.15E-08
487	BTBD3	cg14603345	-0.20	4.21E-11	ILMN_1713964	1.23	4.26E-05
488	VWF	cg27347104	-0.15	4.44E-11	ILMN_1752755	2.37	1.46E-08
489	SLC26A3	cg22294577	-0.16	5.33E-11	ILMN_1760087	1.35	7.71E-03
490	DAPK2	cg23165541	-0.18	8.24E-11	ILMN_1791847	1.30	2.88E-08
491	RPS2	cg18279742	-0.14	1.12E-10	ILMN_2218277	1.44	2.39E-07
492	NDUFA8	cg01536400	-0.14	1.13E-10	ILMN_1759729	1.20	4.57E-05
493	UBD	cg15320474	-0.17	5.78E-10	ILMN_1678841	5.14	1.92E-13
494	MTMR11	cg24620905	-0.11	6.69E-10	ILMN_1769299	2.03	3.10E-10
495	PAQR9	cg26301689	-0.19	1.90E-09	ILMN_1798327	1.62	2.61E-08
496	FBLN1	cg04337944	-0.17	2.92E-09	ILMN_1700541	1.32	6.90E-03
497	PRND	cg09906458	-0.11	4.01E-09	ILMN_1684795	1.32	1.29E-05
498	SPINK1	cg04577715	-0.20	4.66E-09	ILMN_1787266	11.76	3.05E-11
499	TNFRSF4	cg22335801	-0.20	1.19E-08	ILMN_2112256	1.31	5.26E-11
500	PTGFRN	cg03752628	-0.12	1.21E-08	ILMN_1743130	1.32	8.30E-08
501	GPNMB	cg17274742	-0.14	1.26E-08	ILMN_1801205	1.57	2.40E-03
502	FGF21	cg17264470	-0.18	3.15E-08	ILMN_1772789	1.28	3.10E-02
503	CLDN15	cg02512860	-0.21	3.73E-08	ILMN_1682226	1.93	3.17E-10
504	AHCTF1	cg27050763	-0.21	4.27E-08	ILMN_1809139	1.20	2.20E-04
505	GPX1	cg06613840	-0.12	7.34E-08	ILMN_1749662	1.31	3.27E-03
506	ISG20L2	cg00392257	-0.16	1.37E-07	ILMN_2090397	1.21	2.16E-05
507	PLOD3	cg25527547	-0.13	1.91E-07	ILMN_1774836	1.86	3.25E-17
508	CEACAM1	cg20657383	-0.12	2.64E-07	ILMN_1664330	1.38	4.53E-05
509	MYH4	cg23400451	-0.11	2.79E-07	ILMN_1729251	1.21	2.25E-02
510	ATP6V1E2	cg27485921	-0.11	2.89E-07	ILMN_1810235	1.41	4.97E-11
511	PGC	cg17391877	-0.12	3.98E-07	ILMN_1795484	1.57	7.95E-03
512	DEGS1	cg23213217	-0.17	4.29E-07	ILMN_1780058	1.27	1.07E-03
513	TPM3	cg24490338	-0.13	4.48E-07	ILMN_1697567	1.61	2.77E-14
514	SLC2A5	cg24480859	-0.13	4.73E-07	ILMN_1671337	2.07	6.56E-08
515	NCSTN	cg00689010	-0.13	5.87E-07	ILMN_1735180	1.48	1.78E-08
516	TMC5	cg25690265	-0.12	1.07E-06	ILMN_1730117	1.23	1.76E-02
517	COL15A1	cg20503329	-0.14	1.25E-06	ILMN_1768940	2.22	9.12E-15
518	SFN	cg03421300	-0.11	1.54E-06	ILMN_1806607	1.30	5.99E-05
519	ZNF572	cg23968383	-0.18	1.55E-06	ILMN_1802974	1.27	2.22E-08
520	RAB4A	cg06646021	-0.12	2.05E-06	ILMN_1677824	1.34	3.77E-07
521	GCNT3	cg23877385	-0.12	3.82E-06	ILMN_1712082	1.69	2.34E-05
522	RASGRP3	cg01109219	-0.11	3.98E-06	ILMN_1727045	1.25	9.53E-05
523	AQP10	cg20713492	-0.12	4.00E-06	ILMN_2090004	1.25	5.74E-03
524	SRI	cg02399455	-0.12	4.82E-06	ILMN_1682054	1.22	9.48E-07
525	SLC22A11	cg09326702	-0.12	9.52E-06	ILMN_1735386	1.55	6.51E-05
526	CYP17A1	cg09201719	-0.11	1.45E-05	ILMN_1760554	1.49	3.04E-04
527	AQP8	cg07327347	-0.12	2.69E-05	ILMN_1713462	1.22	4.57E-03
528	CARD10	cg13797282	-0.13	3.09E-05	ILMN_1743714	1.21	1.74E-03
529	NUP50	cg07986773	-0.15	3.22E-05	ILMN_1725612	1.22	7.72E-05
530	LGALS4	cg06394229	-0.12	6.40E-05	ILMN_1694034	1.69	6.92E-03
531	MMP9	cg20925811	-0.11	7.53E-05	ILMN_1796316	2.04	1.01E-04
532	S100A10	cg10795646	-0.10	1.04E-04	ILMN_2046730	2.07	1.88E-13
533	CMTM8	cg01617750	-0.11	2.66E-04	ILMN_1710124	1.22	4.94E-03
534	PLCE1	cg09480837	-0.13	2.90E-04	ILMN_1784447	1.38	5.05E-06
535	CAD	cg09947274	-0.10	3.06E-04	ILMN_1810992	1.28	3.19E-10
536	VHL	cg16869108	-0.13	9.87E-04	ILMN_1801984	1.30	4.35E-10
