# Supplementary material for: Methylation Profiles Reveal Distinct Subgroup of Hepatocellular Carcinoma Patients with Poor Prognosis
Source: PLoS One. 2014 Aug 5;9(8):e104158. doi: 10.1371/journal.pone.0104158 (PMC4122406; doi:10.1371/journal.pone.0104158)
Supplement: Table S8 — IPA results for top biological functions enriched in 536 genes with differential methylation and associated expression change. (PDF) [file pone.0104158.s013.pdf]

Table S8. IPA results for top biological functions enriched in 536 genes with differential methylation and associated expression change.

Category	B-H adjusted p-Value	No. of Molecules	Molecules
Cellular Movement	5.17E-12	97	ALPP,ANPEP,ARHGDIB,BCAR3,BMP6,CARD10,CCL16,CCL20,CCL4,CCND1,CD48,CD86,CDH13,CEACAM1,CMTM8,COL7A1,CSF1R,CSF3R,CTBP2,CTSG,CXCL12,CXCL6,DEFA1 (includes others),DNAJB6,DOK1,EDN2,EFNB2,EFNB3,ENG,EPHA2,ESR1,F2,F7,FCGR3A,FGFR1,FLNC,FYN,GDF2,GPLD1,HAVCR2,HES6,HLA-G,HOXA4,IGF2,IGF2BP1,IL10,ITGA9,KLF2,KLF4,LEP,LGALS3,LIFR,LOX,LTK,MIF,MMP9,MYO10,NES,NR4A1,PCSK6,PDGFRB,PMP22,PODN,PPBP,PROC,PROK2,PTGS2,RAC2,RALBP1,RARRES2,RECK,S100A10,S100A12,S100A8,S100A9,SCGB3A1,SDC4,SERPIND1,SFN,SFTPD,SNAI1,SOCS3,SPARC,ST6GAL1,TFF2,THBS4,THY1,TNFRSF1B,TNK2,TPM3,TUBB2B,VHL,VTN,VWF,WASF3,WNT11,ZBTB16
Cellular Growth and Proliferation	3.35E-09	155	ACTG1,AHSG,AKAP12,AKR1B10,ALOX5,ANPEP,APCDD1,BAK1,BCAR3,BCAT1,BMP6,BMPER,BTG3,CARD10,CASP1,CBFA2T3,CBS,CCL20,CCL4,CCND1,CD160,CD86,CD8A,CDA,CDCA7,CDH13,CDKN1C,CDKN2B,CEACAM1,CEACAM6,CGREF1,COL6A2,CSF1R,CSF3R,CTBP2,CXCL12,DAPK2,DEFA1 (includes others),DEGS1,DES,DIRAS3,DLK1,DUSP2,EDN2,EFEMP1,EFNB2,EGR2,EGR3,EIF5A2,EMILIN2,ENG,EPHA2,ESM1,ESR1,F12,F2,F7,FBLN1,FES,FGFR1,FYN,GABRP,GDF2,GJB2,GNMT,GPLD1,GPNMB,GPX1,GSTP1,HAVCR2,HES6,HEXIM1,HGFAC,HOXA5,IGF2,IGF2BP1,IL10,IL10RA,KLF2,KLF4,LAMC3,LEP,LEPREL1,LGALS3,LGALS4,LIFR,LILRB2,LOX,LTK,LY86,MIF,MMP9,MNDA,MSX1,NCSTN,NDN,NES,NGFR,NOTCH3,NR1H3,NR4A1,OXT,PDGFRB,PDK4,PDPK1,PDZK1,PFKP,PLAT,PLCE1,PLCG2,PLK1,PMP22,PODN,PRG2,PRKAR2B,PROC,PROK2,PTGS2,RAC2,RASSF5,RECK,REG1A,REG3A,RRAD,S100A10,SCGB3A1,SCGN,SEMA6A,SERPIND1,SERPINH1,SFN,SFRP5,SFTPD,SLC22A1,SLC26A3,SNAI1,SOCS3,SPARC,SPINT2,SULT2A1,TACSTD2,TERT,TFPI2,TGFBR3,THBS4,TNFRSF1B,TNFRSF4,TNK2,TSLP,TUBB3,UBE2L3,UCHL1,VHL,VTN,ZBTB16
Cell-To-Cell Signaling and Interaction	7.80E-07	35	CCL20,CCL4,CD34,CD86,CEACAM1,CLEC4G,CTSG,CXCL12,DEFA1 (includes others),ENG,ESM1,F12,F2,FCGR3B,HLA-A,IGF2,IL10,ITGA9,ITGAD,LGALS3,LGALS3BP,LIPC,LPL,MARCO,PDGFRB,PROC,RAMP3,S100A10,S100A9,SDC4,SNAI1,SPARC,ST6GAL1,VTN,VWF
Cellular Development	1.34E-05	30	ANPEP,CCL20,CCL4,CD160,CD86,CD8A,CDKN1C,CEACAM1,CSF1R,CSF3R,CXCL12,HAVCR2,HOXA5,IGF2,IL10,IL10RA,KLF4,LEP,LGALS4,LILRB2,MIF,MNDA,RAC2,SFTPD,SOCS3,TERT,TNFRSF1B,TNFRSF4,TSLP,ZBTB16
Molecular Transport	2.48E-05	20	CCL16,CCL20,CCL4,CD8A,CXCL12,DEFA1 (includes others),F2,F7,FCGR3A,FCGR3B,FXYD2,FYN,KCNQ1,LCP2,PLCG2,PPBP,RGS2,SCGN,TRPV4,VWF
Cellular Function and Maintenance	2.48E-05	20	CCL16,CCL20,CCL4,CD8A,CXCL12,DEFA1 (includes others),F2,F7,FCGR3A,FCGR3B,FXYD2,FYN,KCNQ1,LCP2,PLCG2,PPBP,RGS2,SCGN,TRPV4,VWF
Cell Death and Survival	9.29E-05	119	AKAP12,AKR1B1,ALOX5,ANPEP,APOB,APOC3,BAK1,BLVRA,BMP6,CASP1,CBS,CCL4,CCND1,CD160,CD48,CD79B,CD8A,CDA,CDKN1C,CEACAM1,CEACAM6,CFH,COMP,CSF1R,CSF3R,CTBP2,CXCL12,CYP2E1,DAPK2,DDAH2,DEFA1 (includes others),DIRAS3,EFEMP1,EFNB2,EGR3,EMILIN2,ENG,EPHA2,ESR1,F2,F7,FBLN1,FCGR3A,FCGR3B,FGFR1,GABRP,GIMAP5,GPLD1,GPX1,GPX7,GSTP1,GZMA,GZMH,GZMK,HAVCR2,HLA-G,HOXA5,IGF2,IGF2BP1,IL10,KLF2,KLF4,LAG3,LEP,LGALS3,LGALS3BP,LGALS4,LTK,LY86,MIF,MMP9,MNDA,MSX1,NDN,NGFR,NME4,NR4A1,NTF3,PDGFRB,PDPK1,PDZK1,PI3,PLAT,PLK1,PMP22,PPBP,PRG2,PRKAR2B,PROC,PROK2,PTGDS,PTGS2,RAC2,RALBP1,RASGRP2,RASSF2,RASSF5,RNASE1,S100A8,S100A9,SCGB3A1,SEMA6A,SFN,SFRP5,SNAI1,SOCS2,SOCS3,SPARC,ST6GAL1,TERT,TGFBR3,TNFRSF1B,TUBB3,UBD,UCHL1,VHL,VTN,WNT11,ZBTB16
Vitamin and Mineral Metabolism	2.04E-04	17	CCL16,CCL20,CCL4,CD8A,CXCL12,DEFA1 (includes others),F2,F7,FCGR3A,FCGR3B,LCP2,PLCG2,PPBP,RGS2,SCGN,TRPV4,VWF
Cell Morphology	7.49E-04	24	BAK1,CCND1,CDKN1C,CMTM8,CXCL12,EFNB3,EPHA2,ESR1,F2,F7,FES,IL10,KLF2,KLF4,MARCO,MYH4,PMP22,PRKAR2B,SNAI1,TERT,TPM3,VHL,VTN,ZBTB16
Lipid Metabolism	7.51E-04	17	ACSS1,ALOX5,APOA5,APOC3,CFTR,DEGS1,EDN2,F2,IGF2,IL10,LIPC,LPL,MIF,NR1H3,OXT,PTGS2,VTN
