# Supplementary material for: Methylation Profiles Reveal Distinct Subgroup of Hepatocellular Carcinoma Patients with Poor Prognosis
Source: PLoS One. 2014 Aug 5;9(8):e104158. doi: 10.1371/journal.pone.0104158 (PMC4122406; doi:10.1371/journal.pone.0104158)
Supplement: Table S9 — Potential upstream regulators predicted by Ingenuity® knowledge base. Z-score was computed based on the direction change of gene expression in input dataset. Overlap p-value tests the probability of having the targets of upstream regulator in our input dataset by chance. (PDF) [file pone.0104158.s014.pdf]

Table S9. Potential upstream regulators predicted by Ingenuity® knowledge base. Z-score was computed based on the direction change of gene expression in input dataset. Overlap p-value tests the probability of having the targets of upstream regulator in our input dataset by chance.

Upstream Regulator	Regulator Type	Activation z-score	p-value of overlap	No. of potentially regulated molecules	Predicted Mechanistic Network
CEBPB	transcription regulator	-1.70	3.37E-08	13	
SP1	transcription regulator		6.96E-07	21	CEBPB,SP1
STAT3	transcription regulator	0.49	1.09E-06	14	NFKB1,NFkB (complex),RELA,STAT3,TNF
NFkB (complex)	complex	-1.94	2.78E-06	19	CEBPB,IL1B,NFKB1,NFkB (complex),RELA,STAT3,TNF
KIAA1524	other	-0.63	5.50E-06	10	
MGEA5	enzyme		5.52E-06	20	
P38 MAPK	group	0.01	1.04E-05	13	CEBPB,ERK1/2,IL1B,NFKB1,NFkB (complex),P38 MAPK,RELA,SP1,STAT3,TNF
SP3	transcription regulator		1.71E-05	11	HDAC1,NFKB1,SP3
CEBPA	transcription regulator	-2.72	2.28E-05	13	
ESR1	ligand-dependent nuclear receptor	-1.34	2.63E-05	17	ERK1/2,ESR1,HDAC1,NFKB1,NFkB (complex),RELA,SP1,SP3,STAT3
APP	other	0.56	6.02E-05	5	
TNF	cytokine	-1.74	7.63E-05	27	CEBPB,ERK1/2,IL1B,Jnk,NFKB1,NFkB (complex),RELA,SP1,STAT3,TNF
CAMP	other	-0.37	1.11E-04	6	CAMP,CEBPB,IL18,IL1B,NFKB1,NFkB (complex),RELA,STAT3,TNF
HDAC1	transcription regulator		1.48E-04	8	HDAC1,NFKB1,NFkB (complex),RELA,SP3
IL1B	cytokine	0.08	1.71E-04	15	CEBPB,IL18,IL1B,NFKB1,NFkB (complex),RELA
Hsp27	group	-0.15	1.95E-04	5	
POU2F1	transcription regulator		1.95E-04	5	
HNF1A	transcription regulator		2.45E-04	11	
Gm-csf	group	-1.11	2.89E-04	6	CEBPB,Gm-csf,IL1B,NFKB1,NFkB (complex),RELA,STAT3
Lh	complex	-0.69	2.99E-04	16	
IFNG	cytokine	-0.60	3.16E-04	20	CEBPB,IFNG,IL10,IL1B,NFKB1,NFkB (complex),RELA,STAT3
PPARA	ligand-dependent nuclear receptor		3.43E-04	6	
IL5	cytokine	-1.89	3.78E-04	7	
COL18A1	other	0.71	5.32E-04	8	
IL13	cytokine	0.29	5.94E-04	14	
